# Comparative effectiveness of different transarterial embolization therapies alone or in combination with local ablative or adjuvant systemic treatments for unresectable hepatocellular carcinoma: A network meta-analysis of randomized controlled trials

**DOI:** 10.1371/journal.pone.0184597

**Published:** 2017-09-21

**Authors:** Konstantinos Katsanos, Panagiotis Kitrou, Stavros Spiliopoulos, Ioannis Maroulis, Theodore Petsas, Dimitris Karnabatidis

**Affiliations:** 1 Department of Interventional Radiology, Patras University Hospital, School of Medicine, Rion, Greece; 2 Department of Interventional Radiology, Guy's and St. Thomas' Hospitals, NHS Foundation Trust, King's Health Partners, London, United Kingdom; 3 Department of Interventional Radiology, Attikon University Hospital, School of Medicine, Athens, Greece; 4 Department of Liver Surgery, Patras University Hospital, School of Medicine, Rion, Greece; Chang Gung Memorial Hospital Kaohsiung Branch, TAIWAN

## Abstract

**Background:**

The optimal transcatheter embolization strategy for patients with unresectable hepatocellular carcinoma (HCC) remains elusive. We conducted a systematic review and network meta-analysis (NMA) of different embolization options for unresectable HCC.

**Methods:**

Medical databases were searched for randomized controlled trials evaluating bland transarterial embolization (TAE), conventional TACE, drug-eluting bead chemoembolization (DEB-TACE), or transarterial radioembolization (TARE), either alone or combined with adjuvant chemotherapy, or local liver ablation, or external radiotherapy for unresectable HCC up to June 2017. Random effects Bayesian models with a binomial and normal likelihood were fitted (WinBUGS). Primary endpoint was patient survival expressed as hazard ratios (HR) and 95% credible intervals. An exponential model was used to fit patient survival curves. Safety and objective response were calculated as odds ratios (OR) and accompanying 95% credible intervals. Competing treatments were ranked with the SUCRA statistic. Heterogeneity-adjusted effective sample sizes were calculated to evaluate information size for each comparison. Quality of evidence (QoE) was assessed with the GRADE system adapted for NMA reports. All analyses complied with the ISPOR-AMCP-NCP Task Force Report for good practice in NMA.

**Findings:**

The network of evidence included 55 RCTs (12 direct comparisons) with 5,763 patients with preserved liver function and unresectable HCC (intermediate to advanced stage). All embolization strategies achieved a significant survival gain over control treatment (HR range, 0.42–0.76; very low-to-moderate QoE). However, TACE, DEB-TACE, TARE and adjuvant systemic agents did not confer any survival benefit over bland TAE alone (moderate QoE, except low in case of TARE). There was moderate QoE that TACE combined with external radiation or liver ablation achieved the best patient survival (SUCRA 86% and 96%, respectively). Estimated median survival was 13.9 months in control, 18.1 months in TACE, 20.6 months with DEB-TACE, 20.8 months with bland TAE, 30.1 months in TACE plus external radiotherapy, and 33.3 months in TACE plus liver ablation. TARE was the safest treatment (SUCRA 77%), however, all examined therapies were associated with a significantly higher risk of toxicity over control (OR range, 6.35 to 68.5). TACE, DEB-TACE, TARE and adjuvant systemic agents did not improve objective response over bland embolization alone (OR range, 0.85 to 1.65). There was clinical diversity among included randomized controlled trials, but statistical heterogeneity was low.

**Conclusions:**

Chemo- and radio-embolization for unresectable hepatocellular carcinoma may improve tumour objective response and patient survival, but are not more effective than bland particle embolization. Chemoembolization combined with external radiotherapy or local liver ablation may significantly improve tumour response and patient survival rates over embolization monotherapies. Quality of evidence remains mostly low to moderate because of clinical diversity.

**Systematic review registration:**

CRD42016035796 (http://www.crd.york.ac.uk/PROSPERO).

## Introduction

Hepatocellular carcinoma (HCC) is the third leading cause of all cancer-related deaths globally and accounts for 90% of primary liver cancers and approximately 7% of all cancers, representing the fifth most common cancer in men and eighth for women. [1–3] Liver transplantation and surgical resection remain the proposed treatment options for very early and early stage HCC in good surgical candidates. Unfortunately, more than three-quarters of the patients are diagnosed during the intermediate or advanced stages of the disease and considered ineligible for curative resection. [1, 4] In the past, the prognosis of unresectable HCC was poor and its management was limited to systemic pharmacotherapy, external radiotherapy or plain supportive treatments. [5] With the advent of Interventional Oncology that encompasses different percutaneous, image-guided, locoregional therapies, [6, 7] treatment options for unresectable HCC quickly expanded to include transcatheter embolization with or without chemotherapy [8]; i.e. bland transarterial embolization (TAE) [9], conventional transarterial chemoembolization (TACE) [10] or chemoembolization with drug-eluting beads (DEB-TACE) [11]; and percutaneous liver ablation either with chemical agents like alcohol [12], or alternatively with application of radiofrequency (RF) or microwave (MW) energy. [13] Conventional TACE with the transcatheter delivery of a mixture of chemotherapy and embolic material is the current standard of care for unresectable intermediate or advanced stage HCC in patients with preserved liver function. [4, 10] Local radiotherapy with the transarterial delivery of beta-emitting microparticles, currently known as radioembolization (TARE) or selective internal radiation therapy (SIRT) [14, 15], is another emerging treatment for unresectable HCC. In addition, various combinations of locoregional ablative treatments with adjuvant systemic therapies [16, 17] or even external organ radiotherapy have been proposed. [18]

In general, interventional targeted embolization and ablative therapies for the treatment of unresectable HCC aim to increase overall patient survival, while limiting treatment-related side-effects, avoiding untoward complications, and improving the quality of life. [4] Theoretically, this can be accomplished by the inherent advantages of transcatheter (chemo)embolization treatments, which include a minimally invasive approach, enhanced pharmacokinetic profile and intra-tumorous bioavailability due to targeted drug delivery, and presumably more extensive tumour necrosis by combining the ischemic effect of embolization, while sparing surrounding normal liver parenchyma. [8, 19] Moreover, transcatheter embolization treatments do not require general anesthesia or prolonged hospitalization periods. [3, 8]

However, in spite of extensive animal and clinical investigations, and numerous randomized controlled trials (RCT) over the last decades, the optimal embolization treatment strategy for patients with intermediate to advanced stage HCC remains elusive. [7, 8] The authors pursued to perform a mixed treatment comparison with quantitative statistical methods—network meta-analysis (NMA)—of the various transcatheter embolization therapies with or without local ablative or adjuvant systemic treatments for unresectable HCC. Comparative effectiveness of treatments that have or have not been directly compared with each other in head-to-head RCTs can be assessed in a network meta-analysis (NMA) using Bayesian statistics, on the condition that all competing therapies share a common chain or network of evidence. [20, 21] We conducted a Bayesian network meta-analysis of all relevant randomized controlled trials to identify the best treatment option for patients with unresectable intermediate/advanced stage HCC.

## Methods

### Search methods

This systematic review has been registered in the PROSPERO public database (CRD42016035796; http://www.crd.york.ac.uk/PROSPERO). The authors initially collated randomized controlled trials reporting outcomes for unresectable HCC from different transarterial embolization strategies (alone or in combination with other treatments) from previously published relevant meta-analyses. [8, 10, 12, 15, 18, 19, 22–33] Subsequently, electronic searches of PubMed (Medline), EMBASE (Ovid), AMED, Scopus, CENTRAL, the China/Asia On Demand (CAOD) research portal, the PROSPERO and DARE meta-analyses databases as well as online material were performed until June 2017. The terms used included ‘hepatocellular carcinoma’, ‘primary liver cancer’, ‘unresectable’, ‘transcatheter’, ‘embolization’, ‘bland’, ‘chemoembolization’, ‘selective internal radiation therapy’, ‘radioembolization’, ‘radiotherapy’, ‘ablation’, ‘radiofrequency’, ‘alcohol’, ‘TAE’, ‘TACE’, ‘DEB-TACE’, ‘TARE’, ‘SIRT’, ‘sorafenib’, ‘bevacizumab’, ‘drug-eluting’, ‘anti-angiogenic’, ‘randomized’, ‘controlled trial’, and ‘meta-analysis’ along with the pertinent Medical Subjects Headings (MeSH) and combinations thereof with Boolean syntax. Keywords were searched using both British English and American English grammar (e.g. embolisation & embolization). In addition, Interventional Radiology, Medical Oncology and Radiation Oncology peer-reviewed journals in PubMed and Embase were examined. There were no restrictions on language, date or type of publication. KK, PK and SS performed the literature search and data extraction.

### Trial selection and good meta-analysis practice

All steps of the trial selection process complied with the PRISMA (Preferred Reporting Items for Systematic reviews and Meta-Analyses) statement (S1 PRISMA checklist). [34] We searched for and included only RCTs comparing any of the aforementioned endovascular devices with each other, and reporting any of the primary and/or secondary outcome measures as defined below. RCTs were assessed for inclusion in the network meta-analysis (NMA) using a specifically structured question checklist developed in consensus by all authors. Published and unpublished randomised trials with an open-label, single-blind or double-blind design were eligible for inclusion provided that they investigated any type of transcatheter arterial embolization for unresectable hepatocellular carcinoma; with or without chemotherapy, plain or drug-eluting beads, radioactive embolic material; as a stand-alone treatment or in combination with other types of locoregional ablation; chemical or thermal or external radiotherapy; or combined with adjuvant systemic treatments; anti-angiogenic molecules or other agents. RCTs were included provided they reported any of the agreed outcome measures (see endpoints below).

A standardized data extraction form was used to collect the following information from all included trials (by KK, PK and SS): (1) characteristics of the study design methods (randomization, blinding, concealment of allocation, drop-outs, outcome reporting, risk of bias); (2) patient sample size and baseline clinical characteristics (age, gender, tumour size and morphology, liver function, vascular invasion, and performance status); (3) HCC staging according to the Okuda, BCLC, JIS or TNM classification systems; (4) description of active and control interventional treatment (chemotherapy regimen, type of embolic agents, treatment courses, dose and fractionation of radiotherapy, adjuvant anticancer agents, other ablation procedures); and (5) clinical outcomes including overall patient survival, objective response of the treated index tumours, and serious adverse events. Terminology and classification of percutaneous and transcatheter image-guided liver therapies complied with standardized nomenclature and universal reporting criteria proposed by the Society of Interventional Radiology Technology Assessment Committee. [35]

The quality of the RCT trials was assessed independently by two of the authors with the Cochrane Collaboration’s tool for evaluating the risk of bias that examines 7 different methodological items including randomized sequence generation, allocation concealment, blinding of patients and investigators, completeness and selectivity of outcome reporting, and other potential sources of bias. [36] Risk of bias assessment was performed by KK, SS and DK. To help inform healthcare decision making, all analysis methods, reporting quality and interpretation of findings complied with the 26-domain questionnaire of the ISPOR-AMCP-NCP Task Force Report for good practice in indirect treatment comparisons and NMA. [37] Finally, the quality of evidence (QoE) was assessed with Grading of Recommendations Assessment, Development and Evaluation (GRADE) system as adapted for the rating of pooled effect estimates in the case of NMA studies, [38, 39] which considers directness, heterogeneity and imprecision of the mixed treatment comparisons as potential reasons for downgrading of the level of confidence.

### Endpoints

In terms of survival outcome measures, few studies were found to report progression-free survival. Therefore, the primary endpoint was set at overall patient survival that was uniformly reported by all studies and was synthesized on the log-hazard scale as indicated for time-to-event outcomes in cancer studies. [40, 41] Study-specific Hazard Ratios (HRs) and respective variances were retrieved from individual publications or back-calculated from the summary or Kaplan-Meier time-to-event data and quoted log-rank statistics with the equations of Parmar et al. [42] and methods of Tierney et al. [43]. If hazard rates were not available, HR was approximated from event rates under the assumption of constant hazards. Random effects models were fitted to account for clinical diversity and heterogeneity and HRs with 95% credible intervals were calculated.

Treatment effectiveness was assessed by the radiologic response on cross-sectional follow-up imaging as reported by each individual RCT. The effectiveness endpoint was set at Objective response (OR) of the index tumour defined as Complete and Partial Response (CR+PR) according to well-accepted classification systems including the World Health Organization (WHO), [44] the European Association for the Study of the Liver (EASL), [45] the Response Evaluation Criteria In Solid Tumors (RECIST), [46] and modified RECIST (mRECIST) [47] schemes.

All outcome measures of this systematic review were defined according to previously published terminology and accepted reporting criteria for transcatheter therapies for liver malignancies. [35] The safety and toxicity endpoint was set at Serious Adverse Events (SAE) grade 3 and above as defined by the National Cancer Institute Common Terminology Criteria for Adverse Events (CTCAE, version 4.0). [48] All endpoints were analyzed on an intention to treat basis as recommended for reporting and meta-analysis of RCTs. Any disagreements were resolved by consensus.

### Statistical methods

Direct pairwise meta-analyses of head-to-head comparisons were performed using standard frequentist approaches (RevMan 5.2, Cochrane Collaboration). Mixed treatment comparisons of the RCT network were performed with Bayesian inference (WinBUGS 1.4.3, MRC Biostatistics Unit at Cambridge, United Kingdom). Bayesian hierarchical modeling of the present network meta-analysis complied with the NICEDSU (National Institute for Health and Excellence Decision Support Units) guidelines. [49–51] Count statistics of treatment toxicity and objective tumour response were analyzed with a Bayesian random effects model with a binomial likelihood to calculate relative treatment effects expressed as Odds Ratios (OR) between different treatments. Overall patient survival was analyzed with a Bayesian random effects model with a normal likelihood incorporating log hazard ratio statistics from individual trials to calculate Hazard Ratios (HR) between competing treatments. [40] Summary statistics of relative treatment effects are reported as the median and accompanying 95% Credibility Intervals (95% CrI) of the posterior distribution. CrIs serve the same purpose as confidence intervals in frequentist statistics.

In addition, we fitted the respective patient survival curves with an exponential model up to 5 years using absolute survival estimates of conventional TACE, which was the most common comparator and with the largest sample size, as the anchor treatment. Median patient survival (half-life) for each treatment was calculated by combining the fitted hazard rate (exponential decay constant) of the anchor treatment (random effects model) with the pairwise posterior median HR calculated by the Bayesian model for the respective treatment. We also constructed rankograms of cumulative rank probabilities of how each treatment ranks against each other in terms of being the 1st, 2nd, 3rd, etc. best treatment option. We present hierarchies of the effectiveness and safety of competing treatments based on their cumulative rank probabilities and the Surface Area Under the Cumulative Rankograms (SUCRA) as proposed by Salanti et al. [52]

The information size (IS) required for a valid meta-analysis may be assumed to be at least as large as the sample size of a single well-powered RCT designed to confirm or reject the null hypothesis [53, 54]. To assess the adequacy of available information size across different pairwise comparisons that combined direct and indirect evidence within the NMA framework, we performed calculations of the effective sample size for each treatment comparison. We employed the methods proposed by Thorlund and Mills for quantifying sample and information size in NMAs after adjusting for statistical heterogeneity observed in pairwise meta-analyses of individual nodes [55]. Consequently, statistical power and strength of evidence for each treatment comparison may be evaluated by the information fraction (IF; percentage of information size) available for each comparison.

### Heterogeneity, consistency, and meta-regression

Heterogeneity was evaluated with the posterior median of the between-trials standard deviation (σ), [50] while small study effects and publication bias were evaluated by visual inspection of standard and comparison-adjusted funnel plots. [56] Because of conceptual differences in study designs and anticipated diversity in baseline demographics, the observed baseline risk of outcome measures may vary between the reference treatment arms. Baseline risk is a proxy for unmeasured but important patient-level characteristics that may relate to significant clinical heterogeneity. Hence, we extended our analysis to a meta-regression model on trial-specific baseline risk of the control arms to account for the uncertainty and clinical heterogeneity introduced by differences in baseline characteristics of unresectable HCC cohorts. [57] In addition, extensive consistency, sensitivity, and meta-regression analyses were performed to explore heterogeneity and confirm validity as proposed by the ISPOR-AMCP-NCP Task Force. [37, 50] The validity and robustness of NMA depend largely on the distribution of effect modifiers (covariates) not only between studies with the same contrast (i.e. heterogeneity in the case of standard pairwise meta-analysis) but also between different contrasts (i.e. inconsistency between direct and indirect contrast estimates). [58] Any disagreement between the direct evidence available for a specific contrast and the indirect evidence inferred by the rest of the network would give rise to inconsistency. In the case of NMA studies, the risk of network inconsistency is greatly reduced if between-trials heterogeneity is low. [59] To exclude any loop-specific inconsistency and confirm the transitivity assumption, pairwise direct and indirect effect estimates of closed loops of evidence were inspected for any disagreement and the results of the consistency model were compared with those of an alternative unrelated mean effects model without any consistency constraints. [49]

### WinBUGS modeling

Bayesian inference with WinBUGS employs Markov Chain Monte Carlo (MCMC) simulation to calculate the posterior distributions of the interrogated nodes within the framework of the chosen model and likelihood function on the basis of prior assumptions. For the purposes of this analysis, we first fitted a Bayesian hierarchical model for multiple comparisons of different treatment options control best supportive treatment as the reference. Posterior medians (95% CrI) of the point estimates against control treatment were calculated using the freely available NetMetaXL software package [60], and by custom code following the examples of Woods et al. [40] Vague priors were used for all treatment effects and for between-trials heterogeneity variance to avoid bias.

Three Markov chains were compiled and run, while convergence was confirmed with the Brooks—Gelman—Rubin diagnostic tool and by inspection of history plots of monitored nodes. An initial burn-in simulation of 50,000 iterations was discarded and inference of final summary statistics was based on simulation of an additional 100,000 iterations. [51] Global model fit and parsimony was compared between different fitted models to decide on the most accurate model. The goodness of fit was compared with the posterior mean of the total residual deviance and the Deviance Information Criterion (DIC) criterion. Residual deviance must approximate the total number of study arms analyzed in the case of a good model fit the and generally the model with the lowest DIC is preferred. [51] The level of statistical significance was set at α = 0.05 for frequentist inference, while relative treatment effect results associated with 95% CrI that did not cross unity were considered significant in the case of Bayesian inference.

## Results

### Network of evidence

**Fig 1:**
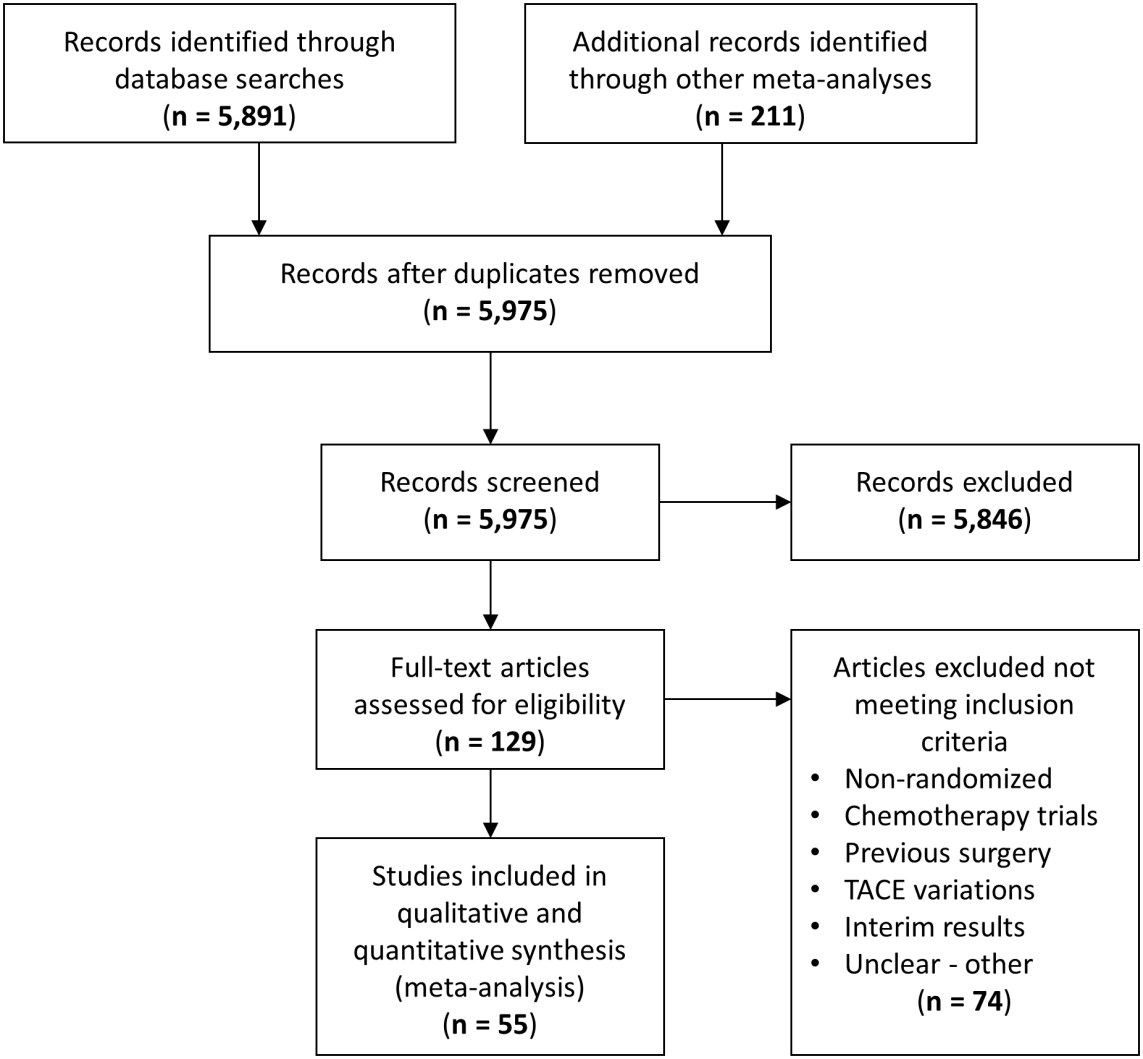
PRISMA flowchart. Trial selection process according to the PRISMA statement.

**Fig 2:**
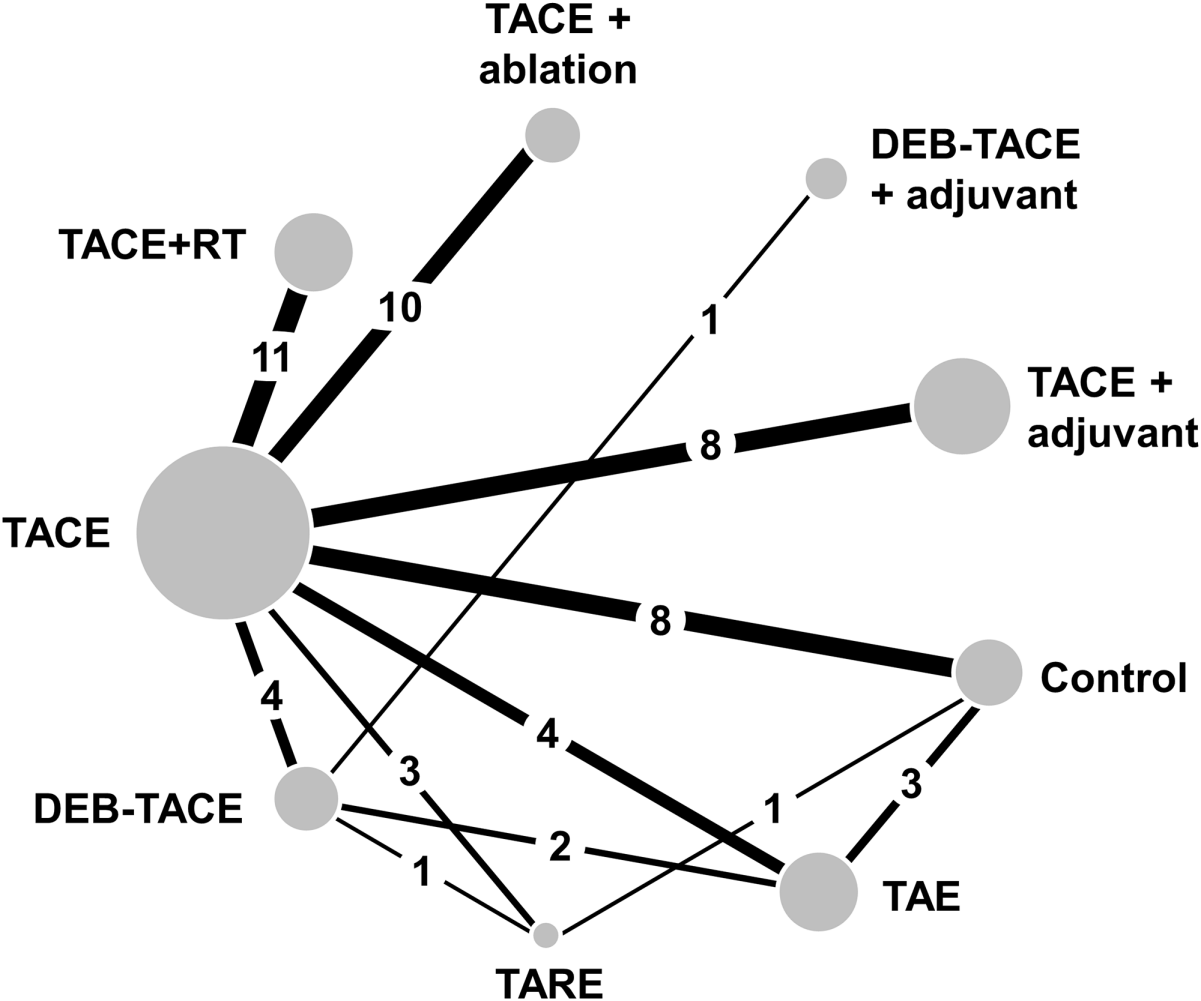
Network of evidence. Straight black lines denote direct head-to-head randomized comparisons. Numbers refer to the number of RCTs with direct comparisons available for each link and the size of circles is proportional to the pooled sample size (patients) available for each treatment node.

Following the PRISMA selection process, 5,975 scientific records were screened for potential inclusion in the network meta-analysis on the basis of their title and abstract (Fig 1). Finally, 55 RCTs (including one three-arm study [61]) published between 1988 and 2017 and reporting on 5,763 patients in total were included and synthesized within a Bayesian framework. The network of evidence involved nine treatment nodes (eight active and one control) and was well connected with conventional TACE as the most common comparator (Fig 2). Four treatment nodes referred to different types of trans-arterial embolization therapy alone (conventional TACE, or DEB-TACE, or TARE, or bland TAE) and another four treatment nodes referred to a combination of transarterial chemoembolization with other locoregional or systemic treatments (TACE and external radiotherapy, or TACE and percutaneous liver ablation, or TACE and adjuvant systemic, or DEB-TACE and an adjuvant systemic agent). Direct evidence was available for 12 comparisons (Table 1); three of them were informed by a single RCT and the rest by more than one RCT (median 3.5; range, 1–11 trials).

**Table 1:** Included randomized controlled trials and baseline patient demographics and index tumour characteristics.


Study & citation	Year	Patients (n)	Age (years)	Male gender (%)	Child-Pugh A/B (#Okuda)	PS (0/1) or KPS	Median stage	Multinodular or diffuse		Follow-up (years)
**Conventional transarterial chemoembolization (TACE) versus best supportive treatment (BST) [n = 8]**										
Groupe d’Etude [62]	1995	96	64y	96%	100% / 0%	NA	NA	59%		4 years
Madden et al. [66]	1993	50	49y	92%	14% / 68%#	1 (1–3)	Okuda II	NA		5 months
Pelletier et al. [68]	1990	42	65y	88%	26% / 52%#	NA	Okuda II	NA		1 year
Pelletier et al. [67]	1998	73	66y	85%	77% / 23%	58% / 38%	Okuda I	NA		2 years
Lo et al. [64]	2002	79	63y	80%	47%/ 53%#	43% / 44%	Okuda II	60%		3.5 years
Llovet et al. [61] (3-arm)	2002	75	65y	73%	69% / 31%	83% / 10%	BCLC B	72%		4 years
FFCD 9402 et al. [63]	2008	123	64y	87%	71% / 29%	37% / 47%	Okuda I	70%		5 years
Mabed et al. [65]	2009	100	52y	65%	69% / 31%	1 (0–2)	Okuda I	58%		1 year
**Bland transarterial embolization (TAE) versus best supportive treatment (BST) [n = 3]**										
Lin et al. [111]	1988	63	50y	92%	100% (A/B)	NA	NA	NA		2 years
Bruix et al. [110]	1998	80	63y	75%	68% / 32%#	68% / 27%	Okuda I	76%		4 years
Llovet et al. [61] (3-arm)	2002	72	65y	73%	67% / 33%	76% / 16%	Okuda II	76%		4 years
**Transarterial radioembolization (TARE) versus best supportive treatment (BST) [n = 1]**										
Raoul et al. [112]	1994	27	66y	96%	52% / 48%	NA	BCLC B	70%		1 year
**Transarterial radioembolization (TARE) versus conventional transarterial chemoembolization (TACE) [n = 2]**										
Raoul et al. [77]	1997	129	65y	95%	75% / 23%	KPS≥70%	Okuda I	50%		4 years
Kolligs et al. [76]	2015	28	66y	86%	64% / 25%	79% / 21%	BCLC B	68%		2 years
Salem et al. [78]	2016	45	63y	73%	56% / 44%	NA	BCLC A	47%		2 years
**Drug-eluting beads chemoembolization (DEB-TACE) versus conventional transarterial chemoembolization (TACE) [n = 4]**										
Lammer et al. [73]	2009	201	67y	87%	83% / 17%	77% / 23%	BCLC B	42%		6 months
Sacco et al. [74]	2011	67	70y	67%	81% / 19%	100% / 0%	BCLC A	34%		3.5 years
Malenstein et al. [75]	2011	30	62y	83%	93% / 7%	63% / 30%	BCLC B	63%		1 month
Golfieri et al. [72]	2014	177	69y	76%	86% / 24%	74% / 26%	BCLC B	54%		2 years
**Bland transarterial embolization (TAE) versus conventional transarterial chemoembolization (TACE) [n = 4]**										
Chang et al. [69]	1994	46	64y	93%	65% / 35%	NA	NA	57%	2 years	
Kawai et al. [70]	1991	286	62y	85%	73% / 24%	52% / 26%	NA	NA	3 years	
Meyer et al. [9]	2013	86	63y	86%	83% / 17%	67% / 20%	BCLC B	67%	3 years	
Yu et al. [71]	2014	90	65y	80%	81% / 19%	66% / 31%	BCLC B	52%	4 years	
**Drug-eluting beads chemoembolization (DEB-TACE) versus bland transarterial embolization (TAE) [n = 2]**										
Malagari et al. [108]	2010	84	70y	77%	58%/ 42%	64% / 36%	NA	38%	1 year	
Brown et al. [107]	2016	101	67y	77%	85% / 15%	86% / 14%	BCLC B	60%	6 years	
**Drug-eluting beads chemoembolization (DEB-TACE) versus transarterial radioembolization (TARE) [n = 1]**										
Pitton et al. [109]	2015	24	71y	75%	79% / 21%	100% / 0%	BCLC B	96%	3 years	
**Conventional transarterial chemoembolization (TACE) plus systemic therapy versus conventional transarterial chemoembolization (TACE) [n = 8]**										
Sansonno et al. [84]	2012	80	73y	60%	100% / 0%	61% / 39%	NA	45%	21 months	
Kudo et al. [81]	2011	458	70y	75%	100% / 0%	88% / 12%	NA	27%	3 years	
Britten et al. [79]	2011	30	59y	50%	93% / 7%	80% / 20%	BCLC B	27%	5 years	
Pinter et al. [83]	2015	32	61y	91%	69% / 31%	100% / 0%	BCLC B	59%	46 months	
Wang et al. [85]	2015	125	55y	85%	85% / 15%	82% / NA	BCLC B	33%	40 months	
Li et al. [82]	2009	216	48y	70%	91% / 9%	76% / NA	Okuda I	55%	3 years	
Kudo et al. [17]	2014	502	58y	84%	94% / 5%	80% / 20%	BCLC B	65%	3 years	
Inaba et al. [80]	2013	101	NA	81%	84% / 16%	93% / 7%	BCLC B	57%	3 years	
**Drug-eluting beads chemoembolization (DEB-TACE) plus adjuvant systemic versus Drug-eluting beads chemoembolization (DEB-TACE) [n = 1]**										
Lencioni et al. [16]	2016	307	64y	85%	100% / 0%	100% / 0%	BCLC B	100%	800 days	
**Conventional transarterial chemoembolization (TACE) plus tumour ablation versus conventional transarterial chemoembolization (TACE) [n = 9]**										
Yang et al. [93]	2008	35	58y	74%	60% / 29%	NA	NA	66%	2 years	
Bartolozzi et al. [86]	1995	53	66y	77%	47% / 53%	NA	NA	40%	3 years	
Becker et al. [87]	2005	52	64y	79%	75% / 25%	NA	Okuda I	37%	30 months	
Wu et al. [90]	1998	102	55y	94%	78% / 17%	NA	NA	NA	3 years	
Xu et al. [91]	2002	45	NA	NA	100% / 0%	NA	NA	0%	3 years	
Yamamoto et al. [92]	1997	100	NA	87%	37% / 42%	NA	JIS II-IV	52%	3 years	
Liu et al. [88]	2009	78	53y	NA	86% / 14%	NA	BCLC C	NA	2 years	
Wang et al. [89]	2007	83	58y	80%	80% / 20%	NA	TNM III	NA	1 year	
Zhao et al. [94]	2011	47	NA	NA	NA	NA	BCLC C	NA	3 years	
Huang et al. [95]	2016	120	60y	77%	100% (A/B)	NA	BCLC B	0%	5 years	
**Conventional transarterial chemoembolization (TACE) plus external radiotherapy versus conventional transarterial chemoembolization (TACE) [n = 11]**										
Xue et al. [103]	1995	41	NA	NA	100% (A/B)	NA	TNM II	NA	1 year	
Leng et al. [96]	2000	75	NA	NA	100% / 0%	KPS≥65%	TNM III	NA	3 years	
Wang et al. [101]	2000	40	37y	92%	85% (A/B)	NA	TNM III	30%	5 years	
Peng et al. [99]	2000	91	NA	NA	NA	NA	TNM II	NA	5 years	
Li et al. [97]	2003	82	51y	NA	61% / 39%	NA	NA	NA	3 years	
Zhao et al. [105]	2006	96	53y	63%	100% / 0%	KPS≥70%	TNM I	NA	3 years	
Shang et al. [100]	2007	76	52y	NA	100% (A/B)	KPS≥70%	TNM I	NA	3 years	
Xiao et al. [106]	2008	60	NA	NA	65% / 35%	KPS≥70%	TNM II	NA	3 years	
Liao et al. [98]	2010	48	NA	NA	71% / 29%	NA	TNM III	NA	3 years	
Wang et al. [102]	2006	108	54y	NA	100% (A/B)	KPS≥65%	TNM III	8%	3 years	
Zhang et al. [104]	2012	259	53y	NA	100% (A/B)	NA	BCLC C	NA	2 years	

TACE was investigated versus Control symptomatic treatment in 8 studies [61–68], versus bland TAE in 4 studies [9, 69–71], versus DEB-TACE in 4 studies [72–75], versus TARE in 3 studies [76–78], versus TACE combined with adjuvant systemic agents in 8 studies [17, 79–85], versus TACE combined percutaneous liver ablation in 10 studies [86–95], and versus combined TACE and external radiotherapy in 11 studies [96–106]. In addition, DEB-TACE was compared directly with TAE in 2 studies [107, 108], with TARE in 1 RCT [109], and with DEB-TACE plus systemic sorafenib in 1 RCT [16]. Finally, TAE alone was compared with Control treatment in 3 studies [61, 110, 111], and TARE with Control in 1 study [112]. There were 3 high-quality RCTs with low risk of bias; the rest of the studies had unclear (at least one unclear domain) to high (at least one high-risk domain) risk of bias according to the COCHRANE tool for risk of bias assessment. The latter was caused by performance bias (absent or unclear blinding of participants and personnel) or detection bias (blinded outcome assessment) in the majority of the studies.

Fifty-one out of the 55 studies recruited patients with unresectable hepatocellular carcinoma classified as intermediate to an advanced stage (i.e. BCLC stage B-C, Okuda stage I-II, or AJCC TNM stage II-III) and 4 studies included unresectable early stage HCC [74, 78, 100, 105]. All studies included patients with preserved liver function (Child-Pugh A and B) and with a predominantly male gender (range, 50–96%). Good performance status (PS: 0–1 or KPS≥65%) was reported in most of the cases and the percentage of randomized patients with a multinodular or diffuse type of HCC varied widely (median, 57%; IQR, 39–67%; max 100%). Fourteen out of the 55 studies reported inclusion of variable rates of patients with documented portal vein thrombosis (range, 2–100%). A detailed description of baseline patient demographics and clinical characteristics is provided in Table A in S1 Appendix.

In the TACE treated arms, conventional transarterial chemoembolization was performed with a lipiodol emulsion of a single chemotherapy agent (doxorubicin [61, 68, 70, 73–75, 78, 83, 86], or epirubicin [63, 66, 72, 76, 80], or cisplatin [9, 62, 64, 67, 69, 71, 77, 82], or mitomycin [87], or a combination chemotherapy regimen [65, 79, 81, 84, 85, 89, 93, 95, 97, 99–106], and was most often followed by gelfoam or other particle embolization of the primary feeding vessels. Meyer et al. performed cisplatin infusion first followed by particle embolization 4–6 hours later [9]. In case of TAE, bland embolization was performed with gelfoam and/or microparticles (microspheres) [9, 61, 69, 70, 107, 108, 110, 111] or alcohol [71]. DEB-TACE involved transcatheter delivery of doxorubicin-eluting DC beads [16, 72–75, 107–109], and TARE of a beta-emitter including ^131^I-labeled Lipiodol [77, 112] or Yttrium-90 microparticles [76, 78, 109]. Adjunctive systemic agents included sorafenib [16, 81, 84], brivanib [17], bevacizumab [79, 83], arsenic trioxide [85], TSU-68 [80], IFN-a [82]. Locoregional liver ablation was reported by means of multiple sessions of radiofrequency ablation (RFA) [88, 89, 93, 94] or percutaneous ethanol injection (PEI) [86–88, 90–92] or argon-helium cryoablation [95]. Finally, external radiotherapy was delivered by 3D conformal [97, 98, 100, 104–106] or moving stripe fractionated protocols [99, 101–103]. Active and control treatment protocols are described in detail in Table B in S1 Appendix. Median follow-up was 3 years on a trial basis (interquartile range, 2.0–3.5 years; max 6.0 years).

### Patient survival

**Fig 3:**
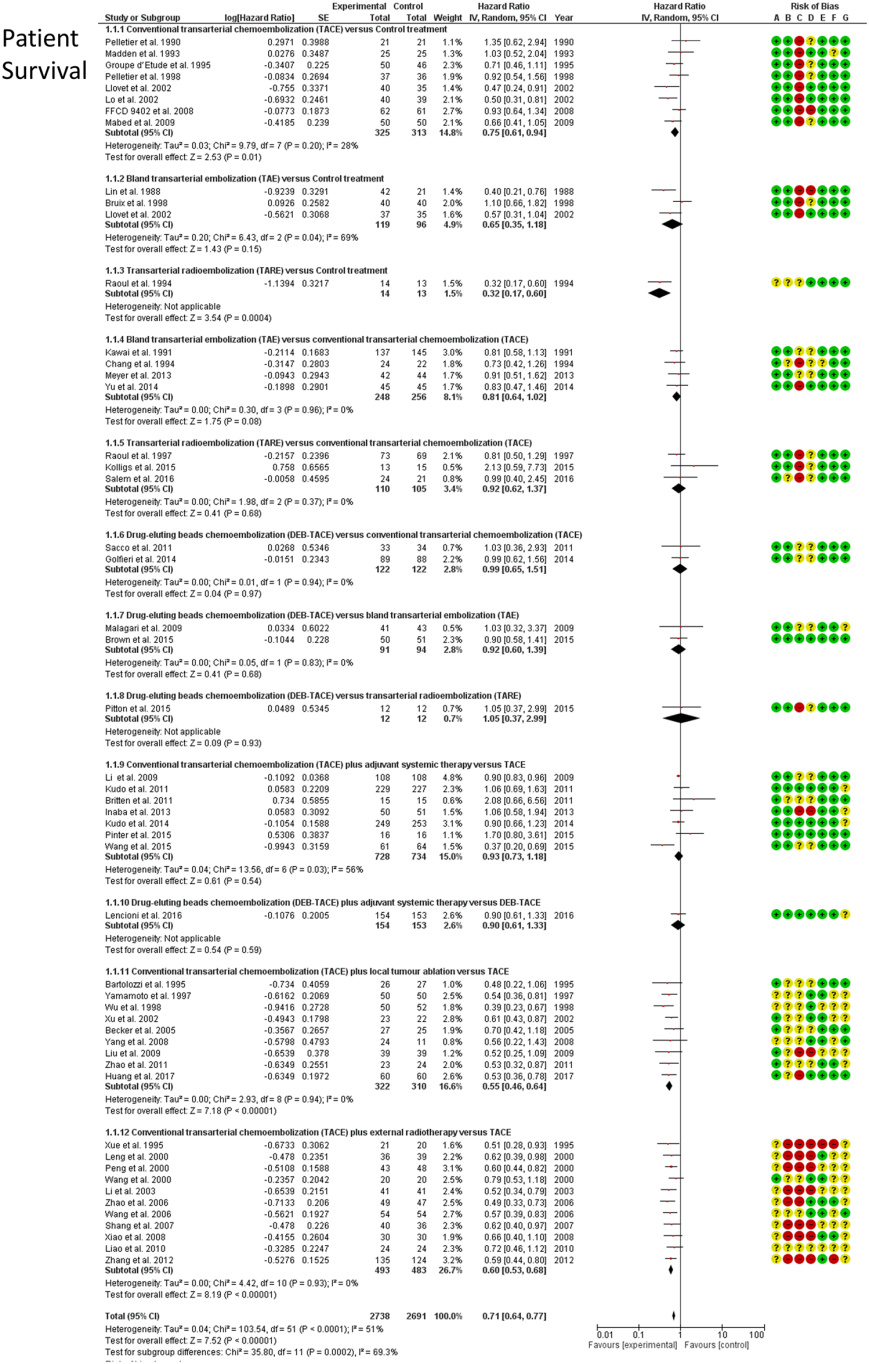
Patient survival. Forest plots (random effects) of direct frequentist analyses (RevMan, Cochrane). Risk of bias assessment by the Cochrane Collaboration tool is presented as well.

**Fig 4:**
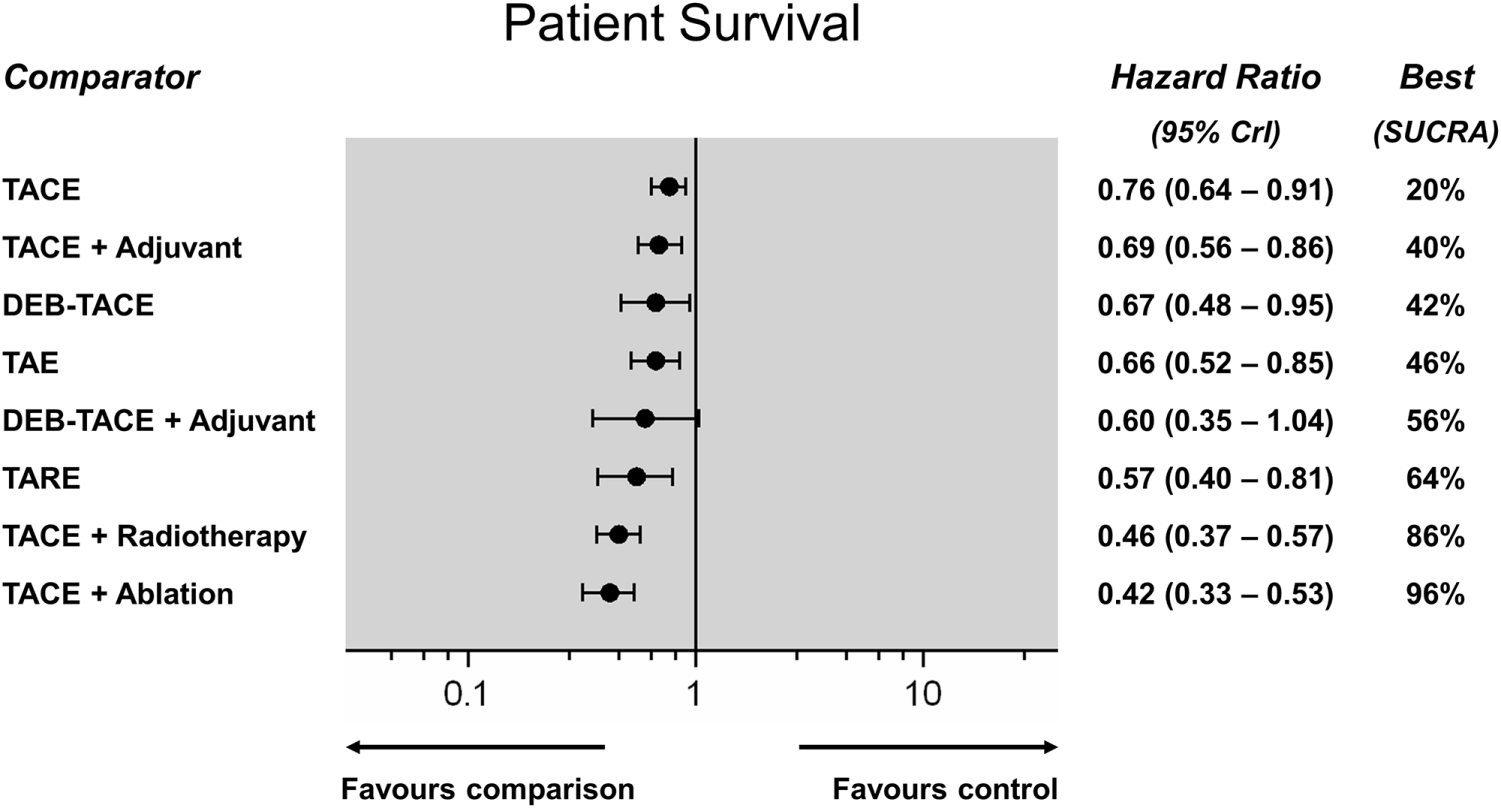
Patient survival network meta-analysis (Random effects forest plot). Different treatments are reported in order of efficacy ranking according to the SUCRA statistic. Black circles denote the posterior median and the black lines denote the associated 95% CrI. Numbers represent hazard ratios (HR) and 95% CrIs. The combination of TACE and ablation was found to be the most effective treatment (SUCRA 95%).

Survival outcomes were reported by 51 RCTs (incl. one 3-arm) reporting on 5,394 patients and 12 direct comparisons in total. Direct meta-analyses (Fig 3) confirmed a significant survival benefit of TACE over best supportive therapy (HR: 0.76; 95%CI: 0.64–0.91) and a similar survival benefit between TAE and TACE (HR: 0.87; 95%CI: 0.71–1.07). In addition, TACE performed worse than TACE plus radiotherapy (HR: 0.60; 95%CI: 0.53–0.69) and TACE plus ablation (HR: 0.54; 95%CI: 0.46–0.65). The NMA synthesis showed that all embolization treatments achieved a significant survival benefit over control except DEB-TACE with adjuvant sorafenib (HR range, 0.42–0.76). Fig 4 shows a hierarchy of different treatments according to the SUCRA statistic and the respective Hazard Ratios (HR). TACE, DEB-TACE, TARE, and adjunctive systemic agents (combined with TACE or DEB-TACE) did not confer a survival benefit over bland TAE. TACE combined with external radiation therapy (SUCRA 86%), or percutaneous tumour ablation (SUCRA 96%), were the most effective treatment strategies. NMA heterogeneity was low (σ = 0.06; 95%CrI: 0.001–0.17). A league table of all pairwise survival comparisons from the NMA synthesis is provided in the S1 Appendix.

### Survival model

**Fig 5:**
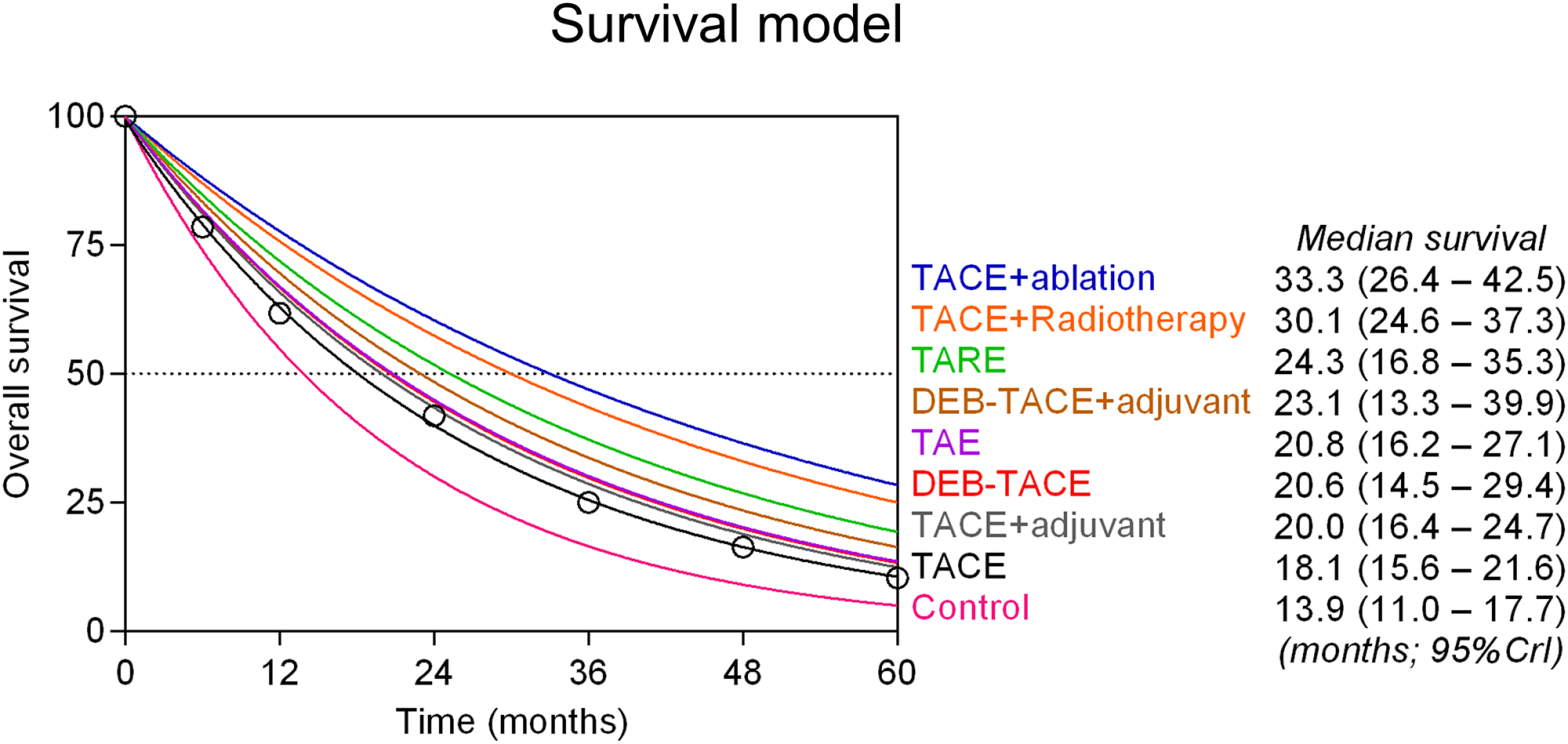
Survival model. Projected survival curves for each treatment were fitted with an exponential model up to 5 years. Conventional TACE was the most common comparator in the overall network of evidence and was used as the anchor treatment because it had the largest sample size. Absolute survival estimates of TACE at different time points were calculated with a standard random effects proportional model weighted by patient sample for each trial (black circles). Median patient survival (half-life) for each treatment was then calculated by combining the fitted hazard rate (exponential decay constant) of the anchor treatment with the pairwise posterior median HR calculated by the Bayesian model for the respective treatment.

The fitted exponential survival model is shown in Fig 5 (posterior median of survival projections; 95% CrIs). Conventional TACE was the most common comparator node (43 out of the 51 RCTs reporting patient survival) and was used as the anchor treatment (least squares non-linear fit *R*^*2*^ = 0.999) for calculating expected median survival outcomes for each of the other treatment options. Median survival period in case of control best supportive treatment was 13.9 months (95%CI: 11.0–17.7) and increased to 18.1 months (95%CI: 15.6–21.6) in the case of TACE, 20.6 months (95%CI: 14.5–29.4) with DEB-TACE, and 20.8 months (95%CI: 16.2–27.1) with bland TAE. Adjuvant systemic agents did not provide any significant survival benefit over transarterial therapies. Median survival increased to 24.3 months (95%CI: 16.8–35.3) in the case of TARE. Projected median survival exceeded 30 months when conventional TACE was combined with external radiotherapy (30.1 months; 95%CI: 24.6–37.3) or with percutaneous liver tumour ablation (33.3 months; 95%CI: 26.4–42.5).

### Objective response

**Fig 6:**
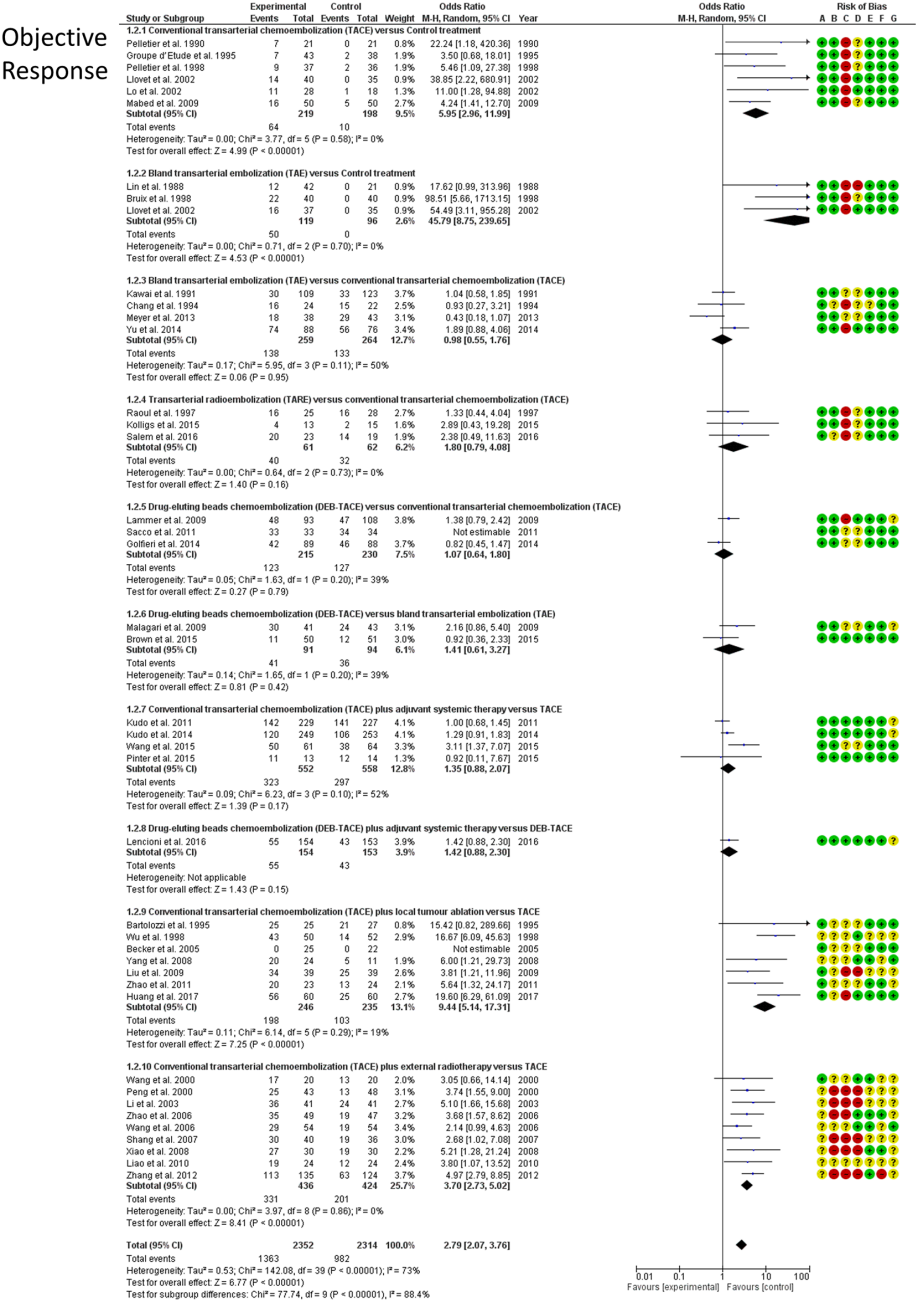
Objective response. Forest plots (random effects) of direct frequentist analyses of patient survival (RevMan, by Cochrane). Risk of bias assessment by the Cochrane Collaboration tool is presented as well.

**Fig 7:**
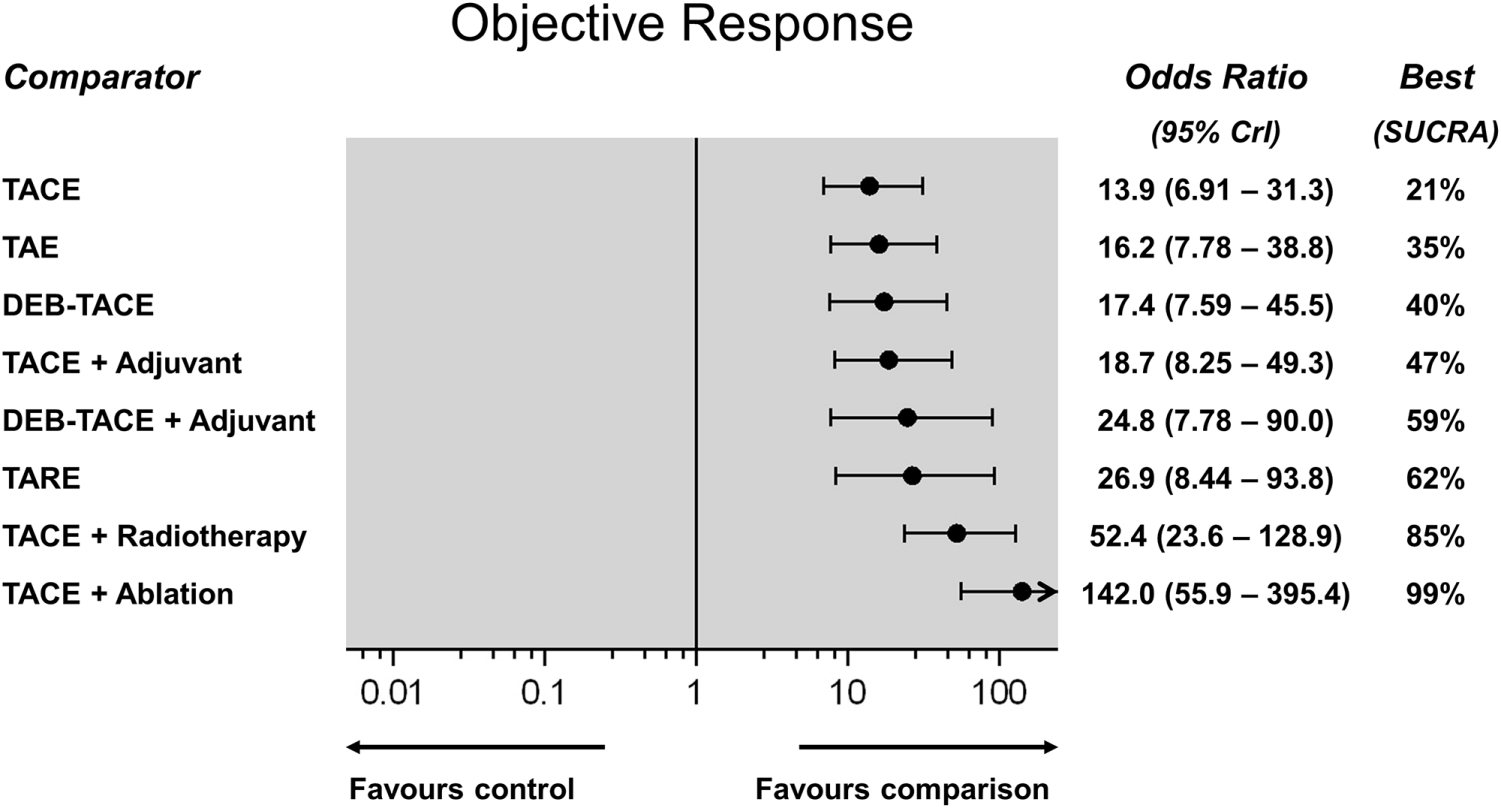
Objective response network meta-analysis (Random effects plot). Different treatments are reported in order of efficacy ranking according to the SUCRA statistic. Black circles denote the posterior median and the black lines denote the associated 95% CrI. Numbers represent odds ratios (OR) and 95% CrIs. The combination of TACE and ablation was found to be the most effective treatment (SUCRA 99%).

**Fig 8:**
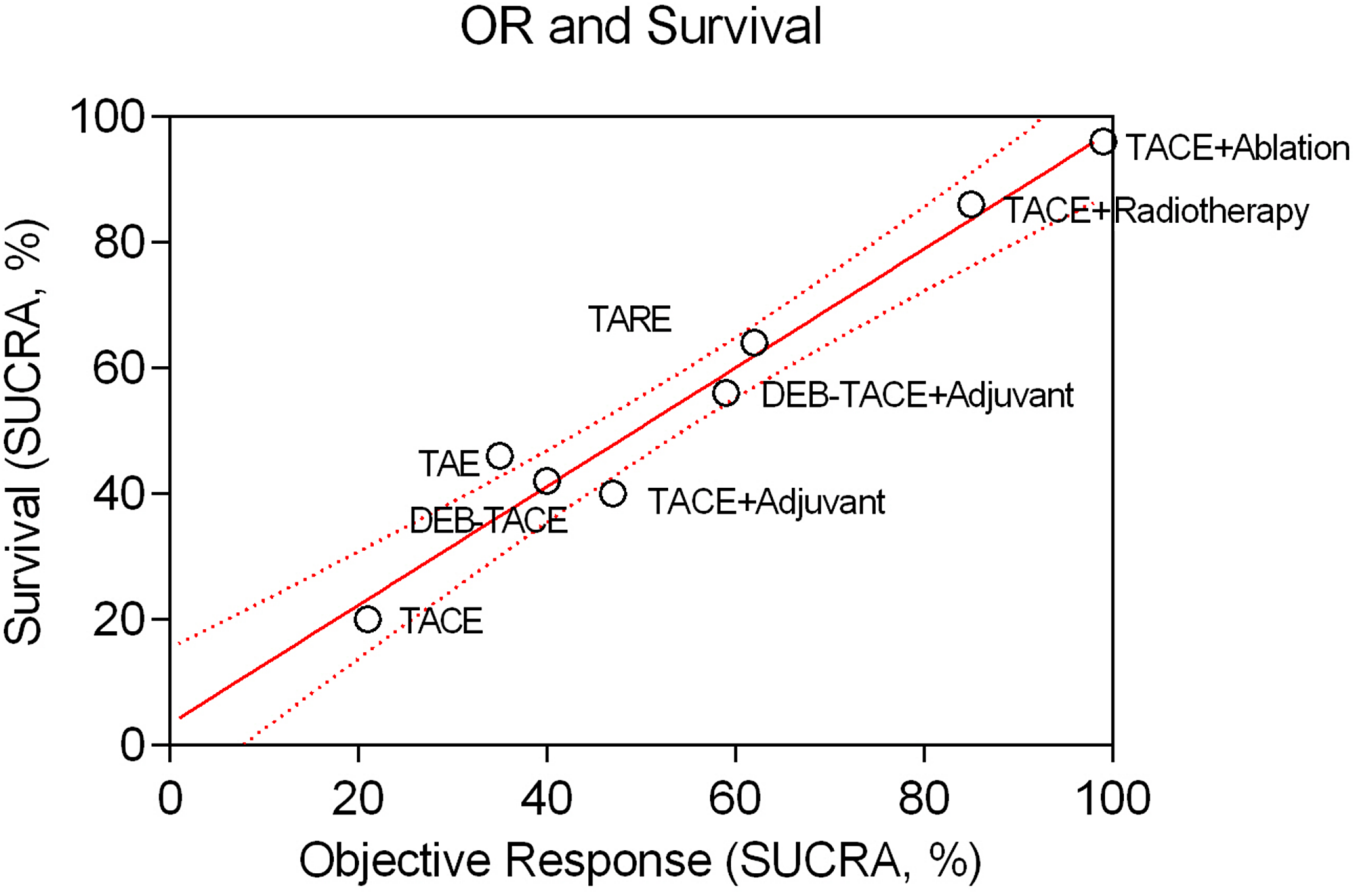
Patient survival and objective response. Two-dimensional ranking of different treatments according to patient survival (y-axis) and objective response (x-axis) based on the cumulative rank probabilities (SUCRA; %). Note the linear correlation (linear regression fit *R*^*2*^ = 0.926) between the 2 outcome metrics.

Rates of the objective response of the treated tumour lesions were reported by 41 RCTs including 4,669 patients and informing 10 direct treatment comparisons. According to direct meta-analyses (Fig 6), both TACE (OR: 5.95; 95%CI: 2.96–11.99) and TAE (OR: 45.8; 95%CI: 8.75–239.7) demonstrated a strong response rate over control treatment. In line with the survival analysis, objective response was also better in case of TACE combined with radiotherapy (OR: 3.7; 95%CI: 2.7–5.0) or ablation (OR: 9.44; 95%CI: 5.14–17.3) over TACE alone. In the NMA analysis, all embolization treatments achieved a significant tumour response. Fig 7 shows a hierarchy of comparative treatment effectiveness according to the SUCRA statistic. Combinations of conventional TACE with external radiation therapy (SUCRA 85%) or percutaneous tumour ablation (SUCRA 99%) were the most effective treatment options. TACE, DEB-TACE, TARE and adjunctive systemic agents (combined with TACE or DEB-TACE) did not improve the objective response of treated tumours compared to bland embolization alone (TAE). TACE with adjunctive ablation achieved a significantly better objective tumour response compared to all other embolization mono- or combination therapies (OR range, 2.17–10.2; league table in S1 Appendix). NMA heterogeneity was low (σ = 0.29; 95%CrI: 0.03–0.63). Comparative effectiveness results of overall patient survival were corroborated by the hierarchical SUCRA results of tumour objective response with high correlation between the two outcome measures (linear regression fit *R*^*2*^ = 0.959 –Fig 8).

### Serious adverse events

**Fig 9:**
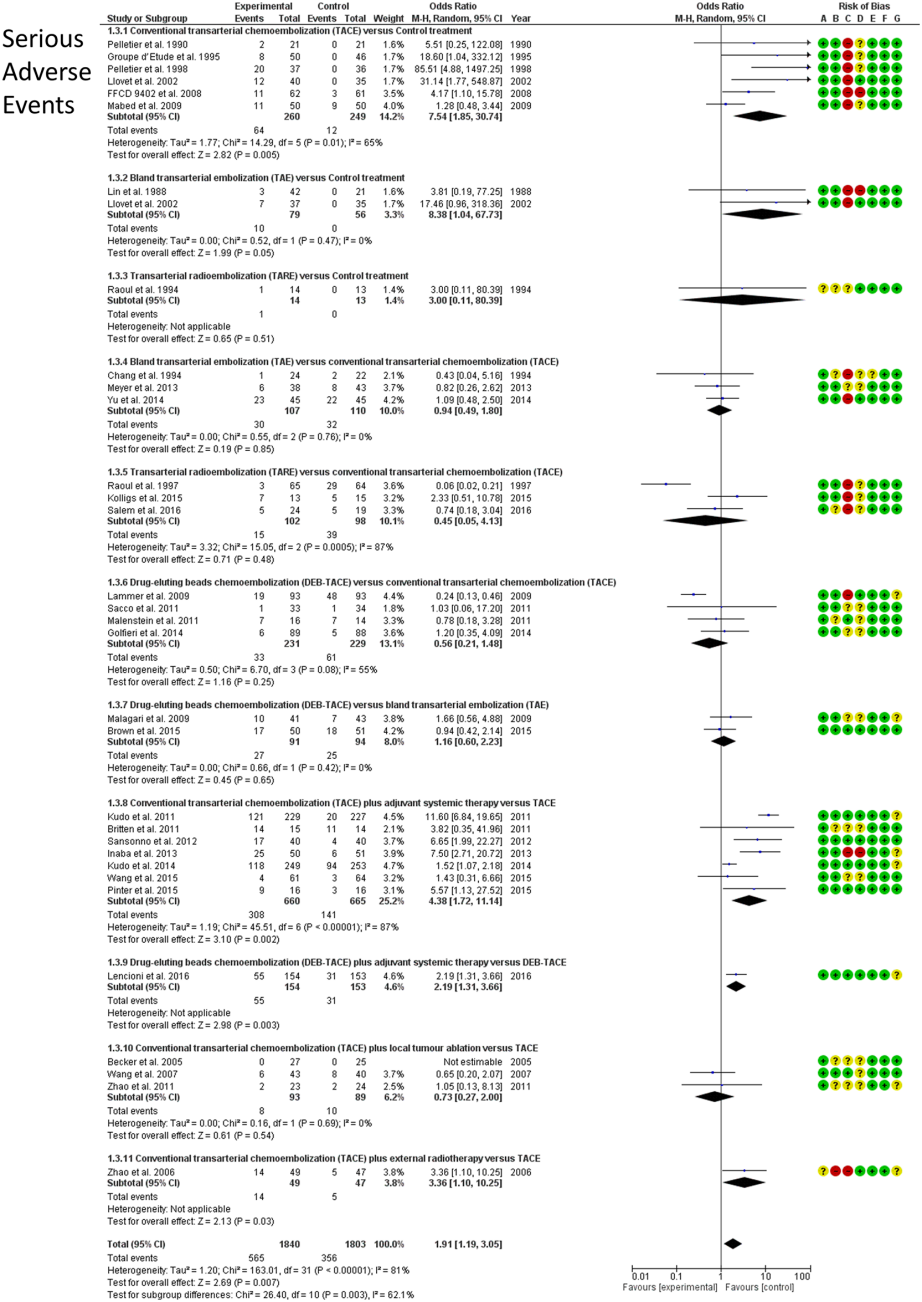
Serious adverse events. Forest plots (random effects) of direct frequentist analyses of patient survival (RevMan, Cochrane). Risk of bias assessment by the Cochrane Collaboration tool is presented as well.

**Fig 10:**
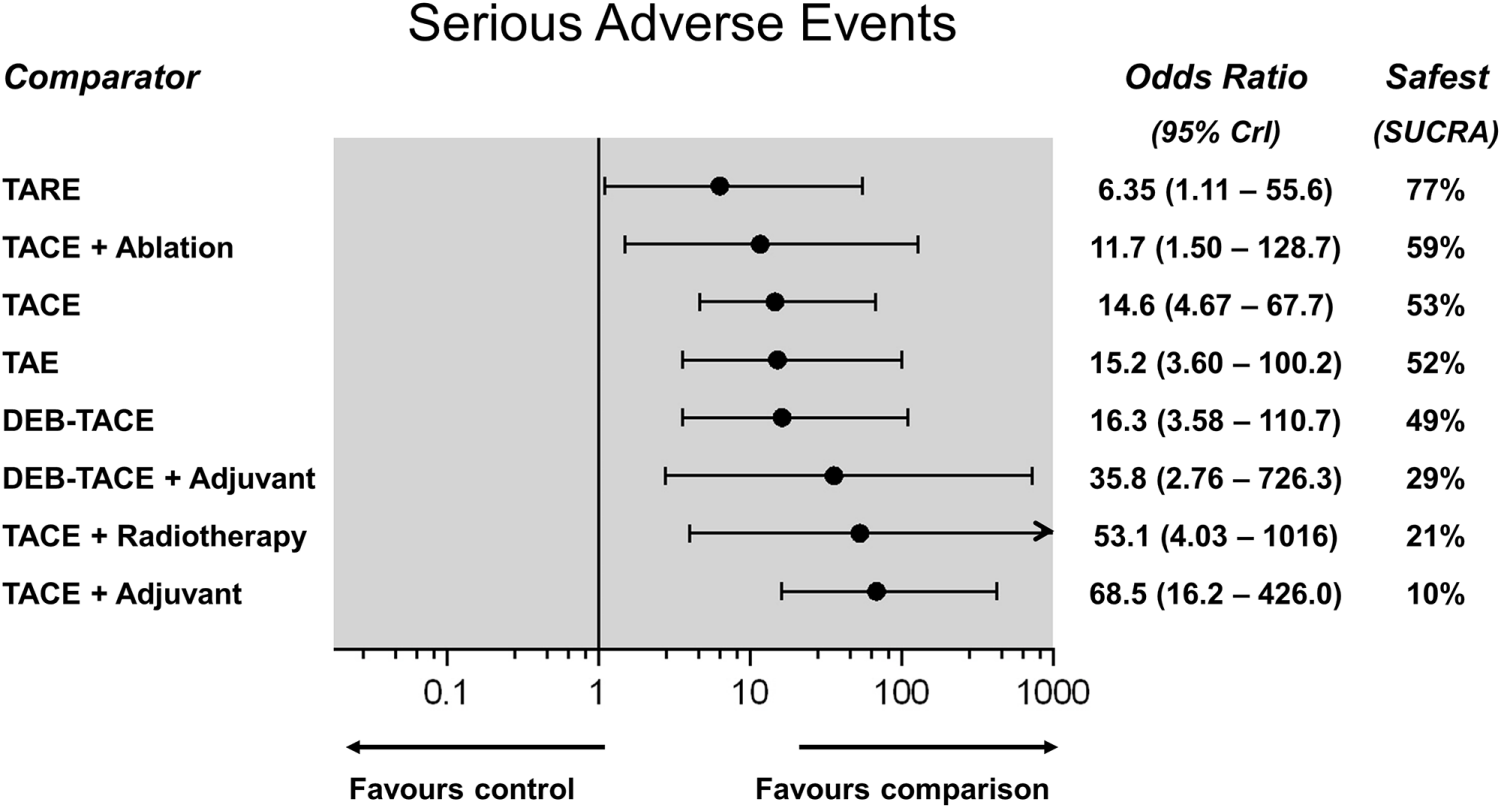
Serious adverse events network meta-analysis (Random effects forest plot). Different treatments are reported in order of safety ranking according to the SUCRA statistic. Black circles denote the posterior median and the black lines denote the associated 95% CrI. Numbers represent odds ratios (OR) and 95% CrIs. TARE was found to be the safest treatment (SUCRA 90%).

Treatment-related serious adverse events (SAE) were reported by 32 RCTs including 3,610 patients for 11 direct treatment comparisons (Fig 9). Safety ranking of different embolization therapies on the basis of cumulative rank probabilities (SUCRA, %), along with the respective ORs (95%CrI) against control as a reference, are shown in Fig 10. TARE was the safest treatment (SUCRA 77%), however, all examined therapies were associated with a significantly higher risk of SAE compared to control (OR range, 6.35–68.5). Most of the other pairwise comparisons showed no significant differences between different embolization regimes in terms of SAE. TACE combined with adjuvant systemic therapies was the highest-risk treatment (SUCRA 10%—league table in S1 Appendix). Between-trial heterogeneity was low (σ = 1.01; 95%CrI: 0.61–1.64).

### Heterogeneity, consistency, and meta-regression

There was good agreement between the consistency and inconsistency (unrelated mean effects) models, suggesting a robust and homogeneous network of evidence (Table C in S1 Appendix). Between-trial statistical heterogeneity in the random effects Bayesian models was low compared to the respective posterior treatment effects (Table D in S1 Appendix). Consequently, application of a fixed effect Bayesian model produced similar numerical results with slightly tighter credible intervals (League tables in S1 Appendix). However, model fit according to the residual deviance and DIC criteria was better in the case of the random effects analyses and hence those were preferred and presented in the present article (Table D in S1 Appendix). There was no obvious asymmetry at visual inspection of funnel plots to suggest publication bias, except in the case of Objective Response (Funnel plots in S1 Appendix). However, that was not evident any more on the comparison- adjusted funnel plot (OR Funnel plot with comparison-specific adjustments in S1 Appendix). Random effects meta-regression analyses to check for risk modifiers demonstrated only weak non-significant correlations in the majority of the tests. Multinodular HCC was the only variable found to be strongly and significantly related to increased rate of adverse events, as well as of higher rates of radiological response (Table E in S1 Appendix).

### Strength and quality of evidence

**Fig 11:**
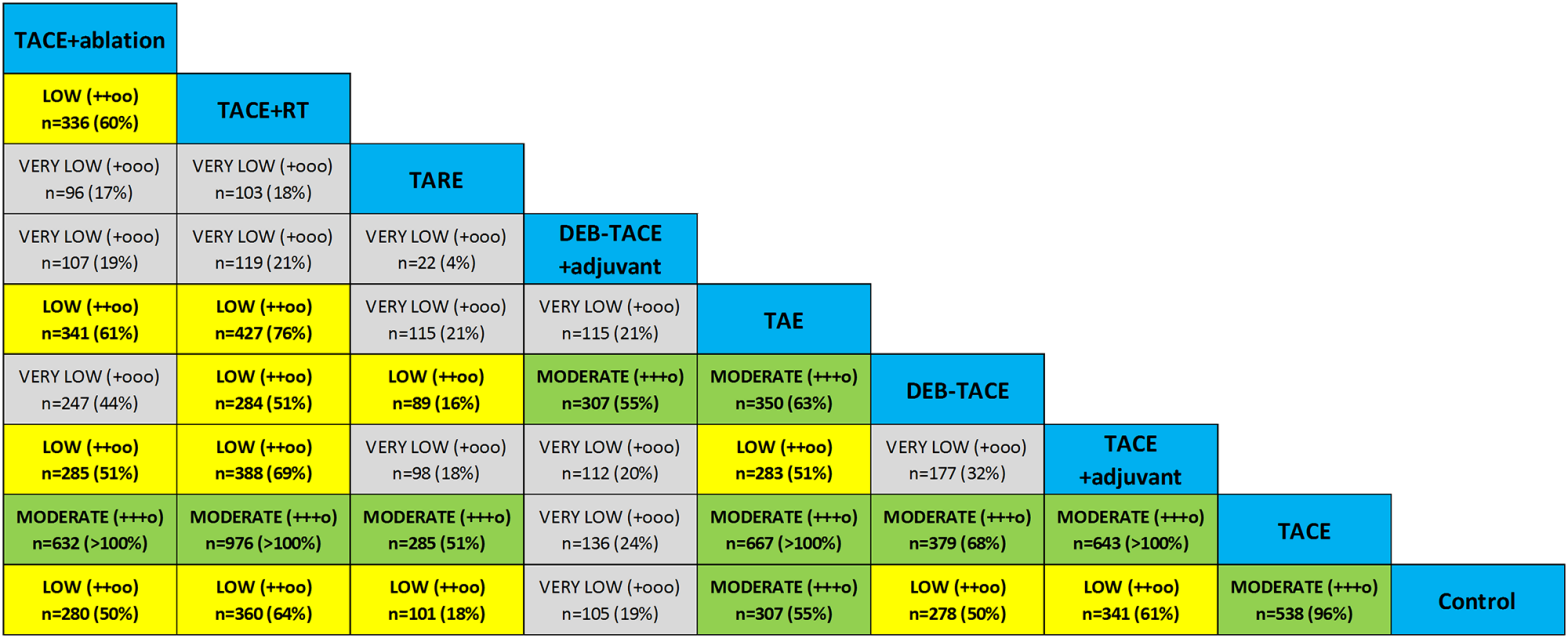
Strength and quality of evidence. QoE was graded as recommended for network meta-analyses on the basis of clinical diversity (between-trial heterogeneity of patient characteristics and/or study design), indirectness (absence of direct randomized comparisons), and imprecision (we chose a threshold of information fraction <50%). Effective sample size n for each comparison is shown along with information fraction (IF; %) in parentheses (compared to n = 560 for a hypothetical well-powered randomized study to detect a survival benefit of HR = 0.70 at 2 years). Color-coded representation of QoE; very low (light gray), low (yellow), moderate (green). There were no cases of high QoE observed.

We calculated a sample size of 560 patients as adequate for the detection of a treatment effect of 30% relative risk reduction of death (HR = 0.7) with a type I error 5% and type II error 20% (power 80%) assuming an average patient survival of 50% at 2 years and a 10% rate of drop-outs or lost to follow-up. Compared to that, the IF was found to be low-to-modeate (range, 4–51%) in case of TARE, and high (range, 50–100%) in all mixed treatment comparisons informed by both direct and indirect evidence. Fig 11 summarizes the strength (effective sample size and IF) and QoE according to the GRADE system for all treatment comparisons in the present NMA.

The GRADE system for assessing quality of evidence considers directness, heterogeneity and imprecision of the mixed treatment comparisons as potential reasons for downgrading the level of confidence in NMA results [113]. We have found no inconsistency and statistical heterogeneity was generally low in the present NMA, however, clinical diversity was evident in the baseline demographics of different RCTs. Hence, in the current analysis, QoE was first downgraded universally because of between-trial diversity in terms of baseline patient characteristics and type and mixture of antineoplastic and/or embolic agents used (Tables A and B in S1 Appendix). Second, it was further downgraded in certain comparisons because of the absence of direct comparative evidence (indirectness).

To evaluate imprecision, we gauged the effective sample size and information fraction of each comparison. We considered an IF<50% as a measure of weaker evidence and potential imprecision; hence, QoE was further downgraded to very low in the relevant comparisons. Overall, there was moderate QoE with sufficient information size when comparing TACE+ablation, TACE+RT, TACE+adjuvant systemic agents and TAE, over TACE alone. Information was also strong enough with moderate QoE in the case of TARE versus TACE, in the cases of TAE compared with control or TACE or DEB-TACE, and in the case of TACE over control treatment (Fig 11).

## Discussion

Contrary to a standard meta-analysis that pools studies comparing a certain pair of treatments, network meta-analysis (NMA) is an established methodology capable of inferring the high level of evidence about any number of treatments by combining direct and indirect randomized comparative research into a single unified analysis while respecting randomization of individual clinical studies. [114] To our knowledge, this is the first comprehensive mixed treatment comparison analysis evaluating the safety and effectiveness of different transarterial embolization therapies either alone or in combination with local ablative or adjuvant systemic treatments for unresectable hepatocellular carcinoma. Most of the patients with hepatocellular carcinoma are diagnosed late at the intermediate-advanced stages of the disease and are ineligible for potentially curative treatments like liver transplantation, resection or curative thermal ablation. According to GIDEON, the largest global observational registry of unresectable HCC to date including more than 3,200 cases, more than half of all HCC patients receive TACE as their primary treatment mode [115]. A lipiodol emulsion of an anticancer agent; usually doxorubicin; followed by gelfoam or other particle embolization remains the most popular form of TACE [8]. Adoption of TACE with an oil emulsion of antineoplastic agents has been primarily driven by early RCTs of bland TAE or TACE versus conservative management more conducted than 10 years ago [8, 61, 64, 67, 68, 110, 111]. However, not only new treatments have emerged like DEB-TACE or TARE or combined locoregional treatments, but above all guideline-recommended therapy for unresectable HCC remains controversial. The ESMO-ESDO guidelines advocate TACE for large or multinodular HCC with good liver function [116], whereas the Canadian CEPO (Comité de l'évolution des pratiques en oncologie) recommends TACE as the standard of care for palliative treatment of eligible HCC patients, but specifically advises against the use of TAE or TARE [117]. In the meantime, a recent heavily disputed Cochrane meta-analysis questioned the firmness of evidence supporting either TAE or TACE in unresectable HCC in general [33]. Hence, the survival benefit of transarterial embolization therapies for unresectable HCC is still under dispute [118].

Most importantly, the present NMA of 55 RCTs comprising more than 5,700 patients has shown that transarterial (chemo)-embolization strategies can confer a clear survival benefit in patients with unresectable HCC by reducing the hazard of death in the range of 24% (in case of TACE) up to 34% (in case of TAE and DEB-TACE). However, surprisingly, none of the transcatheter chemo-embolization options (i.e. TACE and DEB-TACE as standalone treatments or even combined with adjuvant systemic agents) was any better than traditional bland transarterial embolization (TAE). The above findings had a large information size and moderate QoE being supported by direct evidence by 3 trials examining TAE versus best supportive therapy (publication date 1988–2002) [61, 110, 111], 4 trials testing TAE versus TACE (1994–2014) [9, 69–71], and 2 trials comparing TAE versus DEB-TACE (2010–2016) [107, 108]. Internal radiation therapy (TARE) produced an even higher survival benefit (43% reduction of the hazard of death) informed by 3 trials [76–78], but its effectiveness was not significantly better than TAE and evidence was informed only by a moderate information size (very low-to- moderate QoE).

The aforementioned findings, on one hand, support the notion that ischemic necrosis induced by transcatheter embolization of the tumour feeding arteries is the primary mode of therapy in HCC and on the other hand question the need for the widely employed use of antineoplastic agents (most often doxorubicin) as part of the majority of HCC embolization regimens. Neoangiogenesis is a well-known hallmark of hepatocellular carcinoma [119], and hepatic transarterial embolization induces virtually immediate tumour cell death evident on imaging within 24hours [107]. The addition of chemotherapy has been long thought to allow for enhanced intratumoral drug delivery and retention when combined with transarterial ischemic necrosis [120], but HCC is notorious for its low sensitivity to chemotherapy and tendency to develop multidrug resistance [121]. The current results have found moderate QoE according to the GRADE system that TAE is as good as any other chemo-embolization treatment contesting the widespread use of intra-arterial doxorubicin and other chemotherapeutic results.

Another interesting result was that the addition of locoregional ablation in the form of percutaneous ablation or external radiotherapy had a strong additive effect in improving objective response and prolonging patient survival. The combination of TACE with external radiotherapy achieved better response rates (SUCRA 85%) and improved patient survival (SUCRA 86%) that were both significantly better than plain TAE or TACE (low-to-moderate QoE, and IF 61–100%). The combination of TACE with some form of percutaneous ablation (microwave or RF or alcohol) was also significantly better than TAE or TACE and was found to be the best performing treatment ranking first in terms of both OR (SUCRA 99%) and survival (SUCRA 96%). The latter findings support the enhanced therapeutic outcomes in case of combined transarterial and locoregional ablative treatments [18]. Pathology studies have shown that palliative transarterial lipiodol-based treatments may achieve >90% necrosis in widely variable rates; 26–70% of the treated nodules; depending on technique, lesion size and arterial anatomy [122, 123]. Hence, it would be very sensible to combine (chemo)-embolizations with other ablative therapies in order to achieve higher rates of tumor necrosis and thereby prolong patient survival. Comparative safety analysis demonstrated that TARE with a beta-emitter was the safest treatment (SUCRA 77%), whereas combined TACE and liver ablation had the most favourable safety and effectiveness profile (SUCRA 59% and 99%, respectively).

Overall, the findings of the present network meta-analysis are very much in line with the results of several individual direct meta-analyses exploring individual (chemo)-embolization strategies. A recent overview of the major findings of meta-analyses on the management of hepatocellular carcinoma summarized the body of evidence from more than 20 direct meta-analytic reports on embolization therapies for inoperable liver cancer [124]. Seven meta-analyses compared the outcomes of TACE/TAE versus no active treatment or supportive care and overall survival outcomes favoured TACE/TAE [27, 33, 125]. Another 3 reports compared the outcomes of TACE versus TAE and concluded that there was no survival difference [27, 126, 127]. Furthermore, 3 reports looked into DEB-TACE versus TACE and found benefit only in terms of tumour response like in the present work [24, 128, 129]. Four meta-analyses reported outcomes of TACE combined with sorafenib versus TACE alone and again found no survival benefit with the addition of sorafenib [29, 130]. Last, there were 3 meta-analyses exploring the combination of TACE with plain external or conformal radiotherapy and also found that combination therapy produced superior survival outcomes [18, 124]. The present work corroborates all of the above in a single model and further raises the combination of TACE and percutaneous tumour ablation as the best treatment option in terms of both local tumour response and overall patient survival.

We consider the fitted survival model another particular strength of the present study as it may provide absolute expected median survival outcomes for each treatment and help clinicians optimize their decision-making process as well as guide the informed consent of the patients. A previous meta-analysis of the expected survival rates of untreated patients in the control arms of randomized studies of HCC has provided interesting insights into the natural history of this largely heterogeneous patient group. Projected median survival was 12 months in the case of intermediate stage (BCLC category B) cases, and around 6 months in the case of advanced stage (BCLC category C) patients [131]. A recently released systematic review and meta-analysis of more than 10,000 patients with unresectable HCC treated with lipiodol TACE has reported a weighted median survival rate of 19.4 months (95%CI: 16.2–22.6 months) [8]. The above numbers compare favourably with the results of our comparative survival model. In the present analysis, the weighted median survival was calculated to be 13.9 months (95%CI: 11.0–17.8 months) across the control arms of best supportive care and projected to be 18.1 months (95%CI: 15.6–21.6 months) in the TACE arms (anchor treatment). The ESMO-ESDO guidelines quote an expected median survival following TACE treatment of approximately 20 months in the case of BCLC intermediate stage and no more than 11 months in the case of advanced stage HCC. Hence, the authors consider the current evidence synthesis to reflect mostly a population of predominantly intermediate stage hepatocellular carcinoma in line with guideline-recommended use of most transarterial embolization therapies. In parallel with comparative effectiveness results, expected survival outcomes were similar between TAE (median 20.9 months) and different TACE approaches (median range, 18.1–23.1 months), numerically better with TARE (median 25.4 months) and significantly improved with the addition of external radiotherapy or ablation (median >30 months).

Arguably, unresectable HCC is characterized by significant heterogeneity in lesion size, unifocal or multinodular or diffuse patterns of disease, and variable degrees of underlying liver dysfunction [5, 8, 131]. Experts have long advised against TACE in Child-Pugh B patients, whereas TARE and external radiation have been proposed for the more liver dominant types of disease. Hence, one treatment type cannot fit all this heterogeneous category of patients [132]. The authors believe that combination treatments customized to individual patient profiles on the basis of the presented treatment rankings may deliver better clinical results and further improve survival of patients presenting with unresectable HCC and preserved liver function. Most interestingly, we have shown a clear synergy between transarterial embolization and locoregional ablation that needs to be explored further in larger scale studies in properly selected patients.

There are certain limitations to the present analysis. Network meta-analyses are inherently more prone to uncertainty and bias compared to classical meta-analysis. In addition, network meta-analyses are often exploratory to identify areas for more targeted scientific research and to help inform the design of future RCTs. However, sensitivity, consistency, and heterogeneity analyses support the validity of our results. Another limitation is that all 55 studies span 2 decades of medical practice and patient population reflects, as expected, the well-known clinical and anatomical heterogeneity of patients with unresectable HCC. Nonetheless, our survival model is in close agreement with real-life practice supporting the notion of generalizability of our findings. Finally, we have not accounted for differences in the race and geography as certain clusters of studies were most often performed in Asia (e.g. a combination of TACE and external irradiation) or the Western countries (e.g. TACE and DEB-TACE options).

In conclusion, TACE, DEB-TACE, TARE and adjuvant systemic agents neither improved tumour objective response nor conferred any patient survival benefit compared to bland particle embolization (TAE). Combinations of TACE with external radiation or liver ablation achieved the best tumour response and patient survival. Therefore, the current trends of chemoembolization practise are clearly open to question and international guidelines may need to be revised. However, quality of evidence remains low to moderate, and clearly more and larger studies are needed, especially in the fields of radioembolization, on the role of new embolic particulate agents and to further elucidate the synergy of combined transarterial and ablative liver treatments.

## Search strategy

1 “hepatocellular carcinoma”[MESH], 2 “hepatocellular carcinoma”[TW], 3 “liver cancer”[MESH], 4 “liver cancer”[TW]

5 “unresectable”[TW], 6 “inoperable”[TW], 7 “advanced”[TW]

8 “Clinical trial”[Mesh], 9 “Randomized Controlled Trial”[Mesh], 10 “Clinical trial”[TW], 11 “Randomized”[TW], 12 “Meta-analysis”[Mesh], 13 “Meta-analysis”[TW]

14 “embolization”[MESH], 15 “chemoembolization”[MESH], 16 “sorafenib”[MESH], 17 “embolization”[TW], 18 “chemoembolization”[TW], 19 “sorafenib”[TW], 20 “transcatheter” [TW], 21 “ablation”[TW], 22 “radiotherapy”[TW], 23 “radiation”[TW], 24 “radioembolization”[TW], 25 “selective internal radiation therapy”[TW], 26 “radiofrequency”[TW], 27 “alcohol”[TW], 28 “drug-eluting”[TW], 29 “anti-angiog*”[TW], “bevazicumab”[TW], 30 “TACE”[TW], 31 “TAE”[TW], 32 “DEB-TACE”, 33 “TAE”[TW], 34 “SIRT”[TW], 35 “TARE”[TW]

### Search string

(#1 OR #2 OR #3 OR #4) AND

(#5 OR #6 OR #7) AND

(#8 OR #9 OR #10 OR #11 OR #12 OR #13) AND

(OR #14 OR #15 OR #16 OR #17 OR #18 OR #19 OR #20 OR #21 OR #22 OR #23 OR #24 OR #25 OR #26 OR #27 OR #28 OR #29 OR #30 OR #31 OR #32 OR #33 OR #34 OR #35)

## Supporting information

S1 Appendix Supplementary material containing **Table A**. Included randomized controlled trials and baseline patient characteristics, **Table B**. Active and control treatment received in the randomized controlled trials, **Table C**. Inconsistency analysis, **Table D**. Heterogeneity and model fit, **Table E**. Random effects metaregression analyses, **League tables** with fixed and random effects models for all endpoints, and **Funnel plots** (comparison-adjusted) to assess publication bias.
(PDF)

S1 PRISMA Checklist PRISMA checklist. (DOC)

## Abbreviations

BCLC: Barcelona Clinic Liver Cancer staging system
CrI: Credible intervals
CTCAE: Common Terminology Criteria for Adverse Events
DEB-TACE: Drug-eluting bead transarterial chemoembolization
DIC: Deviance information criterion
EASL: European Association for the Study of the Liver
HCC: Hepatocellular carcinoma
HR: Hazard ratio
MW: Microwave ablation
NMA: Network meta-analysis
OR: Objective response
QoE: Quality of Evidence
RCT: Randomized controlled trial
RECIST: Response Evaluation Criteria In Solid Tumors
RF: Radiofrequency ablation
SAE: Serious adverse events
SIRT: Selective internal radiation therapy
SUCRA: Surface Area Under the Cumulative Rankograms
TACE: Transarterial chemoembolization
TAE: Transarterial embolization
TARE: Transarterial radioembolization
