# Supplementary material for: Comparative effectiveness of different transarterial embolization therapies alone or in combination with local ablative or adjuvant systemic treatments for unresectable hepatocellular carcinoma: A network meta-analysis of randomized controlled trials
Source: PLoS One. 2017 Sep 21;12(9):e0184597. doi: 10.1371/journal.pone.0184597 (PMC5608206; doi:10.1371/journal.pone.0184597)
Supplement: S1 Appendix — Supplementary material containing Table A. Included randomized controlled trials and baseline patient characteristics, Table B. Active and control treatment received in the randomized controlled trials, Table C. Inconsistency analysis, Table D. Heterogeneity and model fit, Table E. Random effects metaregression analyses, League tables with fixed and random effects models for all endpoints, and Funnel plots (comparison-adjusted) to assess publication bias. (PDF) [file pone.0184597.s001.pdf]

Table A. Included randomized controlled trials and baseline patient characteristics														
Clinical Trial	Year & country	Study arms	Patients (n)	Age (years)	Male gender	Viral hepatitis	Child-Pugh (A-B-C)	Okuda (I-II-III)	BCLC (0-A-B-C-D) or TNM (I-IV)	ECOG (0-1-2-3) or KPS	Tumour burden (ml, cm, %)	Multifocal or diffuse	Portal vein thrombus	Follow-up (time)
Groupe d'Etude	1995	TACE	50	63 (43–74)	48/50	6/50	50-0-0	NA	NA	NA	NA	25/50	1/50	4 years
	Europe	BST	46	65 (34–75)	44/46	7/46	46-0-0	NA	NA	NA	NA	32/46	6/46	
Madden et al.	1993	TACE	25	48 (24-70)	21/25	NA	NA	3-18-4	NA	1 (1-3)	NA	NA	NA	5 months
	South Africa	BST	25	49 (18-70)	25/25	NA	NA	4-16-5	NA	1 (1-3)	NA	NA	NA	
Pelletier et al.	1990	TACE	21	64 ± 8	19/21	NA	NA	6-11-4	NA	NA	34 ± 31%	NA	excluded	12 months
	France	BST	21	66 ± 11	18/21	NA	NA	5-11-5	NA	NA	41 ± 27%	NA	excluded	
Pelletier et al.	1998	TACE	37	67 (53–80)	31/37	NA	26-11-0	22-10-5	NA	22-14-1-0	27% (5–60)	NA	excluded	2 years
	France-Belgium	BST	36	65 (42–80)	31/36	NA	30-6-0	22-11-3	NA	20-14-2-0	20% (2–70)	NA	excluded	
Lo et al.	2002	TACE	40	62 (53-69)	34/40	34/40	NA	19-21-0	NA	20-16-3-1	7cm (4-14)	23/40	9/40	3.5 years
	Asian pts	BST	39	63 (53-70)	29/39	29/39	NA	18-21-0	NA	14-19-4-2	7cm (5-11)	24/39	12/39	
Llovet et al.	2002	TAE	37	64 (62–67)	30/37	32/37	27-10-0	24-13-0	0-0-28-9-0	28-7-2-0	5.2cm (4.6–6.0)	28/37	excluded	4 years
		TACE	40	63 (61–66)	32/40	37/40	31-9-0	27-13-0	0-0-35-5-0	35-4-1-0	4.9cm (4.0–5.8)	27/40	excluded	
	Spain	BST	35	66 (64–68)	23/35	33/35	21-14-0	22-13-0	0-0-27-8-0	27-4-4-0	4.4cm (3.9–4.9)	27/35	excluded	
FFCD	2008	TACE	62	64.9 ± 7.3	52/62	9/62	46-16-0	46-16-0	NA	18-32-4-1	NA	42/62	7/62	5 years
	France	Tamoxifen	61	63.9 ± 7	55/61	10/61	42-19-0	42-19-0	NA	27-26-2-0	NA	44/61	6/61	
Mabed et al.	2009	TACE	50	52 (36–60)	32/50	46/50	34-16-0	26-24-0	NA	1 (0-2)	NA	30/50	NA	1 year
	Egypt	I.V. DR	50	51 (34–60)	33/50	45/50	35-15-0	28-22-0	NA	1 (0-2)	NA	28/50	NA	
Lin et al.	1988	TAE	21	49.4 ± 10.3	19/21	16/21	21 (A+B)	NA	NA	NA	NA	NA	excluded	2 years
		TAE+IV 5FU	21	49.5 ± 9.2	18/21	17/21	21 (A+B)	NA	NA	NA	NA	NA	excluded	
	China	IV 5FU	21	49.8 + 10.1	21/21	17/21	21 (A+B)	NA	NA	NA	NA	NA	excluded	
Bruix et al.	1998	TAE	40	61 ± 9	30/40	31/40	NA	27-13-0	NA	27-11-2-0	NA	32/40	NA	4 years
	Spain	BST	40	64 ± 8	30/40	31/40	NA	27-13-0	NA	27-11-2-0	NA	29/40	NA	
Raoul et al.	1994	TARE	14	65.4 ± 6.5	26/27	2/14	8-6-0	3-11-0	NA	NA	NA	7/13	14/14	1 year
	France	BST	13	67.6 ± 6.7		2/13	6-7-0	5-8-0	NA	NA	NA	12/14	13/13	
Raoul et al.	1997	TARE	65	64.6 ± 7.0	62/65	NA	53-11-1	35-30-0	NA	KPS>70%	N=19 >50%liver	32/65	excluded	4 years
	France	TACE	64	65.7 ± 6.0	60/64	NA	44-19-1	37-27-0	NA		N=12 >50%liver	33/64	excluded	
Kolligs et al.	2015	TARE	13	65.8 ± 6.73	11/13	NA	9-3-1	NA	0-5-5-3-0	10-3-0-0	137.7ml	67.9% (BCLC>A)	excluded	2 years
	Germany-Spain	TACE	15	66.7 ± 9.04	13/15	NA	9-4-2	NA	0-4-8-3-0	12-3-0-0	235.6ml		excluded	
Salem et al.	2016	TARE	24	62 (58-65)	17/24	16/24	12-12-0	NA	0-18-6-0-0	NA	3.2 (2.7-3.7)	11/24	excluded	2 years
	United States	TACE	21	64 (62-70)	16/21	15/21	15-8-0	NA	0-17-4-0-0	NA	3.0 (2.3-3.6)	10/21	excluded	

Lammer et al.	2009	DEB-TACE	93	67.3 ± 9.1	79/93	38/93	77-16-0	79-14-0	0-24-69-0-0	74-19-0-0	16.1% (<10–50)	35/93	excluded	6 months
	Europe	TACE	108	67.4 ± 8.8	95/108	36/108	89-19-0	103-5-0	0-29-79-0-0	80-28-0-0	16.1% (<10–50)	50/108	excluded	
Sacco et al.	2011	DEB-TACE	33	71.3 ± 7.2	23/33	26/33	29-4-0	NA	0-22-11-0-0	NA	4.47 ± 2.68cm	NA	11/33	3.5 years
	Italy	TACE	34	68.7 ± 8.1	22/34	29/34	25-9-0	NA	0-22-12-0-0	NA	3.85 ± 1.89cm	NA	12/34	
Malenstein et al.	2011	DEB-TACE	16	67.3 ± 9.8	14/16	8/16	14-2-0	NA	0-2-9-5-0	9-7-0-0	NA	11/16	3/16	1 month
	Belgium	TACE	14	56.6 ± 13.4	11/14	4/14	14-0-0	NA	0-1-10-3-0	10-2-2-0	NA	8/14	3/14	
Golfieri et al.	2014	DEB-TACE	89	68.9 ± 8.0	66/89	68/89	75-14-0	NA	0-41-26-22-0	64-25-0-0	3.1 ± 1.6	44/89	excluded	2 years
	Italy	TACE	88	68.3 ± 8.0	69/88	62/88	77-11-0	NA	0-41-23-24-0	67-21-0-0	3.4 ± 1.9	49/88	excluded	
Chang et al.	1994	TACE	22	64 (43-78)	20/22	NA	13-9-0	NA	NA	NA	NA	13/22	excluded	2 years
	China	TAE	24	64 (45-78)	23/24	NA	17-7-0	NA	NA	NA	NA	13/24	excluded	
Kawai et al.	1992	TACE	147	61 (39-83)	125/147	NA	107-33-7	NA	NA	71-38-10-3-0	33cm^2	NA	excluded	3 years
	Japan	TAE	139	62 (41-83)	118/139	NA	102-25-3	NA	NA	77-36-3-1-1	28cm^2	NA	excluded	
Meyer et al.	2013	TACE	44	63 (44-79)	39/44	24/44	38-6-0	22-10-0	0-11-18-12-0	31-8-5-0-0	NA	29/44	excluded	3 years
	UK	TAE	42	62 (31-85)	35/42	25/42	33-9-0	25-8-0	0-9-16-15-0	27-9-6-0-0	NA	29/42	excluded	
Yu et al.	2014	TACE	45	65 (26–86)	37/45	39/45	37-8-0	NA	0-12-33-0	31-12-2-0	NA	23/45	excluded	4 years
	China	TAE	45	65 (26–86)	35/45	42/45	36-9-0	NA	0-5-39-1-0	28-16-0-1	NA	24/45	excluded	
Malagari et al.	2009	DEB-TACE	41	70.7 ± 6.9	31/41	NA	23-18-0	NA	NA	26-15-0-0	NA	18/41	excluded	1 year
	Greece	TAE	43	70 ± 7.9	34/43	NA	26-17-0	NA	NA	28-15-0-0	NA	14/43	excluded	
Brown et al.	2016	DEB-TACE	50	65.5 ± 11.8	41/50	22/50	45-5-0	43-7-0	0-12-23-15-0	43-7-0-0	4.3 ± 3.1cm	38/50	31/50	6 years
	USA	TAE	51	68.3 ± 9.7	37/51	23/50	41-10-0	39-12-0	0-10-22-19-0	44-7-0-0	4.7 ± 3.7cm	39/51	29/51	
Pitton et al.	2015	TARE	12	71.8 ± 7.2	8/12	5/12	10-2-0	NA	0-0-12-0-0	12-0-0-0	6.1 ± 3.6cm	12/12	excluded	3 years
	Germany	DEB-TACE	12	70.5 ± 9.0	10/12	5/12	9-3-0	NA	0-1-11-0-0	12-0-0-0	6.1 ± 3.8cm	11/12	excluded	
Sansonno et al.	2012	TACE + Adj	31	73 ± 4	18/31	31/31	31-0-0	NA	NA	25-6-0-0	7.36 ± 2.22cm	15/31	excluded	21 months
	Italy	TACE	31	72.8 ± 6.4	19/31	31/31	31-0-0	NA	NA	24-7-0-0	6.94 ± 3.34cm	13/31	excluded	
Kudo et al.	2011	TACE + Adj	229	69	174/229	186/229	229-0-0	NA	NA	201-28-0-0	NA	NA	122/458	3 years
	Japan-S. Korea	TACE	229	70	168/229	191/229	229-0-0	NA	NA	202-27-0-0	NA	NA		
Britten et al.	2012	TACE + Adj	15	61 (50-79)	13/15	11/15	13-2-0	NA	0-1-10-4-0	11-4-0-0	6.5 ± 2.0cm	4/15	excluded	5 years
	USA	TACE	15	58 (49-75)	12/15	11/15	15-0-0	NA	0-3-10-2-0	13-2-0-0	7.4 ± 2.9cm	4/15	excluded	
Pinter et al.	2015	TACE + Adj	16	61.1 ± 8.0	16/16	9/16	11-5-0	NA	0-2-14-0-0	16-0-0-0	NA	9/16	NA	46 months
	Austria	TACE	16	61.3 ± 8.7	13/16	5/16	11-5-0	NA	0-2-14-0-0	16-0-0-0	NA	10/16	NA	
Wang et al.	2015	TACE + Adj	61	55 (33-70)	51/61	61/61	52-9-0	NA	0-0-51-10-0	NA	NA	15/61	10/61	40 months
	China	TACE	64	55 (31-70)	55/64	64/64	54-10-0	NA	0-2-50-12-0	NA	NA	26/64	12/64	
Li et al.	2009	TACE + Adj	108	48 (20–73)	77/108	77/108	98-10-0	70-38-0	NA	NA	4.9 ± 1.3cm	59/108	excluded	3 years
	China-Singapore	TACE	108		74/108	86/108	99-9-0	70-38-0	NA	NA	4.8 ± 1.2cm	59/108	excluded	

Kudo et al.	2014	TACE + Adj	249	57 (21-85)	206/249	207/249	239-9-1	NA	0-65-129-54-1	201-48-0-0	NA	158/249	NA	3 years
	Multinational	TACE	253	59 (25-85)	216/253	210/253	231-20-2	NA	0-57-150-44-2	203-50-0-0	NA	170/253	NA	
Inaba et al.	2013	TACE + Adj	50	NA	39/50	42/51	40-9-0	NA	3-18-24-5-0	45-5-0-0	NA	30/50	NA	3 years
	Japan	TACE	51	NA	43/51	40/51	45-6-0	NA	9-13-27-2-0	49-2-0-0	NA	28/51	NA	
Lencioni et al.	2016	DEB-TACE + Adj	154	64.5	135/154	102/154	154-0-0	NA	0-0-154-0-0	154-0-0-0	NA	154/154	excluded	800 days
	Multinational	DEB-TACE	153	63.0	126/153	95/153	153-0-0	NA	0-0-153-0-0	153-0-0-0	NA	153/153	excluded	
Yang et al.	2008	TACE + RFA	24	59.1±11.4	18/24	NA	11-5-1	NA	NA	NA	6.6±0.6	19/24	NA	2 years
	China	TACE	11	57.6±11.8	8/11	NA	10-5-0	NA	NA	NA	6.4±1.0	4/11	NA	
Bartolozzi et al.	1995	TACE + PEI	26	65.3 ± 6.2	19/26	23/26	14-12-0	NA	NA	NA	4.84 ± 1.44cm	8/26	excluded	3 years
	Italy	TACE	27	66.1 ± 4.9	22/27	25/27	11-16-0	NA	NA	NA	5.09 ± 1.36cm	13/27	excluded	
Becker et al.	2005	TACE + PEI	27	64 (47-76)	20/27	7/27	17-10-0	17-9-1	NA	NA	NA	14/27	10/27	30 months
	Germany	TACE	25	63.6(48-79)	21/25	7/25	22-3-0	19-6-0	NA	NA	NA	16/25	9/25	
Wu et al.	1998	TACE + PEI	50	55±18	47/50	NA	40-8-2	NA	NA	NA	5.2±2.3cm	NA	NA	3 years
	China	TACE	52	55±16	49/52	NA	40-9-3	NA	NA	NA	5.2±2.1cm	NA	NA	
Xu et al.	2002	TACE + PEI	23	NA	NA	NA	23-0-0	NA	NA	NA	>5cm	0/23	NA	3 years
	China	TACE	22	NA	NA	NA	22-0-0	NA	NA	NA	>5cm	0/22	NA	
Yamamoto et al.	1997	TACE + PEI	50	NA	42/50	NA	17-23-10	NA	JIS Stage II-IV	NA	>2cm	28/50	included	3 years
	Japan	TACE	50	NA	45/50	NA	20-19-11	NA		NA	>2cm	24/50	included	
Liu et al.	2009	TACE +RFA + PEI	39	53±13	NA	NA	35-4-0	NA	Advanced HCC	NA	7.0±1.9cm	NA	NA	2 years
	China	TACE	39	53±11	NA	NA	32-7-0	NA	Advanced HCC	NA	6.9±2.2cm	NA	NA	
Wang et al.	2007	TACE + RFA	43	58.2*	32/43	NA	34-9-0	NA	(Median TNM stage III)	NA	Median 3.0-3.5cm	NA	excluded	1 year
	China	TACE	40	58.5*	34/40	NA	32-8-0	NA		NA		NA	excluded	
Zhao et al.	2011	TACE + RFA	23	NA	NA	NA	NA	NA	Advanced HCC	NA	<5cm	<=3 lesions	23/23	3 years
	China	TACE	24	NA	NA	NA	NA	NA	Advanced HCC	NA	<5cm	<=3 lesions	24/24	
Huang et al.	2016	TACE + CRYO	60	N=29 <60y	44/60	NA	54-6-0	NA	Intermediate	NA	5.3±1.3cm	1/60	NA	5 years
	China	TACE	60	N=29 <60y	48/60	NA	53-7-0	NA	Intermediate	NA	4.9±1.2cm	0/60	NA	
Xue et al.	1995	TACE + RT	21	NA	NA	NA	A+B	NA	(AJCC TNM stage II)	NA	NA	NA	excluded	1 year
	China	TACE	20	NA	NA	NA	A+B	NA		NA	NA	NA	excluded	
Leng et al.	2000	TACE + RT	36	NA	NA	NA	36-0-0	NA	(0-7-25-4)	≥65	9.7cm	NA	NA	3 years
	China	TACE	39	NA	NA	NA	39-0-0	NA	(0-7-29-3)	≥65	10.4cm	NA	NA	
Wang et al.	2000	TACE + RT	20	35	18/20	5/20	16(A+B)	NA	(0-0-14-6)	NA	NA	5/20	NA	5 years
	China	TACE	20	38	19/20	5/20	18(A+B)	NA	(0-0-15-5)	NA	NA	7/20	NA	
Peng et al.	2000	TACE + RT	43	NA	NA	NA	NA	NA	(AJCC TNM stage II)	NA	n=19 >10cm	NA	11/43	5 years
	China	TACE	48	NA	NA	NA	NA	NA		NA	n=20 >10cm	NA	9/48	

Li et al.	2003	TACE + RT	41	50.3	NA	NA	27-14-0	NA	NA	NA	3.2-11.5cm	NA	excluded	3 years
	China	TACE	41	51.8	NA	NA	23-18-0	NA	NA	NA	3.6-9.0cm	NA	excluded	
Zhao et al.	2006	TACE + 3D-CRT	49	53	32	NA	49-0-0	NA	(36-13-0-0)	≥70	<6cm	NA	excluded	3 years
	China	TACE	47	52	28	NA	47-0-0	NA	(31-16-0-0)	≥70	<6cm	NA	excluded	
Shang et al.	2007	TACE + 3D-CRT	40	52	NA	32/40	40 (A+B)	NA	(28-12-0-0)	≥70	All <6cm	NA	excluded	3 years
	China	TACE	36	54	NA	30/36	36 (A+B)	NA	(22-14-0-0)	≥70	All <6cm	NA	excluded	
Xiao et al.	2008	TACE + 3D-CRT	30	NA	NA	NA	19-11-0	NA	(10-12-8-0)	≥70	2.8-14.5cm	NA	13/30	3 years
	China	TACE	30	NA	NA	NA	20-10-0	NA	(12-13-5-0)	≥70	2.5-16.0cm	NA	8/30	
Liao et al.	2010	TACE + 3D-CRT	24	NA	NA	NA	34-14-0	NA	(TNM III-IV)	NA	NA	NA	NA	3 years
	China	TACE	24	NA	NA	NA		NA		NA	NA	NA	NA	
Wang et al.	2006	TACE + RT	54	NA	NA	NA	(A+B)	NA	(0-8-39-7)	≥65	n=19 >5cm	5/54	NA	3 years
	China	TACE	54	NA	NA	NA	(A+B)	NA	(0-10-38-6)	≥65	n=22 >5cm	4/54	NA	
Zhang et al.	2012	TACE + γ knife-RT	135	53	NA	NA	(A+B)	NA	Advanced HCC	NA	NA	NA	35/135	2 years
	China	TACE	124	53	NA	NA	(A+B)	NA		NA	NA	NA	31/124	

Table B. Active and control treatment received in randomized controlled trials					
**Conventional transarterial chemoembolization (TACE) versus best symptomatic treatment (BST)**					
Clinical trial	TACE protocol	Anticancer drug	Control treatment	Drug	Comments
Groupe d'Etude	Every 2m months for a total of four courses	Cisplatin70 mg + Lipiodol10 ml + Gelfoam particles	Pain medications and treatment of complications	Acetaminophen or morphine, given in doses appropriatefor pain level	Amoxicillin–clavulanicacid (3 g per day) and metronidazole (1.5 g per day) were administered IV for 24h before procedure and continuedfor 8d either IV orp.o.
Madden et al.	One dose and the repeated 4w later if the patient still satisfied the entry criteria of the trial.	5-epidoxorubicin (60 mg/mi) emulsified in 6 ml Lipiodol and 5 ml meglumineiothalamate,	Symptomatic	NA	
Pelletier et al.	One dose and then treatment repeated at the 2nd, 6th and 12thmonths.	Doxorubicin (5O mg per course) and Gelfoam powder	Symptomatic	NA	
Pelletier et al.	Repeated every 3m during the 1sty and thereafter every 4m, unless contraindicated	Cisplatin (2 mg/kg) (+lipiodol+ lecithin + Gelatin sponge) +Tamoxifen (40mg) b.i.d.	Tamoxifen	Tamoxifen (40mg) b.i.d.	1.5 l/day of intravenous fluid from24h before to 48 h after treatment. Amoxicillin-clavulanic acid (3 g per day) was given for 5d IV or p.o.
Lo et al.	Repeated every 2 to 3m unless contraindicated	Cisplatin (1 mg/mL, max 30mg) + lipiodol + gelatin-sponge mixed with 40 mg of gentamicin.	Symptomatic	Treatment for symptoms and complications	IV fluids and Amoxicillin-clavulanic acid (1.2 g), mannitol (20 g), and tropisetron (5 mg) given before the procedure. After procedure, oral amoxicillin-clavulanic acid (375 mg 3 t/d) and sucralfate (500 mg 4t/d) for 3d
Llovet et al.	Baseline, 2mand 6m then every 6m. Treatment was discontinued if any exclusion criteria developed or at the patient's request. Progressive disease led to discontinuation of treatment if vascular invasion or extrahepatic spread developed.	Doxorubicin adjusted to bilirubin (<25·6 µmol/L, 75 mg/m2; 25·6–51·3 µmol/L, 50 mg/m2; 51·3–85·5 µmol/L, 25 mg/m2) + 10 mL lipiodol + Gelfoam fragments	Conservative	Liver decompensation was treated as in patients with non-neoplastic liver disease.	No antibiotic prophylaxis given.
FFCD	Every 2m until tumour stabilisation. After checking for absence of hepatic insufficiency, additional following a 2m period. Later repeated following 4m and then every 6m.	Epirubicin 50 mg + 15 mL lipiodol+ Gelfoam cubes + Tamoxifen daily dose of 20 mg	Tamoxifen	Tamoxifen daily dose of 20 mg	3–4 L/d fluids + furosemide + analgesics if needed. Ceftriaxone (2 g/d) IV 2–3d and then 7–8dp.o.
Mabed et al.	Single session	Cisplatin 50-mg + 40-mg Doxorubicin +lipiodol 10 mL mixed with 10-mgdoxorubicin	Doxorubicin. Cycles repeated as long as dose of 500 mg/m^{2}was notexceeded.	45 mg/m^{2}for 4w (15 mg/m^{2}IV on days 1,8 and 15)	
**Bland transarterial embolization (TAE) versus best symptomatic treatment (BST)**					
Clinical trial	TAE protocol		Control treatment	Drug	Comments
Lin et al.	Baseline and then every month until no new vessel formation was found or until technical failure, development of extrahepatic metastasis or other contraindications for TAE were encountered.	Ivalon particles and Gelfoam powder or cubes.	I.V. 5-FU unless leukopenia, thrombocytopenia, or other contraindications developed.	5-fluorouracil (1.0 g/m2 body surface day for 5 days)	Analgesics and antibiotics were not given routinely after TAE unless abdominal pain or fever suggestive of infection developed.

Bruix et al.	Single session	Small cubes (131 mm) of gelatin injected until achieving absence of flow. In patients with unilobar disease, the distal embolization with gelatin was combined, with the proximal placement of a steel coil.	Symptomatic	Pain was treated avoiding the use of nonsteroidal anti-inflammatory agents.	Analgesics (pentazocine or meperidine) were administered if necessary.
Llovet et al.	Baseline, 2 m and 6 m then every 6 m. Treatment was discontinued if any exclusion criteria developed or at the patient's request. Progressive disease led to discontinuation of treatment if vascular invasion or extrahepatic spread developed.	Gelfoam fragments until flow stagnation was achieved.	Conservative	Liver decompensation was treated as in patientswith non-neoplastic liver disease.	No antibiotic prophylaxis given.
**Transarterialradioembolization (TARE) versus best symptomatic treatment (BST)**					
Clinical trial	TARE protocol	Drug	Control Treatment	Drug	Comments
Raoul et al.	Baseline and then 2, 5, 8 and 12m; canceled or postponed in case of poor performance status or occurrence of extrahepatic metastasis	3ml of ^{131}Iiodized oil (60 mCi)	Conservative	Tamoxifen (20-40 mg/day), NSAIDs, corticosteroids or antalgic drugs at any time.	After the therapeutic injection, the patients were isolated for 7 days for radioprotection of other patients and visitors.
**Transarterialradioembolization (TARE) versus conventional transarterial chemoembolization (TACE)**					
Clinical trial	TARE protocol	Drug	TACE protocol	Drug	Comments
Raoul et al.	Baseline and then 2, 5, 8, 12, and 18 m (If patient's general health status remained satisfactory and no signs of metastasis)	3 ml of ^{131}I-labeled Lipiodol (60 mCi, 2.2 GBq).	Baseline and then 2, 5, 8, 12, and 18 m (If patient's general health status remained satisfactory and no signs of metastasis). If portal vein thrombosis no Gelatin sponge after TACE.	Cisplatinum 70 mg diluted in 140 ml of saline solution and 10 ml Lipiodol. Gelatin-sponge fragments were then injected.	Antibiotic prophylaxy using amoxicillin and clavulanic acid was given to all patients before and after injections.
Kolligs et al.	Single Session	Selective intraarterial implantation of 0.5–3 GBq 90Y-resin microspheres as a lobar, segmental treatments or whole-liver approach	Repeat TACE was conducted every 6 w until tumor enhancement was not observed on MRI or until tumor progression was confirmed	Epirubicin 50 mg/m^2, lipiodol (median 7.0 mL) and embolizing agent.	
Salem et al.	Planning angiography followed by treatment on an outpatient basis	Glass microspheres (TheraSphere; BTG International, West Conshohocken, PA) at a 120-Gy dose	Drug/lipiodol was followed by embolic microspheres (Embospheres; Merit Medical Systems, South Jordan, UT)	75 mg/ m2 (maximum, 150 mg) dosing	
**Drug-eluting beads chemoembolization (DEB-TACE) versus conventional transarterial chemoembolization (TACE)**					
Clinical trial	DEB-TACE protocol	Drug	TACE protocol	Drug	Comments
Lammer et al.	Maximum of 3 chemoembolizations (at baseline, 2 months, and 4 months)	4 ml DC Bead (1 vial of 300–500 lm first, followed by 1 vial of 500–700 lm) loaded with doxorubicin (150 mg per procedure) mixed with nonionic contrast medium. Lipiodol was not used	Maximum of three chemoembolizations (at baseline, 2 months, and 4 months)	doxorubicin (50–75 m to a maximum of 150 mg, adjusted for bilirubin concentration and body surface area) in lipiodol followed by particle embolization with an embolic agent	
Sacco et al.	1.1 cycles	2–4 mL of DC Bead (100–300µm particle size) loaded with Doxorubicin (50 mg per vial; range, 25–150 mg; mean, 55 mg).	1.4 cycles	Doxorubicin (50–75 mg; mean, 57.0 mg) and Lipiodol (10–25 mL; mean, 16.6 mL), followed by selective arterial embolization with gelatin sponge particles	Performed under local analgesia, with antibiotic prophylaxis (ceftriaxone 1 g on days 0, 1, and 2) and antiemetic medications
Malenstein et al.	N/A	1 vial of 25 mg dry microspheres with a nominal diameter of 50–100	N/A	2 syringes (5 ml NaCl 0.9%, 2.5 ml lipiodol and 1.25 mg doxorubicin),	No prophylactic antibiotics were used.

		µm was mixed with doxorubicin and dissolved in 10 ml NaCl 0.9% and 10 ml of contrast medium.		and 1 infusion unit containing the remaining amount of doxorubicin dissolved in NaCl 0.9%.	
Golfieri et al.	Repeated 'on demand' upon demonstration of a persistent viable tumour (i.e.the absence of complete response (CR)) or intra-hepatic distal recurrence at imaging follow-up, provided that liver function had not deteriorated	DC-Beads 100–300 mm (each vial was loaded with 50 mg of a doxorubicin solution).	Repeated 'on demand' upon demonstration of a persistent viable tumour (i.e.the absence of complete response (CR)) or intra-hepatic distal recurrence at imaging follow-up, provided that liver function had not deteriorated	Epirubicin manually emulsified with iodised oil in a proportion of 1 to 1 vial (50 mg of drug with 10 ml of Lipiodol) to a maximum administered dose of 75 mg, followed by embolisation with absorbable gelatin sponge particles	
**Bland transarterial embolization (TAE) versus conventional transarterial chemoembolization (TACE)**					
Clinical trial	TAE protocol		TACE protocol	Drug	Comments
Chang et al.	Every 2-3 months until there was no visible tumor, or the patient could not sustain further TAE, or the patient died.	Lipiodol + gelfoam particles	Every 2-3 months until there was no visible tumor, or the patient could not sustain further TAE, or the patient died.	Cisplatin (50 mg) + Lipiodol + Gelfoam particles	
Kawai et al.	After 4th week could receive additional treatment.	Lipiodol + Gelatin sponge	After 4th week could receive additional treatment.	Adriamycin 40 mg/m^2 + Lipiodol + Gelatin sponge	
Meyer et al.	Repeated up to 3 times at 3-week, and after that up to investigator's discretion.	Polyvinyl alcohol particles (PVA) 50–150 mm.	Repeated up to 3 times at 3-week intervals unless haematological toxicity or if they experienced any grade IV non haematological toxicity. TAE could continue after that and was up to investigator's discretion.	Cisplatin (50 mg in 50 ml) + 4-6h later Polyvinyl alcohol particles (PVA) 50–150 mm.	Prophylactic antibiotics were administered to reduce the risk of infection
Yu et al.	Two treatment sessions conducted 2 months apart	Ethiodized oil–ethanol solution	Two treatment sessions conducted 2 months apart	Cisplatin–ethiodized oil emulsion (0.5 mg cisplatin/ml), followed by 1mm of gelatin-sponge pellets/ml)	Prophylactic antibiotic was not given
**Drug-eluting beads chemoembolization (DEB-TACE) versus bland transarterial embolization (TAE)**					
Clinical trial	DEB-TACE protocol	Drug	TAE protocol		Comments
Malagari et al.	Every 2 months, to a max of 3	DC Beads loaded with Doxorubicin at 37.5 mg/ml of bead suspension (intention to administer 150 mg of Doxorubicin)	Every 2 months, to a max of 3	Bead Block	
Brown et al.	A median of two embolizations	Doxorubicin 150 mg onto 4 or 6 ml of LCB microspheres (37.5 or 50 mg/mL), depending on assessment of a combination of tumor volume and vascularity	A median of two embolizations	Bead Block	
**Drug-eluting beads chemoembolization (DEB-TACE) versus transarterialradioembolization (TARE)**					
Clinical trial	DEB-TACE protocol	Drug	TARE protocol	Drug	Comments
Pitton et al.	TACE was repeated every 6 w until no more viable tumour was detected by MRI. If contraindications appeared, crossover to SIRT was possible according to the protocol.	Doxorubicin 150 mg per session on drug-eluting beads (100-300 µm).	TARE could be repeated once according to the study protocol. In cases with contraindications, crossover to TACE was permitted.	Angiography of the hepatic artery and protective coiling of side branches + 150 MBq 99mTc-MAA (macroaggregated albumin) + Resin-based 90Y loaded microparticles performed in a lobar approach. In case of bilobar tumor spread, treatment was split in two sessions	Patients with crossover from TACE to SIRT were not censored.
**Conventional transarterial chemoembolization (TACE) plus adjuvant systemic therapy versus conventional transarterial chemoembolization (TACE)**					
Clinical trial	TACE protocol	Anticancer drug-Adjuvant therapy	TACE protocol	Anticancer drug	Comments
Sansonno et al.	Repeated at intervals of 4–6w until	TACE + Sorafenib (400mg/twice	Repeated at intervals of 4–6w until	TACE + Placebo	

	complete necrosis of tumor detected.<br>Sorafenib administration was stopped following evidence of tumor progression.	daily) 30 days after TACE	complete necrosis of tumor detected.	TACE: Doxorubicin (30 mg) and mitomycin C (10mg) with 10 mL of iodinated nonionic contrast media and 20mL Lipiodol	
Kudo et al.	Trial was divided into 28-day cycles.	TACE + Sorafenib (400mg/ twice daily)	Trial was divided into 28-day cycles	TACE + Placebo<br>TACE: Gelatin foam + lipiodol + Chemotherapeutic (epirubicin, cisplatin, doxorubicin, mitomycin)	Dose reductions (first 400 mg qd, then 400 mg qod) were allowed for drug-related toxicity
Britten et al.	Day 8 and after once more in 14th week.	TACE + Bevacizumab: 10 mg/kg IV on day 1, one week prior to the first TACE. Post-TACE 10 mg/kg every 2 weeks, as<br>long as serum transaminases had returned to pre-TACE levels, or within normal range	Day 8 and after once more in 14th week.	TACE: doxorubicin 25 mg/m^2 + lipiodol + cisplatin 50 mg/m^2 + mitomycin-C 5 mg/m^2 + Embosphere® microspheres. Stump occlusion of segmental or subsegmental feeding branches was performed with microfibrillar collagen	Cross-Over to Bevacizumab was allowed If progressive disease by 16th Week. No placebo.
Pinter et al.	TACE was repeated twice at 4-week intervals if it was technically feasible and if contrast enhancement of nodules was present at follow-up Imaging.	TACE + Bevacizumab: 5 mg/kg IV prior to the first TACE (same day) and every 14 days thereafter for 52 weeks.	TACE was repeated twice at 4-week intervals if it was technically feasible and if contrast enhancement of nodules was present at follow-up Imaging.	TACE + Placebo (saline infusion)<br>TACE: doxorubicin 75, 50, or 25 mg/m^2 adjusted for a serum bilirubin level of less than 1.5 mg/dL, 1.5–3.0 mg/dL, or greater than 3.0 mg/dL + lipiodol (1:1 ratio) in a total volume of 20 mL, + Bead Block	
Wang et al.	4 courses of As2O3 therapy (21d of treatment per course 2-week interval) + TACE twice within the courses of As2O3 therapy	TACE + Arsenic trioxide (As_2O_3): 10 mg/d IV (drip at least 4h).	TACE 2 times at the same interval period with other group.	TACE: Oxaliplatin (100 mg) + 30 to 50 mg of Epirubicin + 2 to 10 mL of Lipiodol +Gelatin sponge.	
Li M et al.	TACE: Baseline, at week 5 and at week 13.<br>IFN-a1b: One week after each TACE, stopping one week before the next. Stopped if recurrence occurred and if haematological disorders lasted >4weeks	TACE + IFN-a1b: 3mu, 3 times a week IM.	Baseline, at week 5 and at week 13.	TACE: Cisplatin (50 mg) + Lipiodol (10ml) + Gelatin sponge particles	Management of IFN-a1b toxicity Influenza-like syndromes: acetaminophen.<br>Leucocytopenia (< 2500 X 109/l) and thrombocytopenia (< 40 X 109/l): 20 mg Leucogen + 50 mg Batilol three times a day.
Kudo et al.	TACE: Once or twice<br>Brivanib: 28-day cyces	TACE (or DEB TACE) + Brivanib: 800mg once daily p.o. (b/w 2-21d after TACE)	Once or twice	TACE (or DEB TACE) + Placebo<br>TACE: single anticancer agent + lipiodol + embolization agent or DEB + single anticancer agent	Treatment interruptions and dose reductions (first 400 mg qd, then 400 mg qod) were allowed for drug-related toxicity
Inaba et al.	TACE: performed once<br>TSU-68: Discontinued when radiological progression observed or the occurrence of unacceptable adverse events.	TACE + TSU-68 200 mg twice daily (within 2w from TACE)	Performed once	TACE: epirubicin + lipiodol + gelatin sponge	No placebo given
**Drug-eluting beads chemoembolization (DEB-TACE) plus adjuvant systemic therapy versus Drug-eluting beads chemoembolization (DEB-TACE)**					
Clinical trial	DEB-TACE protocol–Anticancer Drug	Adjuvant systemic therapy	DEB-TACE protocol		Comments
Lencioni et al	Sorafenib: 4-week cycles<br>TACE treatments were performed on day 1 (±4 days) of cycles 3, 7, and 13 and every 6 cycles thereafter.	Sorafenib 400 mg twice daily continuously	TACE treatments were performed on day 1 (±4 days) of cycles 3, 7, and 13 and every 6 cycles thereafter.	DEB-TACE: Doxorubicin 150mg, 300-500 µm beads (3-7 days after placebo) + Placebo	Treatment interruptions and up to two dose reductions were permitted for drug-related adverse events
**Conventional transarterial chemoembolization (TACE) plus local tumour ablation versus conventional transarterial chemoembolization (TACE)**					
Clinical trial	TACE protocol – Anticancer drug	Tumour ablation technology	TACE protocol	Anticancer drug	Comments

Yang et al.	TACE: 1-3 treatments; if not improved RFA after 1 week	RFA Power: 100 W, Frequency 30/sec	3–7 times per patient.	Epirubicin (30–50mg) + Hydroxycamptothecin (15–20mg) + Lipiodol + Gelatin sponge (15–30ml)	
Bartolozzi et al.	One TACE + Ethanol Injection 3-4w later in 6-16 sessions (1-2/week)	Ethyl Alcohol 95% (sterile): 16-215ml	TACE: 2 sessions in 3-m interval	TACE: Doxorubicin (20-70mg) + lipiodol (5-20ml) + gelatin sponge	
Becker et al.	TACE: Every 2-6w and then Ethyl Alcohol 10d after each TACE session (6-12 injections per lesion 1-6 d apart) until: no viable tumor, contraindications, patient's death	Ethyl Alcohol 96% (sterile): 1-10ml	TACE: Every 2-6w	TACE: Mitomycin C (10 mg) + Lipiodol (10mL) + Gelatin-sponge particles	
Wu et al.	N/A	Intratumour Ethyl Alcohol/Lipiodol	N/A	N/A	
Xu et al.	N/A	Percutaneous ethanol injection	N/A	N/A	
Yamamoto et al.	N/A	Percutaneous ethanol injection	N/A	N/A	
Liu et al.	N/A	Ethyl Alcohol + RFA	N/A	N/A	
Wang et al.	One TACE then RFA every 2-3w	RFA: 460-KHz generator. Electrode consisted of 9 hook-shaped prongs and is able to ablate a 5.0 cm region. Tumors >3.5 cm were treated with multiple overlapping ablations	TACE every 4w	TACE: Epirubicin-adriamycin (E-ADM 60–80 mg)+ Cisplatin 80–100 mg + Mitomycin-C (8–10 mg) + Lipiodol + Gelatin sponge particles	
Zhao et al.	N/A	RFA	N/A	N/A	
Huang et al.	Argon-helium cryoablation combined with TACE. Repeat monthly if patient survival.	Platinum 25-50mg + Adriamycin 10-40mg + iodine oil 10-20ml	Conventional TACE. Repeat monthly if patient survival.	Platinum 25-50mg + Adriamycin 10-40mg + iodine oil 10-20ml	CT-guided cryoablation for 10-15min (down to -140°C) after TACE
**Conventional transarterial chemoembolization (TACE) plus external radiotherapy versus conventional transarterial chemoembolization (TACE)**					
Clinical trial	TACE protocol – Anticancer drug	Radiotherapy protocol	TACE protocol	Drug	Comments
Xue et al.	Radiotherapy after TACE once daily over 5–6w	Moving Strip: Fraction 1.5-2Gy	N/A	TACE: ADM (20mg) + 5-Fu (1.0g) + Lipiodol	
Leng et al.	N/A	Radiotherapy	N/A	N/A	
Wang et al.	TACE: Every 4, 6, and 8 weeks. Radiotherapy: 1/d 5/w (2w after TACE).	Whole-liver irradiation with the moving strip technique (150–180cGy), when the tumor dose at the center section reached 20–25Gy, the residual foci, as localized by ultrasonography, were treated with local small-field irradiation, 150–180 cGy to a dosage of 20–25Gy, boosting the total tumor dose to 50Gy	Every 4, 6, and 8 weeks.	TACE: Cisplatinum (60mg) + Adriamycin (40mg) + Mitomycin, (10mg) or Floxuridine (1000mg) + Lipiodol (2–10ml of 40%) + pledgets of gelatin sponge.	A third group receiving only Radiotherapy was also recruited
Peng et al.	TACE: PDD (20−40mg) + Adriamycin (40-80mg) + Mitomycin (10-20mg) + 5- FU (1000-1500mg) + Lipiodol (40% ≤20mL) + Gelatin sponge	120Gy per fraction, two fractions per day with 6 hours. Total dose 4-5Gy in 3-4 weeks		TACE: PDD (20−40mg) + Adriamycin (40-80mg) + Mitomycin (10-20mg) + 5- FU (1000-1500mg) + Lipiodol (40% ≤20mL) + Gelatin sponge	
Li et al.	2 TACE cycles with interval of 1 month and then 3D-CRT was started at an interval of 10 to 14d after the 2nd cycle of TACE	Tumor dose: 45Gy delivered in daily fractions of 1.8 Gy, and then another 5.4 Gy were boosted with A shrinkage technique based on CT scan in 1.8 Gy per fraction. The total dose was 50.4 Gy in 28 fractions	2 TACE cycles with interval of 1 month	TACE: Mitomycin-C (6 mg/m^2) + 5-FU (1000 mg/m^2), and cisplatin (40 mg/m^2) + Lipiodol (8–20 mL) + Doxorubicin (30 mg/m^2) + Gelatin sponge particle.	Whole liver irradiation was always avoided

Zhao et al.	3D-CRT after TACE every two days over 2–3w	3D-CRT: Fraction 4–5Gy	N/A	TACE: 5-Fu (0.75 g) + DDP (40mg) + HCPT (15 mg) + Lipiodol (20 ml)	
Shang et al.	3D-CRT after TACE once daily over 5–6w	3D-CRT: Fraction 2.0Gy	N/A	TACE: 5-Fu (1.0 g) + DDP (40–60mg) + EPI-ADM (60mg) + MMC (10–20mg) + Lipiodol (5–20 ml) + Gelatin sponge	
Xiao et al.	3D-CRT 7-21d after TACE	3D-CRT: Fraction 5Gy	N/A	TACE: DDP (100mg) + 5-Fu (1.0mg) + EPI-ADM (50-100mg) + LP (10-30mL) + Gelatin sponge 1-2mm	
Liao et al.	N/A	3D-CRT	N/A	N/A	
Wang et al.	Moving Strip: twice daily over 4–6w	Moving Strip: Fraction 1.15-1.4Gy	N/A	TACE: 5-Fu (1.0g) + DDP (60 mg) + ADM (50 mg)	
Zhang et al.	3D-CRT 28-36d after TACE	3D-CRT: Fraction 4-5Gy	N/A	TACE: Oxaliplatin (100mg/m^2) + Epirubicin (30mg/m^2) + Lipiodol (10-20mL)	

Table C. Inconsistency analysis of treatment effects (random effects models - 95% CrI)		
**Comparison**	**Consistency model**	**Unrelated mean effects**
SERIOUS ADVERSE EVENTS		
Control versus TACE	0.068 (0.015-0.214)	0.073 (0.015-0.247)
TARE versus TACE	0.431 (0.107-1.879)	0.399 (0.089-1.869)
TACE + ablation versus TACE	0.790 (0.136-4.706)	0.800 (0.128-5.0.38)
TAE versus DEB-TACE	0.932 (0.282-3.206)	0.805 (0.141-4.486)
TAE versus TACE	1.040 (0.368-3.095)	0.992 (0.268-3.751)
DEB-TACE versus TACE	1.115 (0.373-3.386)	1.012 (0.260-3.975)
DEB-TACE+adjuvant versus DEB-TACE	2.207 (0.246-20.08)	2.202 (0.220-22.24)
TACE+RT versus TACE	3.598 (0.328-41.60)	3.550 (0.293-45.65)
TACE + adjuvant versus TACE	4.674 (1.836-12.02)	4.665 (1.766-12.50)
OBJECTIVE RESPONSE		
Control versus TACE	0.072 (0.032-0.144)	0.113 (0.049-0.232)
TAE versus DEB-TACE	0.932 (0.530-1.630)	0.703 (0.319-1.531)
TAE versus TACE	1.165 (0.776-1.824)	1.112 (0.694-1.789)
DEB-TACE versus TACE	1.249 (0.761-2.155)	1.073 (0.588-1.946)
TACE + adjuvant versus TACE	1.339 (0.865-2.248)	1.331 (0.886-2.176)
DEB-TACE+adjuvant versus DEB-TACE	1.422 (0.618-3.309)	1.430 (0.646-3.149)
TARE versus TACE	1.926 (0.769-4.969)	1.892 (0.774-4.740)
TACE + RT versus TACE	3.775 (2.551-5.579)	3.770 (2.575-5.518)
TACE + ablation versus TACE	10.19 (5.524-19.27)	10.20 (5.595-19.01)
PATIENT SURVIVAL		
TACE versus Control	0.764 (0.641-0.911)	0.786 (0.646-0.955)
TAE versus Control	0.666 (0.522-0.845)	0.740 (0.488-1.117)
TARE versus Control	0.571 (0.401-0.814)	0.320 (0.169-0.611)
TAE versus TACE	0.870 (0.711-1.065)	0.813 (0.636-1.041)
TARE versus TACE	0.748 (0.537-1.045)	0.909 (0.575-1.440)
DEB-TACE versus TACE	0.881 (0.645-1.200)	0.991 (0.639-1.536)
TACE + adjuvant versus TACE	0.905 (0.803-1.031)	0.905 (0.805-1.036)
TACE + ablation versus TACE	0.545 (0.458-0.649)	0.549 (0.452-0.664)
TACE + RT versus TACE	0.603 (0.529-0.687)	0.603 (0.530-0.686)
DEB-TACE versus TAE	1.012 (0.738-1.388)	0.920 (0.593-1.423)
DEB-TACE versus TARE	1.178 (0.765-1.823)	1.054 (0.365-3.028)
DEB-TACE+adjuvant versus DEB-TACE	0.897 (0.588-1.369)	0.897 (0.591-1.367)

Table D. Heterogeneity and model fit (95% Credible Intervals)				
**Endpoint**	**Model**	**Heteroheneity I^2 (95%CI)**	**Residual deviance**	**DIC statistic**
Serious Adverse Events	Fixed effects	(67 arms)	39.711	385.534
	Random effects	1.01 (0.61-1.64)	53.824	325.631
Objective Response	Fixed effects	(79 arms)	47.194	434.144
	Random effects	0.29 (0.03 - 0.63)	55.535	432.936
Patient Survival	Fixed effects	(105 arms)	9.959	20.658
	Random effects	0.06 (0.001 – 0.17)	14.081	23.252

Table E. Meta-regression analysis with a random effects models (95%CrI)		
**Endpoint**	**Covariate**	**Regression coefficient**
**Serious adverse events**	Publication year	-0.050 ((-0.278) – 0.134
	Patient age	0.103 ((-0.125) – 0.338
	Male gender	-4.121 ((-16.74) – 8.043
	Child-Pugh A stage	-4.006 ((-13.15) – 3.239
	Multinodular HCC	27.35 (9.329 – 49.66)
	Follow-up period	-0.311 ((-1.295) – 0.507
**Objective response**	Publication year	-0.119 ((-0.268) – 0.010)
	Patient age	0.071 ((-0.057) – 0.195)
	Male gender	0.387 ((-7.583) – 8.740)
	Child-Pugh A stage	-2.883 ((-7.111) – 0.946)
	Multinodular HCC	61.13 (17.76 – 128.4)
	Follow-up period	0.516 ((-0.076) – 1.161
**Patient survival**	Publication year	0.004 ((-0.020) – 0.030)
	Patient age	0.012 ((-0.019) – 0.043)
	Male gender	-0.506 ((-2.643) – 1.584)
	Child-Pugh A stage	-0.002 ((-0.009) – 0.005)
	Multinodular HCC	2.914 ((-0.565) – 6.306)
	Follow-up period	0.049 ((-0.060) – 0.158)

SERIOUS ADVERSE EVENTS – Fixed Effects – LEAGUE TABLE

**TACE+adjuv**								
1.02 (0.29 – 3.13)	**TACE+RT**							
1.53 (0.73 – 3.20)	1.50 (0.41 – 6.24)	**DEB-TACE+adjuv**						
**3.38 (1.99 – 5.73)**	**3.31 (1.01 – 12.48)**	**2.20 (1.32 – 3.71)**	**DEB-TACE**					
**3.61 (2.81 – 4.67)**	**3.53 (1.19 – 12.21)**	**2.36 (1.18 – 4.74)**	1.07 (0.67 – 1.70)	**TACE**				
**3.79 (2.24 – 6.46)**	**3.72 (1.14 – 14.00)**	**2.48 (1.21 – 5.12)**	1.12 (0.68 – 1.86)	1.05 (0.66 – 1.68)	**TAE**			
**4.93 (1.77 – 14.02)**	**4.86 (1.10 – 23.82)**	3.22 (0.96 – 11.00)	1.46 (0.49 – 4.45)	1.36 (0.51 – 3.77)	1.30 (0.43 – 3.97)	**TACE+ablation**		
**13.77 (6.73 – 29.24)**	**13.58 (3.74 – 56.17)**	**9.00 (3.42 – 24.18)**	**4.08 (1.80 – 9.51)**	**3.81 (1.95 – 7.75)**	**3.63 (1.60 – 8.43)**	2.80 (0.83 – 9.49)	**TARE**	
**27.37 (13.87 – 57.70)**	**27.07 (7.57 – 111.20)**	**17.92 (7.05 – 47.51)**	**8.12 (3.75 – 18.65)**	**7.56 (4.05 – 15.29)**	**7.22 (3.37 – 16.37)**	**5.58 (1.69 – 18.68)**	1.99 (0.78 – 5.17)	**Control**

SERIOUS ADVERSE EVENTS – Random Effects – LEAGUE TABLE

**TACE+adjuv**								
1.30 (0.10 – 17.21)	**TACE+RT**							
1.90 (0.14 – 26.14)	1.47 (0.05 – 45.94)	**DEB-TACE+adjuv**						
4.20 (0.98 – 17.78)	3.24 (0.23 – 47.30)	2.21 (0.25 – 20.03)	**DEB-TACE**					
**4.52 (1.07 – 18.13)**	3.48 (0.24 – 49.07)	2.37 (0.19 – 28.58)	1.07 (0.31 – 3.55)	**TAE**				
**4.67 (1.84 – 12.02)**	3.60 (0.33 – 41.60)	2.46 (0.21 – 29.16)	1.12 (0.37 – 3.39)	1.04 (0.37 – 3.10)	**TACE**			
5.91 (0.79 – 43.33)	4.55 (0.23 – 92.34)	3.10 (0.15 – 63.65)	1.40 (0.17 – 11.25)	1.31 (0.17 – 10.40)	1.26 (0.21 – 7.35)	**TACE+ablation**		
**10.85 (1.90 – 57.64)**	8.38 (0.49 – 135.90)	5.71 (0.32 – 95.16)	2.59 (0.41 – 15.28)	2.41 (0.40 – 13.98)	2.32 (0.53 – 9.35)	1.84 (0.18 – 17.59)	**TARE**	
**68.51 (16.21 – 426.00)**	**53.10 (4.03 – 1016.00)**	**35.80 (2.76 – 726.30)**	**16.33 (3.58 – 110.68)**	**15.15 (3.60 – 100.15)**	**14.63 (4.67 – 67.70)**	**11.70 (1.50 – 128.70)**	**6.35 (1.11 – 55.59)**	**Control**

OBJECTIVE RESPONSE – Fixed Effects – LEAGUE TABLE

**TACE+ablation**								
**2.80 (1.54 – 5.25)**	**TACE+RT**							
**6.20 (2.80 – 13.92)**	**2.21 (1.12 – 4.32)**	**DEB-TACE+adjuv**						
**5.78 (2.14 – 15.46)**	2.06 (0.84 – 4.94)	0.93 (0.33 – 2.58)	**TARE**					
**8.48 (4.77 – 15.49)**	**3.03 (2.05 – 4.48)**	1.37 (0.72 – 2.62)	1.47 (0.63 – 3.54)	**TACE+adjuv**				
**8.81 (4.71 – 16.94)**	**3.15 (1.97 – 5.02)**	1.43 (0.88 – 2.32)	1.53 (0.62 – 3.82)	1.04 (0.68 – 1.60)	**DEB-TACE**			
**9.47 (5.15 – 17.89)**	**3.38 (2.18 – 5.26)**	1.53 (0.81 – 2.90)	1.64 (0.68 – 4.04)	1.12 (0.75 – 1.67)	1.07 (0.71 – 1.63)	**TAE**		
**10.63 (6.34 – 18.51)**	**3.80 (2.81 – 5.16)**	1.72 (0.95 – 3.14)	1.84 (0.81 – 4.29)	1.26 (0.98 – 1.60)	1.21 (0.85 – 1.72)	1.12 (0.82 – 1.55)	**TACE**	
**145.40 (62.64 – 366.60)**	**51.58 (25.07 – 117.00)**	**23.48 (9.71 – 60.58)**	**25.25 (8.72 – 77.64)**	**17.05 (8.48 – 37.86)**	**16.43 (7.89 – 37.44)**	**15.26 (7.77 – 33.26)**	**13.57 (7.08 – 29.05)**	**Control**

OBJECTIVE RESPONSE – Random Effects – LEAGUE TABLE

**TACE+ablation**								
**2.70 (1.30 – 5.70)**	**TACE+RT**							
**5.31 (1.70 – 16.29)**	1.96 (0.70 – 5.34)	**TARE**						
**5.75 (1.72 – 18.02)**	2.13 (0.71 – 5.94)	1.08 (0.28 – 4.15)	**DEB-TACE+adjuv**					
**7.57 (3.37 – 16.45)**	**2.82 (1.46 – 5.01)**	1.43 (0.50 – 4.02)	1.32 (0.44 – 3.91)	**TACE+adjuv**				
**8.16 (3.56 – 18.27)**	**3.02 (1.54 – 5.67)**	1.54 (0.53 – 4.46)	1.42 (0.62 – 3.31)	1.08 (0.54 – 2.19)	**DEB-TACE**			
**8.74 (4.05 – 18.68)**	**3.23 (1.78 – 5.66)**	1.65 (0.60 – 4.60)	1.52 (0.56 – 4.18)	1.15 (0.62 – 2.19)	1.07 (0.61 – 1.87)	**TAE**		
**10.19 (5.52 – 19.27)**	**3.78 (2.55 – 5.58)**	1.93 (0.77 – 4.97)	1.78 (0.68 – 4.92)	1.34 (0.87 – 2.25)	1.25 (0.76 – 2.16)	1.17 (0.78 – 1.82)	**TACE**	
**142.00 (55.92 – 395.40)**	**52.39 (23.60 – 128.90)**	**26.95 (8.44 – 93.81)**	**24.83 (7.78 – 88.97)**	**18.72 (8.25 – 49.32)**	**17.44 (7.59 – 45.52)**	**16.18 (7.78 – 38.77)**	**13.85 (6.91 – 31.32)**	**Control**

PATIENT SURVIVAL – Fixed Effects – LEAGUE TABLE

**TACE+ablation**								
0.90<br>(0.74 – 1.11)	**TACE+RT**							
0.73<br>(0.51 – 1.05)	0.81<br>(0.57 – 1.14)	**TARE**						
0.69<br>(0.41 – 1.16)	0.76<br>(0.46 – 1.28)	0.94<br>(0.53 – 1.67)	**DEB-TACE+adjuvant**					
**0.63<br>(0.49 – 0.81)**	**0.69<br>(0.55 – 0.87)**	0.86<br>(0.59 – 1.24)	0.91<br>(0.55 – 1.49)	**TAE**				
**0.62<br>(0.44 – 0.87)**	**0.69<br>(0.50 – 0.95)**	0.85<br>(0.56 – 1.29)	0.89<br>(0.61 – 1.33)	0.99<br>(0.73 – 1.34)	**DEB-TACE**			
**0.61<br>(0.51 – 0.72)**	**0.67<br>(0.58 – 0.77)**	0.83<br>(0.60 – 1.15)	0.88<br>(0.53 – 1.44)	0.97<br>(0.79 – 1.18)	0.97<br>(0.72 – 1.33)	**TACE+adjuvant**		
**0.55<br>(0.46 – 0.64)**	**0.60<br>(0.53 – 0.68)**	0.75<br>(0.54 – 1.03)	0.79<br>(0.48 – 1.29)	0.87<br>(0.72 – 1.05)	0.88<br>(0.65 – 1.19)	**0.90<br>(0.84 – 0.96)**	**TACE**	
**0.42<br>(0.33 – 0.53)**	**0.46<br>(0.38 – 0.57)**	**0.57<br>(0.40 – 0.80)**	0.60<br>(0.36 – 1.01)	**0.67<br>(0.53 – 0.84)**	**0.67<br>(0.48 – 0.94)**	**0.69<br>(0.58 – 0.83)**	**0.76<br>(0.65 – 0.90)**	**Control**

PATIENT SURVIVAL – Random Effects – LEAGUE TABLE

**TACE+ablation**								
0.90<br>(0.72 – 1.12)	**TACE+RT**							
0.73<br>(0.50 – 1.06)	0.81<br>(0.56 – 1.15)	**TARE**						
0.69<br>(0.40 – 1.19)	0.76<br>(0.45 – 1.30)	0.94<br>(0.52 – 1.72)	**DEB-TACE+adjuvant**					
**0.63<br>(0.48 – 0.82)**	**0.69<br>(0.55 – 0.88)**	0.86<br>(0.59 – 1.26)	0.91<br>(0.53 – 1.54)	**TAE**				
**0.62<br>(0.43 – 0.88)**	**0.68<br>(0.49 – 0.96)**	0.85<br>(0.55 – 1.31)	0.90<br>(0.59 – 1.37)	0.99<br>(0.72 – 1.36)	**DEB-TACE**			
**0.60<br>(0.49 – 0.74)**	**0.67<br>(0.56 – 0.79)**	0.83<br>(0.58 – 1.18)	0.87<br>(0.50 – 1.49)	0.96<br>(0.75 – 1.22)	0.97<br>(0.69 – 1.35)	**TACE+adjuvant**		
**0.54<br>(0.46 – 0.65)**	**0.60<br>(0.53 – 0.69)**	0.75<br>(0.54 – 1.05)	0.79<br>(0.47 – 1.33)	0.87<br>(0.71 – 1.07)	0.88<br>(0.65 – 1.20)	0.90<br>(0.80 – 1.03)	**TACE**	
**0.42<br>(0.32 – 0.53)**	**0.46<br>(0.37 – 0.57)**	**0.57<br>(0.40 – 0.81)**	0.60<br>(0.35 – 1.04)	**0.66<br>(0.52 – 0.85)**	**0.67<br>(0.48 – 0.95)**	**0.69<br>(0.56 – 0.86)**	**0.76<br>(0.64 – 0.91)**	**Control**

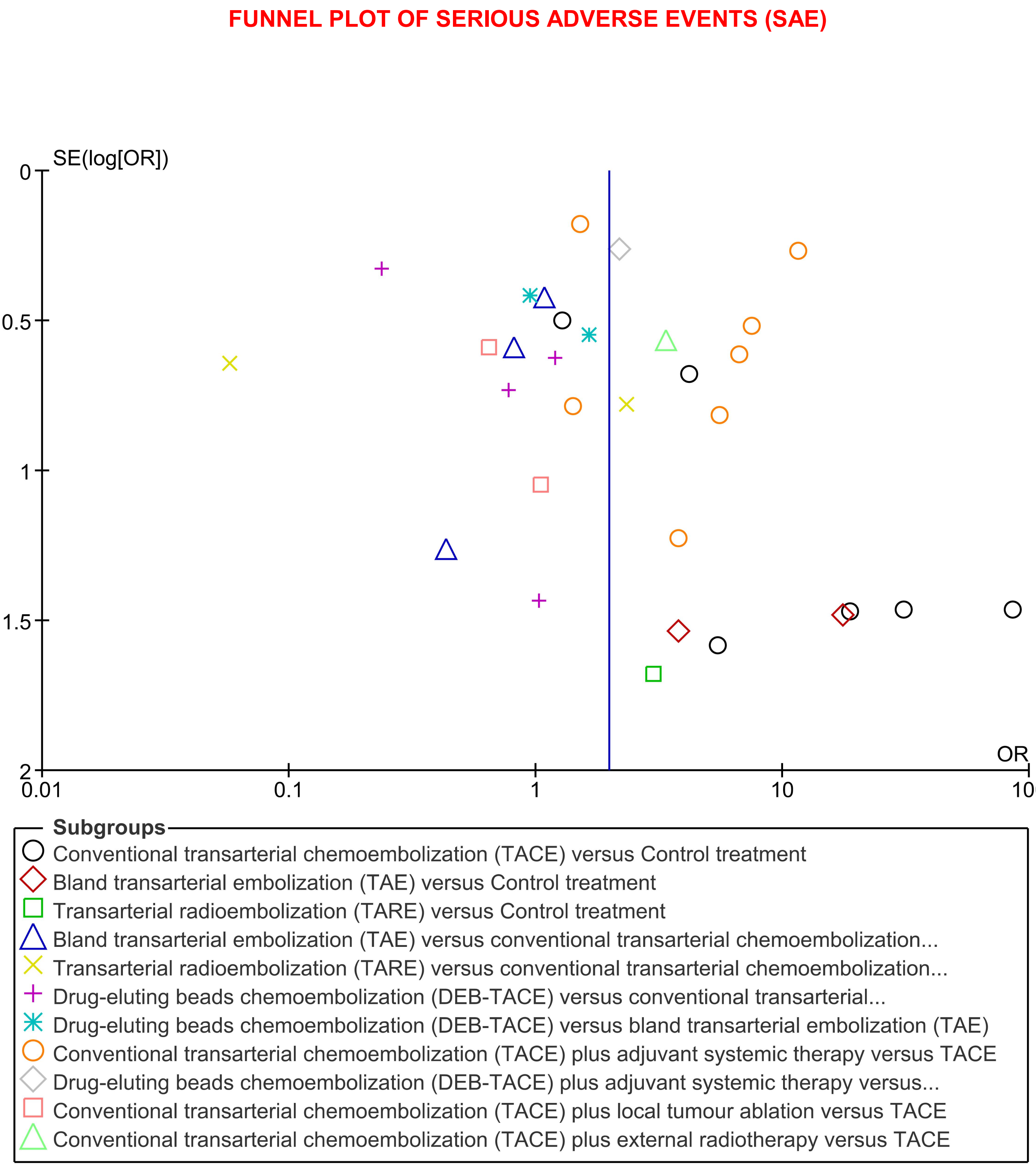
FUNNEL PLOT OF SERIOUS ADVERSE EVENTS (SAE)
SE(log[OR])
0
0.5
1
1.5
2
OR
0.01
0.1
1
10
100
Subgroups
Conventional transarterial chemoembolization (TACE) versus Control treatment
Bland transarterial embolization (TAE) versus Control treatment
Transarterial radioembolization (TARE) versus Control treatment
Bland transarterial embolization (TAE) versus conventional transarterial chemoembolization...
Transarterial radioembolization (TARE) versus conventional transarterial chemoembolization...
Drug-eluting beads chemoembolization (DEB-TACE) versus conventional transarterial...
Drug-eluting beads chemoembolization (DEB-TACE) versus bland transarterial embolization (TAE)
Conventional transarterial chemoembolization (TACE) plus adjuvant systemic therapy versus TACE
Drug-eluting beads chemoembolization (DEB-TACE) plus adjuvant systemic therapy versus...
Conventional transarterial chemoembolization (TACE) plus local tumour ablation versus TACE
Conventional transarterial chemoembolization (TACE) plus external radiotherapy versus TACE

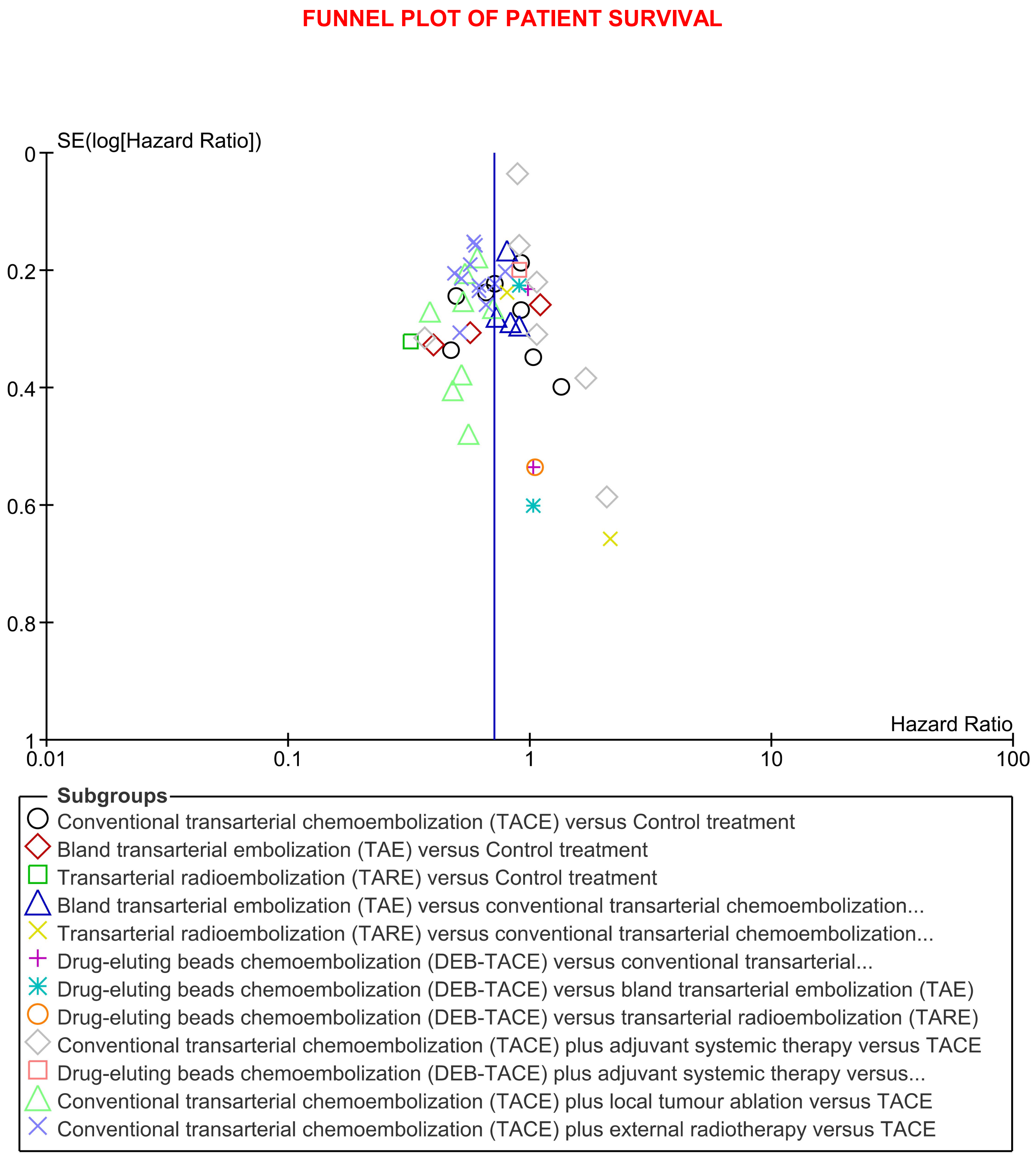
FUNNEL PLOT OF PATIENT SURVIVAL
SE(log[Hazard Ratio])
0
0.2
0.4
0.6
0.8
1
Hazard Ratio
0.01
0.1
1
10
100
Subgroups
Conventional transarterial chemoembolization (TACE) versus Control treatment
Bland transarterial embolization (TAE) versus Control treatment
Transarterial radioembolization (TARE) versus Control treatment
Bland transarterial embolization (TAE) versus conventional transarterial chemoembolization...
Transarterial radioembolization (TARE) versus conventional transarterial chemoembolization...
Drug-eluting beads chemoembolization (DEB-TACE) versus conventional transarterial...
Drug-eluting beads chemoembolization (DEB-TACE) versus bland transarterial embolization (TAE)
Drug-eluting beads chemoembolization (DEB-TACE) versus transarterial radioembolization (TARE)
Conventional transarterial chemoembolization (TACE) plus adjuvant systemic therapy versus TACE
Drug-eluting beads chemoembolization (DEB-TACE) plus adjuvant systemic therapy versus...
Conventional transarterial chemoembolization (TACE) plus local tumour ablation versus TACE
Conventional transarterial chemoembolization (TACE) plus external radiotherapy versus TACE

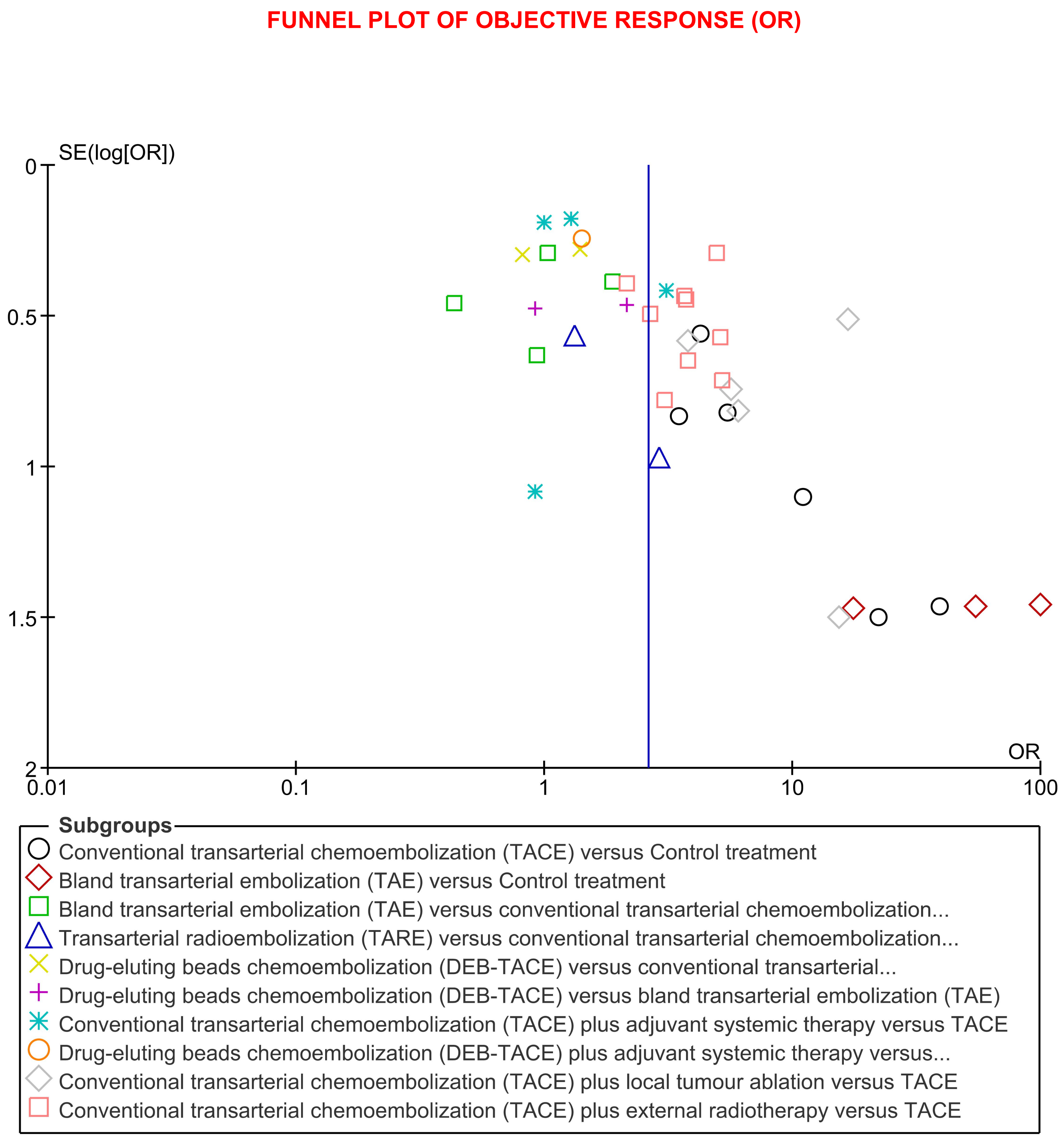
FUNNEL PLOT OF OBJECTIVE RESPONSE (OR)
SE(log[OR])
0
0.5
1
1.5
2
0.01
0.1
1
10
100
OR
Subgroups
Conventional transarterial chemoembolization (TACE) versus Control treatment
Bland transarterial embolization (TAE) versus Control treatment
Bland transarterial embolization (TAE) versus conventional transarterial chemoembolization...
Transarterial radioembolization (TARE) versus conventional transarterial chemoembolization...
Drug-eluting beads chemoembolization (DEB-TACE) versus conventional transarterial...
Drug-eluting beads chemoembolization (DEB-TACE) versus bland transarterial embolization (TAE)
Conventional transarterial chemoembolization (TACE) plus adjuvant systemic therapy versus TACE
Drug-eluting beads chemoembolization (DEB-TACE) plus adjuvant systemic therapy versus...
Conventional transarterial chemoembolization (TACE) plus local tumour ablation versus TACE
Conventional transarterial chemoembolization (TACE) plus external radiotherapy versus TACE

PLOS ONE

Comparative effectiveness of different transarterial embolization therapies alone or in combination with local ablative or adjuvant systemic treatments for unresectable hepatocellular carcinoma:A network meta-analysis of randomized controlled trials.

--Manuscript Draft--

Manuscript Number:	PONE-D-17-10178R1
Article Type:	Research Article
Full Title:	Comparative effectiveness of different transarterial embolization therapies alone or in combination with local ablative or adjuvant systemic treatments for unresectable hepatocellular carcinoma:A network meta-analysis of randomized controlled trials.
Short Title:	Embolization treatments for unresectable hepatocellular carcinoma: Systematic review and network meta-analysis
Corresponding Author:	Konstantinos Katsanos, M.Sc., M.D., Ph.D., E.B.I.R.<br>Guy's and St.Thomas' Hospitals, King's Health Partners<br>London, UNITED KINGDOM
Keywords:	systematic review<br>hepatocellular carcinoma<br>transcatheter<br>embolization<br>chemoembolization<br>radioembolization<br>ablation<br>radiotherapy<br>survival<br>network meta-analysis<br>response<br>unresectable<br>lipiodol
Abstract:	Background: The optimal transcatheter embolization strategy for patients with unresectable hepatocellular carcinoma (HCC) remains elusive. We conducted a systematic review and network meta-analysis (NMA) of different embolization options for unresectable HCC.<br><br>Methods: Medical databases were searched for randomized controlled trials evaluating bland transarterial embolization (TAE), conventional TACE, drug-eluting bead chemoembolization (DEB-TACE), or transarterial radioembolization (TARE), either alone or combined with adjuvant chemotherapy, or local liver ablation, or external radiotherapy for unresectable HCC up to June 2017. Random effects Bayesian models with a binomial and normal likelihood were fitted (WinBUGS). Primary endpoint was patient survival expressed as hazard ratios (HR) and 95% credible intervals. An exponential model was used to fit patient survival curves. Safety and objective response were calculated as odds ratios (OR) and accompanying 95% credible intervals. Competing treatments were ranked with the SUCRA statistic. Heterogeneity-adjusted effective sample sizes were calculated to evaluate information size for each comparison. Quality of evidence (QoE) was assessed with the GRADE system adapted for NMA reports. All analyses complied with the ISPOR-AMCP-NCP Task Force Report for good practice in NMA.<br><br>Findings: The network of evidence included 55 RCTs (12 direct comparisons) with 5,763 patients with preserved liver function and unresectable HCC (intermediate to advanced stage).All embolization strategies achieved a significant survival gain over control treatment (HR range, 0.42-0.76; very low-to-moderate QoE). However, TACE, DEB-TACE, TARE and adjuvant systemic agents did not confer any survival benefit over bland TAE alone (moderate QoE, except low in case of TARE).There was moderate QoE that TACE combined with external radiation or liver ablation achieved the best patient survival (SUCRA 86% and 96%, respectively).Estimated median survival was 13.9 months in control, 18.1 months in TACE, 20.6 months with DEB-TACE, 20.8 months with bland TAE, 30.1 months in TACE plus external radiotherapy,

Powered by Editorial Manager® and ProduXion Manager® from Aries Systems Corporation

	and 33.3 months in TACE plus liver ablation. TARE was the safest treatment (SUCRA 77%), however, all examined therapies were associated with a significantly higher risk of toxicity over control (OR range, 6.35 to 68.5). TACE, DEB-TACE, TARE and adjuvant systemic agents did not improve objective response over bland embolization alone (OR range, 0.85 to 1.65). There was clinical diversity among included randomized controlled trials, but statistical heterogeneity was low.<br><br>Conclusions: Chemo- and radio-embolization for unresectable hepatocellular carcinoma may improve tumour objective response and patient survival, but are not more effective than bland particle embolization. Chemoembolization combined with external radiotherapy or local liver ablation may significantly improve tumour response and patient survival rates over embolization monotherapies. Quality of evidence remains mostly low to moderate because of clinical diversity.<br><br>Systematic review registration: CRD42016035796 (http://www.crd.york.ac.uk/PROSPERO)
**Order of Authors:**	Konstantinos Katsanos, M.Sc., M.D., Ph.D., E.B.I.R.
	Panagiotis Kitrou
	Stavros Spiliopoulos
	Ioannis Maroulis
	Theodore Petsas
	Dimitris Karnabatidis
**Opposed Reviewers:**	
**Response to Reviewers:**	Please refer to attached letter outlining our point-by point response.
**Additional Information:**	
**Question**	**Response**
**Financial Disclosure**<br><br><br>Please describe all sources of funding that have supported your work. **This information is required for submission and will be published with your article, should it be accepted.** A complete funding statement should do the following:<br><br>Include **grant numbers and the URLs** of any funder's website. Use the full name, not acronyms, of funding institutions, and use initials to identify authors who received the funding.<br>**Describe the role** of any sponsors or funders in the study design, data collection and analysis, decision to publish, or preparation of the manuscript. If the funders had **no role** in any of the above, include this sentence at the end of your statement: "*The funders had no role in study design, data collection and analysis, decision to publish, or preparation of the manuscript.*"<br><br>However, if the study was **unfunded**, please provide a statement that clearly indicates this, for example: "*The author(s) received no specific funding for this work.*"	The author(s) received no specific funding for this work.

* typeset	
**Competing Interests**<br><br>You are responsible for recognizing and disclosing on behalf of all authors any competing interest that could be perceived to bias their work, acknowledging all financial support and any other relevant financial or non-financial competing interests.<br><br>Do any authors of this manuscript have competing interests (as described in the PLOS Policy on Declaration and Evaluation of Competing Interests)?<br><br>**If yes**, please provide details about any and all competing interests in the box below. Your response should begin with this statement: *I have read the journal's policy and the authors of this manuscript have the following competing interests:*<br><br>**If no** authors have any competing interests to declare, please enter this statement in the box: "*The authors have declared that no competing interests exist.*"<br><br>* typeset	The authors have declared that no competing interests exist.
**Ethics Statement**<br><br>You must provide an ethics statement if your study involved human participants, specimens or tissue samples, or vertebrate animals, embryos or tissues. All information entered here should **also be included in the Methods section** of your manuscript. Please write "N/A" if your study does not require an ethics statement.<br><br>**Human Subject Research (involved human participants and/or tissue)**<br><br>All research involving human participants must have been approved by the authors'	N/A

Institutional Review Board (IRB) or an equivalent committee, and all clinical investigation must have been conducted according to the principles expressed in the Declaration of Helsinki. Informed consent, written or oral, should also have been obtained from the participants. If no consent was given, the reason must be explained (e.g. the data were analyzed anonymously) and reported. The form of consent (written/oral), or reason for lack of consent, should be indicated in the Methods section of your manuscript.<br><br>Please enter the name of the IRB or Ethics Committee that approved this study in the space below. Include the approval number and/or a statement indicating approval of this research.<br><br>**Animal Research (involved vertebrate animals, embryos or tissues)**<br><br>All animal work must have been conducted according to relevant national and international guidelines. If your study involved non-human primates, you must provide details regarding animal welfare and steps taken to ameliorate suffering; this is in accordance with the recommendations of the Weatherall report, "The use of non-human primates in research." The relevant guidelines followed and the committee that approved the study should be identified in the ethics statement.<br><br>If anesthesia, euthanasia or any kind of animal sacrifice is part of the study, please include briefly in your statement which substances and/or methods were applied.<br><br>Please enter the name of your Institutional Animal Care and Use Committee (IACUC) or other relevant ethics board, and indicate whether they approved this research or granted a formal waiver of ethical approval. Also include an approval number if one was obtained.<br><br>**Field Permit**<br><br>Please indicate the name of the institution	

Powered by Editorial Manager® and ProduXion Manager® from Aries Systems Corporation

or the relevant body that granted permission.	
**Data Availability**<br><br>PLOS journals require authors to make all data underlying the findings described in their manuscript fully available, without restriction and from the time of publication, with only rare exceptions to address legal and ethical concerns (see the PLOS Data Policy and FAQ for further details). When submitting a manuscript, authors must provide a Data Availability Statement that describes where the data underlying their manuscript can be found.<br><br>Your answers to the following constitute your statement about data availability and will be included with the article in the event of publication. **Please note that simply stating 'data available on request from the author' is not acceptable.** ***If, however, your data are only available upon request from the author(s), you must answer "No" to the first question below, and explain your exceptional situation in the text box provided.***<br><br>Do the authors confirm that all data underlying the findings described in their manuscript are fully available without restriction?	Yes - all data are fully available without restriction
Please describe where your data may be found, writing in full sentences. **Your answers should be entered into the box below and will be published in the form you provide them, if your manuscript is accepted.** If you are copying our sample text below, please ensure you replace any instances of XXX with the appropriate details.<br><br>If your data are all contained within the paper and/or Supporting Information files, please state this in your answer below. For example, "All relevant data are within the paper and its Supporting Information files."<br>If your data are held or will be held in a public repository, include URLs, accession numbers or DOIs. For example, "All XXX files are available from the XXX database (accession number(s) XXX, XXX)." If this information will only be available after acceptance, please indicate this by ticking the box below.<br>If neither of these applies but you are able to provide details of access elsewhere, with or without limitations, please do so in	All raw data is contained in the tables and figures of the submitted manuscript.

the box below. For example:<br><br>“Data are available from the XXX Institutional Data Access / Ethics Committee for researchers who meet the criteria for access to confidential data.”<br><br>“Data are from the XXX study whose authors may be contacted at XXX.”<br><br>* typeset	
Additional data availability information:	Tick here if your circumstances are not covered by the questions above and you need the journal’s help to make your data available.

To: Editor in Chief

PLoS ONE Editorial Office **London, August 19th, 2017**

Dear Editor,

We would like to thank you and the expert referees once again for the time and effort spent and their interesting comments and constructive criticisms of our manuscript entitled: **"Comparative effectiveness of different transarterial embolization therapies alone or in combination with local ablative or adjuvant systemic treatments for unresectable hepatocellular carcinoma: A network meta-analysis of randomized controlled trials"** that was submitted for consideration for publication in the journal of **PLoS ONE**.

We have followed the comments of the referees and we hope that we have addressed their questions adequately. We apologise for the delay in submitting our revision as we had to **include another 2 RCTs (Salem et al. 2017 and Huang et al. 2017)** and hence, we had to re-run all analyses and revise all numerical results accordingly (minor decimal differences). Please find attached a **point-by-point** list of all the changes and revisions made. We also attach separately an annotated red-lined text file with numbered lines where you can refer for each revision made.

We believe that the present paper may be of particular interest and value for the average PLoS ONE reader as it shows that **(1) transcatheter arterial embolization therapies actually improve patient survival over control medical treatment** by reducing the hazard of death in the range of 24% (in case of chemoembolization) to 34% (in case of bland transarterial embolization) or 43% in case of radioembolization, **(2)** Transcatheter chemo- and radio-embolization monotherapies, or even combined with systemic chemotherapy, **are not more effective than plain bland particle transarterial embolization**, and **(3) Chemoembolization combined with external radiotherapy or local liver ablation may significantly prolong patient survival** over transarterial embolization monotherapies by **12-15 months extra median survival time**. Therefore, the current trends of chemoembolization for unresectable HCC are clearly open to question and international guidelines may need to be revised.

All authors have made significant contributions to the submitted work and have approved the final version of the manuscript.

In addition, the authors certify that:

(1) There has been no duplicate publication or submission of any part of the work elsewhere,

(2) None of the paper's contents have been previously published

(3) There is no financial arrangement or other relationship with the industry that could be construed as a conflict of interest.

Looking forward to hearing from you,

We thank you in advance,

Yours sincerely,

On behalf of the authors

Dr. K. Katsanos

Editor:

1. Please ensure that your manuscript meets PLOS ONE's style requirements, including those for file naming. The PLOS ONE style templates can be found online

Authors' response: We have followed the PLOS ONE's style requirements and have revised the whole manuscript and appended files according to the relevant style template available online.

2. Please present the full electronic search strategy for at least one database, including any limits used, such that it could be repeated.

Authors' response: We have inserted an example of our electronic search at the end of the manuscript as follows:

Lines 708-727:

Search strategy

1 "hepatocellular carcinoma"[MESH], 2 "hepatocellular carcinoma"[TW], 3 "liver cancer"[MESH], 4 "liver cancer"[TW]

5 "unresectable"[TW], 6 "inoperable"[TW], 7 "advanced"[TW]

8 "Clinical trial"[Mesh], 9 "Randomized Controlled Trial"[Mesh], 10 "Clinical trial"[TW], 11 "Randomized"[TW], 12 "Meta-analysis"[Mesh], 13 "Meta-analysis"[TW]

14 "embolization"[MESH], 15 "chemoembolization"[MESH], 16 "sorafenib"[MESH], 17 "embolization"[TW], 18 "chemoembolization"[TW],19 "sorafenib"[TW], 20 "transcatheter" [TW], 21 "ablation"[TW], 22 "radiotherapy"[TW], 23 "radiation"[TW], 24 "radioembolization"[TW], 25 "selective internal radiation therapy"[TW], 26 "radiofrequency"[TW], 27 "alcohol"[TW], 28 "drug-eluting"[TW], 29 "anti-angiog*"[TW], "bevazicumab"[TW], 30 "TACE"[TW], 31 "TAE"[TW], 32 "DEB-TACE", 33 "TAE"[TW], 34 "SIRT"[TW], 35 "TARE"[TW]

Search String

(#1 OR #2 OR #3 OR #4) AND

(#5 OR #6 OR #7) AND

(#8 OR #9 OR #10 OR #11 OR #12 OR #13) AND

(OR #14 OR #15 OR #16 OR #17 OR #18 OR #19 OR #20 OR #21 OR #22 OR #23 OR #24 OR #25 OR #26 OR #27 OR #28 OR #29 OR #30 OR #31 OR #32 OR #33 OR #34 OR #35)

3. Please state in the methods section who conducted the search, data extraction, and risk bias assessment.

Authors' response: We have provided the requested information as follows:

Line 150: KK, PK and SS performed the literature search and data extraction.

Line 169: A standardized data extraction form was used to collect the following information from all included trials (by KK, PK and SS):

Line 187: Risk of bias assessment was performed by KK, SS and DK.

4. Please assess the publication bias using statistical methods (in addition to funnel plots)

Authors' response: We have provided basic and comparison-adjusted funnel plots in the supporting supplemental material. In the case of network meta-analysis, comparison-adjusted funnel plots is the proposed method for evaluating potential publication bias; no formal statistical methods are currently available. *Please refer to Salanti G, Del Giovane C, Chaimani A, Caldwell DM, Higgins JP. Evaluating the quality of evidence from a network meta-analysis. PLoS One. 2014 Jul 3;9(7):e99682. doi: 10.1371/journal.pone.0099682.*

5. Please include captions for your Supporting Information files at the end of your manuscript beneath the references, and update any in-text citations to match accordingly. Please see our Supporting Information guidelines for more information: http://www.plosone.org/static/supportingInformation.

Authors' response: We have updated all relevant in-text citations and we have included a caption describing the supplementary supporting Information material (S1 Appendix) that reads as follows:

Lines 729-735: S1 Supplementary material and supporting information.
Supplemental material containing **Table 1.** Included randomized controlled trials and baseline patient characteristics, **Table 2.** Active and control treatment received in the randomized controlled trials, **Table 3.** Inconsistency analysis, **Table 4.** Heterogeneity and model fit, **Table 5.** Random effects metaregressions analyses, **League tables** with fixed and random effects models for all endpoints, and **Funnel plots** (adjusted) to assess publication bias.

Reviewer #1:

6. Obviously, the authors spent lots of time on the work. The issue discussed in this work is relatively broad. Several major revision comments should be addressed. The text was so long that the readers cannot easily catch the major findings.

Authors' response: We thank the reviewer for his time and efforts. We have addressed his concerns in detail further below. We understand that the text may appear too long, but this is necessary due to the complexity of the statistical analyses and the multiple endpoints (we have tried to present the results of direct frequentist and mixed Bayesian analyses in a succinct order for each endpoint). Considerable part of the results is available as a supplementary material. We also note that following the advice of the 2nd reviewer, we **included another 2 RCTs (Salem et al. 2017 and Huang et al. 2017)** and hence, we had to re-run all analyses and revise all numerical results accordingly (minor decimal differences – revised figures and Tables throughout the manuscript – results overall nearly identical).

7. Unfortunately, the authors' findings were similar to several previous meta-analyses. I strongly recommend a deep discussion and comparison with similar work. A recent overview of meta-analyses regarding HCC management identified the following:

1) 7 meta-analyses compared the outcomes of TACE/TAE versus no active treatment or supportive care. Finally, TACE/TAE should be favored.

2) 3 meta-analyses compared the outcomes of TACE versus TAE. Finally, TACE was similar to TAE in term of OS.

3) 3 meta-analyses compared the outcomes of DEB-TACE versus cTACE. Finally, DEB-TACE was similar to cTACE in the term of tumor response.

4) 1 meta-analysis compared the outcomes of TACE in combination with 3D-CRT versus TACE alone. Finally, the combination therapy was superior to TACE alone in terms of 1- and 3-year survival.

5) 2 meta-analyses compared the outcomes of TACE in combination with radiotherapy versus TACE alone. Finally, TACE plus radiotherapy should be favored in term of OS.

6) 4 meta-analyses compared the outcomes of TACE in combination with sorafenib versus TACE alone. TACE plus sorafenib was not favored in term of OS.

Authors' response: We thank the reviewer for his points. We have expanded our discussion (even though the manuscript is already quite long) with an additional paragraph discussing similarities and agreements of our work (comprehensive network meta-analysis) with other individual direct meta-analytic efforts by citing the relevant papers aforementioned by the reviewer. To our knowledge, the present work combines all currently available randomized data from different treatments/strategies into ***a single unified body of evidence*** that may help guide/transform everyday practice and help change/revise national and international guidelines in the future.

Lines 586-604: "Overall, the findings of the present network meta-analysis are very much in line with the results of several individual direct meta-analyses exploring individual (chemo)-embolization strategies. A recent overview of the major findings of meta-analyses on the management of hepatocellular carcinoma summarized the body of evidence from more than 20 direct meta-analytic reports on embolization therapies for inoperable liver cancer [124]. Seven meta-analyses compared the outcomes of TACE/TAE versus no active treatment or supportive care and overall survival outcomes favoured TACE/TAE [27,33,125]. Another 3 reports compared the outcomes of TACE versus TAE and concluded that there was no survival difference [27,126,127]. Furthermore, 3 reports looked into DEB-TACE versus TACE and found benefit only in terms of tumour response like in the present work [24,128,129]. Four meta-analyses reported outcomes of TACE combined with sorafenib versus TACE alone and again found no survival benefit with the addition of sorafenib [29,130]. Last, there were 3 meta-analyses exploring the combination of TACE with plain external or conformal radiotherapy and also found that combination therapy produced superior survival outcomes [18,124]. The present work corroborates all of

the above in a single model and further raises the combination of TACE and percutaneous tumour ablation as the best treatment option in terms of both local tumour response and overall patient survival.”

8. The potential analyses and conclusions were partially overlapped. The advantages and disadvantages of different work should be discussed.

Authors' response: We believe that we have embarked into already extensive discussion of our findings in comparison to the literature and previous plain meta-analyses. In addition, the manuscript is already long enough for any further comments.

9. The authors identified two RCTs comparing the outcomes of TARE versus TACE. Two papers were published during an interval of 18 years. Over two decades, the understanding of HCC pathogenesis and management has been largely improved. Is the combination of the two studies appropriate? Please provide the difference and similarity in the study design between them.

Authors' response: All studies included in the TARE-radioembolization arm include use of *a beta-emitter* (including ^{131}I-labeled Lipiodol [77,112] or Yttrium-90 microparticles [76,78,109]). Otherwise, details about the study design and characteristics are provided in detail in the supplementary Tables 1 and 2. They all seem to be similar in terms of patient inclusion criteria. However, we do acknowledge that there are always changes in medical practice over the years that may introduce other unknown risk modifiers. In the limitation paragraph the revised manuscript reads:

Lines 648-650: Another limitation is that all 55 studies span 2 decades of medical practice and patient population reflects, as expected, the well-known clinical and anatomical heterogeneity of patients with unresectable HCC.

Reviewer #2: Very informative and properly conducted meta-analysis.

Authors' response: We thank the reviewer for his positive comment.

10. I suggest to add to the bibliography a recent meta-analysis comparing TACE and TAE: Facciorusso A, et al. Transarterial chemoembolization vs bland embolization in hepatocellular carcinoma: A meta-analysis of randomized trials. UEG Journal 2017, in press. http://journals.sagepub.com/toc/ueg/0/0

Authors' response: We have introduced several more references (including the one proposed above) in a whole new discussion paragraph as noted previously.

Lines 586-604: "Overall, the findings of the present network meta-analysis are very much in line with the results of several individual direct meta-analyses exploring individual (chemo)-embolization strategies. A recent overview of the major findings of meta-analyses on the management of hepatocellular carcinoma summarized the body of evidence from more than 20 direct meta-analytic reports on embolization therapies for inoperable liver cancer [124]. Seven meta-analyses compared the outcomes of TACE/TAE versus no active treatment or supportive care and overall survival outcomes favoured TACE/TAE [27,33,125]. Another 3 reports compared the outcomes of TACE versus TAE and concluded that there was no survival difference [27,126,127]. Furthermore, 3 reports looked into DEB-TACE versus TACE and found benefit only in terms of tumour response like in the present work [24,128,129]. Four meta-analyses reported outcomes of TACE combined with sorafenib versus TACE alone and again found no survival benefit with the addition of sorafenib [29,130]. Last, there were 3 meta-analyses exploring the combination of TACE with plain external or conformal radiotherapy and also found that combination therapy produced superior survival outcomes [18,124]. The present work corroborates all of the above in a single model and further raises the combination of TACE and percutaneous tumour ablation as the best treatment option in terms of both local tumour response and overall patient survival."

11. Even though it was published after the literature search period, i strongly suggest to include the recent RCT conducted by the Chicago group on the comparison between TACE and TARE: Y90 Radioembolization Significantly Prolongs Time to Progression Compared With Chemoembolization in Patients With Hepatocellular Carcinoma. Salem R et al, Gastorenterology 2016.

Authors' response: We thank the reviewer for his valuable suggestion. Indeed, we updated our literature search and have introduced **2 more RCTs in the revised analysis (1 in the TARE arm and 1 in the combine TACE and ablation arm)** and we have re-iterated all numerical calculations. Revised results (minor mostly changes without any change in the overall hierarchy, direction and magnitude of the results) and updated figures are presented throughout the revised manuscript.

- *Salem R, Gordon AC, Mouli S, Hickey R, Kallini J, et al. (2016) Y90 Radioembolization Significantly Prolongs Time to Progression Compared With Chemoembolization in Patients With Hepatocellular Carcinoma. Gastroenterology 151: 1155-1163 e1152.*

- *Huang C, Zhuang W, Feng H, Guo H, Tang Y, et al. (2016) Analysis of therapeutic effectiveness and prognostic factor on argon-helium cryoablation combined with transcatheter arterial chemoembolization for the treatment of advanced hepatocellular carcinoma. J Cancer Res Ther 12: C148-C152.*

12. Be careful when including in the analysis the paper by Meyer et al (ref 9) as it is performed not exactly with TACE but with chemotherapy infusion over 15 minutes followed by embolization 4-6 hours later. This aspect should be at least commented in the discussion.

Authors' response: We have acknowledged this aspect of the Meyer et al randomized study in the methods section as follows:

Lines 372-373: **"**In the TACE treated arms, conventional transarterial chemoembolization was performed with a lipiodol emulsion of a single chemotherapy agent (doxorubicin [61,68,70,73-75,78,83,86], or epirubicin [63,66,72,76,80], or cisplatin [9,62,64,67,69,71,77,82], or mitomycin [87], or a combination chemotherapy regimen [65,79,81,84,85,89,93,95,97,99-106], and was most often followed by gelfoam or other particle embolization of the primary feeding vessels**.** Meyer et al. performed cisplatin infusion first followed by particle embolization 4-6 hours later [9]."

13. I am not sure the analysis takes properly into account all the variables, such as tumor stage, treatment scheduled (whether "on demand" or pre-defined), number of sessions, response criteria adopted, and so forth.....

Authors' response: Baseline patient variables and tumour index characteristics of all included studies are provided in Table 1. We performed a random effects meta-regression analysis to search for risk modifiers and predictors that may significantly affect our results. The findings are outlined in supplementary table 5 as below.

**Table 5. Meta-regression analysis with a random effects models (95%CrI)**		
**Endpoint**	**Covariate**	**Regression coefficient**
**Serious adverse events**	Publication year	-0.050 ((-0.278) – 0.134
	Patient age	0.103 ((-0.125) – 0.338
	Male gender	-4.121 ((-16.74) – 8.043
	Child-Pugh A stage	-4.006 ((-13.15) – 3.239
	Multinodular HCC	27.35 (9.329 – 49.66)
	Follow-up period	-0.311 ((-1.295) – 0.507
**Objective response**	Publication year	-0.119 ((-0.268) – 0.010)
	Patient age	0.071 ((-0.057) – 0.195)
	Male gender	0.387 ((-7.583) – 8.740)
	Child-Pugh A stage	-2.883 ((-7.111) – 0.946)
	Multinodular HCC	61.13 (17.76 – 128.4)
	Follow-up period	0.516 ((-0.076) – 1.161
**Patient survival**	Publication year	0.004 ((-0.020) – 0.030)
	Patient age	0.012 ((-0.019) – 0.043)
	Male gender	-0.506 ((-2.643) – 1.584)
	Child-Pugh A stage	-0.002 ((-0.009) – 0.005)
	Multinodular HCC	2.914 ((-0.565) – 6.306)
	Follow-up period	0.049 ((-0.060) – 0.158)

Random effects meta-regression analyses to check for risk modifiers demonstrated only weak non-significant correlations in the majority of the tests. Multinodular HCC was the only variable found to be strongly and significantly related to increased rate of adverse events, as well as of higher rates of radiological response (Supplemental table 5).

Manuscript
Click here to download Manuscript PLOS ONE Katsanos Revised.docx

Comparative effectiveness of different transarterial embolization therapies alone or in combination with local ablative or adjuvant systemic treatments for unresectable hepatocellular carcinoma: A network meta-analysis of randomized controlled trials

Konstantinos Katsanos, MSc, MD, PhD, EBIR*,†, Panagiotis Kitrou, MD, PhD,*

Stavros Spiliopoulos, MD, PhD, EBIR ‡, Ioannis Maroulis, MD, PhD §,

Theodore Petsas, MD, PhD *, Dimitris Karnabatidis, MD, PhD, EBIR *

* Department of Interventional Radiology, Patras University Hospital, School of Medicine, Rion, 26504, Greece

† Department of Interventional Radiology, Guy's and St. Thomas' Hospitals, NHS Foundation Trust, King's Health Partners, London, SE1 7EH, United Kingdom

‡ Department of Interventional Radiology, Attikon University Hospital, School of Medicine, Athens, Greece

§ Department of Liver Surgery, Patras University Hospital, School of Medicine, Rion, 26504, Greece

Corresponding author: Dr. Konstantinos Katsanos

katsanos@med.upatras.gr, Assistant Professor, Department of Interventional Radiology, Patras University Hospital, School of Medicine, Rion, 26504, Greece, Tel: +30 2610 999218, Fax: +30 2610 999218

&

konstantinos.katsanos@gstt.nhs.uk, Honorary Consultant, Department of Interventional Radiology, Guy's and St.Thomas' Hospitals, NHS Foundation Trust, King's Health Partners, London, SE1 7EH, United Kingdom, NHS Foundation Trust, Tel: +44 (0)207 188 5550, Fax: +44 (0)207 928 8071

No conflicts of interest or relationship with the industry

Short title:

Transcatheter embolization therapies for unresectable hepatocellular carcinoma: Systematic review and network meta-analysis

Abstract

Background: The optimal transcatheter embolization strategy for patients with unresectable hepatocellular carcinoma (HCC) remains elusive. We conducted a systematic review and network meta-analysis (NMA) of different embolization options for unresectable HCC.

Methods: Medical databases were searched for randomized controlled trials evaluating bland transarterial embolization (TAE), conventional TACE, drug-eluting bead chemoembolization (DEB-TACE), or transarterial radioembolization (TARE), either alone or combined with adjuvant chemotherapy, or local liver ablation, or external radiotherapy for unresectable HCC up to June 2017. Random effects Bayesian models with a binomial and normal likelihood were fitted (WinBUGS). Primary endpoint was patient survival expressed as hazard ratios (HR) and 95% credible intervals. An exponential model was used to fit patient survival curves. Safety and objective response were calculated as odds ratios (OR) and accompanying 95% credible intervals. Competing treatments were ranked with the SUCRA statistic. Heterogeneity-adjusted effective sample sizes were calculated to evaluate information size for each comparison. Quality of evidence (QoE) was assessed with the GRADE system adapted for NMA reports. All analyses complied with the ISPOR-AMCP-NCP Task Force Report for good practice in NMA.

Findings: The network of evidence included 55 RCTs (12 direct comparisons) with 5,763 patients with preserved liver function and unresectable HCC (intermediate to advanced stage).All embolization strategies achieved a significant survival gain over control treatment (HR range, 0.42-0.76; very low-to-moderate QoE). However, TACE, DEB-TACE, TARE and adjuvant systemic agents did not confer any survival

benefit over bland TAE alone (moderate QoE, except low in case of TARE).There was moderate QoE that TACE combined with external radiation or liver ablation achieved the best patient survival (SUCRA 86% and 96%, respectively).Estimated median survival was 13.9 months in control, 18.1 months in TACE, 20.6 months with DEB-TACE, 20.8 months with bland TAE, 30.1 months in TACE plus external radiotherapy, and 33.3 months in TACE plus liver ablation. TARE was the safest treatment (SUCRA 77%), however, all examined therapies were associated with a significantly higher risk of toxicity over control (OR range, 6.35 to 68.5). TACE, DEB-TACE, TARE and adjuvant systemic agents did not improve objective response over bland embolization alone (OR range, 0.85 to 1.65). There was clinical diversity among included randomized controlled trials, but statistical heterogeneity was low.

Conclusions: Chemo- and radio-embolization for unresectable hepatocellular carcinoma may improve tumour objective response and patient survival, but are not more effective than bland particle embolization. Chemoembolization combined with external radiotherapy or local liver ablation may significantly improve tumour response and patient survival rates over embolization monotherapies. Quality of evidence remains mostly low to moderate because of clinical diversity.

Systematic review registration: CRD42016035796 (http://www.crd.york.ac.uk/PROSPERO)

Keywords

Hepatocellular carcinoma, embolization, chemoembolization, radioembolization, ablation, radiotherapy, survival, network meta-analysis, objective response, unresectable, systematic review

Abbreviations	
HCC	Hepatocellular carcinoma
RF	Radiofrequency ablation
MW	Microwave ablation
TAE	Transarterial embolization
TACE	Transarterial chemoembolization
DEB-TACE	Drug-eluting bead transarterial chemoembolization
TARE	Transarterial radioembolization
SIRT	Selective internal radiation therapy
BCLC	Barcelona Clinic Liver Cancer staging system
RCT	Randomized controlled trial
NMA	Network meta-analysis
QoE	Quality of Evidence
DIC	Deviance information criterion
EASL	European Association for the Study of the Liver
RECIST	Response Evaluation Criteria In Solid Tumors
OR	Objective response
SAE	Serious adverse events
HR	Hazard ratio
CTCAE	Common Terminology Criteria for Adverse Events
SUCRA	Surface Area Under the Cumulative Rankograms
CrI	Credible intervals

Introduction

Hepatocellular carcinoma (HCC) is the third leading cause of all cancer-related deaths globally and accounts for 90% of primary liver cancers and approximately 7% of all cancers, representing the fifth most common cancer in men and eighth for women.[1-3] Liver transplantation and surgical resection remain the proposed treatment options for very early and early stage HCC in good surgical candidates. Unfortunately, more than three-quarters of the patients are diagnosed during the intermediate or advanced stages of the disease and considered ineligible for curative resection.[1,4] In the past, the prognosis of unresectable HCC was poor and its management was limited to systemic pharmacotherapy, external radiotherapy or plain supportive treatments.[5] With the advent of Interventional Oncology that encompasses different percutaneous, image-guided, locoregional therapies,[6,7] treatment options for unresectable HCC quickly expanded to include transcatheter embolization with or without chemotherapy [8]; i.e. bland transarterial embolization (TAE)[9], conventional transarterial chemoembolization (TACE)[10] or chemoembolization with drug-eluting beads (DEB-TACE)[11]; and percutaneous liver ablation either with chemical agents like alcohol[12], or alternatively with application of radiofrequency (RF) or microwave (MW) energy.[13] Conventional TACE with the transcatheter delivery of a mixture of chemotherapy and embolic material is the current standard of care for unresectable intermediate or advanced stage HCC in patients with preserved liver function.[4,10] Local radiotherapy with the transarterial delivery of beta-emitting microparticles, currently known as radioembolization (TARE) or selective internal radiation therapy (SIRT) [14,15], is another emerging treatment for unresectable HCC. In addition, various combinations of locoregional

ablative treatments with adjuvant systemic therapies[16,17] or even external organ radiotherapy have been proposed.[18]

In general, interventional targeted embolization and ablative therapies for the treatment of unresectable HCC aim to increase overall patient survival, while limiting treatment-related side-effects, avoiding untoward complications, and improving the quality of life.[4] Theoretically, this can be accomplished by the inherent advantages of transcatheter (chemo)embolization treatments, which include a minimally invasive approach, enhanced pharmacokinetic profile and intra-tumorous bioavailability due to targeted drug delivery, and presumably more extensive tumour necrosis by combining the ischemic effect of embolization, while sparing surrounding normal liver parenchyma.[8,19] Moreover, transcatheter embolization treatments do not require general anesthesia or prolonged hospitalization periods.[3,8]

However, in spite of extensive animal and clinical investigations, and numerous randomized controlled trials (RCT) over the last decades, the optimal embolization treatment strategy for patients with intermediate to advanced stage HCC remains elusive.[7,8] The authors pursued to perform a mixed treatment comparison with quantitative statistical methods – network meta-analysis (NMA) - of the various transcatheter embolization therapies with or without local ablative or adjuvant systemic treatments for unresectable HCC. Comparative effectiveness of treatments that have or have not been directly compared with each other in head-to-head RCTs can be assessed in a network meta-analysis (NMA) using Bayesian statistics, on the condition that all competing therapies share a common chain or network of evidence.[20,21] We conducted a Bayesian network meta-analysis of all relevant randomized controlled trials to identify the best treatment option for patients with unresectable intermediate/advanced stage HCC.

Methods

Search methods

This systematic review has been registered in the PROSPERO public database (CRD42016035796; http://www.crd.york.ac.uk/PROSPERO). The authors initially collated randomized controlled trials reporting outcomes for unresectable HCC from different transarterial embolization strategies (alone or in combination with other treatments) from previously published relevant meta-analyses.[8,10,12,15,18,19,22-33] Subsequently, electronic searches of PubMed (Medline), EMBASE (Ovid), AMED, Scopus, CENTRAL, the China/Asia On Demand (CAOD) research portal, the PROSPERO and DARE meta-analyses databases as well as online material were performed until June 2017. The terms used included ‘hepatocellular carcinoma’, ‘primary liver cancer’, ‘unresectable’, ‘transcatheter’, ‘embolization’, ‘bland’, ‘chemoembolization’, ‘selective internal radiation therapy’, ‘radioembolization’, ‘radiotherapy’, ‘ablation’, ‘radiofrequency’, ‘alcohol’, ‘TAE’, ‘TACE’, ‘DEB-TACE’, ‘TARE’, ‘SIRT’, ‘sorafenib’, ‘bevacizumab’, ‘drug-eluting’, ‘anti-angiogenic’, ‘randomized’, ‘controlled trial’, and ‘meta-analysis’ along with the pertinent Medical Subjects Headings (MeSH) and combinations thereof with Boolean syntax. Keywords were searched using both British English and American English grammar (e.g. embolisation & embolization). In addition, Interventional Radiology, Medical Oncology and Radiation Oncology peer-reviewed journals in PubMed and Embase were examined. There were no restrictions on language, date or type of publication. KK, PK and SS performed the literature search and data extraction.

Trial selection and good meta-analysis practice

All steps of the trial selection process complied with the PRISMA (Preferred Reporting Items for Systematic reviews and Meta-Analyses) statement.[34] We searched for and included only RCTs comparing any of the aforementioned endovascular devices with each other, and reporting any of the primary and/or secondary outcome measures as defined below. RCTs were assessed for inclusion in the network meta-analysis (NMA) using a specifically structured question checklist developed in consensus by all authors. Published and unpublished randomised trials with an open-label, single-blind or double-blind design were eligible for inclusion provided that they investigated any type of transcatheter arterial embolization for unresectable hepatocellular carcinoma; with or without chemotherapy, plain or drug-eluting beads, radioactive embolic material; as a stand-alone treatment or in combination with other types of locoregional ablation; chemical or thermal or external radiotherapy; or combined with adjuvant systemic treatments; anti-angiogenic molecules or other agents. RCTs were included provided they reported any of the agreed outcome measures (see endpoints below).

A standardized data extraction form was used to collect the following information from all included trials (by KK, PK and SS): (1) characteristics of the study design methods (randomization, blinding, concealment of allocation, drop-outs, outcome reporting, risk of bias); (2) patient sample size and baseline clinical characteristics (age, gender, tumour size and morphology, liver function, vascular invasion, and performance status); (3) HCC staging according to the Okuda, BCLC, JIS or TNM classification systems; (4) description of active and control interventional treatment (chemotherapy regimen, type of embolic agents, treatment courses, dose and

fractionation of radiotherapy, adjuvant anticancer agents, other ablation procedures); and (5) clinical outcomes including overall patient survival, objective response of the treated index tumours, and serious adverse events. Terminology and classification of percutaneous and transcatheter image-guided liver therapies complied with standardized nomenclature and universal reporting criteria proposed by the Society of Interventional Radiology Technology Assessment Committee.[35]

The quality of the RCT trials was assessed independently by two of the authors with the Cochrane Collaboration’s tool for evaluating the risk of bias that examines 7 different methodological items including randomized sequence generation, allocation concealment, blinding of patients and investigators, completeness and selectivity of outcome reporting, and other potential sources of bias.[36] Risk of bias assessment was performed by KK, SS and DK. To help inform healthcare decision making, all analysis methods, reporting quality and interpretation of findings complied with the 26-domain questionnaire of the ISPOR-AMCP-NCP Task Force Report for good practice in indirect treatment comparisons and NMA.[37] Finally, the quality of evidence (QoE) was assessed with Grading of Recommendations Assessment, Development and Evaluation (GRADE) system as adapted for the rating of pooled effect estimates in the case of NMA studies,[38,39] which considers directness, heterogeneity and imprecision of the mixed treatment comparisons as potential reasons for downgrading of the level of confidence.

Endpoints

In terms of survival outcome measures, few studies were found to report progression-free survival. Therefore, the primary endpoint was set at overall patient survival that was uniformly reported by all studies and was synthesized on the log-hazard scale as indicated for time-to-event outcomes in cancer studies.[40,41] Study-specific Hazard Ratios (HRs) and respective variances were retrieved from individual publications or back-calculated from the summary or Kaplan-Meier time-to-event data and quoted log-rank statistics with the equations of Parmar et al.[42] and methods of Tierney et al.[43]. If hazard rates were not available, HR was approximated from event rates under the assumption of constant hazards. Random effects models were fitted to account for clinical diversity and heterogeneity and HRs with 95% credible intervals were calculated.

Treatment effectiveness was assessed by the radiologic response on cross-sectional follow-up imaging as reported by each individual RCT. The effectiveness endpoint was set at Objective response (OR) of the index tumour defined as Complete and Partial Response (CR+PR) according to well-accepted classification systems including the World Health Organization (WHO),[44] the European Association for the Study of the Liver (EASL),[45] the Response Evaluation Criteria In Solid Tumors (RECIST),[46] and modified RECIST (mRECIST)[47] schemes.

All outcome measures of this systematic review were defined according to previously published terminology and accepted reporting criteria for transcatheter therapies for liver malignancies.[35] The safety and toxicity endpoint was set at Serious Adverse Events (SAE) grade 3 and above as defined by the National Cancer Institute Common Terminology Criteria for Adverse Events (CTCAE, version 4.0).[48] All

endpoints were analyzed on an intention to treat basis as recommended for reporting and meta-analysis of RCTs. Any disagreements were resolved by consensus.

Statistical methods

Direct pairwise meta-analyses of head-to-head comparisons were performed using standard frequentist approaches (RevMan 5.2, Cochrane Collaboration). Mixed treatment comparisons of the RCT network were performed with Bayesian inference (WinBUGS 1.4.3, MRC Biostatistics Unit at Cambridge, United Kingdom). Bayesian hierarchical modeling of the present network meta-analysis complied with the NICEDSU (National Institute for Health and Excellence Decision Support Units) guidelines.[49-51] Count statistics of treatment toxicity and objective tumour response were analyzed with a Bayesian random effects model with a binomial likelihood to calculate relative treatment effects expressed as Odds Ratios (OR) between different treatments. Overall patient survival was analyzed with a Bayesian random effects model with a normal likelihood incorporating log hazard ratio statistics from individual trials to calculate Hazard Ratios (HR) between competing treatments.[40] Summary statistics of relative treatment effects are reported as the median and accompanying 95% Credibility Intervals (95% CrI) of the posterior distribution. CrIs serve the same purpose as confidence intervals in frequentist statistics.

In addition, we fitted the respective patient survival curves with an exponential model up to 5 years using absolute survival estimates of conventional TACE, which was the most common comparator and with the largest sample size, as the anchor treatment. Median patient survival (half-life) for each treatment was calculated by combining the fitted hazard rate (exponential decay constant) of the anchor treatment (random effects model) with the pairwise posterior median HR calculated by the Bayesian model for the respective treatment. We also constructed rankograms of cumulative rank probabilities of how each treatment ranks against each other in terms of being

the 1st, 2nd, 3rd, etc. best treatment option. We present hierarchies of the effectiveness and safety of competing treatments based on their cumulative rank probabilities and the Surface Area Under the Cumulative Rankograms (SUCRA) as proposed by Salanti et al.[52]

The information size (IS) required for a valid meta-analysis may be assumed to be at least as large as the sample size of a single well-powered RCT designed to confirm or reject the null hypothesis [53,54]. To assess the adequacy of available information size across different pairwise comparisons that combined direct and indirect evidence within the NMA framework, we performed calculations of the effective sample size for each treatment comparison. We employed the methods proposed by Thorlund and Mills for quantifying sample and information size in NMAs after adjusting for statistical heterogeneity observed in pairwise meta-analyses of individual nodes [55]. Consequently, statistical power and strength of evidence for each treatment comparison may be evaluated by the information fraction (IF; percentage of information size) available for each comparison.

Heterogeneity, consistency, and meta-regression

Heterogeneity was evaluated with the posterior median of the between-trials standard deviation (σ),[50] while small study effects and publication bias were evaluated by visual inspection of standard and comparison-adjusted funnel plots.[56] Because of conceptual differences in study designs and anticipated diversity in baseline demographics, the observed baseline risk of outcome measures may vary between the reference treatment arms. Baseline risk is a proxy for unmeasured but important patient-level characteristics that may relate to significant clinical heterogeneity. Hence, we extended our analysis to a meta-regression model on trial-specific baseline risk of the control arms to account for the uncertainty and clinical heterogeneity introduced by differences in baseline characteristics of unresectable HCC cohorts.[57] In addition, extensive consistency, sensitivity, and meta-regression analyses were performed to explore heterogeneity and confirm validity as proposed by the ISPOR-AMCP-NCP Task Force.[37,50] The validity and robustness of NMA depend largely on the distribution of effect modifiers (covariates) not only between studies with the same contrast (i.e. heterogeneity in the case of standard pairwise meta-analysis) but also between different contrasts (i.e. inconsistency between direct and indirect contrast estimates).[58] Any disagreement between the direct evidence available for a specific contrast and the indirect evidence inferred by the rest of the network would give rise to inconsistency. In the case of NMA studies, the risk of network inconsistency is greatly reduced if between-trials heterogeneity is low.[59] To exclude any loop-specific inconsistency and confirm the transitivity assumption, pairwise direct and indirect effect estimates of closed loops of evidence were inspected for any disagreement and the results of the consistency model were

compared with those of an alternative unrelated mean effects model without any consistency constraints.[49]

WinBUGS modeling

Bayesian inference with WinBUGS employs Markov Chain Monte Carlo (MCMC) simulation to calculate the posterior distributions of the interrogated nodes within the framework of the chosen model and likelihood function on the basis of prior assumptions. For the purposes of this analysis, we first fitted a Bayesian hierarchical model for multiple comparisons of different treatment options control best supportive treatment as the reference. Posterior medians (95% CrI) of the point estimates against control treatment were calculated using the freely available NetMetaXL software package[60], and by custom code following the examples of Woods et al.. [40] Vague priors were used for all treatment effects and for between-trials heterogeneity variance to avoid bias.

Three Markov chains were compiled and run, while convergence was confirmed with the Brooks–Gelman–Rubin diagnostic tool and by inspection of history plots of monitored nodes. An initial burn-in simulation of 50,000 iterations was discarded and inference of final summary statistics was based on simulation of an additional 100,000 iterations.[51] Global model fit and parsimony was compared between different fitted models to decide on the most accurate model. The goodness of fit was compared with the posterior mean of the total residual deviance and the Deviance Information Criterion (DIC) criterion. Residual deviance must approximate the total number of study arms analyzed in the case of a good model fit the and generally the model with the lowest DIC is preferred.[51] The level of statistical

significance was set at $\alpha=0.05$ for frequentist inference, while relative treatment effect results associated with 95% CrI that did not cross unity were considered significant in the case of Bayesian inference.

Results

Network of evidence

Following the PRISMA selection process, 5,975 scientific records were screened for potential inclusion in the network meta-analysis on the basis of their title and abstract (Figure 1). Finally, 55 RCTs (including one three-arm study [61]) published between 1988 and 2017 and reporting on 5,763 patients in total were included and synthesized within a Bayesian framework. The network of evidence involved nine treatment nodes (eight active and one control) and was well connected with conventional TACE as the most common comparator (Figure 2). Four treatment nodes referred to different types of trans-arterial embolization therapy alone (conventional TACE, or DEB-TACE, or TARE, or bland TAE) and another four treatment nodes referred to a combination of transarterial chemoembolization with other locoregional or systemic treatments (TACE and external radiotherapy, or TACE and percutaneous liver ablation, or TACE and adjuvant systemic, or DEB-TACE and an adjuvant systemic agent). Direct evidence was available for 12 comparisons (Table 1); three of them were informed by a single RCT and the rest by more than one RCT (median 3.5; range, 1-11 trials).

TACE was investigated versus Control symptomatic treatment in 8 studies [61-68], versus bland TAE in 4 studies [9,69-71], versus DEB-TACE in 4 studies [72-75], versus TARE in 3 studies [76-78], versus TACE combined with adjuvant systemic agents in 8 studies [17,79-85], versus TACE combined percutaneous liver ablation in 10 studies [86-95], and versus combined TACE and external radiotherapy in 11 studies [96-106]. In addition, DEB-TACE was compared directly with TAE in 2 studies [107,108], with TARE in 1 RCT [109], and with DEB-TACE plus systemic

sorafenib in 1 RCT [16]. Finally, TAE alone was compared with Control treatment in 3 studies [61,110,111], and TARE with Control in 1 study [112]. There were 3 high-quality RCTs with low risk of bias; the rest of the studies had unclear (at least one unclear domain) to high (at least one high-risk domain) risk of bias according to the COCHRANE tool for risk of bias assessment. The latter was caused by performance bias (absent or unclear blinding of participants and personnel) or detection bias (blinded outcome assessment) in the majority of the studies.

Fifty-one out of the 55 studies recruited patients with unresectable hepatocellular carcinoma classified as intermediate to an advanced stage (i.e. BCLC stage B-C, Okuda stage I-II, or AJCC TNM stage II-III) and 4 studies included unresectable early stage HCC [74,78,100,105]. All studies included patients with preserved liver function (Child-Pugh A and B) and with a predominantly male gender (range, 50-96%). Good performance status (PS: 0-1 or KPS≥65%) was reported in most of the cases and the percentage of randomized patients with a multinodular or diffuse type of HCC varied widely (median, 57%; IQR, 39-67%; max 100%). Fourteen out of the 55 studies reported inclusion of variable rates of patients with documented portal vein thrombosis (range, 2-100%). A detailed description of baseline patient demographics and clinical characteristics is provided in Supplemental Table 1.

In the TACE treated arms, conventional transarterial chemoembolization was performed with a lipiodol emulsion of a single chemotherapy agent (doxorubicin [61,68,70,73-75,78,83,86], or epirubicin [63,66,72,76,80], or cisplatin [9,62,64,67,69,71,77,82], or mitomycin [87], or a combination chemotherapy regimen [65,79,81,84,85,89,93,95,97,99-106], and was most often followed by gelfoam or other particle embolization of the primary feeding vessels. Meyer et al. performed cisplatin infusion first followed by particle embolization 4-6 hours later [9]. In case of

TAE, bland embolization was performed with gelfoam and/or microparticles (microspheres) [9,61,69,70,107,108,110,111] or alcohol [71]. DEB-TACE involved transcatheter delivery of doxorubicin-eluting DC beads [16,72-75,107-109], and TARE of a beta-emitter including ^{131}I-labeled Lipiodol [77,112] or Yttrium-90 microparticles [76,78,109]. Adjunctive systemic agents included sorafenib [16,81,84], brivanib [17], bevacizumab [79,83], arsenic trioxide [85], TSU-68 [80], IFN-a [82]. Locoregional liver ablation was reported by means of multiple sessions of radiofrequency ablation (RFA) [88,89,93,94] or percutaneous ethanol injection (PEI) [86-88,90-92] or argon-helium cryoablation [95]. Finally, external radiotherapy was delivered by 3D conformal [97,98,100,104-106] or moving stripe fractionated protocols [99,101-103]. Active and control treatment protocols are described in detail in Supplemental Table 2. Median follow-up was 3 years on a trial basis (interquartile range, 2.0–3.5 years; max 6.0 years).

Patient Survival

Survival outcomes were reported by 51 RCTs (incl. one 3-arm) reporting on 5,394 patients and 12 direct comparisons in total. Direct meta-analyses (Figure 3) confirmed a significant survival benefit of TACE over best supportive therapy (HR: 0.76; 95%CI: 0.64-0.91) and a similar survival benefit between TAE and TACE (HR: 0.87; 95%CI: 0.71-1.07). In addition, TACE performed worse than TACE plus radiotherapy (HR: 0.60; 95%CI: 0.53-0.69) and TACE plus ablation (HR: 0.54; 95%CI: 0.46-0.65). The NMA synthesis showed that all embolization treatments achieved a significant survival benefit over control except DEB-TACE with adjuvant sorafenib (HR range, 0.42-0.76). Figure 4 shows a hierarchy of different treatments

according to the SUCRA statistic and the respective Hazard Ratios (HR). TACE, DEB-TACE, TARE, and adjunctive systemic agents (combined with TACE or DEB-TACE) did not confer a survival benefit over bland TAE. TACE combined with external radiation therapy (SUCRA 86%), or percutaneous tumour ablation (SUCRA 96%), were the most effective treatment strategies. NMA heterogeneity was low (σ= 0.06; 95%CrI: 0.001-0.17). A league table of all pairwise survival comparisons from the NMA synthesis is provided in the Supplemental material.

Survival model

The fitted exponential survival model is shown in Figure 5 (posterior median of survival projections; 95% CrIs). Conventional TACE was the most common comparator node (43 out of the 51 RCTs reporting patient survival) and was used as the anchor treatment (least squares non-linear fit R^2=0.999) for calculating expected median survival outcomes for each of the other treatment options. Median survival period in case of control best supportive treatment was 13.9 months (95%CI: 11.0-17.7) and increased to 18.1 months (95%CI: 15.6-21.6) in the case of TACE, 20.6 months (95%CI: 14.5-29.4) with DEB-TACE, and 20.8 months (95%CI: 16.2-27.1) with bland TAE. Adjuvant systemic agents did not provide any significant survival benefit over transarterial therapies. Median survival increased to 24.3 months (95%CI: 16.8-35.3) in the case of TARE. Projected median survival exceeded 30 months when conventional TACE was combined with external radiotherapy (30.1 months; 95%CI: 24.6-37.3) or with percutaneous liver tumour ablation (33.3 months; 95%CI: 26.4-42.5).

Objective Response

Rates of the objective response of the treated tumour lesions were reported by 41 RCTs including 4,669 patients and informing 10 direct treatment comparisons. According to direct meta-analyses (Figure 6), both TACE (OR: 5.95; 95%CI: 2.96-11.99) and TAE (OR: 45.8; 95%CI: 8.75-239.7) demonstrated a strong response rate over control treatment. In line with the survival analysis, objective response was also better in case of TACE combined with radiotherapy (OR: 3.7; 95%CI: 2.7-5.0) or ablation (OR: 9.44; 95%CI: 5.14-17.3) over TACE alone. In the NMA analysis, all embolization treatments achieved a significant tumour response. Figure 7 shows a hierarchy of comparative treatment effectiveness according to the SUCRA statistic. Combinations of conventional TACE with external radiation therapy (SUCRA 85%) or percutaneous tumour ablation (SUCRA 99%) were the most effective treatment options. TACE, DEB-TACE, TARE and adjunctive systemic agents (combined with TACE or DEB-TACE) did not improve the objective response of treated tumours compared to bland embolization alone (TAE). TACE with adjunctive ablation achieved a significantly better objective tumour response compared to all other embolization mono- or combination therapies (OR range, 2.17-10.2; league table in the Supplemental material). NMA heterogeneity was low (σ= 0.29; 95%CrI: 0.03-0.63). Comparative effectiveness results of overall patient survival were corroborated by the hierarchical SUCRA results of tumour objective response with high correlation between the two outcome measures (linear regression fit R^2=0.959 – Figure 8).

Serious Adverse Events

Treatment-related serious adverse events (SAE) were reported by 32 RCTs including 3,610 patients for 11 direct treatment comparisons (Figure 9). Safety ranking of different embolization therapies on the basis of cumulative rank probabilities (SUCRA, %), along with the respective ORs (95%CrI) against control as a reference, are shown in Figure 10. TARE was the safest treatment (SUCRA 77%), however, all examined therapies were associated with a significantly higher risk of SAE compared to control (OR range, 6.35-68.5). Most of the other pairwise comparisons showed no significant differences between different embolization regimes in terms of SAE. TACE combined with adjuvant systemic therapies was the highest-risk treatment (SUCRA 10% - league table in the Supplemental material). Between-trial heterogeneity was low ($\sigma = 1.01$; 95%CrI: 0.61-1.64).

Heterogeneity, consistency, and meta-regression

There was good agreement between the consistency and inconsistency (unrelated mean effects) models, suggesting a robust and homogeneous network of evidence (Supplemental table 3). Between-trial statistical heterogeneity in the random effects Bayesian models was low compared to the respective posterior treatment effects (Supplemental table 4). Consequently, application of a fixed effect Bayesian model produced similar numerical results with slightly tighter credible intervals (Supplemental league tables). However, model fit according to the residual deviance and DIC criteria was better in the case of the random effects analyses and hence those were preferred and presented in the present article (Supplemental table 4). There was no obvious asymmetry at visual inspection of funnel plots to suggest publication bias, except in the case of Objective Response (Supplemental funnel plots). However, that was not evident any more on the comparison- adjusted funnel plot (Supplemental OR funnel plot with comparison-specific adjustments). Random effects meta-regression analyses to check for risk modifiers demonstrated only weak non-significant correlations in the majority of the tests. Multinodular HCC was the only variable found to be strongly and significantly related to increased rate of adverse events, as well as of higher rates of radiological response (Supplemental table 5).

Strength and Quality of evidence

We calculated a sample size of 560 patients as adequate for the detection of a treatment effect of 30% relative risk reduction of death (HR=0.7) with a type I error 5% and type II error 20% (power 80%) assuming an average patient survival of 50% at 2 years and a 10% rate of drop-outs or lost to follow-up. Compared to that, the IF was found to be low-to-modeate (range, 4-51%) in case of TARE, and high (range, 50-100%) in all mixed treatment comparisons informed by both direct and indirect evidence. Figure 11 summarizes the strength (effective sample size and IF) and QoE according to the GRADE system for all treatment comparisons in the present NMA.

The GRADE system for assessing quality of evidence considers directness, heterogeneity and imprecision of the mixed treatment comparisons as potential reasons for downgrading the level of confidence in NMA results [113]. We have found no inconsistency and statistical heterogeneity was generally low in the present NMA, however, clinical diversity was evident in the baseline demographics of different RCTs. Hence, in the current analysis, QoE was first downgraded universally because of between-trial diversity in terms of baseline patient characteristics and type and mixture of antineoplastic and/or embolic agents used (Supplemental tables 1 & 2). Second, it was further downgraded in certain comparisons because of the absence of direct comparative evidence (indirectness).

To evaluate imprecision, we gauged the effective sample size and information fraction of each comparison. We considered an IF<50% as a measure of weaker evidence and potential imprecision; hence, QoE was further downgraded to very low in the relevant comparisons. Overall, there was moderate QoE with sufficient information size when comparing TACE+ablation, TACE+RT, TACE+adjuvant

systemic agents and TAE, over TACE alone. Information was also strong enough with moderate QoE in the case of TARE versus TACE, in the cases of TAE compared with control or TACE or DEB-TACE, and in the case of TACE over control treatment (Figure 11).

Discussion

Contrary to a standard meta-analysis that pools studies comparing a certain pair of treatments, network meta-analysis (NMA) is an established methodology capable of inferring the high level of evidence about any number of treatments by combining direct and indirect randomized comparative research into a single unified analysis while respecting randomization of individual clinical studies.[114] To our knowledge, this is the first comprehensive mixed treatment comparison analysis evaluating the safety and effectiveness of different transarterial embolization therapies either alone or in combination with local ablative or adjuvant systemic treatments for unresectable hepatocellular carcinoma. Most of the patients with hepatocellular carcinoma are diagnosed late at the intermediate-advanced stages of the disease and are ineligible for potentially curative treatments like liver transplantation, resection or curative thermal ablation. According to GIDEON, the largest global observational registry of unresectable HCC to date including more than 3,200 cases, more than half of all HCC patients receive TACE as their primary treatment mode [115]. A lipiodol emulsion of an anticancer agent; usually doxorubicin; followed by gelfoam or other particle embolization remains the most popular form of TACE [8]. Adoption of TACE with an oil emulsion of antineoplastic agents has been primarily driven by early RCTs of bland TAE or TACE versus conservative management more conducted than 10 years ago [8,61,64,67,68,110,111]. However, not only new treatments have emerged like DEB-TACE or TARE or combined locoregional treatments, but above all guideline-recommended therapy for unresectable HCC remains controversial. The ESMO-ESDO guidelines advocate TACE for large or multinodular HCC with good liver function [116], whereas the Canadian CEPO (Comité de l'évolution des pratiques en oncologie) recommends TACE as the standard of care for palliative

treatment of eligible HCC patients, but specifically advises against the use of TAE or TARE [117]. In the meantime, a recent heavily disputed Cochrane meta-analysis questioned the firmness of evidence supporting either TAE or TACE in unresectable HCC in general [33]. Hence, the survival benefit of transarterial embolization therapies for unresectable HCC is still under dispute [118].

Most importantly, the present NMA of 55 RCTs comprising more than 5,700 patients has shown that transarterial (chemo)-embolization strategies can confer a clear survival benefit in patients with unresectable HCC by reducing the hazard of death in the range of 24% (in case of TACE) up to 34% (in case of TAE and DEB-TACE). However, surprisingly, none of the transcatheter chemo-embolization options (i.e. TACE and DEB-TACE as standalone treatments or even combined with adjuvant systemic agents) was any better than traditional bland transarterial embolization (TAE). The above findings had a large information size and moderate QoE being supported by direct evidence by 3 trials examining TAE versus best supportive therapy (publication date 1988-2002)[61,110,111], 4 trials testing TAE versus TACE (1994-2014)[9,69-71], and 2 trials comparing TAE versus DEB-TACE (2010-2016)[107,108]. Internal radiation therapy (TARE) produced an even higher survival benefit (43% reduction of the hazard of death) informed by 3 trials [76-78], but its effectiveness was not significantly better than TAE and evidence was informed only by a moderate information size (very low-to- moderate QoE).

The aforementioned findings, on one hand, support the notion that ischemic necrosis induced by transcatheter embolization of the tumour feeding arteries is the primary mode of therapy in HCC and on the other hand question the need for the widely employed use of antineoplastic agents (most often doxorubicin) as part of the majority of HCC embolization regimens. Neoangiogenesis is a well-known hallmark

of hepatocellular carcinoma [119], and hepatic transarterial embolization induces virtually immediate tumour cell death evident on imaging within 24hours [107]. The addition of chemotherapy has been long thought to allow for enhanced intratumoral drug delivery and retention when combined with transarterial ischemic necrosis [120], but HCC is notorious for its low sensitivity to chemotherapy and tendency to develop multidrug resistance [121]. The current results have found moderate QoE according to the GRADE system that TAE is as good as any other chemo-embolization treatment contesting the widespread use of intra-arterial doxorubicin and other chemotherapeutic results.

Another interesting result was that the addition of locoregional ablation in the form of percutaneous ablation or external radiotherapy had a strong additive effect in improving objective response and prolonging patient survival. The combination of TACE with external radiotherapy achieved better response rates (SUCRA 85%) and improved patient survival (SUCRA 86%) that were both significantly better than plain TAE or TACE (low-to-moderate QoE, and IF 61-100%). The combination of TACE with some form of percutaneous ablation (microwave or RF or alcohol) was also significantly better than TAE or TACE and was found to be the best performing treatment ranking first in terms of both OR (SUCRA 99%) and survival (SUCRA 96%). The latter findings support the enhanced therapeutic outcomes in case of combined transarterial and locoregional ablative treatments [18]. Pathology studies have shown that palliative transarterial lipiodol-based treatments may achieve >90% necrosis in widely variable rates; 26-70% of the treated nodules; depending on technique, lesion size and arterial anatomy [122,123]. Hence, it would be very sensible to combine (chemo)-embolizations with other ablative therapies in order to achieve higher rates of tumor necrosis and thereby prolong patient survival.

Comparative safety analysis demonstrated that TARE with a beta-emitter was the safest treatment (SUCRA 77%), whereas combined TACE and liver ablation had the most favourable safety and effectiveness profile (SUCRA 59% and 99%, respectively).

Overall, the findings of the present network meta-analysis are very much in line with the results of several individual direct meta-analyses exploring individual (chemo)-embolization strategies. A recent overview of the major findings of meta-analyses on the management of hepatocellular carcinoma summarized the body of evidence from more than 20 direct meta-analytic reports on embolization therapies for inoperable liver cancer [124]. Seven meta-analyses compared the outcomes of TACE/TAE versus no active treatment or supportive care and overall survival outcomes favoured TACE/TAE [27,33,125]. Another 3 reports compared the outcomes of TACE versus TAE and concluded that there was no survival difference [27,126,127]. Furthermore, 3 reports looked into DEB-TACE versus TACE and found benefit only in terms of tumour response like in the present work [24,128,129]. Four meta-analyses reported outcomes of TACE combined with sorafenib versus TACE alone and again found no survival benefit with the addition of sorafenib [29,130]. Last, there were 3 meta-analyses exploring the combination of TACE with plain external or conformal radiotherapy and also found that combination therapy produced superior survival outcomes [18,124]. The present work corroborates all of the above in a single model and further raises the combination of TACE and percutaneous tumour ablation as the best treatment option in terms of both local tumour response and overall patient survival.

We consider the fitted survival model another particular strength of the present study as it may provide absolute expected median survival outcomes for each treatment

and help clinicians optimize their decision-making process as well as guide the informed consent of the patients. A previous meta-analysis of the expected survival rates of untreated patients in the control arms of randomized studies of HCC has provided interesting insights into the natural history of this largely heterogeneous patient group. Projected median survival was 12 months in the case of intermediate stage (BCLC category B) cases, and around 6 months in the case of advanced stage (BCLC category C) patients [131]. A recently released systematic review and meta-analysis of more than 10,000 patients with unresectable HCC treated with lipiodol TACE has reported a weighted median survival rate of 19.4 months (95%CI: 16.2-22.6 months) [8]. The above numbers compare favourably with the results of our comparative survival model. In the present analysis, the weighted median survival was calculated to be 13.9 months (95%CI: 11.0-17.8 months) across the control arms of best supportive care and projected to be 18.1 months (95%CI: 15.6-21.6 months) in the TACE arms (anchor treatment). The ESMO-ESDO guidelines quote an expected median survival following TACE treatment of approximately 20 months in the case of BCLC intermediate stage and no more than 11 months in the case of advanced stage HCC. Hence, the authors consider the current evidence synthesis to reflect mostly a population of predominantly intermediate stage hepatocellular carcinoma in line with guideline-recommended use of most transarterial embolization therapies. In parallel with comparative effectiveness results, expected survival outcomes were similar between TAE (median 20.9 months) and different TACE approaches (median range, 18.1-23.1 months), numerically better with TARE (median 25.4 months) and significantly improved with the addition of external radiotherapy or ablation (median >30 months).

Arguably, unresectable HCC is characterized by significant heterogeneity in lesion size, unifocal or multinodular or diffuse patterns of disease, and variable degrees of underlying liver dysfunction [5,8,131]. Experts have long advised against TACE in Child-Pugh B patients, whereas TARE and external radiation have been proposed for the more liver dominant types of disease. Hence, one treatment type cannot fit all this heterogeneous category of patients [132]. The authors believe that combination treatments customized to individual patient profiles on the basis of the presented treatment rankings may deliver better clinical results and further improve survival of patients presenting with unresectable HCC and preserved liver function. Most interestingly, we have shown a clear synergy between transarterial embolization and locoregional ablation that needs to be explored further in larger scale studies in properly selected patients.

There are certain limitations to the present analysis. Network meta-analyses are inherently more prone to uncertainty and bias compared to classical meta-analysis. In addition, network meta-analyses are often exploratory to identify areas for more targeted scientific research and to help inform the design of future RCTs. However, sensitivity, consistency, and heterogeneity analyses support the validity of our results. Another limitation is that all 55 studies span 2 decades of medical practice and patient population reflects, as expected, the well-known clinical and anatomical heterogeneity of patients with unresectable HCC. Nonetheless, our survival model is in close agreement with real-life practice supporting the notion of generalizability of our findings. Finally, we have not accounted for differences in the race and geography as certain clusters of studies were most often performed in Asia (e.g. a combination of TACE and external irradiation) or the Western countries (e.g. TACE and DEB-TACE options).

In conclusion, TACE, DEB-TACE, TARE and adjuvant systemic agents neither improved tumour objective response nor conferred any patient survival benefit compared to bland particle embolization (TAE). Combinations of TACE with external radiation or liver ablation achieved the best tumour response and patient survival. Therefore, the current trends of chemoembolization practise are clearly open to question and international guidelines may need to be revised. However, quality of evidence remains low to moderate, and clearly more and larger studies are needed, especially in the fields of radioembolization, on the role of new embolic particulate agents and to further elucidate the synergy of combined transarterial and ablative liver treatments.

Author contributions

All authors have made significant contributions to the submitted work by participating in the conceptualization of the present meta-analysis, selection of the included trials and abstraction of the relevant data, drafting, revision and final approval of the submitted manuscript. The corresponding author was personally responsible for all Bayesian statistical modeling and preparation of the initial manuscript draft. All authors meet authorship criteria according to the ICMJE recommendations: (1) substantial contributions to conception and design, acquisition of data, or analysis and interpretation of data; 2) drafting the article or revising it critically for important intellectual content; 3) final approval of the version to be published, and 4) agreement to be accountable for all aspects of the work in ensuring that questions related to the accuracy or integrity of any part of the work are appropriately investigated and resolved.

Funding

There was no funding source for this study. All authors had unrestricted access to the datasets and can take responsibility for the integrity of the data and the accuracy of the data analysis. The lead and corresponding author (K.K.) had access to the whole dataset, performed all statistical analyses and has final overall responsibility for the submitted version of the manuscript (study guarantor). The lead and corresponding author (K.K.) affirms that the manuscript is an honest, accurate, and transparent account of the study being reported. All raw data are provided in the direct frequentist plots provided in the Supplementary material. WinBUGS code and other statistical files used are available on request by the authors.

Disclosures

None of the authors has any conflicts of interest to declare. All authors have completed the ICMJE uniform disclosure form at www.icmje.org/coi_disclosure.pdf and declare that: (1) none of them have received support from any company for the submitted work; (2) none of them have any relationships with companies that might have an interest in the submitted work in the previous 3 years; (3) their spouses, partners, or children have no financial relationships that may be relevant to the submitted work; and (4) none of them have any non-financial interests or other relationships or activities that could appear to have influenced the submitted work.

Search strategy

1 “hepatocellular carcinoma”[MESH], 2 “hepatocellular carcinoma”[TW], 3 “liver cancer”[MESH], 4 “liver cancer”[TW]

5 “unresectable”[TW], 6 “inoperable”[TW], 7 “advanced”[TW]

8 “Clinical trial”[Mesh], 9 “Randomized Controlled Trial”[Mesh], 10 “Clinical trial”[TW], 11 “Randomized”[TW], 12 “Meta-analysis”[Mesh], 13 “Meta-analysis”[TW]

14 “embolization”[MESH], 15 “chemoembolization”[MESH], 16 “sorafenib”[MESH], 17 “embolization”[TW], 18 “chemoembolization”[TW],19 “sorafenib”[TW], 20 “transcatheter” [TW], 21 “ablation”[TW], 22 “radiotherapy”[TW], 23 “radiation”[TW], 24 “radioembolization”[TW], 25 “selective internal radiation therapy”[TW], 26 “radiofrequency”[TW], 27 “alcohol”[TW], 28 “drug-eluting”[TW], 29 “anti-angiog*”[TW], “bevazicumab”[TW], 30 “TACE”[TW], 31 “TAE”[TW], 32 “DEB-TACE”, 33 “TAE”[TW], 34 “SIRT”[TW], 35 “TARE”[TW]

Search String

(#1 OR #2 OR #3 OR #4) AND

(#5 OR #6 OR #7) AND

(#8 OR #9 OR #10 OR #11 OR #12 OR #13) AND

(OR #14 OR #15 OR #16 OR #17 OR #18 OR #19 OR #20 OR #21 OR #22 OR #23 OR #24 OR #25 OR #26 OR #27 OR #28 OR #29 OR #30 OR #31 OR #32 OR #33 OR #34 OR #35)

S1 Supplementary material and supporting information. Supplemental material containing **Table 1.** Included randomized controlled trials and baseline patient characteristics, **Table 2.** Active and control treatment received in the randomized controlled trials, **Table 3.** Inconsistency analysis, **Table 4.** Heterogeneity and model fit, **Table 5.** Random effects metaregressions analyses, **League tables** with fixed and random effects models for all endpoints, and **Funnel plots** (comparison-adjusted) to assess publication bias.

S2 Supporting information. PRISMA checklist

Figure legends

Figure 1. PRISMA flowchart. Trial selection process according to the PRISMA statement.

Figure 2. Network of evidence. Straight black lines denote direct head-to-head randomized comparisons. Numbers refer to the number of RCTs with direct comparisons available for each link and the size of circles is proportional to the pooled sample size (patients) available for each treatment node.

Figure 3. Patient survival. Forest plots (random effects) of direct frequentist analyses (RevMan, Cochrane). Risk of bias assessment by the Cochrane Collaboration tool is presented as well.

Figure 4. Patient Survival network meta-analysis (Random effects forest plot). Different treatments are reported in order of efficacy ranking according to the SUCRA statistic. Black circles denote the posterior median and the black lines denote the associated 95% CrI. Numbers represent hazard ratios (HR) and 95% CrIs. The combination of TACE and ablation was found to be the most effective treatment (SUCRA 95%).

Figure 5. Survival model. Projected survival curves for each treatment were fitted with an exponential model up to 5 years. Conventional TACE was the most common comparator in the overall network of evidence and was used as the anchor treatment because it had the largest sample size. Absolute survival estimates of TACE at different time points were calculated with a standard random effects proportional model weighted by patient sample for each trial (black circles). Median patient survival (half-life) for each treatment was then calculated by combining the fitted

hazard rate (exponential decay constant) of the anchor treatment with the pairwise posterior median HR calculated by the Bayesian model for the respective treatment.

Figure 6. Objective Response. Forest plots (random effects) of direct frequentist analyses of patient survival (RevMan, by Cochrane). Risk of bias assessment by the Cochrane Collaboration tool is presented as well.

Figure 7. Objective Response network meta-analysis (Random effects forest plot). Different treatments are reported in order of efficacy ranking according to the SUCRA statistic. Black circles denote the posterior median and the black lines denote the associated 95% CrI. Numbers represent odds ratios (OR) and 95% CrIs. The combination of TACE and ablation was found to be the most effective treatment (SUCRA 99%).

Figure 8. Patient survival and objective response. Two-dimensional ranking of different treatments according to patient survival (y-axis) and objective response (x-axis) based on the cumulative rank probabilities (SUCRA; %). Note the linear correlation (linear regression fit R^2=0.926) between the 2 outcome metrics.

Figure 9. Serious adverse events. Forest plots (random effects) of direct frequentist analyses of patient survival (RevMan, Cochrane). Risk of bias assessment by the Cochrane Collaboration tool is presented as well.

Figure 10. Serious Adverse Events network meta-analysis (Random effects forest plot). Different treatments are reported in order of safety ranking according to the SUCRA statistic. Black circles denote the posterior median and the black lines denote the associated 95% CrI. Numbers represent odds ratios (OR) and 95% CrIs. TARE was found to be the safest treatment (SUCRA 90%).

Figure 11. Strength and quality of evidence. QoE was graded as recommended for network meta-analyses on the basis of clinical diversity (between-trial heterogeneity of patient characteristics and/or study design), indirectness (absence of direct randomized comparisons), and imprecision (we chose a threshold of information fraction <50%). Effective sample size n for each comparison is shown along with information fraction (IF; %) in parentheses (compared to n=560 for a hypothetical well-powered randomized study to detect a survival benefit of HR=0.70 at 2 years). Color-coded representation of QoE; very low (light gray), low (yellow), moderate (green). There were no cases of high QoE observed.

Table 1. Included randomized controlled trials and baseline patient demographics and index tumour characteristics									
**Study & citation**	**Year**	**Patients (n)**	**Age (years)**	**Male Gender (%)**	**Child-Pugh A/B (#Okuda)**	**PS (0/1) or KPS**	**Median stage**	**Multinodular or diffuse**	**Follow-up (years)**
**Conventional transarterial chemoembolization (TACE) versus best supportive treatment (BST) [n=8]**									
Groupe d'Etude [62]	1995	96	64y	96%	100% / 0%	NA	NA	59%	4 years
Madden et al.[66]	1993	50	49y	92%	14% / 68%#	1 (1-3)	Okuda II	NA	5 months
Pelletier et al.[68]	1990	42	65y	88%	26% / 52%#	NA	Okuda II	NA	1 year
Pelletier et al.[67]	1998	73	66y	85%	77% / 23%	58% / 38%	Okuda I	NA	2 years
Lo et al.[64]	2002	79	63y	80%	47%/ 53%#	43% / 44%	Okuda II	60%	3.5 years
Llovet et al.[61] (3-arm)*	2002	75	65y	73%	69% / 31%	83% / 10%	BCLC B	72%	4 years
FFCD 9402 et al.[63]	2008	123	64y	87%	71% / 29%	37% / 47%	Okuda I	70%	5 years
Mabed et al.[65]	2009	100	52y	65%	69% / 31%	1 (0-2)	Okuda I	58%	1 year
**Bland transarterial embolization (TAE) versus best supportive treatment (BST) [n=3]**									
Lin et al.[111]	1988	63	50y	92%	100% (A/B)	NA	NA	NA	2 years
Bruix et al.[110]	1998	80	63y	75%	68% / 32%#	68% / 27%	Okuda I	76%	4 years
Llovet et al.[61] (3-arm)*	2002	72	65y	73%	67% / 33%	76% / 16%	Okuda II	76%	4 years
**Transarterial radioembolization (TARE) versus best supportive treatment (BST) [n=1]**									
Raoul et al.[112]	1994	27	66y	96%	52% / 48%	NA	BCLC B	70%	1 year
**Transarterial radioembolization (TARE) versus conventional transarterial chemoembolization (TACE) [n=2]**									
Raoul et al.[77]	1997	129	65y	95%	75% / 23%	KPS≥70%	Okuda I	50%	4 years
Kolligs et al.[76]	2015	28	66y	86%	64% / 25%	79% / 21%	BCLC B	68%	2 years
Salem et al.[78]	2016	45	63y	73%	56% / 44%	NA	BCLC A	47%	2 years
**Drug-eluting beads chemoembolization (DEB-TACE) versus conventional transarterial chemoembolization (TACE) [n=4]**									
Lammer et al.[73]	2009	201	67y	87%	83% / 17%	77% / 23%	BCLC B	42%	6 months
Sacco et al.[74]	2011	67	70y	67%	81% / 19%	100% / 0%	BCLC A	34%	3.5 years

Malenstein et al.[75]	2011	30	62y	83%	93% / 7%	63% / 30%	BCLC B	63%	1 month
Golfieri et al.[72]	2014	177	69y	76%	86% / 24%	74% / 26%	BCLC B	54%	2 years
**Bland transarterial embolization (TAE) versus conventional transarterial chemoembolization (TACE) [n=4]**									
Chang et al.[69]	1994	46	64y	93%	65% / 35%	NA	NA	57%	2 years
Kawai et al.[70]	1991	286	62y	85%	73% / 24%	52% / 26%	NA	NA	3 years
Meyer et al.[9]	2013	86	63y	86%	83% / 17%	67% / 20%	BCLC B	67%	3 years
Yu et al.[71]	2014	90	65y	80%	81% / 19%	66% / 31%	BCLC B	52%	4 years
**Drug-eluting beads chemoembolization (DEB-TACE) versus bland transarterial embolization (TAE) [n=2]**									
Malagari et al.[108]	2010	84	70y	77%	58%/ 42%	64% / 36%	NA	38%	1 year
Brown et al.[107]	2016	101	67y	77%	85% / 15%	86% / 14%	BCLC B	60%	6 years
**Drug-eluting beads chemoembolization (DEB-TACE) versus transarterial radioembolization (TARE) [n=1]**									
Pitton et al.[109]	2015	24	71y	75%	79% / 21%	100% / 0%	BCLC B	96%	3 years
**Conventional transarterial chemoembolization (TACE) plus systemic therapy versus conventional transarterial chemoembolization (TACE) [n=8]**									
Sansonno et al.[84]	2012	80	73y	60%	100% / 0%	61% / 39%	NA	45%	21 months
Kudo et al.[81]	2011	458	70y	75%	100% / 0%	88% / 12%	NA	27%	3 years
Britten et al.[79]	2011	30	59y	50%	93% / 7%	80% / 20%	BCLC B	27%	5 years
Pinter et al.[83]	2015	32	61y	91%	69% / 31%	100% / 0%	BCLC B	59%	46 months
Wang et al.[85]	2015	125	55y	85%	85% / 15%	82% / NA	BCLC B	33%	40 months
Li et al.[82]	2009	216	48y	70%	91% / 9%	76% / NA	Okuda I	55%	3 years
Kudo et al.[17]	2014	502	58y	84%	94% / 5%	80% / 20%	BCLC B	65%	3 years
Inaba et al.[80]	2013	101	NA	81%	84% / 16%	93% / 7%	BCLC B	57%	3 years

**Drug-eluting beads chemoembolization (DEB-TACE) plus adjuvant systemic versus Drug-eluting beads chemoembolization (DEB-TACE) [n=1]**									
Lencioni et al.[16]	2016	307	64y	85%	100% / 0%	100% / 0%	BCLC B	100%	800 days
**Conventional transarterial chemoembolization (TACE) plus tumour ablation versus conventional transarterial chemoembolization (TACE) [n=9]**									
Yang et al. [93]	2008	35	58y	74%	60% / 29%	NA	NA	66%	2 years
Bartolozzi et al.[86]	1995	53	66y	77%	47% / 53%	NA	NA	40%	3 years
Becker et al.[87]	2005	52	64y	79%	75% / 25%	NA	Okuda I	37%	30 months
Wu et al.[90]	1998	102	55y	94%	78% / 17%	NA	NA	NA	3 years
Xu et al.[91]	2002	45	NA	NA	100% / 0%	NA	NA	0%	3 years
Yamamoto et al.[92]	1997	100	NA	87%	37% / 42%	NA	JIS II-IV	52%	3 years
Liu et al.[88]	2009	78	53y	NA	86% / 14%	NA	BCLC C	NA	2 years
Wang et al.[89]	2007	83	58y	80%	80% / 20%	NA	TNM III	NA	1 year
Zhao et al.[94]	2011	47	NA	NA	NA	NA	BCLC C	NA	3 years
Huang et al.[95]	2016	120	60y	77%	100% (A/B)	NA	BCLC B	0%	5 years
**Conventional transarterial chemoembolization (TACE) plus external radiotherapy versus conventional transarterial chemoembolization (TACE) [n=11]**									
Xue et al.[103]	1995	41	NA	NA	100% (A/B)	NA	TNM II	NA	1 year
Leng et al.[96]	2000	75	NA	NA	100% / 0%	KPS≥65%	TNM III	NA	3 years
Wang et al.[101]	2000	40	37y	92%	85% (A/B)	NA	TNM III	30%	5 years
Peng et al.[99]	2000	91	NA	NA	NA	NA	TNM II	NA	5 years
Li et al.[97]	2003	82	51y	NA	61% / 39%	NA	NA	NA	3 years
Zhao et al.[105]	2006	96	53y	63%	100% / 0%	KPS≥70%	TNM I	NA	3 years
Shang et al.[100]	2007	76	52y	NA	100% (A/B)	KPS≥70%	TNM I	NA	3 years
Xiao et al.[106]	2008	60	NA	NA	65% / 35%	KPS≥70%	TNM II	NA	3 years

Liao et al.[98]	2010	48	NA	NA	71% / 29%	NA	TNM III	NA	3 years
Wang et al.[102]	2006	108	54y	NA	100% (A/B)	KPS≥65%	TNM III	8%	3 years
Zhang et al.[104]	2012	259	53y	NA	100% (A/B)	NA	BCLC C	NA	2 years

References

1. Bruix J, Sherman M, Practice Guidelines Committee AAftSoLD (2005) Management of hepatocellular carcinoma. Hepatology 42: 1208-1236.
2. de Baere T, Arai Y, Lencioni R, Geschwind JF, Rilling W, et al. (2016) Treatment of Liver Tumors with Lipiodol TACE: Technical Recommendations from Experts Opinion. Cardiovasc Intervent Radiol 39: 334-343.
3. Llovet JM, Zucman-Rossi J, Pikarsky E, Sangro B, Schwartz M, et al. (2016) Hepatocellular carcinoma. Nat Rev Dis Primers 2: 16018.
4. Bruix J, Reig M, Sherman M (2016) Evidence-Based Diagnosis, Staging, and Treatment of Patients With Hepatocellular Carcinoma. Gastroenterology 150: 835-853.
5. Mathurin P, Rixe O, Carbonell N, Bernard B, Cluzel P, et al. (1998) Review article: Overview of medical treatments in unresectable hepatocellular carcinoma--an impossible meta-analysis? Aliment Pharmacol Ther 12: 111-126.
6. Delicque J, Boulin M, Guiu B, Pelage JP, Escal L, et al. (2016) Interventional oncology for hepatocellular carcinoma. Clin Res Hepatol Gastroenterol.
7. Jansen MC, van Hillegersberg R, Chamuleau RA, van Delden OM, Gouma DJ, et al. (2005) Outcome of regional and local ablative therapies for hepatocellular carcinoma: a collective review. Eur J Surg Oncol 31: 331-347.
8. Lencioni R, de Baere T, Soulen MC, Rilling WS, Geschwind JH (2016) Lipiodol transarterial chemoembolization for hepatocellular carcinoma: A systematic review of efficacy and safety data. Hepatology.
9. Meyer T, Kirkwood A, Roughton M, Beare S, Tsochatzis E, et al. (2013) A randomised phase II/III trial of 3-weekly cisplatin-based sequential

transarterial chemoembolisation vs embolisation alone for hepatocellular carcinoma. Br J Cancer 108: 1252-1259.

10. Li L, Tian J, Liu P, Wang X, Zhu Z (2016) Transarterial chemoembolization combination therapy vs monotherapy in unresectable hepatocellular carcinoma: a meta-analysis. Tumori: 0.
11. Ray CE, Jr., Brown AC, Green TJ, Winston H, Curran C, et al. (2015) Survival outcomes in patients with advanced hepatocellular carcinoma treated with drug-eluting bead chemoembolization. AJR Am J Roentgenol 204: 440-447.
12. Fu Y, Zhao X, Yun Q, Zhu X, Zhu Y, et al. (2015) Transarterial chemoembolization (TACE) plus percutaneous ethanol injection (PEI) for the treatment of unresectable hepatocellular carcinoma: a meta-analysis of randomized controlled trials. Int J Clin Exp Med 8: 10388-10400.
13. Facciorusso A, Di Maso M, Muscatiello N (2016) Microwave ablation versus radiofrequency ablation for the treatment of hepatocellular carcinoma: A systematic review and meta-analysis. Int J Hyperthermia: 1-6.
14. Kallini JR, Gabr A, Salem R, Lewandowski RJ (2016) Transarterial Radioembolization with Yttrium-90 for the Treatment of Hepatocellular Carcinoma. Adv Ther.
15. Vente MA, Wondergem M, van der Tweel I, van den Bosch MA, Zonnenberg BA, et al. (2009) Yttrium-90 microsphere radioembolization for the treatment of liver malignancies: a structured meta-analysis. Eur Radiol 19: 951-959.
16. Lencioni R, Llovet JM, Han G, Tak WY, Yang J, et al. (2016) Sorafenib or placebo plus TACE with doxorubicin-eluting beads for intermediate stage HCC: The SPACE trial. J Hepatol 64: 1090-1098.

17. Kudo M, Han G, Finn RS, Poon RT, Blanc JF, et al. (2014) Brivanib as adjuvant therapy to transarterial chemoembolization in patients with hepatocellular carcinoma: A randomized phase III trial. Hepatology 60: 1697-1707.

18. Huo YR, Eslick GD (2015) Transcatheter Arterial Chemoembolization Plus Radiotherapy Compared With Chemoembolization Alone for Hepatocellular Carcinoma: A Systematic Review and Meta-analysis. JAMA Oncol 1: 756-765.

19. Facciorusso A, Di Maso M, Muscatiello N (2016) Drug-eluting beads versus conventional chemoembolization for the treatment of unresectable hepatocellular carcinoma: A meta-analysis. Dig Liver Dis.

20. Katsanos K, Spiliopoulos S, Karunanithy N, Krokidis M, Sabharwal T, et al. (2014) Bayesian network meta-analysis of nitinol stents, covered stents, drug-eluting stents, and drug-coated balloons in the femoropopliteal artery. J Vasc Surg 59: 1123-1133 e1128.

21. Katsanos K, Spiliopoulos S, Saha P, Diamantopoulos A, Karunanithy N, et al. (2015) Comparative Efficacy and Safety of Different Antiplatelet Agents for Prevention of Major Cardiovascular Events and Leg Amputations in Patients with Peripheral Arterial Disease: A Systematic Review and Network Meta-Analysis. PLoS One 10: e0135692.

22. Ni JY, Liu SS, Xu LF, Sun HL, Chen YT (2013) Transarterial chemoembolization combined with percutaneous radiofrequency ablation versus TACE and PRFA monotherapy in the treatment for hepatocellular carcinoma: a meta-analysis. J Cancer Res Clin Oncol 139: 653-659.

23. Bhardwaj N, Strickland AD, Ahmad F, Dennison AR, Lloyd DM (2010) Liver ablation techniques: a review. Surg Endosc 24: 254-265.

24. Xie ZB, Wang XB, Peng YC, Zhu SL, Ma L, et al. (2015) Systematic review comparing the safety and efficacy of conventional and drug-eluting bead transarterial chemoembolization for inoperable hepatocellular carcinoma. Hepatol Res 45: 190-200.

25. Ni JY, Xu LF, Wang WD, Sun HL, Chen YT (2014) Conventional transarterial chemoembolization vs microsphere embolization in hepatocellular carcinoma: a meta-analysis. World J Gastroenterol 20: 17206-17217.

26. Han S, Zhang X, Zou L, Lu C, Zhang J, et al. (2014) Does drug-eluting bead transcatheter arterial chemoembolization improve the management of patients with hepatocellular carcinoma? A meta-analysis. PLoS One 9: e102686.

27. Camma C, Schepis F, Orlando A, Albanese M, Shahied L, et al. (2002) Transarterial chemoembolization for unresectable hepatocellular carcinoma: meta-analysis of randomized controlled trials. Radiology 224: 47-54.

28. Liao M, Huang J, Zhang T, Wu H (2013) Transarterial chemoembolization in combination with local therapies for hepatocellular carcinoma: a meta-analysis. PLoS One 8: e68453.

29. Liu L, Chen H, Wang M, Zhao Y, Cai G, et al. (2014) Combination therapy of sorafenib and TACE for unresectable HCC: a systematic review and meta-analysis. PLoS One 9: e91124.

30. Meng MB, Cui YL, Lu Y, She B, Chen Y, et al. (2009) Transcatheter arterial chemoembolization in combination with radiotherapy for unresectable hepatocellular carcinoma: a systematic review and meta-analysis. Radiother Oncol 92: 184-194.

31. Yang M, Yuan JQ, Bai M, Han GH (2014) Transarterial chemoembolization combined with sorafenib for unresectable hepatocellular carcinoma: a systematic review and meta-analysis. Mol Biol Rep 41: 6575-6582.

32. Zou LQ, Zhang BL, Chang Q, Zhu FP, Li YY, et al. (2014) 3D conformal radiotherapy combined with transcatheter arterial chemoembolization for hepatocellular carcinoma. World J Gastroenterol 20: 17227-17234.

33. Oliveri RS, Wetterslev J, Gluud C (2011) Transarterial (chemo)embolisation for unresectable hepatocellular carcinoma. Cochrane Database Syst Rev: CD004787.

34. Moher D, Liberati A, Tetzlaff J, Altman DG, Group P (2009) Preferred reporting items for systematic reviews and meta-analyses: the PRISMA statement. BMJ 339: b2535.

35. Gaba RC, Lewandowski RJ, Hickey R, Baerlocher MO, Cohen EI, et al. (2016) Transcatheter Therapy for Hepatic Malignancy: Standardization of Terminology and Reporting Criteria. J Vasc Interv Radiol 27: 457-473.

36. Higgins JP, Altman DG, Gotzsche PC, Juni P, Moher D, et al. (2011) The Cochrane Collaboration's tool for assessing risk of bias in randomised trials. BMJ 343: d5928.

37. Jansen JP, Trikalinos T, Cappelleri JC, Daw J, Andes S, et al. (2014) Indirect treatment comparison/network meta-analysis study questionnaire to assess relevance and credibility to inform health care decision making: an ISPOR-AMCP-NPC Good Practice Task Force report. Value Health 17: 157-173.

38. Guyatt GH, Oxman AD, Vist GE, Kunz R, Falck-Ytter Y, et al. (2008) GRADE: an emerging consensus on rating quality of evidence and strength of recommendations. BMJ 336: 924-926.

39. Puhan MA, Schunemann HJ, Murad MH, Li T, Brignardello-Petersen R, et al. (2014) A GRADE Working Group approach for rating the quality of treatment effect estimates from network meta-analysis. BMJ 349: g5630.

40. Woods BS, Hawkins N, Scott DA (2010) Network meta-analysis on the log-hazard scale, combining count and hazard ratio statistics accounting for multi-arm trials: a tutorial. BMC Med Res Methodol 10: 54.

41. Hirooka T, Hamada C, Yoshimura I (2009) A note on estimating treatment effect for time-to-event data in a literature-based meta-analysis. Methods Inf Med 48: 104-112.

42. Parmar MK, Torri V, Stewart L (1998) Extracting summary statistics to perform meta-analyses of the published literature for survival endpoints. Stat Med 17: 2815-2834.

43. Tierney JF, Stewart LA, Ghersi D, Burdett S, Sydes MR (2007) Practical methods for incorporating summary time-to-event data into meta-analysis. Trials 8: 16.

44. (1979) World Health Organization. WHO handbook for reporting results of cancer treatment. Available at: http://appswhoint/iris/bitstream/10665/37200/1/WHO_OFFSET_48pdf Accessed 13 May 2016.

45. Bruix J, Sherman M, Llovet JM, Beaugrand M, Lencioni R, et al. (2001) Clinical management of hepatocellular carcinoma. Conclusions of the Barcelona-2000 EASL conference. European Association for the Study of the Liver. J Hepatol 35: 421-430.

46. Eisenhauer EA, Therasse P, Bogaerts J, Schwartz LH, Sargent D, et al. (2009) New response evaluation criteria in solid tumours: revised RECIST guideline (version 1.1). Eur J Cancer 45: 228-247.

47. Lencioni R, Llovet JM (2010) Modified RECIST (mRECIST) assessment for hepatocellular carcinoma. Semin Liver Dis 30: 52-60.

48. (2010) US Department of Health and Human Services; National Institutes of Health; National Cancer Institute. Common terminology criteria for adverse events (CTCAE). Version 4.0. Available at: http://evsncinihgov/ftp1/CTCAE/CTCAE_403_2010-06-14_QuickReference_85x11pdf Accessed 13 May 2016.

49. Dias S, Welton NJ, Sutton AJ, Caldwell DM, Lu G, et al. (2013) Evidence synthesis for decision making 4: inconsistency in networks of evidence based on randomized controlled trials. Med Decis Making 33: 641-656.

50. Dias S, Sutton AJ, Welton NJ, Ades AE (2013) Evidence synthesis for decision making 3: heterogeneity--subgroups, meta-regression, bias, and bias-adjustment. Med Decis Making 33: 618-640.

51. Dias S, Sutton AJ, Ades AE, Welton NJ (2013) Evidence synthesis for decision making 2: a generalized linear modeling framework for pairwise and network meta-analysis of randomized controlled trials. Med Decis Making 33: 607-617.

52. Salanti G, Ades AE, Ioannidis JP (2011) Graphical methods and numerical summaries for presenting results from multiple-treatment meta-analysis: an overview and tutorial. J Clin Epidemiol 64: 163-171.

53. Wetterslev J, Thorlund K, Brok J, Gluud C (2009) Estimating required information size by quantifying diversity in random-effects model meta-analyses. BMC Med Res Methodol 9: 86.

54. Brok J, Thorlund K, Gluud C, Wetterslev J (2008) Trial sequential analysis reveals insufficient information size and potentially false positive results in many meta-analyses. J Clin Epidemiol 61: 763-769.

55. Thorlund K, Mills EJ (2012) Sample size and power considerations in network meta-analysis. Syst Rev 1: 41.

56. Chaimani A, Higgins JP, Mavridis D, Spyridonos P, Salanti G (2013) Graphical tools for network meta-analysis in STATA. PLoS One 8: e76654.

57. Achana FA, Cooper NJ, Dias S, Lu G, Rice SJ, et al. (2013) Extending methods for investigating the relationship between treatment effect and baseline risk from pairwise meta-analysis to network meta-analysis. Stat Med 32: 752-771.

58. Jansen JP, Naci H (2013) Is network meta-analysis as valid as standard pairwise meta-analysis? It all depends on the distribution of effect modifiers. BMC Med 11: 159.

59. Dias S, Welton NJ, Caldwell DM, Ades AE (2010) Checking consistency in mixed treatment comparison meta-analysis. Stat Med 29: 932-944.

60. Brown S, Hutton B, Clifford T, Coyle D, Grima D, et al. (2014) A Microsoft-Excel-based tool for running and critically appraising network meta-analyses--an overview and application of NetMetaXL. Syst Rev 3: 110.

61. Llovet JM, Real MI, Montana X, Planas R, Coll S, et al. (2002) Arterial embolisation or chemoembolisation versus symptomatic treatment in patients with unresectable hepatocellular carcinoma: a randomised controlled trial. Lancet 359: 1734-1739.

62. (1995) A comparison of lipiodol chemoembolization and conservative treatment for unresectable hepatocellular carcinoma. Groupe d'Etude et de Traitement du Carcinome Hepatocellulaire. N Engl J Med 332: 1256-1261.

63. Doffoel M, Bonnetain F, Bouche O, Vetter D, Abergel A, et al. (2008) Multicentre randomised phase III trial comparing Tamoxifen alone or with Transarterial Lipiodol Chemoembolisation for unresectable hepatocellular carcinoma in cirrhotic patients (Federation Francophone de Cancerologie Digestive 9402). Eur J Cancer 44: 528-538.

64. Lo CM, Ngan H, Tso WK, Liu CL, Lam CM, et al. (2002) Randomized controlled trial of transarterial lipiodol chemoembolization for unresectable hepatocellular carcinoma. Hepatology 35: 1164-1171.

65. Mabed M, Esmaeel M, El-Khodary T, Awad M, Amer T (2009) A randomized controlled trial of transcatheter arterial chemoembolization with lipiodol, doxorubicin and cisplatin versus intravenous doxorubicin for patients with unresectable hepatocellular carcinoma. Eur J Cancer Care (Engl) 18: 492-499.

66. Madden MV, Krige JE, Bailey S, Beningfield SJ, Geddes C, et al. (1993) Randomised trial of targeted chemotherapy with lipiodol and 5-epidoxorubicin compared with symptomatic treatment for hepatoma. Gut 34: 1598-1600.

67. Pelletier G, Ducreux M, Gay F, Luboinski M, Hagege H, et al. (1998) Treatment of unresectable hepatocellular carcinoma with lipiodol chemoembolization: a multicenter randomized trial. Groupe CHC. J Hepatol 29: 129-134.

68. Pelletier G, Roche A, Ink O, Anciaux ML, Derhy S, et al. (1990) A randomized trial of hepatic arterial chemoembolization in patients with unresectable hepatocellular carcinoma. J Hepatol 11: 181-184.

69. Chang JM, Tzeng WS, Pan HB, Yang CF, Lai KH (1994) Transcatheter arterial embolization with or without cisplatin treatment of hepatocellular carcinoma. A randomized controlled study. Cancer 74: 2449-2453.

70. Kawai S, Okamura J, Ogawa M, Ohashi Y, Tani M, et al. (1992) Prospective and randomized clinical trial for the treatment of hepatocellular carcinoma--a comparison of lipiodol-transcatheter arterial embolization with and without adriamycin (first cooperative study). The Cooperative Study Group for Liver Cancer Treatment of Japan. Cancer Chemother Pharmacol 31 Suppl: S1-6.

71. Yu SC, Hui JW, Hui EP, Chan SL, Lee KF, et al. (2014) Unresectable hepatocellular carcinoma: randomized controlled trial of transarterial ethanol ablation versus transcatheter arterial chemoembolization. Radiology 270: 607-620.

72. Golfieri R, Giampalma E, Renzulli M, Cioni R, Bargellini I, et al. (2014) Randomised controlled trial of doxorubicin-eluting beads vs conventional chemoembolisation for hepatocellular carcinoma. Br J Cancer 111: 255-264.

73. Lammer J, Malagari K, Vogl T, Pilleul F, Denys A, et al. (2010) Prospective randomized study of doxorubicin-eluting-bead embolization in the treatment of hepatocellular carcinoma: results of the PRECISION V study. Cardiovasc Intervent Radiol 33: 41-52.

74. Sacco R, Bargellini I, Bertini M, Bozzi E, Romano A, et al. (2011) Conventional versus doxorubicin-eluting bead transarterial chemoembolization for hepatocellular carcinoma. J Vasc Interv Radiol 22: 1545-1552.

75. van Malenstein H, Maleux G, Vandecaveye V, Heye S, Laleman W, et al. (2011) A randomized phase II study of drug-eluting beads versus transarterial chemoembolization for unresectable hepatocellular carcinoma. Onkologie 34: 368-376.

76. Kolligs FT, Bilbao JI, Jakobs T, Inarrairaegui M, Nagel JM, et al. (2015) Pilot randomized trial of selective internal radiation therapy vs. chemoembolization in unresectable hepatocellular carcinoma. Liver Int 35: 1715-1721.

77. Raoul JL, Guyader D, Bretagne JF, Heautot JF, Duvauferrier R, et al. (1997) Prospective randomized trial of chemoembolization versus intra-arterial injection of 131I-labeled-iodized oil in the treatment of hepatocellular carcinoma. Hepatology 26: 1156-1161.

78. Salem R, Gordon AC, Mouli S, Hickey R, Kallini J, et al. (2016) Y90 Radioembolization Significantly Prolongs Time to Progression Compared With Chemoembolization in Patients With Hepatocellular Carcinoma. Gastroenterology 151: 1155-1163 e1152.

79. Britten CD, Gomes AS, Wainberg ZA, Elashoff D, Amado R, et al. (2012) Transarterial chemoembolization plus or minus intravenous bevacizumab in the treatment of hepatocellular cancer: a pilot study. BMC Cancer 12: 16.

80. Inaba Y, Kanai F, Aramaki T, Yamamoto T, Tanaka T, et al. (2013) A randomised phase II study of TSU-68 in patients with hepatocellular carcinoma treated by transarterial chemoembolisation. Eur J Cancer 49: 2832-2840.

81. Kudo M, Imanaka K, Chida N, Nakachi K, Tak WY, et al. (2011) Phase III study of sorafenib after transarterial chemoembolisation in Japanese and Korean patients with unresectable hepatocellular carcinoma. Eur J Cancer 47: 2117-2127.

82. Li M, Lu C, Cheng J, Zhang J, Cao C, et al. (2009) Combination therapy with transarterial chemoembolization and interferon-alpha compared with transarterial chemoembolization alone for hepatitis B virus related

unresectable hepatocellular carcinoma. J Gastroenterol Hepatol 24: 1437-1444.

83. Pinter M, Ulbrich G, Sieghart W, Kolblinger C, Reiberger T, et al. (2015) Hepatocellular Carcinoma: A Phase II Randomized Controlled Double-Blind Trial of Transarterial Chemoembolization in Combination with Biweekly Intravenous Administration of Bevacizumab or a Placebo. Radiology 277: 903-912.

84. Sansonno D, Lauletta G, Russi S, Conteduca V, Sansonno L, et al. (2012) Transarterial chemoembolization plus sorafenib: a sequential therapeutic scheme for HCV-related intermediate-stage hepatocellular carcinoma: a randomized clinical trial. Oncologist 17: 359-366.

85. Wang H, Liu Y, Wang X, Liu D, Sun Z, et al. (2015) Randomized clinical control study of locoregional therapy combined with arsenic trioxide for the treatment of hepatocellular carcinoma. Cancer 121: 2917-2925.

86. Bartolozzi C, Lencioni R, Caramella D, Vignali C, Cioni R, et al. (1995) Treatment of large HCC: transcatheter arterial chemoembolization combined with percutaneous ethanol injection versus repeated transcatheter arterial chemoembolization. Radiology 197: 812-818.

87. Becker G, Soezgen T, Olschewski M, Laubenberger J, Blum HE, et al. (2005) Combined TACE and PEI for palliative treatment of unresectable hepatocellular carcinoma. World J Gastroenterol 11: 6104-6109.

88. Liu DX, Li HD, Li XF, al. e (2009) Evaluation of TACE combined with RFA and PEI in treating advanced hepatic carcinoma. J Intervent Radiol 18: 389-393.

89. Wang YB, Chen MH, Yan K, Yang W, Dai Y, et al. (2007) Quality of life after radiofrequency ablation combined with transcatheter arterial

chemoembolization for hepatocellular carcinoma: comparison with transcatheter arterial chemoembolization alone. Qual Life Res 16: 389-397.

90. Wu PH, Li L, Zhang YM (1998) Transcatheter arterial chemoembolization combined with CT-guided percutaneous intratumoral injection of lipiodol-ethanol for the treatment of hepatocellular carcinoma. Chin J Oncol 20: 391-393.
91. Xu GH, Wen HC, Li ZW, al. e (2002) Evaluation of hepatic chemoembolization and percutaneous ethanol injection in the treatment of HCC. Chin J Radiology 1: 66-68.
92. Yamamoto K, Masuzawa M, Kato M, Kurosawa K, Kaneko A, et al. (1997) Evaluation of combined therapy with chemoembolization and ethanol injection for advanced hepatocellular carcinoma. Semin Oncol 24: S6-50-S56-55.
93. Yang P, Liang M, Zhang Y, Shen B (2008) Clinical application of a combination therapy of lentinan, multi-electrode RFA and TACE in HCC. Adv Ther 25: 787-794.
94. Zhao M, Wang JP, Li W, Huang ZL, Zhang FJ, et al. (2011) [Comparison of safety and efficacy for transcatheter arterial chemoembolization alone and plus radiofrequency ablation in the treatment of single branch portal vein tumor thrombus of hepatocellular carcinoma and their prognosis factors]. Zhonghua Yi Xue Za Zhi 91: 1167-1172.
95. Huang C, Zhuang W, Feng H, Guo H, Tang Y, et al. (2016) Analysis of therapeutic effectiveness and prognostic factor on argon-helium cryoablation combined with transcatheter arterial chemoembolization for the treatment of advanced hepatocellular carcinoma. J Cancer Res Ther 12: C148-C152.

96. Leng ZQ, Liang ZY, Shi S, Hu ZX (2000) Comparison of treatment results of interventional therapy alone, radiotherapy alone, and combined interventional therapy plus radiotherapy for primary hepatic cancer. Chin J Radiat Oncol 9: 99-101.

97. Li Y, Yan Y, Zhang HB, Guo ZW, Yan ZC, et al. (2003) Three-dimensional conformal radiation combined with transarterial chemoembolization for unresectable primary liver cancer. Chin J Radiat Oncol 12: 30-32.

98. Liao XF, He HJ, Zhou ZS, Hu W, Zhu XP (2010) Three-dimensional conformal radiotherapy combined with interventional therapy in treatment of primary hepatocellular carcinoma. J Prac Oncol 25: 681-683.

99. Peng KG, Han FS, Liu H, Song MZ (2000) Clinical study of unresectable liver cancer treated by intraoperative hepatic arterial embolization and post-operative hyperfractionation radiotherapy. Chin J Radiat Oncol 9: 11-13.

100. Shang Y, You GX, Xu HY, Chen MC (2007) Prospective randomized clinical study of transcatheter arterial chemoembolization, combined with three-dimensional conformal radiotherapy for primary liver cancer: An analysis of 40 cases. Shijie Huaren Xiaohua Zazhi 15: 3140-3142.

101. Wang G, Shen W, Song M, Xu H (2000) Results of combined treatment with transcatheter hepatic arterial chemoembolization and whole-liver irradiation with the moving strip technique in unresectable hepatocellular carcinoma. . Int J Clin Oncol 5: 380-385.

102. Wang XH, Li JX, Gao K (2006) Radiotherapy combined with hepatic cheoembolization in the treatment of 54 primary liver cancer. Shanxi Medical Journal 35: 461-462.

103. Xue HZ, Meng GD, Wang YW, Jiang QF (1995) Transcatheter arterial chemoembolization plus radiotherapy in the treatment of hepatocellular carcinoma. Chin J Radiat Oncol 4: 84-85.

104. Zhang Z, Yang X, Wena M, Wan J (2012) Evaluation of TACE combined with gamma—knife radiotherapy for primary hepatocellular carcinoma. J Intervent Radiol (China) 7: 596-599.

105. Zhao MH, Lang FP, Jiang QA, Ma JJ, Song YX (2006) Three-dimensional conformal radiotherapy combined with transcatheter arterial chemoembolization for inoperable primary liver cancer. Chin J Radiat Oncol 15: 39-41.

106. Xiao Z, Ouyang T, Yu R, Jiang X, Reng H, et al. (2008) Transcatheter arterial chemoembolization combined with 3-dimensional conformal radiotherapy for patients with unresectable primary hepatic carcinoma. Chinese Journal of Clinical Oncology 35: 18-21.

107. Brown KT, Do RK, Gonen M, Covey AM, Getrajdman GI, et al. (2016) Randomized Trial of Hepatic Artery Embolization for Hepatocellular Carcinoma Using Doxorubicin-Eluting Microspheres Compared With Embolization With Microspheres Alone. J Clin Oncol.

108. Malagari K, Pomoni M, Kelekis A, Pomoni A, Dourakis S, et al. (2010) Prospective randomized comparison of chemoembolization with doxorubicin-eluting beads and bland embolization with BeadBlock for hepatocellular carcinoma. Cardiovasc Intervent Radiol 33: 541-551.

109. Pitton MB, Kloeckner R, Ruckes C, Wirth GM, Eichhorn W, et al. (2015) Randomized comparison of selective internal radiotherapy (SIRT) versus drug-eluting bead transarterial chemoembolization (DEB-TACE) for the

treatment of hepatocellular carcinoma. Cardiovasc Intervent Radiol 38: 352-360.

110. Bruix J, Llovet JM, Castells A, Montana X, Bru C, et al. (1998) Transarterial embolization versus symptomatic treatment in patients with advanced hepatocellular carcinoma: results of a randomized, controlled trial in a single institution. Hepatology 27: 1578-1583.

111. Lin DY, Liaw YF, Lee TY, Lai CM (1988) Hepatic arterial embolization in patients with unresectable hepatocellular carcinoma--a randomized controlled trial. Gastroenterology 94: 453-456.

112. Raoul JL, Guyader D, Bretagne JF, Duvauferrier R, Bourguet P, et al. (1994) Randomized controlled trial for hepatocellular carcinoma with portal vein thrombosis: intra-arterial iodine-131-iodized oil versus medical support. J Nucl Med 35: 1782-1787.

113. Puhan MA, Schunemann HJ, Murad MH, Li T, Brignardello-Petersen R, et al. (2014) A GRADE Working Group approach for rating the quality of treatment effect estimates from network meta-analysis. BMJ 349: g5630.

114. Lan T, Chang L, Mn R, Wu L, Yuan YF (2016) Comparative Efficacy of Interventional Therapies for Early-stage Hepatocellular Carcinoma: A PRISMA-compliant Systematic Review and Network Meta-analysis. Medicine (Baltimore) 95: e3185.

115. Marrero JA, Kudo M, Venook AP, Ye SL, Bronowicki JP, et al. (2016) Observational registry of sorafenib use in clinical practice across Child-Pugh subgroups: The GIDEON study. J Hepatol 65: 1140-1147.

116. Verslype C, Rosmorduc O, Rougier P, Group EGW (2012) Hepatocellular carcinoma: ESMO-ESDO Clinical Practice Guidelines for diagnosis, treatment and follow-up. Ann Oncol 23 Suppl 7: vii41-48.

117. Boily G, Villeneuve JP, Lacoursiere L, Chaudhury P, Couture F, et al. (2015) Transarterial embolization therapies for the treatment of hepatocellular carcinoma: CEPO review and clinical recommendations. HPB (Oxford) 17: 52-65.

118. Forner A, Llovet JM, Bruix J (2012) Chemoembolization for intermediate HCC: is there proof of survival benefit? J Hepatol 56: 984-986.

119. Park YN, Yang CP, Fernandez GJ, Cubukcu O, Thung SN, et al. (1998) Neoangiogenesis and sinusoidal "capillarization" in dysplastic nodules of the liver. Am J Surg Pathol 22: 656-662.

120. Rammohan A, Sathyanesan J, Ramaswami S, Lakshmanan A, Senthil-Kumar P, et al. (2012) Embolization of liver tumors: Past, present and future. World J Radiol 4: 405-412.

121. Wen L, Liang C, Chen E, Chen W, Liang F, et al. (2016) Regulation of Multi-drug Resistance in hepatocellular carcinoma cells is TRPC6/Calcium Dependent. Sci Rep 6: 23269.

122. Higuchi T, Kikuchi M, Okazaki M (1994) Hepatocellular carcinoma after transcatheter hepatic arterial embolization. A histopathologic study of 84 resected cases. Cancer 73: 2259-2267.

123. Kwan SW, Fidelman N, Ma E, Kerlan RK, Jr., Yao FY (2012) Imaging predictors of the response to transarterial chemoembolization in patients with hepatocellular carcinoma: a radiological-pathological correlation. Liver Transpl 18: 727-736.

124. Qi X, Zhao Y, Li H, Guo X, Han G (2016) Management of hepatocellular carcinoma: an overview of major findings from meta-analyses. Oncotarget 7: 34703-34751.

125. Llovet JM, Bruix J (2003) Systematic review of randomized trials for unresectable hepatocellular carcinoma: Chemoembolization improves survival. Hepatology 37: 429-442.

126. Marelli L, Stigliano R, Triantos C, Senzolo M, Cholongitas E, et al. (2007) Transarterial therapy for hepatocellular carcinoma: which technique is more effective? A systematic review of cohort and randomized studies. Cardiovasc Intervent Radiol 30: 6-25.

127. Facciorusso A, Bellanti F, Villani R, Salvatore V, Muscatiello N, et al. (2017) Transarterial chemoembolization vs bland embolization in hepatocellular carcinoma: A meta-analysis of randomized trials. United European Gastroenterol J 5: 511-518.

128. Gao S, Yang Z, Zheng Z, Yao J, Deng M, et al. (2013) Doxorubicin-eluting bead versus conventional TACE for unresectable hepatocellular carcinoma: a meta-analysis. Hepatogastroenterology 60: 813-820.

129. Huang K, Zhou Q, Wang R, Cheng D, Ma Y (2014) Doxorubicin-eluting beads versus conventional transarterial chemoembolization for the treatment of hepatocellular carcinoma. J Gastroenterol Hepatol 29: 920-925.

130. Zhang L, Hu P, Chen X, Bie P (2014) Transarterial chemoembolization (TACE) plus sorafenib versus TACE for intermediate or advanced stage hepatocellular carcinoma: a meta-analysis. PLoS One 9: e100305.

131. Cabibbo G, Enea M, Attanasio M, Bruix J, Craxi A, et al. (2010) A meta-analysis of survival rates of untreated patients in randomized clinical trials of hepatocellular carcinoma. Hepatology 51: 1274-1283.

132. Salem R, Mazzaferro V, Sangro B (2013) Yttrium 90 radioembolization for the treatment of hepatocellular carcinoma: biological lessons, current challenges, and clinical perspectives. Hepatology 58: 2188-2197.

 Click here to download Figure Fig1.TIF

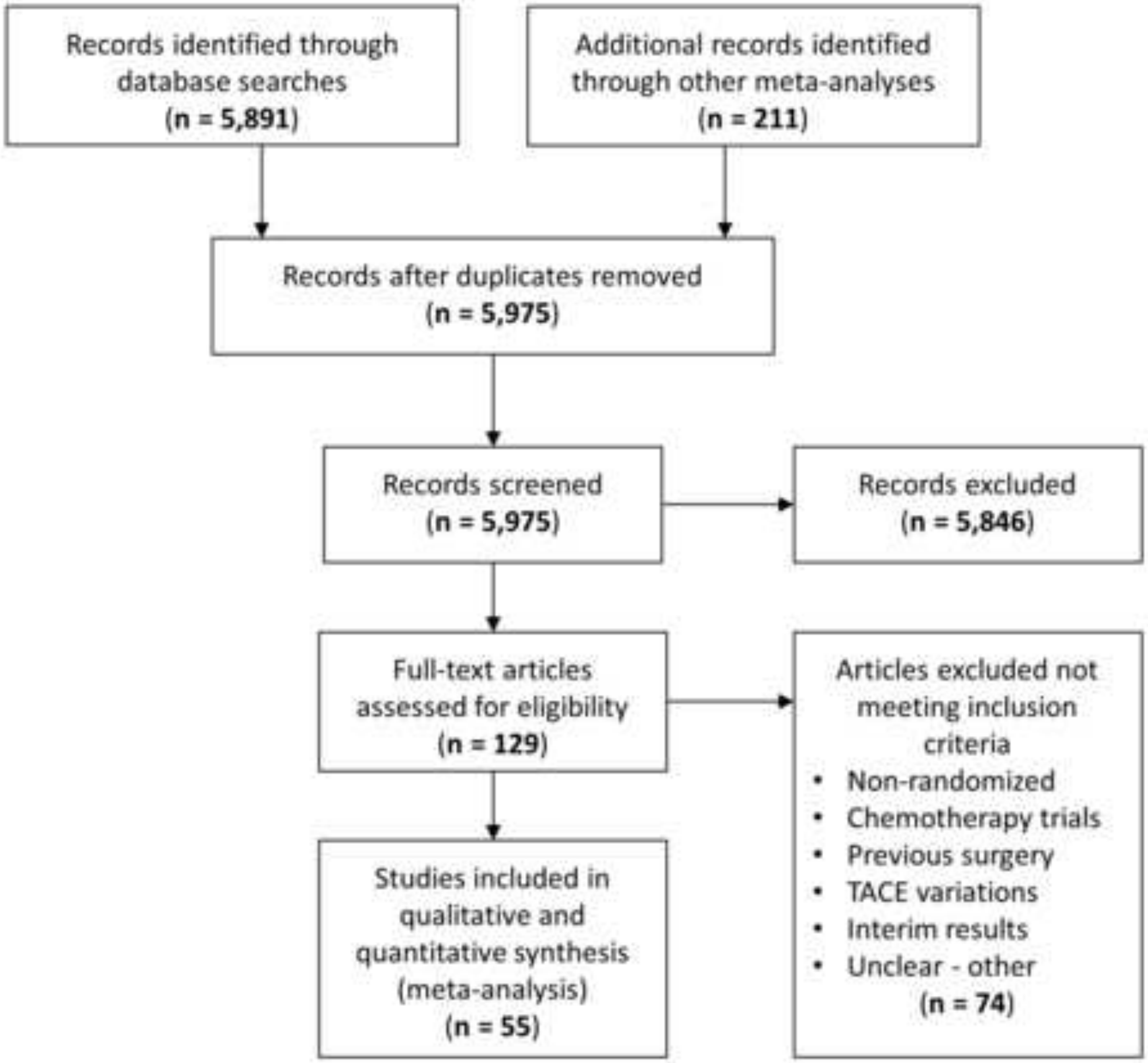

Figure 2

Click here to download Figure Fig2.TIF

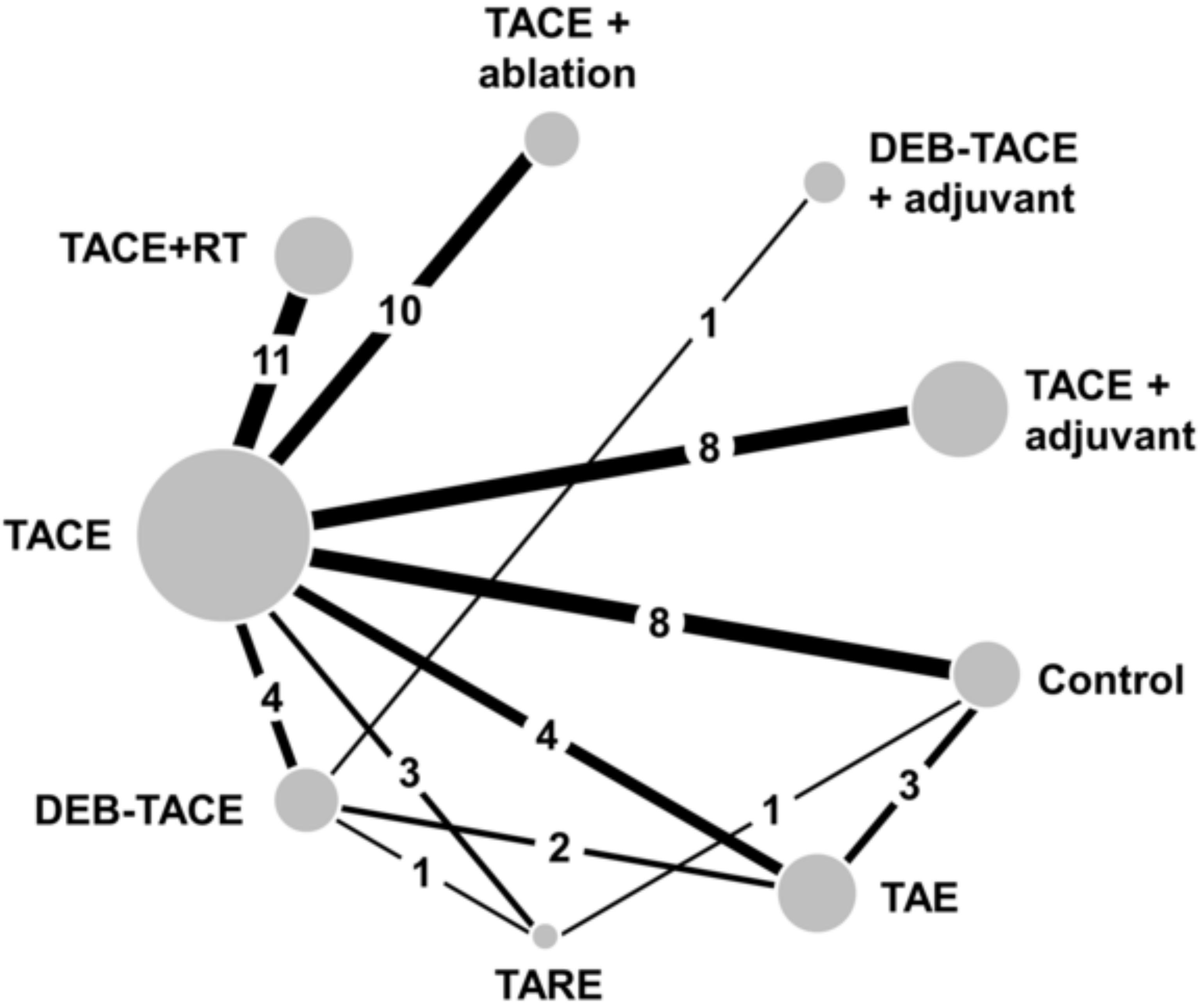

Figure 3

Click here to download Figure Fig3.TIF

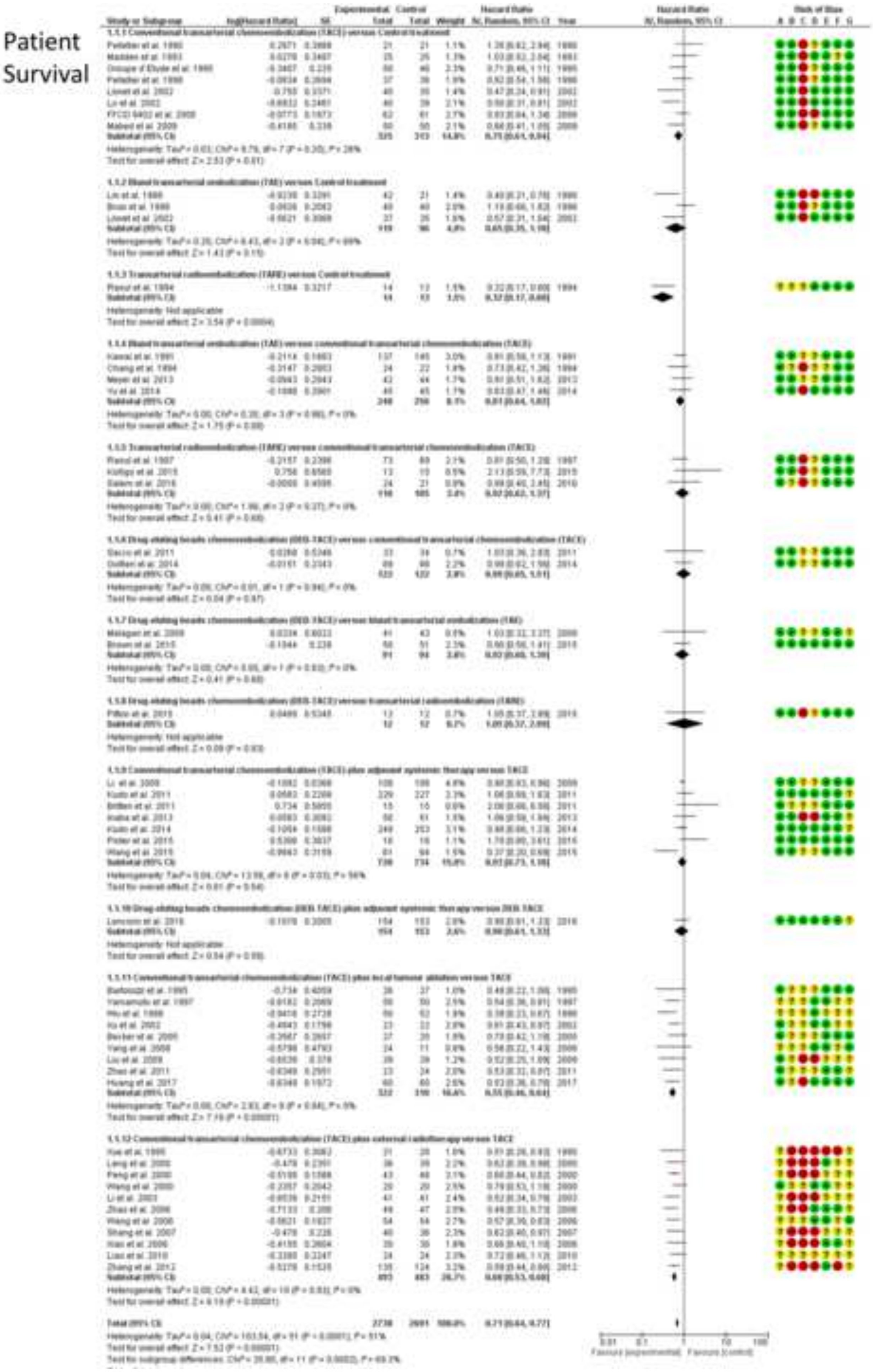

Figure 4

Click here to download Figure Fig4.TIF

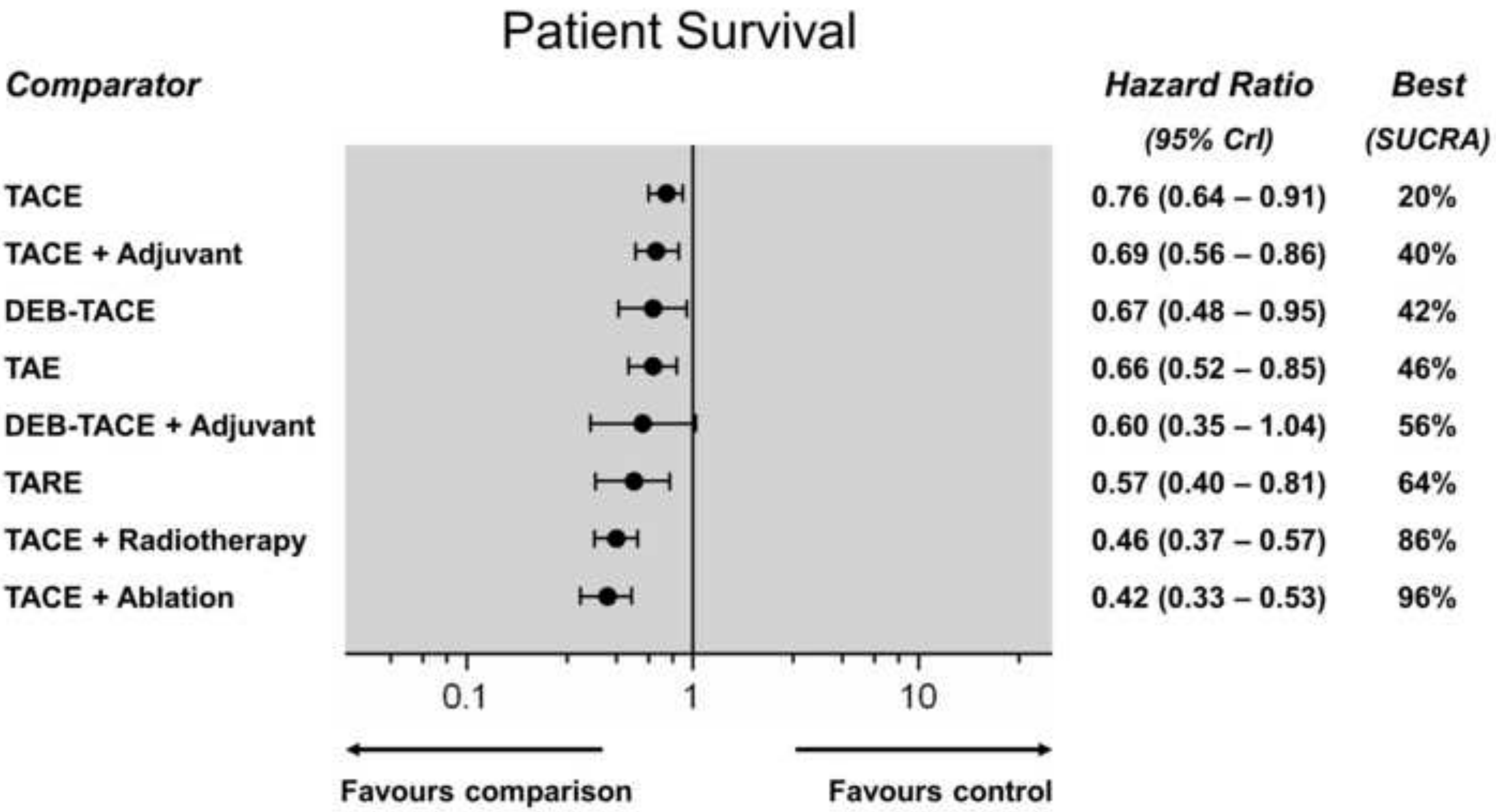

Figure 5

Click here to download Figure Fig5.TIF

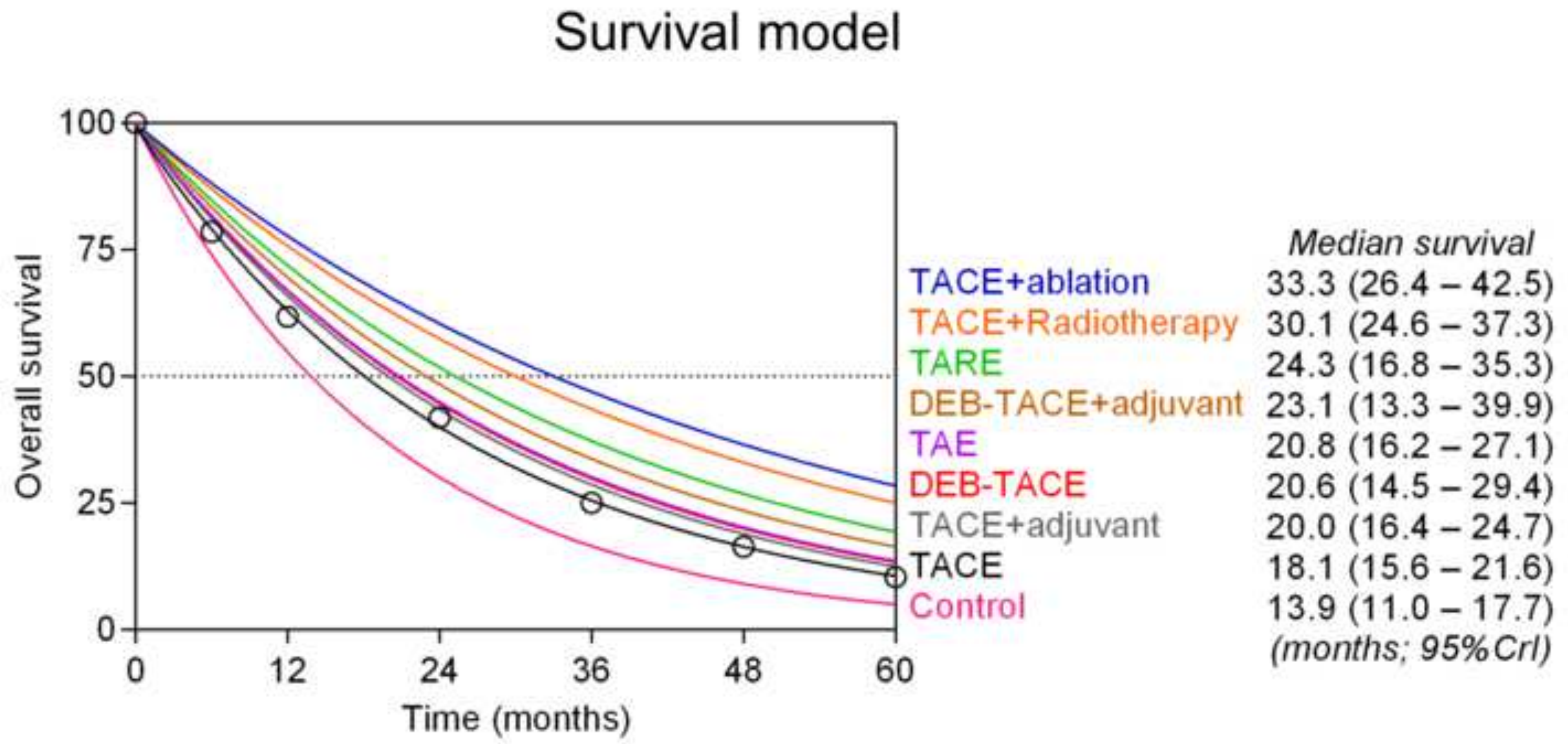

Figure 6

Click here to download Figure Fig6.TIF

Objective Response

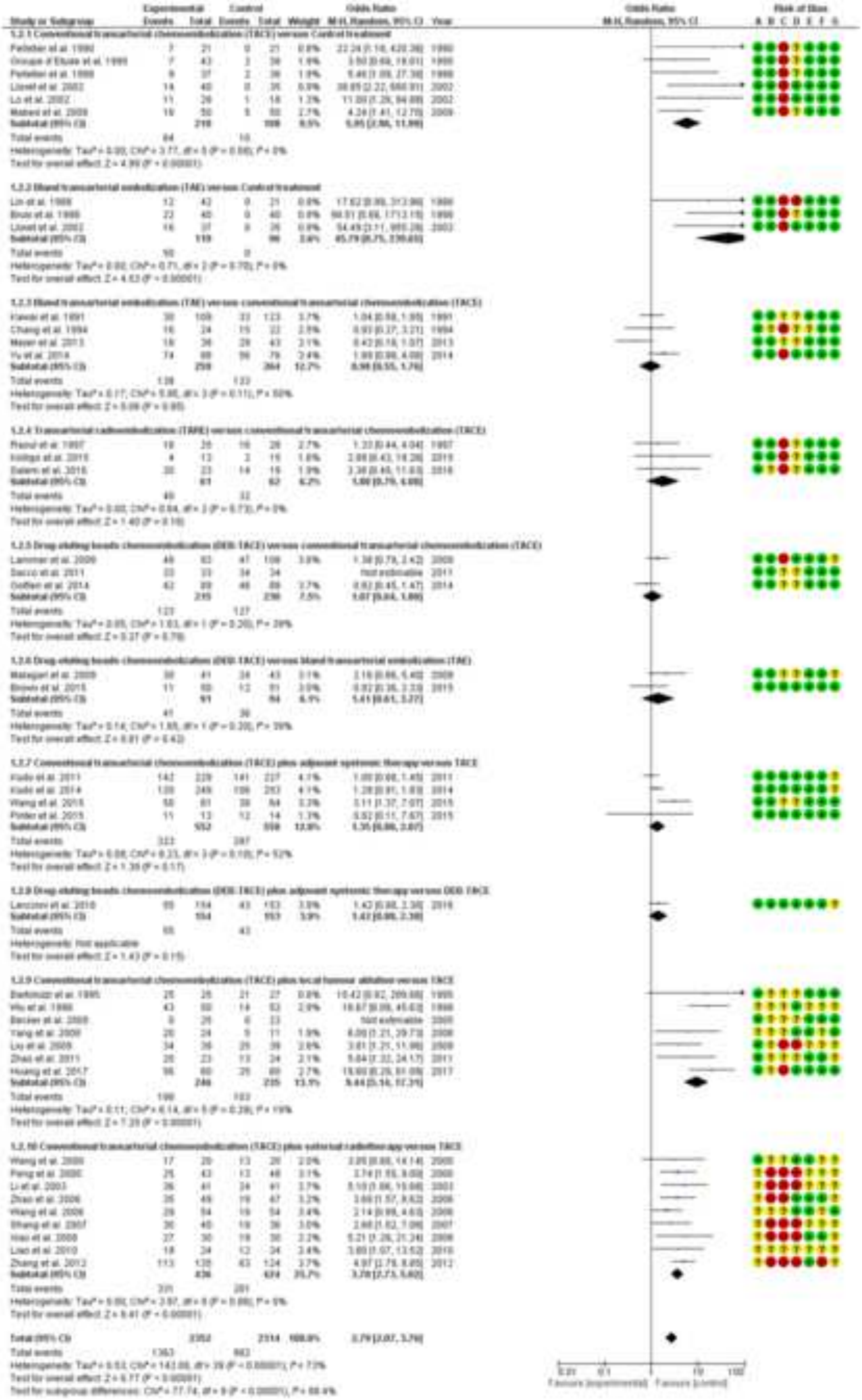

Figure 7

Click here to download Figure Fig7.TIF

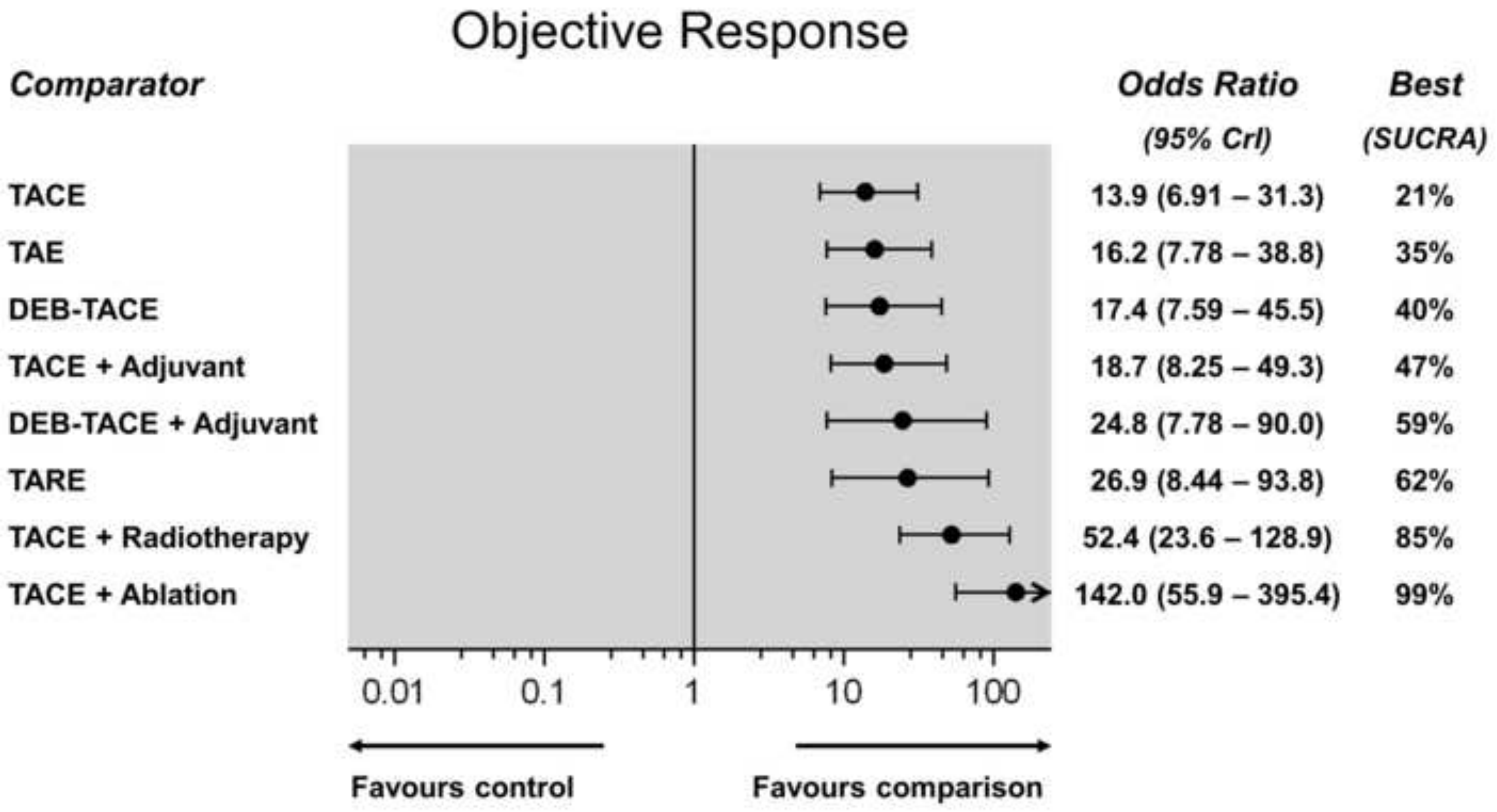

Figure 8

Click here to download Figure Fig8.TIF

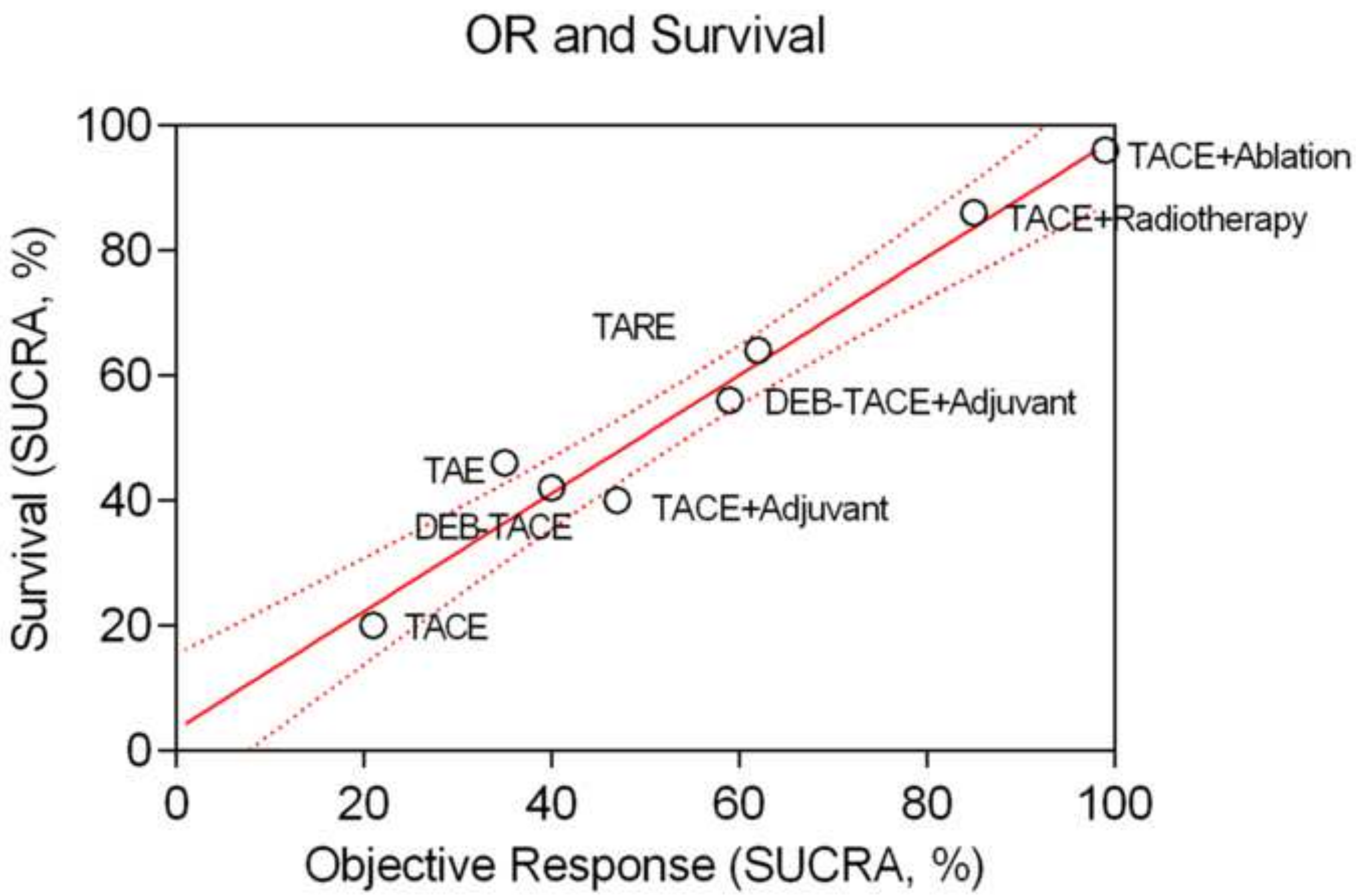

Figure 9

Click here to download Figure Fig9.TIF

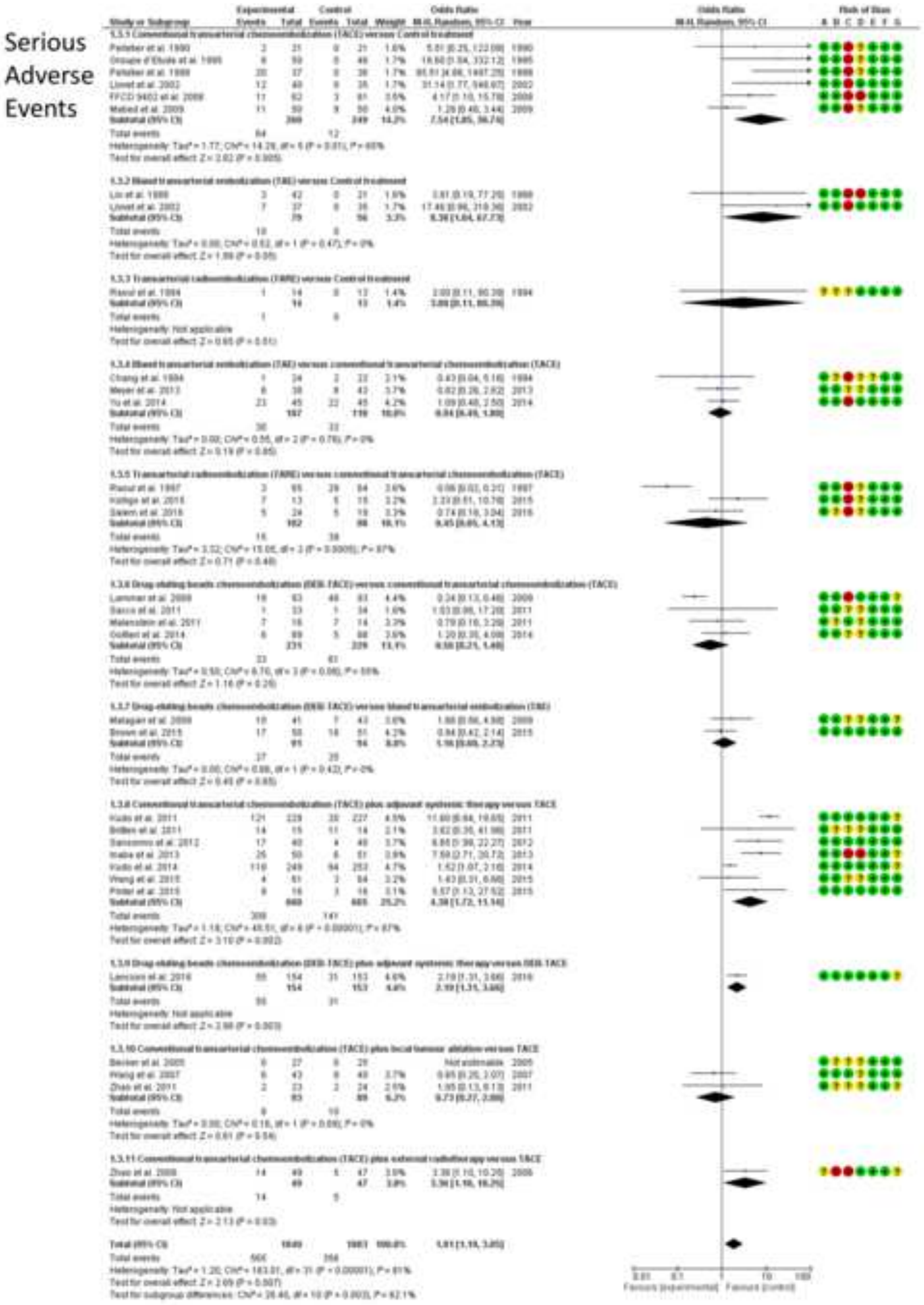

Figure 10 Click here to download Figure Fig10.TIF

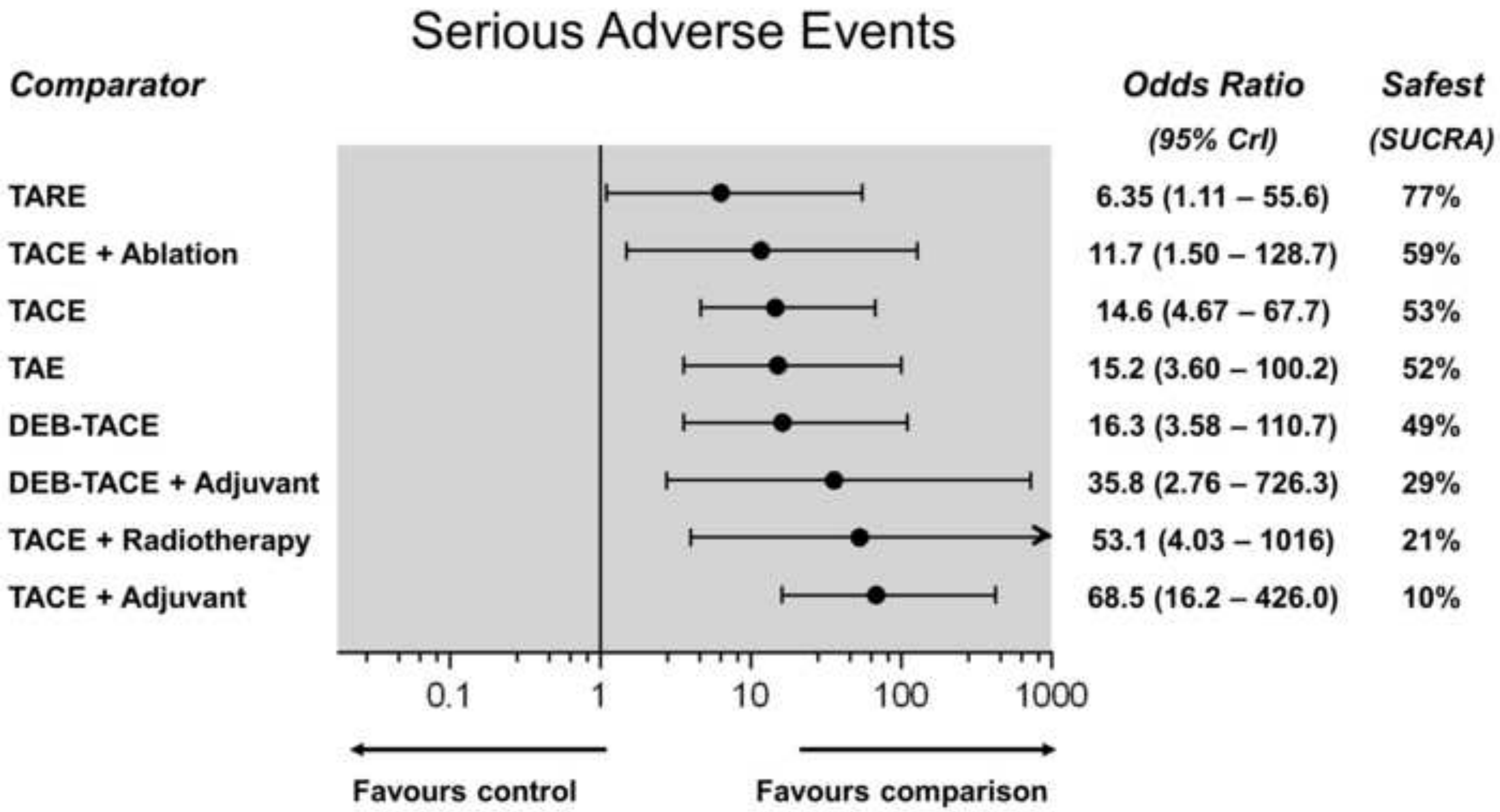

Figure 11

Click here to download Figure Fig11.TIF

**TACE+ablation**								
LOW (++oo) n=336 (60%)	**TACE+RT**							
VERY LOW (+ooo) n=96 (17%)	VERY LOW (+ooo) n=103 (18%)	**TARE**						
VERY LOW (+ooo) n=107 (19%)	VERY LOW (+ooo) n=119 (21%)	VERY LOW (+ooo) n=22 (4%)	**DEB-TACE +adjuvant**					
LOW (++oo) n=341 (61%)	LOW (++oo) n=427 (76%)	VERY LOW (+ooo) n=115 (21%)	VERY LOW (+ooo) n=115 (21%)	**TAE**				
VERY LOW (+ooo) n=247 (44%)	LOW (++oo) n=284 (51%)	LOW (++oo) n=89 (16%)	MODERATE (+++o) n=307 (55%)	MODERATE (+++o) n=350 (63%)	**DEB-TACE**			
LOW (++oo) n=285 (51%)	LOW (++oo) n=388 (69%)	VERY LOW (+ooo) n=98 (18%)	VERY LOW (+ooo) n=112 (20%)	LOW (++oo) n=283 (51%)	VERY LOW (+ooo) n=177 (32%)	**TACE +adjuvant**		
MODERATE (+++o) n=632 (>100%)	MODERATE (+++o) n=976 (>100%)	MODERATE (+++o) n=285 (51%)	VERY LOW (+ooo) n=136 (24%)	MODERATE (+++o) n=667 (>100%)	MODERATE (+++o) n=379 (68%)	MODERATE (+++o) n=643 (>100%)	**TACE**	
LOW (++oo) n=280 (50%)	LOW (++oo) n=360 (64%)	LOW (++oo) n=101 (18%)	VERY LOW (+ooo) n=105 (19%)	MODERATE (+++o) n=307 (55%)	LOW (++oo) n=278 (50%)	LOW (++oo) n=341 (61%)	MODERATE (+++o) n=538 (96%)	**Control**

Supplementary tables

Click here to access/download
Supporting Information
S1 Appendix.pdf

Click here to access/download
Supporting Information
S2 PRISMA PLOS checklist.doc

Comparative effectiveness of different transarterial embolization therapies alone or in combination with local ablative or adjuvant systemic treatments for unresectable hepatocellular carcinoma: A network meta-analysis of randomized controlled trials

Konstantinos Katsanos, MSc, MD, PhD, EBIR*,†, Panagiotis Kitrou, MD, PhD,*

Stavros Spiliopoulos, MD, PhD, EBIR ‡, Ioannis Maroulis, MD, PhD §,

Theodore Petsas, MD, PhD *, Dimitris Karnabatidis, MD, PhD, EBIR *

* Department of Interventional Radiology, Patras University Hospital, School of Medicine, Rion, 26504, Greece

† Department of Interventional Radiology, Guy's and St. Thomas' Hospitals, NHS Foundation Trust, King's Health Partners, London, SE1 7EH, United Kingdom

‡ Department of Interventional Radiology, Attikon University Hospital, School of Medicine, Athens, Greece

§ Department of Liver Surgery, Patras University Hospital, School of Medicine, Rion, 26504, Greece

Corresponding author: Dr. Konstantinos Katsanos

katsanos@med.upatras.gr, Assistant Professor, Department of Interventional Radiology, Patras University Hospital, School of Medicine, Rion, 26504, Greece, Tel: +30 2610 999218, Fax: +30 2610 999218

&

konstantinos.katsanos@gstt.nhs.uk, Honorary Consultant, Department of Interventional Radiology, Guy's and St.Thomas' Hospitals, NHS Foundation Trust, King's Health Partners, London, SE1 7EH, United Kingdom, NHS Foundation Trust, Tel: +44 (0)207 188 5550, Fax: +44 (0)207 928 8071

No conflicts of interest or relationship with the industry

Short title:

Transcatheter embolization therapies for unresectable hepatocellular carcinoma: Systematic review and network meta-analysis

Abstract

Background: The optimal transcatheter embolization strategy for patients with unresectable hepatocellular carcinoma (HCC) remains elusive. We conducted a systematic review and network meta-analysis (NMA) of different embolization options for unresectable HCC.

Methods: Medical databases were searched for randomized controlled trials evaluating bland transarterial embolization (TAE), conventional TACE, drug-eluting bead chemoembolization (DEB-TACE), or transarterial radioembolization (TARE), either alone or combined with adjuvant chemotherapy, or local liver ablation, or external radiotherapy for unresectable HCC up to June 2017. Random effects Bayesian models with a binomial and normal likelihood were fitted (WinBUGS). Primary endpoint was patient survival expressed as hazard ratios (HR) and 95% credible intervals. An exponential model was used to fit patient survival curves. Safety and objective response were calculated as odds ratios (OR) and accompanying 95% credible intervals. Competing treatments were ranked with the SUCRA statistic. Heterogeneity-adjusted effective sample sizes were calculated to evaluate information size for each comparison. Quality of evidence (QoE) was assessed with the GRADE system adapted for NMA reports. All analyses complied with the ISPOR-AMCP-NCP Task Force Report for good practice in NMA.

Findings: The network of evidence included 55 RCTs (12 direct comparisons) with 5,763 patients with preserved liver function and unresectable HCC (intermediate to advanced stage).All embolization strategies achieved a significant survival gain over control treatment (HR range, 0.42-0.76; very low-to-moderate QoE). However, TACE, DEB-TACE, TARE and adjuvant systemic agents did not confer any survival

benefit over bland TAE alone (moderate QoE, except low in case of TARE).There was moderate QoE that TACE combined with external radiation or liver ablation achieved the best patient survival (SUCRA 86% and 96%, respectively).Estimated median survival was 13.9 months in control, 18.1 months in TACE, 20.6 months with DEB-TACE, 20.8 months with bland TAE, 30.1 months in TACE plus external radiotherapy, and 33.3 months in TACE plus liver ablation. TARE was the safest treatment (SUCRA 77%), however, all examined therapies were associated with a significantly higher risk of toxicity over control (OR range, 6.35 to 68.5). TACE, DEB-TACE, TARE and adjuvant systemic agents did not improve objective response over bland embolization alone (OR range, 0.85 to 1.65). There was clinical diversity among included randomized controlled trials, but statistical heterogeneity was low.

Conclusions: Chemo- and radio-embolization for unresectable hepatocellular carcinoma may improve tumour objective response and patient survival, but are not more effective than bland particle embolization. Chemoembolization combined with external radiotherapy or local liver ablation may significantly improve tumour response and patient survival rates over embolization monotherapies. Quality of evidence remains mostly low to moderate because of clinical diversity.

Systematic review registration: CRD42016035796 (http://www.crd.york.ac.uk/PROSPERO)

Keywords

Hepatocellular carcinoma, embolization, chemoembolization, radioembolization, ablation, radiotherapy, survival, network meta-analysis, objective response, unresectable, systematic review

**Abbreviations**	
HCC	Hepatocellular carcinoma
RF	Radiofrequency ablation
MW	Microwave ablation
TAE	Transarterial embolization
TACE	Transarterial chemoembolization
DEB-TACE	Drug-eluting bead transarterial chemoembolization
TARE	Transarterial radioembolization
SIRT	Selective internal radiation therapy
BCLC	Barcelona Clinic Liver Cancer staging system
RCT	Randomized controlled trial
NMA	Network meta-analysis
QoE	Quality of Evidence
DIC	Deviance information criterion
EASL	European Association for the Study of the Liver
RECIST	Response Evaluation Criteria In Solid Tumors
OR	Objective response
SAE	Serious adverse events
HR	Hazard ratio
CTCAE	Common Terminology Criteria for Adverse Events
SUCRA	Surface Area Under the Cumulative Rankograms
CrI	Credible intervals

Introduction

Hepatocellular carcinoma (HCC) is the third leading cause of all cancer-related deaths globally and accounts for 90% of primary liver cancers and approximately 7% of all cancers, representing the fifth most common cancer in men and eighth for women.[1-3] Liver transplantation and surgical resection remain the proposed treatment options for very early and early stage HCC in good surgical candidates. Unfortunately, more than three-quarters of the patients are diagnosed during the intermediate or advanced stages of the disease and considered ineligible for curative resection.[1,4] In the past, the prognosis of unresectable HCC was poor and its management was limited to systemic pharmacotherapy, external radiotherapy or plain supportive treatments.[5] With the advent of Interventional Oncology that encompasses different percutaneous, image-guided, locoregional therapies,[6,7] treatment options for unresectable HCC quickly expanded to include transcatheter embolization with or without chemotherapy [8]; i.e. bland transarterial embolization (TAE)[9], conventional transarterial chemoembolization (TACE)[10] or chemoembolization with drug-eluting beads (DEB-TACE)[11]; and percutaneous liver ablation either with chemical agents like alcohol[12], or alternatively with application of radiofrequency (RF) or microwave (MW) energy.[13] Conventional TACE with the transcatheter delivery of a mixture of chemotherapy and embolic material is the current standard of care for unresectable intermediate or advanced stage HCC in patients with preserved liver function.[4,10] Local radiotherapy with the transarterial delivery of beta-emitting microparticles, currently known as radioembolization (TARE) or selective internal radiation therapy (SIRT) [14,15], is another emerging treatment for unresectable HCC. In addition, various combinations of locoregional

ablative treatments with adjuvant systemic therapies[16,17] or even external organ radiotherapy have been proposed.[18]

In general, interventional targeted embolization and ablative therapies for the treatment of unresectable HCC aim to increase overall patient survival, while limiting treatment-related side-effects, avoiding untoward complications, and improving the quality of life.[4] Theoretically, this can be accomplished by the inherent advantages of transcatheter (chemo)embolization treatments, which include a minimally invasive approach, enhanced pharmacokinetic profile and intra-tumorous bioavailability due to targeted drug delivery, and presumably more extensive tumour necrosis by combining the ischemic effect of embolization, while sparing surrounding normal liver parenchyma.[8,19] Moreover, transcatheter embolization treatments do not require general anesthesia or prolonged hospitalization periods.[3,8]

However, in spite of extensive animal and clinical investigations, and numerous randomized controlled trials (RCT) over the last decades, the optimal embolization treatment strategy for patients with intermediate to advanced stage HCC remains elusive.[7,8] The authors pursued to perform a mixed treatment comparison with quantitative statistical methods – network meta-analysis (NMA) - of the various transcatheter embolization therapies with or without local ablative or adjuvant systemic treatments for unresectable HCC. Comparative effectiveness of treatments that have or have not been directly compared with each other in head-to-head RCTs can be assessed in a network meta-analysis (NMA) using Bayesian statistics, on the condition that all competing therapies share a common chain or network of evidence.[20,21] We conducted a Bayesian network meta-analysis of all relevant randomized controlled trials to identify the best treatment option for patients with unresectable intermediate/advanced stage HCC.

Methods

Search methods

This systematic review has been registered in the PROSPERO public database (CRD42016035796; http://www.crd.york.ac.uk/PROSPERO). The authors initially collated randomized controlled trials reporting outcomes for unresectable HCC from different transarterial embolization strategies (alone or in combination with other treatments) from previously published relevant meta-analyses.[8,10,12,15,18,19,22-33] Subsequently, electronic searches of PubMed (Medline), EMBASE (Ovid), AMED, Scopus, CENTRAL, the China/Asia On Demand (CAOD) research portal, the PROSPERO and DARE meta-analyses databases as well as online material were performed until June 2017. The terms used included ‘hepatocellular carcinoma’, ‘primary liver cancer’, ‘unresectable’, ‘transcatheter’, ‘embolization’, ‘bland’, ‘chemoembolization’, ‘selective internal radiation therapy’, ‘radioembolization’, ‘radiotherapy’, ‘ablation’, ‘radiofrequency’, ‘alcohol’, ‘TAE’, ‘TACE’, ‘DEB-TACE’, ‘TARE’, ‘SIRT’, ‘sorafenib’, ‘bevacizumab’, ‘drug-eluting’, ‘anti-angiogenic’, ‘randomized’, ‘controlled trial’, and ‘meta-analysis’ along with the pertinent Medical Subjects Headings (MeSH) and combinations thereof with Boolean syntax. Keywords were searched using both British English and American English grammar (e.g. embolisation & embolization). In addition, Interventional Radiology, Medical Oncology and Radiation Oncology peer-reviewed journals in PubMed and Embase were examined. There were no restrictions on language, date or type of publication. KK, PK and SS performed the literature search and data extraction.

Trial selection and good meta-analysis practice

All steps of the trial selection process complied with the PRISMA (Preferred Reporting Items for Systematic reviews and Meta-Analyses) statement.[34] We searched for and included only RCTs comparing any of the aforementioned endovascular devices with each other, and reporting any of the primary and/or secondary outcome measures as defined below. RCTs were assessed for inclusion in the network meta-analysis (NMA) using a specifically structured question checklist developed in consensus by all authors. Published and unpublished randomised trials with an open-label, single-blind or double-blind design were eligible for inclusion provided that they investigated any type of transcatheter arterial embolization for unresectable hepatocellular carcinoma; with or without chemotherapy, plain or drug-eluting beads, radioactive embolic material; as a stand-alone treatment or in combination with other types of locoregional ablation; chemical or thermal or external radiotherapy; or combined with adjuvant systemic treatments; anti-angiogenic molecules or other agents. RCTs were included provided they reported any of the agreed outcome measures (see endpoints below).

A standardized data extraction form was used to collect the following information from all included trials (by KK, PK and SS): (1) characteristics of the study design methods (randomization, blinding, concealment of allocation, drop-outs, outcome reporting, risk of bias); (2) patient sample size and baseline clinical characteristics (age, gender, tumour size and morphology, liver function, vascular invasion, and performance status); (3) HCC staging according to the Okuda, BCLC, JIS or TNM classification systems; (4) description of active and control interventional treatment (chemotherapy regimen, type of embolic agents, treatment courses, dose and

fractionation of radiotherapy, adjuvant anticancer agents, other ablation procedures); and (5) clinical outcomes including overall patient survival, objective response of the treated index tumours, and serious adverse events. Terminology and classification of percutaneous and transcatheter image-guided liver therapies complied with standardized nomenclature and universal reporting criteria proposed by the Society of Interventional Radiology Technology Assessment Committee.[35]

The quality of the RCT trials was assessed independently by two of the authors with the Cochrane Collaboration's tool for evaluating the risk of bias that examines 7 different methodological items including randomized sequence generation, allocation concealment, blinding of patients and investigators, completeness and selectivity of outcome reporting, and other potential sources of bias.[36] Risk of bias assessment was performed by KK, SS and DK. To help inform healthcare decision making, all analysis methods, reporting quality and interpretation of findings complied with the 26-domain questionnaire of the ISPOR-AMCP-NCP Task Force Report for good practice in indirect treatment comparisons and NMA.[37] Finally, the quality of evidence (QoE) was assessed with Grading of Recommendations Assessment, Development and Evaluation (GRADE) system as adapted for the rating of pooled effect estimates in the case of NMA studies,[38,39] which considers directness, heterogeneity and imprecision of the mixed treatment comparisons as potential reasons for downgrading of the level of confidence.

Endpoints

In terms of survival outcome measures, few studies were found to report progression-free survival. Therefore, the primary endpoint was set at overall patient survival that was uniformly reported by all studies and was synthesized on the log-hazard scale as indicated for time-to-event outcomes in cancer studies.[40,41] Study-specific Hazard Ratios (HRs) and respective variances were retrieved from individual publications or back-calculated from the summary or Kaplan-Meier time-to-event data and quoted log-rank statistics with the equations of Parmar et al.[42] and methods of Tierney et al.[43]. If hazard rates were not available, HR was approximated from event rates under the assumption of constant hazards. Random effects models were fitted to account for clinical diversity and heterogeneity and HRs with 95% credible intervals were calculated.

Treatment effectiveness was assessed by the radiologic response on cross-sectional follow-up imaging as reported by each individual RCT. The effectiveness endpoint was set at Objective response (OR) of the index tumour defined as Complete and Partial Response (CR+PR) according to well-accepted classification systems including the World Health Organization (WHO),[44] the European Association for the Study of the Liver (EASL),[45] the Response Evaluation Criteria In Solid Tumors (RECIST),[46] and modified RECIST (mRECIST)[47] schemes.

All outcome measures of this systematic review were defined according to previously published terminology and accepted reporting criteria for transcatheter therapies for liver malignancies.[35] The safety and toxicity endpoint was set at Serious Adverse Events (SAE) grade 3 and above as defined by the National Cancer Institute Common Terminology Criteria for Adverse Events (CTCAE, version 4.0).[48] All

endpoints were analyzed on an intention to treat basis as recommended for reporting and meta-analysis of RCTs. Any disagreements were resolved by consensus.

Statistical methods

Direct pairwise meta-analyses of head-to-head comparisons were performed using standard frequentist approaches (RevMan 5.2, Cochrane Collaboration). Mixed treatment comparisons of the RCT network were performed with Bayesian inference (WinBUGS 1.4.3, MRC Biostatistics Unit at Cambridge, United Kingdom). Bayesian hierarchical modeling of the present network meta-analysis complied with the NICEDSU (National Institute for Health and Excellence Decision Support Units) guidelines.[49-51] Count statistics of treatment toxicity and objective tumour response were analyzed with a Bayesian random effects model with a binomial likelihood to calculate relative treatment effects expressed as Odds Ratios (OR) between different treatments. Overall patient survival was analyzed with a Bayesian random effects model with a normal likelihood incorporating log hazard ratio statistics from individual trials to calculate Hazard Ratios (HR) between competing treatments.[40] Summary statistics of relative treatment effects are reported as the median and accompanying 95% Credibility Intervals (95% CrI) of the posterior distribution. CrIs serve the same purpose as confidence intervals in frequentist statistics.

In addition, we fitted the respective patient survival curves with an exponential model up to 5 years using absolute survival estimates of conventional TACE, which was the most common comparator and with the largest sample size, as the anchor treatment. Median patient survival (half-life) for each treatment was calculated by combining the fitted hazard rate (exponential decay constant) of the anchor treatment (random effects model) with the pairwise posterior median HR calculated by the Bayesian model for the respective treatment. We also constructed rankograms of cumulative rank probabilities of how each treatment ranks against each other in terms of being

the 1st, 2nd, 3rd, etc. best treatment option. We present hierarchies of the effectiveness and safety of competing treatments based on their cumulative rank probabilities and the Surface Area Under the Cumulative Rankograms (SUCRA) as proposed by Salanti et al.[52]

The information size (IS) required for a valid meta-analysis may be assumed to be at least as large as the sample size of a single well-powered RCT designed to confirm or reject the null hypothesis [53,54]. To assess the adequacy of available information size across different pairwise comparisons that combined direct and indirect evidence within the NMA framework, we performed calculations of the effective sample size for each treatment comparison. We employed the methods proposed by Thorlund and Mills for quantifying sample and information size in NMAs after adjusting for statistical heterogeneity observed in pairwise meta-analyses of individual nodes [55]. Consequently, statistical power and strength of evidence for each treatment comparison may be evaluated by the information fraction (IF; percentage of information size) available for each comparison.

Heterogeneity, consistency, and meta-regression

Heterogeneity was evaluated with the posterior median of the between-trials standard deviation (σ),[50] while small study effects and publication bias were evaluated by visual inspection of standard and comparison-adjusted funnel plots.[56] Because of conceptual differences in study designs and anticipated diversity in baseline demographics, the observed baseline risk of outcome measures may vary between the reference treatment arms. Baseline risk is a proxy for unmeasured but important patient-level characteristics that may relate to significant clinical heterogeneity. Hence, we extended our analysis to a meta-regression model on trial-specific baseline risk of the control arms to account for the uncertainty and clinical heterogeneity introduced by differences in baseline characteristics of unresectable HCC cohorts.[57] In addition, extensive consistency, sensitivity, and meta-regression analyses were performed to explore heterogeneity and confirm validity as proposed by the ISPOR-AMCP-NCP Task Force.[37,50] The validity and robustness of NMA depend largely on the distribution of effect modifiers (covariates) not only between studies with the same contrast (i.e. heterogeneity in the case of standard pairwise meta-analysis) but also between different contrasts (i.e. inconsistency between direct and indirect contrast estimates).[58] Any disagreement between the direct evidence available for a specific contrast and the indirect evidence inferred by the rest of the network would give rise to inconsistency. In the case of NMA studies, the risk of network inconsistency is greatly reduced if between-trials heterogeneity is low.[59] To exclude any loop-specific inconsistency and confirm the transitivity assumption, pairwise direct and indirect effect estimates of closed loops of evidence were inspected for any disagreement and the results of the consistency model were

compared with those of an alternative unrelated mean effects model without any consistency constraints.[49]

WinBUGS modeling

Bayesian inference with WinBUGS employs Markov Chain Monte Carlo (MCMC) simulation to calculate the posterior distributions of the interrogated nodes within the framework of the chosen model and likelihood function on the basis of prior assumptions. For the purposes of this analysis, we first fitted a Bayesian hierarchical model for multiple comparisons of different treatment options control best supportive treatment as the reference. Posterior medians (95% CrI) of the point estimates against control treatment were calculated using the freely available NetMetaXL software package[60], and by custom code following the examples of Woods et al.. [40] Vague priors were used for all treatment effects and for between-trials heterogeneity variance to avoid bias.

Three Markov chains were compiled and run, while convergence was confirmed with the Brooks–Gelman–Rubin diagnostic tool and by inspection of history plots of monitored nodes. An initial burn-in simulation of 50,000 iterations was discarded and inference of final summary statistics was based on simulation of an additional 100,000 iterations.[51] Global model fit and parsimony was compared between different fitted models to decide on the most accurate model. The goodness of fit was compared with the posterior mean of the total residual deviance and the Deviance Information Criterion (DIC) criterion. Residual deviance must approximate the total number of study arms analyzed in the case of a good model fit the and generally the model with the lowest DIC is preferred.[51] The level of statistical

significance was set at $\alpha=0.05$ for frequentist inference, while relative treatment effect results associated with 95% CrI that did not cross unity were considered significant in the case of Bayesian inference.

Results

Network of evidence

Following the PRISMA selection process, 5,975 scientific records were screened for potential inclusion in the network meta-analysis on the basis of their title and abstract (Figure 1). Finally, 55 RCTs (including one three-arm study [61]) published between 1988 and 2017 and reporting on 5,763 patients in total were included and synthesized within a Bayesian framework. The network of evidence involved nine treatment nodes (eight active and one control) and was well connected with conventional TACE as the most common comparator (Figure 2). Four treatment nodes referred to different types of trans-arterial embolization therapy alone (conventional TACE, or DEB-TACE, or TARE, or bland TAE) and another four treatment nodes referred to a combination of transarterial chemoembolization with other locoregional or systemic treatments (TACE and external radiotherapy, or TACE and percutaneous liver ablation, or TACE and adjuvant systemic, or DEB-TACE and an adjuvant systemic agent). Direct evidence was available for 12 comparisons (Table 1); three of them were informed by a single RCT and the rest by more than one RCT (median 3.5; range, 1-11 trials).

TACE was investigated versus Control symptomatic treatment in 8 studies [61-68], versus bland TAE in 4 studies [9,69-71], versus DEB-TACE in 4 studies [72-75], versus TARE in 3 studies [76-78], versus TACE combined with adjuvant systemic agents in 8 studies [17,79-85], versus TACE combined percutaneous liver ablation in 10 studies [86-95], and versus combined TACE and external radiotherapy in 11 studies [96-106]. In addition, DEB-TACE was compared directly with TAE in 2 studies [107,108], with TARE in 1 RCT [109], and with DEB-TACE plus systemic

sorafenib in 1 RCT [16]. Finally, TAE alone was compared with Control treatment in 3 studies [61,110,111], and TARE with Control in 1 study [112]. There were 3 high-quality RCTs with low risk of bias; the rest of the studies had unclear (at least one unclear domain) to high (at least one high-risk domain) risk of bias according to the COCHRANE tool for risk of bias assessment. The latter was caused by performance bias (absent or unclear blinding of participants and personnel) or detection bias (blinded outcome assessment) in the majority of the studies.

Fifty-one out of the 55 studies recruited patients with unresectable hepatocellular carcinoma classified as intermediate to an advanced stage (i.e. BCLC stage B-C, Okuda stage I-II, or AJCC TNM stage II-III) and 4 studies included unresectable early stage HCC [74,78,100,105]. All studies included patients with preserved liver function (Child-Pugh A and B) and with a predominantly male gender (range, 50-96%). Good performance status (PS: 0-1 or KPS≥65%) was reported in most of the cases and the percentage of randomized patients with a multinodular or diffuse type of HCC varied widely (median, 57%; IQR, 39-67%; max 100%). Fourteen out of the 55 studies reported inclusion of variable rates of patients with documented portal vein thrombosis (range, 2-100%). A detailed description of baseline patient demographics and clinical characteristics is provided in Supplemental Table 1.

In the TACE treated arms, conventional transarterial chemoembolization was performed with a lipiodol emulsion of a single chemotherapy agent (doxorubicin [61,68,70,73-75,78,83,86], or epirubicin [63,66,72,76,80], or cisplatin [9,62,64,67,69,71,77,82], or mitomycin [87], or a combination chemotherapy regimen [65,79,81,84,85,89,93,95,97,99-106], and was most often followed by gelfoam or other particle embolization of the primary feeding vessels. Meyer et al. performed cisplatin infusion first followed by particle embolization 4-6 hours later [9]. In case of

TAE, bland embolization was performed with gelfoam and/or microparticles (microspheres) [9,61,69,70,107,108,110,111] or alcohol [71]. DEB-TACE involved transcatheter delivery of doxorubicin-eluting DC beads [16,72-75,107-109], and TARE of a beta-emitter including ^{131}I-labeled Lipiodol [77,112] or Yttrium-90 microparticles [76,78,109]. Adjunctive systemic agents included sorafenib [16,81,84], brivanib [17], bevacizumab [79,83], arsenic trioxide [85], TSU-68 [80], IFN-a [82]. Locoregional liver ablation was reported by means of multiple sessions of radiofrequency ablation (RFA) [88,89,93,94] or percutaneous ethanol injection (PEI) [86-88,90-92] or argon-helium cryoablation [95]. Finally, external radiotherapy was delivered by 3D conformal [97,98,100,104-106] or moving stripe fractionated protocols [99,101-103]. Active and control treatment protocols are described in detail in Supplemental Table 2. Median follow-up was 3 years on a trial basis (interquartile range, 2.0–3.5 years; max 6.0 years).

Patient Survival

Survival outcomes were reported by 51 RCTs (incl. one 3-arm) reporting on 5,394 patients and 12 direct comparisons in total. Direct meta-analyses (Figure 3) confirmed a significant survival benefit of TACE over best supportive therapy (HR: 0.76; 95%CI: 0.64-0.91) and a similar survival benefit between TAE and TACE (HR: 0.87; 95%CI: 0.71-1.07). In addition, TACE performed worse than TACE plus radiotherapy (HR: 0.60; 95%CI: 0.53-0.69) and TACE plus ablation (HR: 0.54; 95%CI: 0.46-0.65). The NMA synthesis showed that all embolization treatments achieved a significant survival benefit over control except DEB-TACE with adjuvant sorafenib (HR range, 0.42-0.76). Figure 4 shows a hierarchy of different treatments

according to the SUCRA statistic and the respective Hazard Ratios (HR). TACE, DEB-TACE, TARE, and adjunctive systemic agents (combined with TACE or DEB-TACE) did not confer a survival benefit over bland TAE. TACE combined with external radiation therapy (SUCRA 86%), or percutaneous tumour ablation (SUCRA 96%), were the most effective treatment strategies. NMA heterogeneity was low (σ= 0.06; 95%CrI: 0.001-0.17). A league table of all pairwise survival comparisons from the NMA synthesis is provided in the Supplemental material.

Survival model

The fitted exponential survival model is shown in Figure 5 (posterior median of survival projections; 95% CrIs). Conventional TACE was the most common comparator node (43 out of the 51 RCTs reporting patient survival) and was used as the anchor treatment (least squares non-linear fit R^2=0.999) for calculating expected median survival outcomes for each of the other treatment options. Median survival period in case of control best supportive treatment was 13.9 months (95%CI: 11.0-17.7) and increased to 18.1 months (95%CI: 15.6-21.6) in the case of TACE, 20.6 months (95%CI: 14.5-29.4) with DEB-TACE, and 20.8 months (95%CI: 16.2-27.1) with bland TAE. Adjuvant systemic agents did not provide any significant survival benefit over transarterial therapies. Median survival increased to 24.3 months (95%CI: 16.8-35.3) in the case of TARE. Projected median survival exceeded 30 months when conventional TACE was combined with external radiotherapy (30.1 months; 95%CI: 24.6-37.3) or with percutaneous liver tumour ablation (33.3 months; 95%CI: 26.4-42.5).

Objective Response

Rates of the objective response of the treated tumour lesions were reported by 41 RCTs including 4,669 patients and informing 10 direct treatment comparisons. According to direct meta-analyses (Figure 6), both TACE (OR: 5.95; 95%CI: 2.96-11.99) and TAE (OR: 45.8; 95%CI: 8.75-239.7) demonstrated a strong response rate over control treatment. In line with the survival analysis, objective response was also better in case of TACE combined with radiotherapy (OR: 3.7; 95%CI: 2.7-5.0) or ablation (OR: 9.44; 95%CI: 5.14-17.3) over TACE alone. In the NMA analysis, all embolization treatments achieved a significant tumour response. Figure 7 shows a hierarchy of comparative treatment effectiveness according to the SUCRA statistic. Combinations of conventional TACE with external radiation therapy (SUCRA 85%) or percutaneous tumour ablation (SUCRA 99%) were the most effective treatment options. TACE, DEB-TACE, TARE and adjunctive systemic agents (combined with TACE or DEB-TACE) did not improve the objective response of treated tumours compared to bland embolization alone (TAE). TACE with adjunctive ablation achieved a significantly better objective tumour response compared to all other embolization mono- or combination therapies (OR range, 2.17-10.2; league table in the Supplemental material). NMA heterogeneity was low (σ= 0.29; 95%CrI: 0.03-0.63). Comparative effectiveness results of overall patient survival were corroborated by the hierarchical SUCRA results of tumour objective response with high correlation between the two outcome measures (linear regression fit R^2=0.959 – Figure 8).

Serious Adverse Events

Treatment-related serious adverse events (SAE) were reported by 32 RCTs including 3,610 patients for 11 direct treatment comparisons (Figure 9). Safety ranking of different embolization therapies on the basis of cumulative rank probabilities (SUCRA, %), along with the respective ORs (95%CrI) against control as a reference, are shown in Figure 10. TARE was the safest treatment (SUCRA 77%), however, all examined therapies were associated with a significantly higher risk of SAE compared to control (OR range, 6.35-68.5). Most of the other pairwise comparisons showed no significant differences between different embolization regimes in terms of SAE. TACE combined with adjuvant systemic therapies was the highest-risk treatment (SUCRA 10% - league table in the Supplemental material). Between-trial heterogeneity was low (σ= 1.01; 95%CrI: 0.61-1.64).

Heterogeneity, consistency, and meta-regression

There was good agreement between the consistency and inconsistency (unrelated mean effects) models, suggesting a robust and homogeneous network of evidence (Supplemental table 3). Between-trial statistical heterogeneity in the random effects Bayesian models was low compared to the respective posterior treatment effects (Supplemental table 4). Consequently, application of a fixed effect Bayesian model produced similar numerical results with slightly tighter credible intervals (Supplemental league tables). However, model fit according to the residual deviance and DIC criteria was better in the case of the random effects analyses and hence those were preferred and presented in the present article (Supplemental table 4). There was no obvious asymmetry at visual inspection of funnel plots to suggest publication bias, except in the case of Objective Response (Supplemental funnel plots). However, that was not evident any more on the comparison- adjusted funnel plot (Supplemental OR funnel plot with comparison-specific adjustments). Random effects meta-regression analyses to check for risk modifiers demonstrated only weak non-significant correlations in the majority of the tests. Multinodular HCC was the only variable found to be strongly and significantly related to increased rate of adverse events, as well as of higher rates of radiological response (Supplemental table 5).

Strength and Quality of evidence

We calculated a sample size of 560 patients as adequate for the detection of a treatment effect of 30% relative risk reduction of death (HR=0.7) with a type I error 5% and type II error 20% (power 80%) assuming an average patient survival of 50% at 2 years and a 10% rate of drop-outs or lost to follow-up. Compared to that, the IF was found to be low-to-modeate (range, 4-51%) in case of TARE, and high (range, 50-100%) in all mixed treatment comparisons informed by both direct and indirect evidence. Figure 11 summarizes the strength (effective sample size and IF) and QoE according to the GRADE system for all treatment comparisons in the present NMA.

The GRADE system for assessing quality of evidence considers directness, heterogeneity and imprecision of the mixed treatment comparisons as potential reasons for downgrading the level of confidence in NMA results [113]. We have found no inconsistency and statistical heterogeneity was generally low in the present NMA, however, clinical diversity was evident in the baseline demographics of different RCTs. Hence, in the current analysis, QoE was first downgraded universally because of between-trial diversity in terms of baseline patient characteristics and type and mixture of antineoplastic and/or embolic agents used (Supplemental tables 1 & 2). Second, it was further downgraded in certain comparisons because of the absence of direct comparative evidence (indirectness).

To evaluate imprecision, we gauged the effective sample size and information fraction of each comparison. We considered an IF<50% as a measure of weaker evidence and potential imprecision; hence, QoE was further downgraded to very low in the relevant comparisons. Overall, there was moderate QoE with sufficient information size when comparing TACE+ablation, TACE+RT, TACE+adjuvant

systemic agents and TAE, over TACE alone. Information was also strong enough with moderate QoE in the case of TARE versus TACE, in the cases of TAE compared with control or TACE or DEB-TACE, and in the case of TACE over control treatment (Figure 11).

Discussion

Contrary to a standard meta-analysis that pools studies comparing a certain pair of treatments, network meta-analysis (NMA) is an established methodology capable of inferring the high level of evidence about any number of treatments by combining direct and indirect randomized comparative research into a single unified analysis while respecting randomization of individual clinical studies.[114] To our knowledge, this is the first comprehensive mixed treatment comparison analysis evaluating the safety and effectiveness of different transarterial embolization therapies either alone or in combination with local ablative or adjuvant systemic treatments for unresectable hepatocellular carcinoma. Most of the patients with hepatocellular carcinoma are diagnosed late at the intermediate-advanced stages of the disease and are ineligible for potentially curative treatments like liver transplantation, resection or curative thermal ablation. According to GIDEON, the largest global observational registry of unresectable HCC to date including more than 3,200 cases, more than half of all HCC patients receive TACE as their primary treatment mode [115]. A lipiodol emulsion of an anticancer agent; usually doxorubicin; followed by gelfoam or other particle embolization remains the most popular form of TACE [8]. Adoption of TACE with an oil emulsion of antineoplastic agents has been primarily driven by early RCTs of bland TAE or TACE versus conservative management more conducted than 10 years ago [8,61,64,67,68,110,111]. However, not only new treatments have emerged like DEB-TACE or TARE or combined locoregional treatments, but above all guideline-recommended therapy for unresectable HCC remains controversial. The ESMO-ESDO guidelines advocate TACE for large or multinodular HCC with good liver function [116], whereas the Canadian CEPO (Comité de l'évolution des pratiques en oncologie) recommends TACE as the standard of care for palliative

treatment of eligible HCC patients, but specifically advises against the use of TAE or TARE [117]. In the meantime, a recent heavily disputed Cochrane meta-analysis questioned the firmness of evidence supporting either TAE or TACE in unresectable HCC in general [33]. Hence, the survival benefit of transarterial embolization therapies for unresectable HCC is still under dispute [118].

Most importantly, the present NMA of 55 RCTs comprising more than 5,700 patients has shown that transarterial (chemo)-embolization strategies can confer a clear survival benefit in patients with unresectable HCC by reducing the hazard of death in the range of 24% (in case of TACE) up to 34% (in case of TAE and DEB-TACE). However, surprisingly, none of the transcatheter chemo-embolization options (i.e. TACE and DEB-TACE as standalone treatments or even combined with adjuvant systemic agents) was any better than traditional bland transarterial embolization (TAE). The above findings had a large information size and moderate QoE being supported by direct evidence by 3 trials examining TAE versus best supportive therapy (publication date 1988-2002)[61,110,111], 4 trials testing TAE versus TACE (1994-2014)[9,69-71], and 2 trials comparing TAE versus DEB-TACE (2010-2016)[107,108]. Internal radiation therapy (TARE) produced an even higher survival benefit (43% reduction of the hazard of death) informed by 3 trials [76-78], but its effectiveness was not significantly better than TAE and evidence was informed only by a moderate information size (very low-to- moderate QoE).

The aforementioned findings, on one hand, support the notion that ischemic necrosis induced by transcatheter embolization of the tumour feeding arteries is the primary mode of therapy in HCC and on the other hand question the need for the widely employed use of antineoplastic agents (most often doxorubicin) as part of the majority of HCC embolization regimens. Neoangiogenesis is a well-known hallmark

of hepatocellular carcinoma [119], and hepatic transarterial embolization induces virtually immediate tumour cell death evident on imaging within 24hours [107]. The addition of chemotherapy has been long thought to allow for enhanced intratumoral drug delivery and retention when combined with transarterial ischemic necrosis [120], but HCC is notorious for its low sensitivity to chemotherapy and tendency to develop multidrug resistance [121]. The current results have found moderate QoE according to the GRADE system that TAE is as good as any other chemo-embolization treatment contesting the widespread use of intra-arterial doxorubicin and other chemotherapeutic results.

Another interesting result was that the addition of locoregional ablation in the form of percutaneous ablation or external radiotherapy had a strong additive effect in improving objective response and prolonging patient survival. The combination of TACE with external radiotherapy achieved better response rates (SUCRA 85%) and improved patient survival (SUCRA 86%) that were both significantly better than plain TAE or TACE (low-to-moderate QoE, and IF 61-100%). The combination of TACE with some form of percutaneous ablation (microwave or RF or alcohol) was also significantly better than TAE or TACE and was found to be the best performing treatment ranking first in terms of both OR (SUCRA 99%) and survival (SUCRA 96%). The latter findings support the enhanced therapeutic outcomes in case of combined transarterial and locoregional ablative treatments [18]. Pathology studies have shown that palliative transarterial lipiodol-based treatments may achieve >90% necrosis in widely variable rates; 26-70% of the treated nodules; depending on technique, lesion size and arterial anatomy [122,123]. Hence, it would be very sensible to combine (chemo)-embolizations with other ablative therapies in order to achieve higher rates of tumor necrosis and thereby prolong patient survival.

Comparative safety analysis demonstrated that TARE with a beta-emitter was the safest treatment (SUCRA 77%), whereas combined TACE and liver ablation had the most favourable safety and effectiveness profile (SUCRA 59% and 99%, respectively).

Overall, the findings of the present network meta-analysis are very much in line with the results of several individual direct meta-analyses exploring individual (chemo)-embolization strategies. A recent overview of the major findings of meta-analyses on the management of hepatocellular carcinoma summarized the body of evidence from more than 20 direct meta-analytic reports on embolization therapies for inoperable liver cancer [124]. Seven meta-analyses compared the outcomes of TACE/TAE versus no active treatment or supportive care and overall survival outcomes favoured TACE/TAE [27,33,125]. Another 3 reports compared the outcomes of TACE versus TAE and concluded that there was no survival difference [27,126,127]. Furthermore, 3 reports looked into DEB-TACE versus TACE and found benefit only in terms of tumour response like in the present work [24,128,129]. Four meta-analyses reported outcomes of TACE combined with sorafenib versus TACE alone and again found no survival benefit with the addition of sorafenib [29,130]. Last, there were 3 meta-analyses exploring the combination of TACE with plain external or conformal radiotherapy and also found that combination therapy produced superior survival outcomes [18,124]. The present work corroborates all of the above in a single model and further raises the combination of TACE and percutaneous tumour ablation as the best treatment option in terms of both local tumour response and overall patient survival.

We consider the fitted survival model another particular strength of the present study as it may provide absolute expected median survival outcomes for each treatment

and help clinicians optimize their decision-making process as well as guide the informed consent of the patients. A previous meta-analysis of the expected survival rates of untreated patients in the control arms of randomized studies of HCC has provided interesting insights into the natural history of this largely heterogeneous patient group. Projected median survival was 12 months in the case of intermediate stage (BCLC category B) cases, and around 6 months in the case of advanced stage (BCLC category C) patients [131]. A recently released systematic review and meta-analysis of more than 10,000 patients with unresectable HCC treated with lipiodol TACE has reported a weighted median survival rate of 19.4 months (95%CI: 16.2-22.6 months) [8]. The above numbers compare favourably with the results of our comparative survival model. In the present analysis, the weighted median survival was calculated to be 13.9 months (95%CI: 11.0-17.8 months) across the control arms of best supportive care and projected to be 18.1 months (95%CI: 15.6-21.6 months) in the TACE arms (anchor treatment). The ESMO-ESDO guidelines quote an expected median survival following TACE treatment of approximately 20 months in the case of BCLC intermediate stage and no more than 11 months in the case of advanced stage HCC. Hence, the authors consider the current evidence synthesis to reflect mostly a population of predominantly intermediate stage hepatocellular carcinoma in line with guideline-recommended use of most transarterial embolization therapies. In parallel with comparative effectiveness results, expected survival outcomes were similar between TAE (median 20.9 months) and different TACE approaches (median range, 18.1-23.1 months), numerically better with TARE (median 25.4 months) and significantly improved with the addition of external radiotherapy or ablation (median >30 months).

Arguably, unresectable HCC is characterized by significant heterogeneity in lesion size, unifocal or multinodular or diffuse patterns of disease, and variable degrees of underlying liver dysfunction [5,8,131]. Experts have long advised against TACE in Child-Pugh B patients, whereas TARE and external radiation have been proposed for the more liver dominant types of disease. Hence, one treatment type cannot fit all this heterogeneous category of patients [132]. The authors believe that combination treatments customized to individual patient profiles on the basis of the presented treatment rankings may deliver better clinical results and further improve survival of patients presenting with unresectable HCC and preserved liver function. Most interestingly, we have shown a clear synergy between transarterial embolization and locoregional ablation that needs to be explored further in larger scale studies in properly selected patients.

There are certain limitations to the present analysis. Network meta-analyses are inherently more prone to uncertainty and bias compared to classical meta-analysis. In addition, network meta-analyses are often exploratory to identify areas for more targeted scientific research and to help inform the design of future RCTs. However, sensitivity, consistency, and heterogeneity analyses support the validity of our results. Another limitation is that all 55 studies span 2 decades of medical practice and patient population reflects, as expected, the well-known clinical and anatomical heterogeneity of patients with unresectable HCC. Nonetheless, our survival model is in close agreement with real-life practice supporting the notion of generalizability of our findings. Finally, we have not accounted for differences in the race and geography as certain clusters of studies were most often performed in Asia (e.g. a combination of TACE and external irradiation) or the Western countries (e.g. TACE and DEB-TACE options).

In conclusion, TACE, DEB-TACE, TARE and adjuvant systemic agents neither improved tumour objective response nor conferred any patient survival benefit compared to bland particle embolization (TAE). Combinations of TACE with external radiation or liver ablation achieved the best tumour response and patient survival. Therefore, the current trends of chemoembolization practise are clearly open to question and international guidelines may need to be revised. However, quality of evidence remains low to moderate, and clearly more and larger studies are needed, especially in the fields of radioembolization, on the role of new embolic particulate agents and to further elucidate the synergy of combined transarterial and ablative liver treatments.

Author contributions

All authors have made significant contributions to the submitted work by participating in the conceptualization of the present meta-analysis, selection of the included trials and abstraction of the relevant data, drafting, revision and final approval of the submitted manuscript. The corresponding author was personally responsible for all Bayesian statistical modeling and preparation of the initial manuscript draft. All authors meet authorship criteria according to the ICMJE recommendations: (1) substantial contributions to conception and design, acquisition of data, or analysis and interpretation of data; 2) drafting the article or revising it critically for important intellectual content; 3) final approval of the version to be published, and 4) agreement to be accountable for all aspects of the work in ensuring that questions related to the accuracy or integrity of any part of the work are appropriately investigated and resolved.

Funding

There was no funding source for this study. All authors had unrestricted access to the datasets and can take responsibility for the integrity of the data and the accuracy of the data analysis. The lead and corresponding author (K.K.) had access to the whole dataset, performed all statistical analyses and has final overall responsibility for the submitted version of the manuscript (study guarantor). The lead and corresponding author (K.K.) affirms that the manuscript is an honest, accurate, and transparent account of the study being reported. All raw data are provided in the direct frequentist plots provided in the Supplementary material. WinBUGS code and other statistical files used are available on request by the authors.

Disclosures

None of the authors has any conflicts of interest to declare. All authors have completed the ICMJE uniform disclosure form at www.icmje.org/coi_disclosure.pdf and declare that: (1) none of them have received support from any company for the submitted work; (2) none of them have any relationships with companies that might have an interest in the submitted work in the previous 3 years; (3) their spouses, partners, or children have no financial relationships that may be relevant to the submitted work; and (4) none of them have any non-financial interests or other relationships or activities that could appear to have influenced the submitted work.

Search strategy

1 “hepatocellular carcinoma”[MESH], 2 “hepatocellular carcinoma”[TW], 3 “liver cancer”[MESH], 4 “liver cancer”[TW]

5 “unresectable”[TW], 6 “inoperable”[TW], 7 “advanced”[TW]

8 “Clinical trial”[Mesh], 9 “Randomized Controlled Trial”[Mesh], 10 “Clinical trial”[TW], 11 “Randomized”[TW], 12 “Meta-analysis”[Mesh], 13 “Meta-analysis”[TW]

14 “embolization”[MESH], 15 “chemoembolization”[MESH], 16 “sorafenib”[MESH], 17 “embolization”[TW], 18 “chemoembolization”[TW],19 “sorafenib”[TW], 20 “transcatheter” [TW], 21 “ablation”[TW], 22 “radiotherapy”[TW], 23 “radiation”[TW], 24 “radioembolization”[TW], 25 “selective internal radiation therapy”[TW], 26 “radiofrequency”[TW], 27 “alcohol”[TW], 28 “drug-eluting”[TW], 29 “anti-angiog*”[TW], “bevazicumab”[TW], 30 “TACE”[TW], 31 “TAE”[TW], 32 “DEB-TACE”, 33 “TAE”[TW], 34 “SIRT”[TW], 35 “TARE”[TW]

Search String

(#1 OR #2 OR #3 OR #4) AND

(#5 OR #6 OR #7) AND

(#8 OR #9 OR #10 OR #11 OR #12 OR #13) AND

(OR #14 OR #15 OR #16 OR #17 OR #18 OR #19 OR #20 OR #21 OR #22 OR #23 OR #24 OR #25 OR #26 OR #27 OR #28 OR #29 OR #30 OR #31 OR #32 OR #33 OR #34 OR #35)

S1 Supplementary material and supporting information. Supplemental material containing **Table 1.** Included randomized controlled trials and baseline patient characteristics, **Table 2.** Active and control treatment received in the randomized controlled trials, **Table 3.** Inconsistency analysis, **Table 4.** Heterogeneity and model fit, **Table 5.** Random effects metaregressions analyses, **League tables** with fixed and random effects models for all endpoints, and **Funnel plots** (comparison-adjusted) to assess publication bias.

S2 Supporting information. PRISMA checklist

Figure legends

Figure 1. PRISMA flowchart. Trial selection process according to the PRISMA statement.

Figure 2. Network of evidence. Straight black lines denote direct head-to-head randomized comparisons. Numbers refer to the number of RCTs with direct comparisons available for each link and the size of circles is proportional to the pooled sample size (patients) available for each treatment node.

Figure 3. Patient survival. Forest plots (random effects) of direct frequentist analyses (RevMan, Cochrane). Risk of bias assessment by the Cochrane Collaboration tool is presented as well.

Figure 4. Patient Survival network meta-analysis (Random effects forest plot). Different treatments are reported in order of efficacy ranking according to the SUCRA statistic. Black circles denote the posterior median and the black lines denote the associated 95% CrI. Numbers represent hazard ratios (HR) and 95% CrIs. The combination of TACE and ablation was found to be the most effective treatment (SUCRA 95%).

Figure 5. Survival model. Projected survival curves for each treatment were fitted with an exponential model up to 5 years. Conventional TACE was the most common comparator in the overall network of evidence and was used as the anchor treatment because it had the largest sample size. Absolute survival estimates of TACE at different time points were calculated with a standard random effects proportional model weighted by patient sample for each trial (black circles). Median patient survival (half-life) for each treatment was then calculated by combining the fitted

hazard rate (exponential decay constant) of the anchor treatment with the pairwise posterior median HR calculated by the Bayesian model for the respective treatment.

Figure 6. Objective Response. Forest plots (random effects) of direct frequentist analyses of patient survival (RevMan, by Cochrane). Risk of bias assessment by the Cochrane Collaboration tool is presented as well.

Figure 7. Objective Response network meta-analysis (Random effects forest plot). Different treatments are reported in order of efficacy ranking according to the SUCRA statistic. Black circles denote the posterior median and the black lines denote the associated 95% CrI. Numbers represent odds ratios (OR) and 95% CrIs. The combination of TACE and ablation was found to be the most effective treatment (SUCRA 99%).

Figure 8. Patient survival and objective response. Two-dimensional ranking of different treatments according to patient survival (y-axis) and objective response (x-axis) based on the cumulative rank probabilities (SUCRA; %). Note the linear correlation (linear regression fit R^2=0.926) between the 2 outcome metrics.

Figure 9. Serious adverse events. Forest plots (random effects) of direct frequentist analyses of patient survival (RevMan, Cochrane). Risk of bias assessment by the Cochrane Collaboration tool is presented as well.

Figure 10. Serious Adverse Events network meta-analysis (Random effects forest plot). Different treatments are reported in order of safety ranking according to the SUCRA statistic. Black circles denote the posterior median and the black lines denote the associated 95% CrI. Numbers represent odds ratios (OR) and 95% CrIs. TARE was found to be the safest treatment (SUCRA 90%).

Figure 11. Strength and quality of evidence. QoE was graded as recommended for network meta-analyses on the basis of clinical diversity (between-trial heterogeneity of patient characteristics and/or study design), indirectness (absence of direct randomized comparisons), and imprecision (we chose a threshold of information fraction <50%). Effective sample size n for each comparison is shown along with information fraction (IF; %) in parentheses (compared to n=560 for a hypothetical well-powered randomized study to detect a survival benefit of HR=0.70 at 2 years). Color-coded representation of QoE; very low (light gray), low (yellow), moderate (green). There were no cases of high QoE observed.

Table 1. Included randomized controlled trials and baseline patient demographics and index tumour characteristics									
**Study & citation**	**Year**	**Patients (n)**	**Age (years)**	**Male Gender (%)**	**Child-Pugh A/B (#Okuda)**	**PS (0/1) or KPS**	**Median stage**	**Multinodular or diffuse**	**Follow-up (years)**
**Conventional transarterial chemoembolization (TACE) versus best supportive treatment (BST) [n=8]**									
Groupe d'Etude [62]	1995	96	64y	96%	100% / 0%	NA	NA	59%	4 years
Madden et al.[66]	1993	50	49y	92%	14% / 68%#	1 (1-3)	Okuda II	NA	5 months
Pelletier et al.[68]	1990	42	65y	88%	26% / 52%#	NA	Okuda II	NA	1 year
Pelletier et al.[67]	1998	73	66y	85%	77% / 23%	58% / 38%	Okuda I	NA	2 years
Lo et al.[64]	2002	79	63y	80%	47%/ 53%#	43% / 44%	Okuda II	60%	3.5 years
Llovet et al.[61] (3-arm)*	2002	75	65y	73%	69% / 31%	83% / 10%	BCLC B	72%	4 years
FFCD 9402 et al.[63]	2008	123	64y	87%	71% / 29%	37% / 47%	Okuda I	70%	5 years
Mabed et al.[65]	2009	100	52y	65%	69% / 31%	1 (0-2)	Okuda I	58%	1 year
**Bland transarterial embolization (TAE) versus best supportive treatment (BST) [n=3]**									
Lin et al.[111]	1988	63	50y	92%	100% (A/B)	NA	NA	NA	2 years
Bruix et al.[110]	1998	80	63y	75%	68% / 32%#	68% / 27%	Okuda I	76%	4 years
Llovet et al.[61] (3-arm)*	2002	72	65y	73%	67% / 33%	76% / 16%	Okuda II	76%	4 years
**Transarterial radioembolization (TARE) versus best supportive treatment (BST) [n=1]**									
Raoul et al.[112]	1994	27	66y	96%	52% / 48%	NA	BCLC B	70%	1 year
**Transarterial radioembolization (TARE) versus conventional transarterial chemoembolization (TACE) [n=2]**									
Raoul et al.[77]	1997	129	65y	95%	75% / 23%	KPS≥70%	Okuda I	50%	4 years
Kolligs et al.[76]	2015	28	66y	86%	64% / 25%	79% / 21%	BCLC B	68%	2 years
Salem et al.[78]	2016	45	63y	73%	56% / 44%	NA	BCLC A	47%	2 years
**Drug-eluting beads chemoembolization (DEB-TACE) versus conventional transarterial chemoembolization (TACE) [n=4]**									
Lammer et al.[73]	2009	201	67y	87%	83% / 17%	77% / 23%	BCLC B	42%	6 months
Sacco et al.[74]	2011	67	70y	67%	81% / 19%	100% / 0%	BCLC A	34%	3.5 years

Malenstein et al.[75]	2011	30	62y	83%	93% / 7%	63% / 30%	BCLC B	63%	1 month
Golfieri et al.[72]	2014	177	69y	76%	86% / 24%	74% / 26%	BCLC B	54%	2 years
**Bland transarterial embolization (TAE) versus conventional transarterial chemoembolization (TACE) [n=4]**									
Chang et al.[69]	1994	46	64y	93%	65% / 35%	NA	NA	57%	2 years
Kawai et al.[70]	1991	286	62y	85%	73% / 24%	52% / 26%	NA	NA	3 years
Meyer et al.[9]	2013	86	63y	86%	83% / 17%	67% / 20%	BCLC B	67%	3 years
Yu et al.[71]	2014	90	65y	80%	81% / 19%	66% / 31%	BCLC B	52%	4 years
**Drug-eluting beads chemoembolization (DEB-TACE) versus bland transarterial embolization (TAE) [n=2]**									
Malagari et al.[108]	2010	84	70y	77%	58%/ 42%	64% / 36%	NA	38%	1 year
Brown et al.[107]	2016	101	67y	77%	85% / 15%	86% / 14%	BCLC B	60%	6 years
**Drug-eluting beads chemoembolization (DEB-TACE) versus transarterial radioembolization (TARE) [n=1]**									
Pitton et al.[109]	2015	24	71y	75%	79% / 21%	100% / 0%	BCLC B	96%	3 years
**Conventional transarterial chemoembolization (TACE) plus systemic therapy versus conventional transarterial chemoembolization (TACE) [n=8]**									
Sansonno et al.[84]	2012	80	73y	60%	100% / 0%	61% / 39%	NA	45%	21 months
Kudo et al.[81]	2011	458	70y	75%	100% / 0%	88% / 12%	NA	27%	3 years
Britten et al.[79]	2011	30	59y	50%	93% / 7%	80% / 20%	BCLC B	27%	5 years
Pinter et al.[83]	2015	32	61y	91%	69% / 31%	100% / 0%	BCLC B	59%	46 months
Wang et al.[85]	2015	125	55y	85%	85% / 15%	82% / NA	BCLC B	33%	40 months
Li et al.[82]	2009	216	48y	70%	91% / 9%	76% / NA	Okuda I	55%	3 years
Kudo et al.[17]	2014	502	58y	84%	94% / 5%	80% / 20%	BCLC B	65%	3 years
Inaba et al.[80]	2013	101	NA	81%	84% / 16%	93% / 7%	BCLC B	57%	3 years

**Drug-eluting beads chemoembolization (DEB-TACE) plus adjuvant systemic versus Drug-eluting beads chemoembolization (DEB-TACE) [n=1]**									
Lencioni et al.[16]	2016	307	64y	85%	100% / 0%	100% / 0%	BCLC B	100%	800 days
**Conventional transarterial chemoembolization (TACE) plus tumour ablation versus conventional transarterial chemoembolization (TACE) [n=9]**									
Yang et al. [93]	2008	35	58y	74%	60% / 29%	NA	NA	66%	2 years
Bartolozzi et al.[86]	1995	53	66y	77%	47% / 53%	NA	NA	40%	3 years
Becker et al.[87]	2005	52	64y	79%	75% / 25%	NA	Okuda I	37%	30 months
Wu et al.[90]	1998	102	55y	94%	78% / 17%	NA	NA	NA	3 years
Xu et al.[91]	2002	45	NA	NA	100% / 0%	NA	NA	0%	3 years
Yamamoto et al.[92]	1997	100	NA	87%	37% / 42%	NA	JIS II-IV	52%	3 years
Liu et al.[88]	2009	78	53y	NA	86% / 14%	NA	BCLC C	NA	2 years
Wang et al.[89]	2007	83	58y	80%	80% / 20%	NA	TNM III	NA	1 year
Zhao et al.[94]	2011	47	NA	NA	NA	NA	BCLC C	NA	3 years
Huang et al.[95]	2016	120	60y	77%	100% (A/B)	NA	BCLC B	0%	5 years
**Conventional transarterial chemoembolization (TACE) plus external radiotherapy versus conventional transarterial chemoembolization (TACE) [n=11]**									
Xue et al.[103]	1995	41	NA	NA	100% (A/B)	NA	TNM II	NA	1 year
Leng et al.[96]	2000	75	NA	NA	100% / 0%	KPS≥65%	TNM III	NA	3 years
Wang et al.[101]	2000	40	37y	92%	85% (A/B)	NA	TNM III	30%	5 years
Peng et al.[99]	2000	91	NA	NA	NA	NA	TNM II	NA	5 years
Li et al.[97]	2003	82	51y	NA	61% / 39%	NA	NA	NA	3 years
Zhao et al.[105]	2006	96	53y	63%	100% / 0%	KPS≥70%	TNM I	NA	3 years
Shang et al.[100]	2007	76	52y	NA	100% (A/B)	KPS≥70%	TNM I	NA	3 years
Xiao et al.[106]	2008	60	NA	NA	65% / 35%	KPS≥70%	TNM II	NA	3 years

Liao et al.[98]	2010	48	NA	NA	71% / 29%	NA	TNM III	NA	3 years
Wang et al.[102]	2006	108	54y	NA	100% (A/B)	KPS≥65%	TNM III	8%	3 years
Zhang et al.[104]	2012	259	53y	NA	100% (A/B)	NA	BCLC C	NA	2 years

References

1. Bruix J, Sherman M, Practice Guidelines Committee AAftSoLD (2005) Management of hepatocellular carcinoma. Hepatology 42: 1208-1236.
2. de Baere T, Arai Y, Lencioni R, Geschwind JF, Rilling W, et al. (2016) Treatment of Liver Tumors with Lipiodol TACE: Technical Recommendations from Experts Opinion. Cardiovasc Intervent Radiol 39: 334-343.
3. Llovet JM, Zucman-Rossi J, Pikarsky E, Sangro B, Schwartz M, et al. (2016) Hepatocellular carcinoma. Nat Rev Dis Primers 2: 16018.
4. Bruix J, Reig M, Sherman M (2016) Evidence-Based Diagnosis, Staging, and Treatment of Patients With Hepatocellular Carcinoma. Gastroenterology 150: 835-853.
5. Mathurin P, Rixe O, Carbonell N, Bernard B, Cluzel P, et al. (1998) Review article: Overview of medical treatments in unresectable hepatocellular carcinoma--an impossible meta-analysis? Aliment Pharmacol Ther 12: 111-126.
6. Delicque J, Boulin M, Guiu B, Pelage JP, Escal L, et al. (2016) Interventional oncology for hepatocellular carcinoma. Clin Res Hepatol Gastroenterol.
7. Jansen MC, van Hillegersberg R, Chamuleau RA, van Delden OM, Gouma DJ, et al. (2005) Outcome of regional and local ablative therapies for hepatocellular carcinoma: a collective review. Eur J Surg Oncol 31: 331-347.
8. Lencioni R, de Baere T, Soulen MC, Rilling WS, Geschwind JH (2016) Lipiodol transarterial chemoembolization for hepatocellular carcinoma: A systematic review of efficacy and safety data. Hepatology.
9. Meyer T, Kirkwood A, Roughton M, Beare S, Tsochatzis E, et al. (2013) A randomised phase II/III trial of 3-weekly cisplatin-based sequential

transarterial chemoembolisation vs embolisation alone for hepatocellular carcinoma. Br J Cancer 108: 1252-1259.

10. Li L, Tian J, Liu P, Wang X, Zhu Z (2016) Transarterial chemoembolization combination therapy vs monotherapy in unresectable hepatocellular carcinoma: a meta-analysis. Tumori: 0.
11. Ray CE, Jr., Brown AC, Green TJ, Winston H, Curran C, et al. (2015) Survival outcomes in patients with advanced hepatocellular carcinoma treated with drug-eluting bead chemoembolization. AJR Am J Roentgenol 204: 440-447.
12. Fu Y, Zhao X, Yun Q, Zhu X, Zhu Y, et al. (2015) Transarterial chemoembolization (TACE) plus percutaneous ethanol injection (PEI) for the treatment of unresectable hepatocellular carcinoma: a meta-analysis of randomized controlled trials. Int J Clin Exp Med 8: 10388-10400.
13. Facciorusso A, Di Maso M, Muscatiello N (2016) Microwave ablation versus radiofrequency ablation for the treatment of hepatocellular carcinoma: A systematic review and meta-analysis. Int J Hyperthermia: 1-6.
14. Kallini JR, Gabr A, Salem R, Lewandowski RJ (2016) Transarterial Radioembolization with Yttrium-90 for the Treatment of Hepatocellular Carcinoma. Adv Ther.
15. Vente MA, Wondergem M, van der Tweel I, van den Bosch MA, Zonnenberg BA, et al. (2009) Yttrium-90 microsphere radioembolization for the treatment of liver malignancies: a structured meta-analysis. Eur Radiol 19: 951-959.
16. Lencioni R, Llovet JM, Han G, Tak WY, Yang J, et al. (2016) Sorafenib or placebo plus TACE with doxorubicin-eluting beads for intermediate stage HCC: The SPACE trial. J Hepatol 64: 1090-1098.

17. Kudo M, Han G, Finn RS, Poon RT, Blanc JF, et al. (2014) Brivanib as adjuvant therapy to transarterial chemoembolization in patients with hepatocellular carcinoma: A randomized phase III trial. Hepatology 60: 1697-1707.
18. Huo YR, Eslick GD (2015) Transcatheter Arterial Chemoembolization Plus Radiotherapy Compared With Chemoembolization Alone for Hepatocellular Carcinoma: A Systematic Review and Meta-analysis. JAMA Oncol 1: 756-765.
19. Facciorusso A, Di Maso M, Muscatiello N (2016) Drug-eluting beads versus conventional chemoembolization for the treatment of unresectable hepatocellular carcinoma: A meta-analysis. Dig Liver Dis.
20. Katsanos K, Spiliopoulos S, Karunanithy N, Krokidis M, Sabharwal T, et al. (2014) Bayesian network meta-analysis of nitinol stents, covered stents, drug-eluting stents, and drug-coated balloons in the femoropopliteal artery. J Vasc Surg 59: 1123-1133 e1128.
21. Katsanos K, Spiliopoulos S, Saha P, Diamantopoulos A, Karunanithy N, et al. (2015) Comparative Efficacy and Safety of Different Antiplatelet Agents for Prevention of Major Cardiovascular Events and Leg Amputations in Patients with Peripheral Arterial Disease: A Systematic Review and Network Meta-Analysis. PLoS One 10: e0135692.
22. Ni JY, Liu SS, Xu LF, Sun HL, Chen YT (2013) Transarterial chemoembolization combined with percutaneous radiofrequency ablation versus TACE and PRFA monotherapy in the treatment for hepatocellular carcinoma: a meta-analysis. J Cancer Res Clin Oncol 139: 653-659.
23. Bhardwaj N, Strickland AD, Ahmad F, Dennison AR, Lloyd DM (2010) Liver ablation techniques: a review. Surg Endosc 24: 254-265.

24. Xie ZB, Wang XB, Peng YC, Zhu SL, Ma L, et al. (2015) Systematic review comparing the safety and efficacy of conventional and drug-eluting bead transarterial chemoembolization for inoperable hepatocellular carcinoma. Hepatol Res 45: 190-200.

25. Ni JY, Xu LF, Wang WD, Sun HL, Chen YT (2014) Conventional transarterial chemoembolization vs microsphere embolization in hepatocellular carcinoma: a meta-analysis. World J Gastroenterol 20: 17206-17217.

26. Han S, Zhang X, Zou L, Lu C, Zhang J, et al. (2014) Does drug-eluting bead transcatheter arterial chemoembolization improve the management of patients with hepatocellular carcinoma? A meta-analysis. PLoS One 9: e102686.

27. Camma C, Schepis F, Orlando A, Albanese M, Shahied L, et al. (2002) Transarterial chemoembolization for unresectable hepatocellular carcinoma: meta-analysis of randomized controlled trials. Radiology 224: 47-54.

28. Liao M, Huang J, Zhang T, Wu H (2013) Transarterial chemoembolization in combination with local therapies for hepatocellular carcinoma: a meta-analysis. PLoS One 8: e68453.

29. Liu L, Chen H, Wang M, Zhao Y, Cai G, et al. (2014) Combination therapy of sorafenib and TACE for unresectable HCC: a systematic review and meta-analysis. PLoS One 9: e91124.

30. Meng MB, Cui YL, Lu Y, She B, Chen Y, et al. (2009) Transcatheter arterial chemoembolization in combination with radiotherapy for unresectable hepatocellular carcinoma: a systematic review and meta-analysis. Radiother Oncol 92: 184-194.

31. Yang M, Yuan JQ, Bai M, Han GH (2014) Transarterial chemoembolization combined with sorafenib for unresectable hepatocellular carcinoma: a systematic review and meta-analysis. Mol Biol Rep 41: 6575-6582.

32. Zou LQ, Zhang BL, Chang Q, Zhu FP, Li YY, et al. (2014) 3D conformal radiotherapy combined with transcatheter arterial chemoembolization for hepatocellular carcinoma. World J Gastroenterol 20: 17227-17234.

33. Oliveri RS, Wetterslev J, Gluud C (2011) Transarterial (chemo)embolisation for unresectable hepatocellular carcinoma. Cochrane Database Syst Rev: CD004787.

34. Moher D, Liberati A, Tetzlaff J, Altman DG, Group P (2009) Preferred reporting items for systematic reviews and meta-analyses: the PRISMA statement. BMJ 339: b2535.

35. Gaba RC, Lewandowski RJ, Hickey R, Baerlocher MO, Cohen EI, et al. (2016) Transcatheter Therapy for Hepatic Malignancy: Standardization of Terminology and Reporting Criteria. J Vasc Interv Radiol 27: 457-473.

36. Higgins JP, Altman DG, Gotzsche PC, Juni P, Moher D, et al. (2011) The Cochrane Collaboration's tool for assessing risk of bias in randomised trials. BMJ 343: d5928.

37. Jansen JP, Trikalinos T, Cappelleri JC, Daw J, Andes S, et al. (2014) Indirect treatment comparison/network meta-analysis study questionnaire to assess relevance and credibility to inform health care decision making: an ISPOR-AMCP-NPC Good Practice Task Force report. Value Health 17: 157-173.

38. Guyatt GH, Oxman AD, Vist GE, Kunz R, Falck-Ytter Y, et al. (2008) GRADE: an emerging consensus on rating quality of evidence and strength of recommendations. BMJ 336: 924-926.

39. Puhan MA, Schunemann HJ, Murad MH, Li T, Brignardello-Petersen R, et al. (2014) A GRADE Working Group approach for rating the quality of treatment effect estimates from network meta-analysis. BMJ 349: g5630.

40. Woods BS, Hawkins N, Scott DA (2010) Network meta-analysis on the log-hazard scale, combining count and hazard ratio statistics accounting for multi-arm trials: a tutorial. BMC Med Res Methodol 10: 54.

41. Hirooka T, Hamada C, Yoshimura I (2009) A note on estimating treatment effect for time-to-event data in a literature-based meta-analysis. Methods Inf Med 48: 104-112.

42. Parmar MK, Torri V, Stewart L (1998) Extracting summary statistics to perform meta-analyses of the published literature for survival endpoints. Stat Med 17: 2815-2834.

43. Tierney JF, Stewart LA, Ghersi D, Burdett S, Sydes MR (2007) Practical methods for incorporating summary time-to-event data into meta-analysis. Trials 8: 16.

44. (1979) World Health Organization. WHO handbook for reporting results of cancer treatment. Available at: http://appswhoint/iris/bitstream/10665/37200/1/WHO_OFFSET_48pdf Accessed 13 May 2016.

45. Bruix J, Sherman M, Llovet JM, Beaugrand M, Lencioni R, et al. (2001) Clinical management of hepatocellular carcinoma. Conclusions of the Barcelona-2000 EASL conference. European Association for the Study of the Liver. J Hepatol 35: 421-430.

46. Eisenhauer EA, Therasse P, Bogaerts J, Schwartz LH, Sargent D, et al. (2009) New response evaluation criteria in solid tumours: revised RECIST guideline (version 1.1). Eur J Cancer 45: 228-247.

47. Lencioni R, Llovet JM (2010) Modified RECIST (mRECIST) assessment for hepatocellular carcinoma. Semin Liver Dis 30: 52-60.

48. (2010) US Department of Health and Human Services; National Institutes of Health; National Cancer Institute. Common terminology criteria for adverse events (CTCAE). Version 4.0. Available at: http://evsncinihgov/ftp1/CTCAE/CTCAE_403_2010-06-14_QuickReference_85x11pdf Accessed 13 May 2016.

49. Dias S, Welton NJ, Sutton AJ, Caldwell DM, Lu G, et al. (2013) Evidence synthesis for decision making 4: inconsistency in networks of evidence based on randomized controlled trials. Med Decis Making 33: 641-656.

50. Dias S, Sutton AJ, Welton NJ, Ades AE (2013) Evidence synthesis for decision making 3: heterogeneity--subgroups, meta-regression, bias, and bias-adjustment. Med Decis Making 33: 618-640.

51. Dias S, Sutton AJ, Ades AE, Welton NJ (2013) Evidence synthesis for decision making 2: a generalized linear modeling framework for pairwise and network meta-analysis of randomized controlled trials. Med Decis Making 33: 607-617.

52. Salanti G, Ades AE, Ioannidis JP (2011) Graphical methods and numerical summaries for presenting results from multiple-treatment meta-analysis: an overview and tutorial. J Clin Epidemiol 64: 163-171.

53. Wetterslev J, Thorlund K, Brok J, Gluud C (2009) Estimating required information size by quantifying diversity in random-effects model meta-analyses. BMC Med Res Methodol 9: 86.

54. Brok J, Thorlund K, Gluud C, Wetterslev J (2008) Trial sequential analysis reveals insufficient information size and potentially false positive results in many meta-analyses. J Clin Epidemiol 61: 763-769.

55. Thorlund K, Mills EJ (2012) Sample size and power considerations in network meta-analysis. Syst Rev 1: 41.

56. Chaimani A, Higgins JP, Mavridis D, Spyridonos P, Salanti G (2013) Graphical tools for network meta-analysis in STATA. PLoS One 8: e76654.

57. Achana FA, Cooper NJ, Dias S, Lu G, Rice SJ, et al. (2013) Extending methods for investigating the relationship between treatment effect and baseline risk from pairwise meta-analysis to network meta-analysis. Stat Med 32: 752-771.

58. Jansen JP, Naci H (2013) Is network meta-analysis as valid as standard pairwise meta-analysis? It all depends on the distribution of effect modifiers. BMC Med 11: 159.

59. Dias S, Welton NJ, Caldwell DM, Ades AE (2010) Checking consistency in mixed treatment comparison meta-analysis. Stat Med 29: 932-944.

60. Brown S, Hutton B, Clifford T, Coyle D, Grima D, et al. (2014) A Microsoft-Excel-based tool for running and critically appraising network meta-analyses--an overview and application of NetMetaXL. Syst Rev 3: 110.

61. Llovet JM, Real MI, Montana X, Planas R, Coll S, et al. (2002) Arterial embolisation or chemoembolisation versus symptomatic treatment in patients with unresectable hepatocellular carcinoma: a randomised controlled trial. Lancet 359: 1734-1739.

62. (1995) A comparison of lipiodol chemoembolization and conservative treatment for unresectable hepatocellular carcinoma. Groupe d'Etude et de Traitement du Carcinome Hepatocellulaire. N Engl J Med 332: 1256-1261.

63. Doffoel M, Bonnetain F, Bouche O, Vetter D, Abergel A, et al. (2008) Multicentre randomised phase III trial comparing Tamoxifen alone or with Transarterial Lipiodol Chemoembolisation for unresectable hepatocellular carcinoma in cirrhotic patients (Federation Francophone de Cancerologie Digestive 9402). Eur J Cancer 44: 528-538.

64. Lo CM, Ngan H, Tso WK, Liu CL, Lam CM, et al. (2002) Randomized controlled trial of transarterial lipiodol chemoembolization for unresectable hepatocellular carcinoma. Hepatology 35: 1164-1171.

65. Mabed M, Esmaeel M, El-Khodary T, Awad M, Amer T (2009) A randomized controlled trial of transcatheter arterial chemoembolization with lipiodol, doxorubicin and cisplatin versus intravenous doxorubicin for patients with unresectable hepatocellular carcinoma. Eur J Cancer Care (Engl) 18: 492-499.

66. Madden MV, Krige JE, Bailey S, Beningfield SJ, Geddes C, et al. (1993) Randomised trial of targeted chemotherapy with lipiodol and 5-epidoxorubicin compared with symptomatic treatment for hepatoma. Gut 34: 1598-1600.

67. Pelletier G, Ducreux M, Gay F, Luboinski M, Hagege H, et al. (1998) Treatment of unresectable hepatocellular carcinoma with lipiodol chemoembolization: a multicenter randomized trial. Groupe CHC. J Hepatol 29: 129-134.

68. Pelletier G, Roche A, Ink O, Anciaux ML, Derhy S, et al. (1990) A randomized trial of hepatic arterial chemoembolization in patients with unresectable hepatocellular carcinoma. J Hepatol 11: 181-184.

69. Chang JM, Tzeng WS, Pan HB, Yang CF, Lai KH (1994) Transcatheter arterial embolization with or without cisplatin treatment of hepatocellular carcinoma. A randomized controlled study. Cancer 74: 2449-2453.

70. Kawai S, Okamura J, Ogawa M, Ohashi Y, Tani M, et al. (1992) Prospective and randomized clinical trial for the treatment of hepatocellular carcinoma--a comparison of lipiodol-transcatheter arterial embolization with and without adriamycin (first cooperative study). The Cooperative Study Group for Liver Cancer Treatment of Japan. Cancer Chemother Pharmacol 31 Suppl: S1-6.

71. Yu SC, Hui JW, Hui EP, Chan SL, Lee KF, et al. (2014) Unresectable hepatocellular carcinoma: randomized controlled trial of transarterial ethanol ablation versus transcatheter arterial chemoembolization. Radiology 270: 607-620.

72. Golfieri R, Giampalma E, Renzulli M, Cioni R, Bargellini I, et al. (2014) Randomised controlled trial of doxorubicin-eluting beads vs conventional chemoembolisation for hepatocellular carcinoma. Br J Cancer 111: 255-264.

73. Lammer J, Malagari K, Vogl T, Pilleul F, Denys A, et al. (2010) Prospective randomized study of doxorubicin-eluting-bead embolization in the treatment of hepatocellular carcinoma: results of the PRECISION V study. Cardiovasc Intervent Radiol 33: 41-52.

74. Sacco R, Bargellini I, Bertini M, Bozzi E, Romano A, et al. (2011) Conventional versus doxorubicin-eluting bead transarterial chemoembolization for hepatocellular carcinoma. J Vasc Interv Radiol 22: 1545-1552.

75. van Malenstein H, Maleux G, Vandecaveye V, Heye S, Laleman W, et al. (2011) A randomized phase II study of drug-eluting beads versus transarterial chemoembolization for unresectable hepatocellular carcinoma. Onkologie 34: 368-376.

76. Kolligs FT, Bilbao JI, Jakobs T, Inarrairaegui M, Nagel JM, et al. (2015) Pilot randomized trial of selective internal radiation therapy vs. chemoembolization in unresectable hepatocellular carcinoma. Liver Int 35: 1715-1721.

77. Raoul JL, Guyader D, Bretagne JF, Heautot JF, Duvauferrier R, et al. (1997) Prospective randomized trial of chemoembolization versus intra-arterial injection of 131I-labeled-iodized oil in the treatment of hepatocellular carcinoma. Hepatology 26: 1156-1161.

78. Salem R, Gordon AC, Mouli S, Hickey R, Kallini J, et al. (2016) Y90 Radioembolization Significantly Prolongs Time to Progression Compared With Chemoembolization in Patients With Hepatocellular Carcinoma. Gastroenterology 151: 1155-1163 e1152.

79. Britten CD, Gomes AS, Wainberg ZA, Elashoff D, Amado R, et al. (2012) Transarterial chemoembolization plus or minus intravenous bevacizumab in the treatment of hepatocellular cancer: a pilot study. BMC Cancer 12: 16.

80. Inaba Y, Kanai F, Aramaki T, Yamamoto T, Tanaka T, et al. (2013) A randomised phase II study of TSU-68 in patients with hepatocellular carcinoma treated by transarterial chemoembolisation. Eur J Cancer 49: 2832-2840.

81. Kudo M, Imanaka K, Chida N, Nakachi K, Tak WY, et al. (2011) Phase III study of sorafenib after transarterial chemoembolisation in Japanese and Korean patients with unresectable hepatocellular carcinoma. Eur J Cancer 47: 2117-2127.

82. Li M, Lu C, Cheng J, Zhang J, Cao C, et al. (2009) Combination therapy with transarterial chemoembolization and interferon-alpha compared with transarterial chemoembolization alone for hepatitis B virus related

unresectable hepatocellular carcinoma. J Gastroenterol Hepatol 24: 1437-1444.

83. Pinter M, Ulbrich G, Sieghart W, Kolblinger C, Reiberger T, et al. (2015) Hepatocellular Carcinoma: A Phase II Randomized Controlled Double-Blind Trial of Transarterial Chemoembolization in Combination with Biweekly Intravenous Administration of Bevacizumab or a Placebo. Radiology 277: 903-912.
84. Sansonno D, Lauletta G, Russi S, Conteduca V, Sansonno L, et al. (2012) Transarterial chemoembolization plus sorafenib: a sequential therapeutic scheme for HCV-related intermediate-stage hepatocellular carcinoma: a randomized clinical trial. Oncologist 17: 359-366.
85. Wang H, Liu Y, Wang X, Liu D, Sun Z, et al. (2015) Randomized clinical control study of locoregional therapy combined with arsenic trioxide for the treatment of hepatocellular carcinoma. Cancer 121: 2917-2925.
86. Bartolozzi C, Lencioni R, Caramella D, Vignali C, Cioni R, et al. (1995) Treatment of large HCC: transcatheter arterial chemoembolization combined with percutaneous ethanol injection versus repeated transcatheter arterial chemoembolization. Radiology 197: 812-818.
87. Becker G, Soezgen T, Olschewski M, Laubenberger J, Blum HE, et al. (2005) Combined TACE and PEI for palliative treatment of unresectable hepatocellular carcinoma. World J Gastroenterol 11: 6104-6109.
88. Liu DX, Li HD, Li XF, al. e (2009) Evaluation of TACE combined with RFA and PEI in treating advanced hepatic carcinoma. J Intervent Radiol 18: 389-393.
89. Wang YB, Chen MH, Yan K, Yang W, Dai Y, et al. (2007) Quality of life after radiofrequency ablation combined with transcatheter arterial

chemoembolization for hepatocellular carcinoma: comparison with transcatheter arterial chemoembolization alone. Qual Life Res 16: 389-397.

90. Wu PH, Li L, Zhang YM (1998) Transcatheter arterial chemoembolization combined with CT-guided percutaneous intratumoral injection of lipiodol-ethanol for the treatment of hepatocellular carcinoma. Chin J Oncol 20: 391-393.
91. Xu GH, Wen HC, Li ZW, al. e (2002) Evaluation of hepatic chemoembolization and percutaneous ethanol injection in the treatment of HCC. Chin J Radiology 1: 66-68.
92. Yamamoto K, Masuzawa M, Kato M, Kurosawa K, Kaneko A, et al. (1997) Evaluation of combined therapy with chemoembolization and ethanol injection for advanced hepatocellular carcinoma. Semin Oncol 24: S6-50-S56-55.
93. Yang P, Liang M, Zhang Y, Shen B (2008) Clinical application of a combination therapy of lentinan, multi-electrode RFA and TACE in HCC. Adv Ther 25: 787-794.
94. Zhao M, Wang JP, Li W, Huang ZL, Zhang FJ, et al. (2011) [Comparison of safety and efficacy for transcatheter arterial chemoembolization alone and plus radiofrequency ablation in the treatment of single branch portal vein tumor thrombus of hepatocellular carcinoma and their prognosis factors]. Zhonghua Yi Xue Za Zhi 91: 1167-1172.
95. Huang C, Zhuang W, Feng H, Guo H, Tang Y, et al. (2016) Analysis of therapeutic effectiveness and prognostic factor on argon-helium cryoablation combined with transcatheter arterial chemoembolization for the treatment of advanced hepatocellular carcinoma. J Cancer Res Ther 12: C148-C152.

96. Leng ZQ, Liang ZY, Shi S, Hu ZX (2000) Comparison of treatment results of interventional therapy alone, radiotherapy alone, and combined interventional therapy plus radiotherapy for primary hepatic cancer. Chin J Radiat Oncol 9: 99-101.

97. Li Y, Yan Y, Zhang HB, Guo ZW, Yan ZC, et al. (2003) Three-dimensional conformal radiation combined with transarterial chemoembolization for unresectable primary liver cancer. Chin J Radiat Oncol 12: 30-32.

98. Liao XF, He HJ, Zhou ZS, Hu W, Zhu XP (2010) Three-dimensional conformal radiotherapy combined with interventional therapy in treatment of primary hepatocellular carcinoma. J Prac Oncol 25: 681-683.

99. Peng KG, Han FS, Liu H, Song MZ (2000) Clinical study of unresectable liver cancer treated by intraoperative hepatic arterial embolization and post-operative hyperfractionation radiotherapy. Chin J Radiat Oncol 9: 11-13.

100. Shang Y, You GX, Xu HY, Chen MC (2007) Prospective randomized clinical study of transcatheter arterial chemoembolization, combined with three-dimensional conformal radiotherapy for primary liver cancer: An analysis of 40 cases. Shijie Huaren Xiaohua Zazhi 15: 3140-3142.

101. Wang G, Shen W, Song M, Xu H (2000) Results of combined treatment with transcatheter hepatic arterial chemoembolization and whole-liver irradiation with the moving strip technique in unresectable hepatocellular carcinoma. . Int J Clin Oncol 5: 380-385.

102. Wang XH, Li JX, Gao K (2006) Radiotherapy combined with hepatic cheoembolization in the treatment of 54 primary liver cancer. Shanxi Medical Journal 35: 461-462.

103. Xue HZ, Meng GD, Wang YW, Jiang QF (1995) Transcatheter arterial chemoembolization plus radiotherapy in the treatment of hepatocellular carcinoma. Chin J Radiat Oncol 4: 84-85.

104. Zhang Z, Yang X, Wena M, Wan J (2012) Evaluation of TACE combined with gamma—knife radiotherapy for primary hepatocellular carcinoma. J Intervent Radiol (China) 7: 596-599.

105. Zhao MH, Lang FP, Jiang QA, Ma JJ, Song YX (2006) Three-dimensional conformal radiotherapy combined with transcatheter arterial chemoembolization for inoperable primary liver cancer. Chin J Radiat Oncol 15: 39-41.

106. Xiao Z, Ouyang T, Yu R, Jiang X, Reng H, et al. (2008) Transcatheter arterial chemoembolization combined with 3-dimensional conformal radiotherapy for patients with unresectable primary hepatic carcinoma. Chinese Journal of Clinical Oncology 35: 18-21.

107. Brown KT, Do RK, Gonen M, Covey AM, Getrajdman GI, et al. (2016) Randomized Trial of Hepatic Artery Embolization for Hepatocellular Carcinoma Using Doxorubicin-Eluting Microspheres Compared With Embolization With Microspheres Alone. J Clin Oncol.

108. Malagari K, Pomoni M, Kelekis A, Pomoni A, Dourakis S, et al. (2010) Prospective randomized comparison of chemoembolization with doxorubicin-eluting beads and bland embolization with BeadBlock for hepatocellular carcinoma. Cardiovasc Intervent Radiol 33: 541-551.

109. Pitton MB, Kloeckner R, Ruckes C, Wirth GM, Eichhorn W, et al. (2015) Randomized comparison of selective internal radiotherapy (SIRT) versus drug-eluting bead transarterial chemoembolization (DEB-TACE) for the

treatment of hepatocellular carcinoma. Cardiovasc Intervent Radiol 38: 352-360.

110. Bruix J, Llovet JM, Castells A, Montana X, Bru C, et al. (1998) Transarterial embolization versus symptomatic treatment in patients with advanced hepatocellular carcinoma: results of a randomized, controlled trial in a single institution. Hepatology 27: 1578-1583.

111. Lin DY, Liaw YF, Lee TY, Lai CM (1988) Hepatic arterial embolization in patients with unresectable hepatocellular carcinoma--a randomized controlled trial. Gastroenterology 94: 453-456.

112. Raoul JL, Guyader D, Bretagne JF, Duvauferrier R, Bourguet P, et al. (1994) Randomized controlled trial for hepatocellular carcinoma with portal vein thrombosis: intra-arterial iodine-131-iodized oil versus medical support. J Nucl Med 35: 1782-1787.

113. Puhan MA, Schunemann HJ, Murad MH, Li T, Brignardello-Petersen R, et al. (2014) A GRADE Working Group approach for rating the quality of treatment effect estimates from network meta-analysis. BMJ 349: g5630.

114. Lan T, Chang L, Mn R, Wu L, Yuan YF (2016) Comparative Efficacy of Interventional Therapies for Early-stage Hepatocellular Carcinoma: A PRISMA-compliant Systematic Review and Network Meta-analysis. Medicine (Baltimore) 95: e3185.

115. Marrero JA, Kudo M, Venook AP, Ye SL, Bronowicki JP, et al. (2016) Observational registry of sorafenib use in clinical practice across Child-Pugh subgroups: The GIDEON study. J Hepatol 65: 1140-1147.

116. Verslype C, Rosmorduc O, Rougier P, Group EGW (2012) Hepatocellular carcinoma: ESMO-ESDO Clinical Practice Guidelines for diagnosis, treatment and follow-up. Ann Oncol 23 Suppl 7: vii41-48.

117. Boily G, Villeneuve JP, Lacoursiere L, Chaudhury P, Couture F, et al. (2015) Transarterial embolization therapies for the treatment of hepatocellular carcinoma: CEPO review and clinical recommendations. HPB (Oxford) 17: 52-65.

118. Forner A, Llovet JM, Bruix J (2012) Chemoembolization for intermediate HCC: is there proof of survival benefit? J Hepatol 56: 984-986.

119. Park YN, Yang CP, Fernandez GJ, Cubukcu O, Thung SN, et al. (1998) Neoangiogenesis and sinusoidal "capillarization" in dysplastic nodules of the liver. Am J Surg Pathol 22: 656-662.

120. Rammohan A, Sathyanesan J, Ramaswami S, Lakshmanan A, Senthil-Kumar P, et al. (2012) Embolization of liver tumors: Past, present and future. World J Radiol 4: 405-412.

121. Wen L, Liang C, Chen E, Chen W, Liang F, et al. (2016) Regulation of Multi-drug Resistance in hepatocellular carcinoma cells is TRPC6/Calcium Dependent. Sci Rep 6: 23269.

122. Higuchi T, Kikuchi M, Okazaki M (1994) Hepatocellular carcinoma after transcatheter hepatic arterial embolization. A histopathologic study of 84 resected cases. Cancer 73: 2259-2267.

123. Kwan SW, Fidelman N, Ma E, Kerlan RK, Jr., Yao FY (2012) Imaging predictors of the response to transarterial chemoembolization in patients with hepatocellular carcinoma: a radiological-pathological correlation. Liver Transpl 18: 727-736.

124. Qi X, Zhao Y, Li H, Guo X, Han G (2016) Management of hepatocellular carcinoma: an overview of major findings from meta-analyses. Oncotarget 7: 34703-34751.

125. Llovet JM, Bruix J (2003) Systematic review of randomized trials for unresectable hepatocellular carcinoma: Chemoembolization improves survival. Hepatology 37: 429-442.

126. Marelli L, Stigliano R, Triantos C, Senzolo M, Cholongitas E, et al. (2007) Transarterial therapy for hepatocellular carcinoma: which technique is more effective? A systematic review of cohort and randomized studies. Cardiovasc Intervent Radiol 30: 6-25.

127. Facciorusso A, Bellanti F, Villani R, Salvatore V, Muscatiello N, et al. (2017) Transarterial chemoembolization vs bland embolization in hepatocellular carcinoma: A meta-analysis of randomized trials. United European Gastroenterol J 5: 511-518.

128. Gao S, Yang Z, Zheng Z, Yao J, Deng M, et al. (2013) Doxorubicin-eluting bead versus conventional TACE for unresectable hepatocellular carcinoma: a meta-analysis. Hepatogastroenterology 60: 813-820.

129. Huang K, Zhou Q, Wang R, Cheng D, Ma Y (2014) Doxorubicin-eluting beads versus conventional transarterial chemoembolization for the treatment of hepatocellular carcinoma. J Gastroenterol Hepatol 29: 920-925.

130. Zhang L, Hu P, Chen X, Bie P (2014) Transarterial chemoembolization (TACE) plus sorafenib versus TACE for intermediate or advanced stage hepatocellular carcinoma: a meta-analysis. PLoS One 9: e100305.

131. Cabibbo G, Enea M, Attanasio M, Bruix J, Craxi A, et al. (2010) A meta-analysis of survival rates of untreated patients in randomized clinical trials of hepatocellular carcinoma. Hepatology 51: 1274-1283.

132. Salem R, Mazzaferro V, Sangro B (2013) Yttrium 90 radioembolization for the treatment of hepatocellular carcinoma: biological lessons, current challenges, and clinical perspectives. Hepatology 58: 2188-2197.

To: Editor in Chief

PLoS ONE Editorial Office **London, August 19th, 2017**

Dear Editor,

We would like to thank you and the expert referees once again for the time and effort spent and their interesting comments and constructive criticisms of our manuscript entitled: **"Comparative effectiveness of different transarterial embolization therapies alone or in combination with local ablative or adjuvant systemic treatments for unresectable hepatocellular carcinoma: A network meta-analysis of randomized controlled trials"** that was submitted for consideration for publication in the journal of **PLoS ONE**.

We have followed the comments of the referees and we hope that we have addressed their questions adequately. We apologise for the delay in submitting our revision as we had to **include another 2 RCTs (Salem et al. 2017 and Huang et al. 2017)** and hence, we had to re-run all analyses and revise all numerical results accordingly (minor decimal differences). Please find attached a **point-by-point** list of all the changes and revisions made. We also attach separately an annotated red-lined text file with numbered lines where you can refer for each revision made.

We believe that the present paper may be of particular interest and value for the average PLoS ONE reader as it shows that **(1) transcatheter arterial embolization therapies actually improve patient survival over control medical treatment** by reducing the hazard of death in the range of 24% (in case of chemoembolization) to 34% (in case of bland transarterial embolization) or 43% in case of radioembolization, **(2)** Transcatheter chemo- and radio-embolization monotherapies, or even combined with systemic chemotherapy, **are not more effective than plain bland particle transarterial embolization**, and **(3) Chemoembolization combined with external radiotherapy or local liver ablation may significantly prolong patient survival** over transarterial embolization monotherapies by **12-15 months extra median survival time**. Therefore, the current trends of chemoembolization for unresectable HCC are clearly open to question and international guidelines may need to be revised.

All authors have made significant contributions to the submitted work and have approved the final version of the manuscript.

In addition, the authors certify that:

(1) There has been no duplicate publication or submission of any part of the work elsewhere,

(2) None of the paper's contents have been previously published

(3) There is no financial arrangement or other relationship with the industry that could be construed as a conflict of interest.

Looking forward to hearing from you,

We thank you in advance,

Yours sincerely,

On behalf of the authors

Dr. K. Katsanos

Editor:

1. Please ensure that your manuscript meets PLOS ONE's style requirements, including those for file naming. The PLOS ONE style templates can be found online

Authors' response: We have followed the PLOS ONE's style requirements and have revised the whole manuscript and appended files according to the relevant style template available online.

2. Please present the full electronic search strategy for at least one database, including any limits used, such that it could be repeated.

Authors' response: We have inserted an example of our electronic search at the end of the manuscript as follows:

Lines 708-727:

Search strategy

1 "hepatocellular carcinoma"[MESH], 2 "hepatocellular carcinoma"[TW], 3 "liver cancer"[MESH], 4 "liver cancer"[TW]

5 "unresectable"[TW], 6 "inoperable"[TW], 7 "advanced"[TW]

8 "Clinical trial"[Mesh], 9 "Randomized Controlled Trial"[Mesh], 10 "Clinical trial"[TW], 11 "Randomized"[TW], 12 "Meta-analysis"[Mesh], 13 "Meta-analysis"[TW]

14 "embolization"[MESH], 15 "chemoembolization"[MESH], 16 "sorafenib"[MESH], 17 "embolization"[TW], 18 "chemoembolization"[TW],19 "sorafenib"[TW], 20 "transcatheter" [TW], 21 "ablation"[TW], 22 "radiotherapy"[TW], 23 "radiation"[TW], 24 "radioembolization"[TW], 25 "selective internal radiation therapy"[TW], 26 "radiofrequency"[TW], 27 "alcohol"[TW], 28 "drug-eluting"[TW], 29 "anti-angiog*"[TW], "bevazicumab"[TW], 30 "TACE"[TW], 31 "TAE"[TW], 32 "DEB-TACE", 33 "TAE"[TW], 34 "SIRT"[TW], 35 "TARE"[TW]

Search String

(#1 OR #2 OR #3 OR #4) AND

(#5 OR #6 OR #7) AND

(#8 OR #9 OR #10 OR #11 OR #12 OR #13) AND

(OR #14 OR #15 OR #16 OR #17 OR #18 OR #19 OR #20 OR #21 OR #22 OR #23 OR #24 OR #25 OR #26 OR #27 OR #28 OR #29 OR #30 OR #31 OR #32 OR #33 OR #34 OR #35)

3. Please state in the methods section who conducted the search, data extraction, and risk bias assessment.

Authors' response: We have provided the requested information as follows:

Line 150: KK, PK and SS performed the literature search and data extraction.

Line 169: A standardized data extraction form was used to collect the following information from all included trials (by KK, PK and SS):

Line 187: Risk of bias assessment was performed by KK, SS and DK.

4. Please assess the publication bias using statistical methods (in addition to funnel plots)

Authors' response: We have provided basic and comparison-adjusted funnel plots in the supporting supplemental material. In the case of network meta-analysis, comparison-adjusted funnel plots is the proposed method for evaluating potential publication bias; no formal statistical methods are currently available. *Please refer to Salanti G, Del Giovane C, Chaimani A, Caldwell DM, Higgins JP. Evaluating the quality of evidence from a network meta-analysis. PLoS One. 2014 Jul 3;9(7):e99682. doi: 10.1371/journal.pone.0099682.*

5. Please include captions for your Supporting Information files at the end of your manuscript beneath the references, and update any in-text citations to match accordingly. Please see our Supporting Information guidelines for more information: http://www.plosone.org/static/supportingInformation.

Authors' response: We have updated all relevant in-text citations and we have included a caption describing the supplementary supporting Information material (S1 Appendix) that reads as follows:

Lines 729-735: S1 Supplementary material and supporting information.
Supplemental material containing **Table 1.** Included randomized controlled trials and baseline patient characteristics, **Table 2.** Active and control treatment received in the randomized controlled trials, **Table 3.** Inconsistency analysis, **Table 4.** Heterogeneity and model fit, **Table 5.** Random effects metaregressions analyses, **League tables** with fixed and random effects models for all endpoints, and **Funnel plots** (adjusted) to assess publication bias.

Reviewer #1:

6. Obviously, the authors spent lots of time on the work. The issue discussed in this work is relatively broad. Several major revision comments should be addressed. The text was so long that the readers cannot easily catch the major findings.

Authors' response: We thank the reviewer for his time and efforts. We have addressed his concerns in detail further below. We understand that the text may appear too long, but this is necessary due to the complexity of the statistical analyses and the multiple endpoints (we have tried to present the results of direct frequentist and mixed Bayesian analyses in a succinct order for each endpoint). Considerable part of the results is available as a supplementary material. We also note that following the advice of the 2nd reviewer, we **included another 2 RCTs (Salem et al. 2017 and Huang et al. 2017)** and hence, we had to re-run all analyses and revise all numerical results accordingly (minor decimal differences – revised figures and Tables throughout the manuscript – results overall nearly identical).

7. Unfortunately, the authors' findings were similar to several previous meta-analyses. I strongly recommend a deep discussion and comparison with similar work. A recent overview of meta-analyses regarding HCC management identified the following:

1) 7 meta-analyses compared the outcomes of TACE/TAE versus no active treatment or supportive care. Finally, TACE/TAE should be favored.

2) 3 meta-analyses compared the outcomes of TACE versus TAE. Finally, TACE was similar to TAE in term of OS.

3) 3 meta-analyses compared the outcomes of DEB-TACE versus cTACE. Finally, DEB-TACE was similar to cTACE in the term of tumor response.

4) 1 meta-analysis compared the outcomes of TACE in combination with 3D-CRT versus TACE alone. Finally, the combination therapy was superior to TACE alone in terms of 1- and 3-year survival.

5) 2 meta-analyses compared the outcomes of TACE in combination with radiotherapy versus TACE alone. Finally, TACE plus radiotherapy should be favored in term of OS.

6) 4 meta-analyses compared the outcomes of TACE in combination with sorafenib versus TACE alone. TACE plus sorafenib was not favored in term of OS.

Authors' response: We thank the reviewer for his points. We have expanded our discussion (even though the manuscript is already quite long) with an additional paragraph discussing similarities and agreements of our work (comprehensive network meta-analysis) with other individual direct meta-analytic efforts by citing the relevant papers aforementioned by the reviewer. To our knowledge, the present work combines all currently available randomized data from different treatments/strategies into ***a single unified body of evidence*** that may help guide/transform everyday practice and help change/revise national and international guidelines in the future.

Lines 586-604: "Overall, the findings of the present network meta-analysis are very much in line with the results of several individual direct meta-analyses exploring individual (chemo)-embolization strategies. A recent overview of the major findings of meta-analyses on the management of hepatocellular carcinoma summarized the body of evidence from more than 20 direct meta-analytic reports on embolization therapies for inoperable liver cancer [124]. Seven meta-analyses compared the outcomes of TACE/TAE versus no active treatment or supportive care and overall survival outcomes favoured TACE/TAE [27,33,125]. Another 3 reports compared the outcomes of TACE versus TAE and concluded that there was no survival difference [27,126,127]. Furthermore, 3 reports looked into DEB-TACE versus TACE and found benefit only in terms of tumour response like in the present work [24,128,129]. Four meta-analyses reported outcomes of TACE combined with sorafenib versus TACE alone and again found no survival benefit with the addition of sorafenib [29,130]. Last, there were 3 meta-analyses exploring the combination of TACE with plain external or conformal radiotherapy and also found that combination therapy produced superior survival outcomes [18,124]. The present work corroborates all of

the above in a single model and further raises the combination of TACE and percutaneous tumour ablation as the best treatment option in terms of both local tumour response and overall patient survival.”

8. The potential analyses and conclusions were partially overlapped. The advantages and disadvantages of different work should be discussed.

Authors' response: We believe that we have embarked into already extensive discussion of our findings in comparison to the literature and previous plain meta-analyses. In addition, the manuscript is already long enough for any further comments.

9. The authors identified two RCTs comparing the outcomes of TARE versus TACE. Two papers were published during an interval of 18 years. Over two decades, the understanding of HCC pathogenesis and management has been largely improved. Is the combination of the two studies appropriate? Please provide the difference and similarity in the study design between them.

Authors' response: All studies included in the TARE-radioembolization arm include use of *a beta-emitter* (including ^{131}I-labeled Lipiodol [77,112] or Yttrium-90 microparticles [76,78,109]). Otherwise, details about the study design and characteristics are provided in detail in the supplementary Tables 1 and 2. They all seem to be similar in terms of patient inclusion criteria. However, we do acknowledge that there are always changes in medical practice over the years that may introduce other unknown risk modifiers. In the limitation paragraph the revised manuscript reads:

Lines 648-650: Another limitation is that all 55 studies span 2 decades of medical practice and patient population reflects, as expected, the well-known clinical and anatomical heterogeneity of patients with unresectable HCC.

Reviewer #2: Very informative and properly conducted meta-analysis.

Authors' response: We thank the reviewer for his positive comment.

10. I suggest to add to the bibliography a recent meta-analysis comparing TACE and TAE: Facciorusso A, et al. Transarterial chemoembolization vs bland embolization in hepatocellular carcinoma: A meta-analysis of randomized trials. UEG Journal 2017, in press. http://journals.sagepub.com/toc/ueg/0/0

Authors' response: We have introduced several more references (including the one proposed above) in a whole new discussion paragraph as noted previously.

Lines 586-604: "Overall, the findings of the present network meta-analysis are very much in line with the results of several individual direct meta-analyses exploring individual (chemo)-embolization strategies. A recent overview of the major findings of meta-analyses on the management of hepatocellular carcinoma summarized the body of evidence from more than 20 direct meta-analytic reports on embolization therapies for inoperable liver cancer [124]. Seven meta-analyses compared the outcomes of TACE/TAE versus no active treatment or supportive care and overall survival outcomes favoured TACE/TAE [27,33,125]. Another 3 reports compared the outcomes of TACE versus TAE and concluded that there was no survival difference [27,126,127]. Furthermore, 3 reports looked into DEB-TACE versus TACE and found benefit only in terms of tumour response like in the present work [24,128,129]. Four meta-analyses reported outcomes of TACE combined with sorafenib versus TACE alone and again found no survival benefit with the addition of sorafenib [29,130]. Last, there were 3 meta-analyses exploring the combination of TACE with plain external or conformal radiotherapy and also found that combination therapy produced superior survival outcomes [18,124]. The present work corroborates all of the above in a single model and further raises the combination of TACE and percutaneous tumour ablation as the best treatment option in terms of both local tumour response and overall patient survival."

11. Even though it was published after the literature search period, i strongly suggest to include the recent RCT conducted by the Chicago group on the comparison between TACE and TARE: Y90 Radioembolization Significantly Prolongs Time to Progression Compared With Chemoembolization in Patients With Hepatocellular Carcinoma. Salem R et al, Gastorenterology 2016.

Authors' response: We thank the reviewer for his valuable suggestion. Indeed, we updated our literature search and have introduced **2 more RCTs in the revised analysis (1 in the TARE arm and 1 in the combine TACE and ablation arm)** and we have re-iterated all numerical calculations. Revised results (minor mostly changes without any change in the overall hierarchy, direction and magnitude of the results) and updated figures are presented throughout the revised manuscript.

- *Salem R, Gordon AC, Mouli S, Hickey R, Kallini J, et al. (2016) Y90 Radioembolization Significantly Prolongs Time to Progression Compared With Chemoembolization in Patients With Hepatocellular Carcinoma. Gastroenterology 151: 1155-1163 e1152.*

- *Huang C, Zhuang W, Feng H, Guo H, Tang Y, et al. (2016) Analysis of therapeutic effectiveness and prognostic factor on argon-helium cryoablation combined with transcatheter arterial chemoembolization for the treatment of advanced hepatocellular carcinoma. J Cancer Res Ther 12: C148-C152.*

12. Be careful when including in the analysis the paper by Meyer et al (ref 9) as it is performed not exactly with TACE but with chemotherapy infusion over 15 minutes followed by embolization 4-6 hours later. This aspect should be at least commented in the discussion.

Authors' response: We have acknowledged this aspect of the Meyer et al randomized study in the methods section as follows:

Lines 372-373: **"**In the TACE treated arms, conventional transarterial chemoembolization was performed with a lipiodol emulsion of a single chemotherapy agent (doxorubicin [61,68,70,73-75,78,83,86], or epirubicin [63,66,72,76,80], or cisplatin [9,62,64,67,69,71,77,82], or mitomycin [87], or a combination chemotherapy regimen [65,79,81,84,85,89,93,95,97,99-106], and was most often followed by gelfoam or other particle embolization of the primary feeding vessels**.** Meyer et al. performed cisplatin infusion first followed by particle embolization 4-6 hours later [9]."

13. I am not sure the analysis takes properly into account all the variables, such as tumor stage, treatment scheduled (whether "on demand" or pre-defined), number of sessions, response criteria adopted, and so forth.....

Authors' response: Baseline patient variables and tumour index characteristics of all included studies are provided in Table 1. We performed a random effects meta-regression analysis to search for risk modifiers and predictors that may significantly affect our results. The findings are outlined in supplementary table 5 as below.

**Table 5. Meta-regression analysis with a random effects models (95%CrI)**		
**Endpoint**	**Covariate**	**Regression coefficient**
**Serious adverse events**	Publication year	-0.050 ((-0.278) – 0.134
	Patient age	0.103 ((-0.125) – 0.338
	Male gender	-4.121 ((-16.74) – 8.043
	Child-Pugh A stage	-4.006 ((-13.15) – 3.239
	Multinodular HCC	27.35 (9.329 – 49.66)
	Follow-up period	-0.311 ((-1.295) – 0.507
**Objective response**	Publication year	-0.119 ((-0.268) – 0.010)
	Patient age	0.071 ((-0.057) – 0.195)
	Male gender	0.387 ((-7.583) – 8.740)
	Child-Pugh A stage	-2.883 ((-7.111) – 0.946)
	Multinodular HCC	61.13 (17.76 – 128.4)
	Follow-up period	0.516 ((-0.076) – 1.161
**Patient survival**	Publication year	0.004 ((-0.020) – 0.030)
	Patient age	0.012 ((-0.019) – 0.043)
	Male gender	-0.506 ((-2.643) – 1.584)
	Child-Pugh A stage	-0.002 ((-0.009) – 0.005)
	Multinodular HCC	2.914 ((-0.565) – 6.306)
	Follow-up period	0.049 ((-0.060) – 0.158)

Random effects meta-regression analyses to check for risk modifiers demonstrated only weak non-significant correlations in the majority of the tests. Multinodular HCC was the only variable found to be strongly and significantly related to increased rate of adverse events, as well as of higher rates of radiological response (Supplemental table 5).
